# Anesthesia and analgesia for experimental craniotomy in mice and rats: a systematic scoping review comparing the years 2009 and 2019

**DOI:** 10.3389/fnins.2023.1143109

**Published:** 2023-05-03

**Authors:** Hannah King, Maria Reiber, Vanessa Philippi, Helen Stirling, Katharina Aulehner, Marion Bankstahl, André Bleich, Verena Buchecker, Aylina Glasenapp, Paulin Jirkof, Nina Miljanovic, Katharina Schönhoff, Lara von Schumann, Cathalijn Leenaars, Heidrun Potschka

**Affiliations:** ^1^Institute of Pharmacology, Toxicology, and Pharmacy, Ludwig Maximilian University of Munich, Munich, Germany; ^2^Hannover Medical School, Institute for Laboratory Animal Science, Hanover, Germany; ^3^Office for Animal Welfare and 3Rs, University of Zurich, Zurich, Switzerland

**Keywords:** neuroscience, multimodal analgesia, pain, animal welfare, refinement, surgery, postoperative pain, neurosurgery

## Abstract

Experimental craniotomies are a common surgical procedure in neuroscience. Because inadequate analgesia appears to be a problem in animal-based research, we conducted this review and collected information on management of craniotomy-associated pain in laboratory mice and rats. A comprehensive search and screening resulted in the identification of 2235 studies, published in 2009 and 2019, describing craniotomy in mice and/or rats. While key features were extracted from all studies, detailed information was extracted from a random subset of 100 studies/year. Reporting of perioperative analgesia increased from 2009 to 2019. However, the majority of studies from both years did not report pharmacologic pain management. Moreover, reporting of multimodal treatments remained at a low level, and monotherapeutic approaches were more common. Among drug groups, reporting of pre- and postoperative administration of non-steroidal anti-inflammatory drugs, opioids, and local anesthetics in 2019 exceeded that of 2009. In summary, these results suggest that inadequate analgesia and oligoanalgesia are persistent issues associated with experimental intracranial surgery. This underscores the need for intensified training of those working with laboratory rodents subjected to craniotomies.

**Systematic review registration:**

https://osf.io/7d4qe.

## 1. Introduction

Lack of analgesia and oligoanalgesia (failure to provide adequate analgesia in patients) remains a persistent problem in animal-based research (Richardson and Flecknell, 2005; Stokes et al., 2009; Jirkof, 2017; Flecknell, 2018; Foley et al., 2019). While the type and severity of pain can be anticipated in the context of surgical interventions in experimental animals, scientists often refrain from the application of an adequate analgesia regimen because of concerns about an effect of analgesic drugs on readout parameters (Jirkof, 2017; Peterson et al., 2017; Jirkof and Potschka, 2021). However, it needs to be considered that there is a multitude of analgesic compounds from different drug classes, providing the option to carefully choose the regimen based on the expected interfering effects (Vadivelu et al., 2016; Jirkof, 2017; Jirkof and Potschka, 2021). Moreover, study design can be adjusted to prevent interference, for example with an extension of recovery phases or the inclusion of respective control groups (Jirkof, 2017; Moser, 2020; Jirkof and Potschka, 2021). Most importantly, it should be kept in mind that uncontrolled or insufficiently controlled pain itself, with all its physiological consequences, can have a major impact on research parameters, thereby limiting study quality and rigor of research data (Jirkof, 2017; Peterson et al., 2017; Flecknell, 2018; Jirkof and Potschka, 2021). Thus, with lack of adequate analgesia and oligoanalgesia scientists on one hand violate the 3R principle, which calls for refinement, i.e., a minimization of suffering and pain. On the other hand, they also risk poor data quality, which in turn might affect subsequent planning of animal-based studies.

In neuroscientific research, mice and rats represent the most widely used species. Intracranial implants are often required for recordings, stimulations, or local administration procedures. In this context, reports about undertreatment of pain associated with craniotomy in human patients raise concerns about respective pain management in laboratory rodents with central nervous system (CNS) implants (Dunn et al., 2016; Lutman et al., 2018; Bello et al., 2022). In human patients, oligoanalgesia for neurosurgical procedures is related to concerns about an impact of analgesic drugs on hemorrhage risks and intracranial pressure (Basali et al., 2000; Vadivelu et al., 2016; Bello et al., 2022). Different reviews have discussed to what extent these concerns are justified and how one can optimize the analgesic regimen without an increased risk of postsurgical complications (Dunn et al., 2016; Lutman et al., 2018; Bello et al., 2022). A systematic review from 2017 revealed that there is a significant divergence in studies conducted to explore the efficacy of different regimens, concluding that it is difficult to provide recommendations except for the application of a regional scalp block with local anesthetics (Tsaousi et al., 2017). It has been emphasized that undertreatment of pain in the context of craniotomy procedures can result in prolonged postsurgical pain including headache (Dunn et al., 2016; Lutman et al., 2018; Thapa and Euasobhon, 2018), a risk that has been previously underestimated (Kaur et al., 2000; Gottschalk et al., 2007). Experts of the American College of Laboratory Animal Medicine (ACLAM) Task Force have stated that intracerebral electrode implantation in laboratory rodents is associated with minimal to mild pain only (Kohn et al., 2007). Considering the experience from human patients, this expert opinion might have underestimated the risk of postsurgical pain associated with intracranial procedures.

Stokes et al. (2009) analyzed the literature of analgesic and anesthetic procedures in rodents undergoing surgical interventions. They used a structured multiphase search approach and included 172 papers. While they found improvements from 2000–2001 to 2005–2006, their findings suggest that less than 25% of laboratory rodents receive analgesic drugs in the perioperative phase (Stokes et al., 2009). Since then, several authors and groups have emphasized the need for adequate analgesia in laboratory rodents, have discussed the negative implications of oligoanalgesia for animal welfare and study quality, and have provided guidelines and recommendations for choice of analgesic regimen for different experimental procedures in mice and rats (Gargiulo et al., 2012; Jirkof et al., 2013; Carbone and Austin, 2016; Jirkof, 2017; Flecknell, 2018; Cho et al., 2019; Foley et al., 2019; Jirkof and Potschka, 2021). We hypothesized that these publications and recommendations contributed to an improvement of perioperative pain management in experimental animals and tested this hypothesis with a systematic scoping review. Perioperative anesthetic and analgesic management must be planned as a whole, and the analgesic regimen should take the anesthesia into account. Thus, we decided to assess the complete anesthetic and perioperative pain management approaches in mice and rats undergoing craniotomy described in literature. To allow for completion of our analyses in a reasonable time span, we used a partial literature sample from the years 2009 and 2019. These years allow for analyses of the development during the decade following the report by Stokes et al. (2009).

## 2. Materials and methods

### 2.1. Protocol registration

The study protocol was published on the Open Science Framework database (doi: 10.17605/OSF.IO/G5F6K)^1^ on December 3rd, 2020, prior to starting the screening process. The SYRCLE (Systematic Review Centre for Laboratory animal Experimentation’s) protocol template was used to create the study protocol (de Vries, 2015). The study protocol can also be found in Supplementary Methods 1. This publication follows the PRISMA (Preferred Reporting Items for Systematic Reviews and Meta-Analyses) guidelines; the checklist, including the extension for scoping reviews can be found in Supplementary Methods 2. The review question was: *What are the common approaches to analgesic and anesthetic management for experimental craniotomy in mice and rats?*

### 2.2. Data source and search strategy

A comprehensive search string was constructed for PubMed and used to identify relevant studies (M.R. under supervision from H.P. and C.L.). The search string consisted of two main elements, the investigated population, mice and rats, and the investigated procedure, craniotomy. The search string was constructed to include relevant plural terms, alternative spellings and relevant synonyms for terms, combined with “OR” for each element. The elements population and procedure were combined with “AND.” The final search string and a full overview of all included terms can be found in Supplementary Methods 3.

The final PubMed search was performed on October 19th, 2020. All retrieved studies were saved in a bibliographic reference manager (EndNote™ X9.3.3). The review focused on the years 2009 and 2019, with the latter selected as the most recent completed year prior to the search. The year 2009 was selected so that differences and changes in the approach could be assessed over one decade. The retrieved references from these years were uploaded to Rayyan, an online tool supporting systematic reference screening (Ouzzani et al., 2016).

### 2.3. Study selection

Study screening was performed in two separate phases: first screening of titles and abstracts, then screening of full texts. Inclusion and exclusion criteria were predefined in the study protocol and are listed in Table 1. Both phases were completed by two independent reviewers. Before screening, reviewers were trained with SYRCLE’s e-learning for preclinical systematic reviews and by completing a pre-screened training set of 50 abstracts, of which 95% had to be screened according to requirements.

**TABLE 1:** Exclusion criteria for selection phases.

Exclusion criteria prioritized per selection phase
Title-abstract-screening No English language No mice and/or rats used No craniotomy
Full-text-screening No English language No mice and/or rats used No craniotomy No original *in vivo* data

Exclusion criteria defined in the study protocol. Please refer to Supplementary Methods 1 for the study protocol.

For title and abstract screening, H.K. screened all studies, while the reference set was divided among six reviewers as the second screener (K.A., M.R., M.B., A.G., H.S., P.J.). During title and abstract screening, we included studies published in English language describing craniotomy and/or any type of surgery or other procedure indicating craniotomy in mice and/or rats. For full-text screening, H.K. screened all studies, while the reference set was divided among eight scientists (N.M., H.S., K.A., P.J., M.B., A.G., L.S. and K.S.). During full text screening, we included primary *in vivo* studies, published in English, and describing craniotomy in mice and/or rats. For both screening phases, discrepancies were discussed and solved without the need for a third reviewer.

### 2.4. Data extraction

Data extraction was carried out in two phases. First, comprehensive data were extracted from a random subset of 200 out of 2235 references (Supplementary Tables 1, 2). Studies were initially sorted by year and then by alphabet (for first author) before import to Excel. Using a randomization tool,^2^ a sequence was generated with the length corresponding to the number of included studies from each year. The first 100 numbers from each sequence determined the random sample. Study characteristics to be extracted (Table 2) were predefined in the study protocol. Prior to data extraction, a test extraction of ten studies per year was performed, which served as the basis for the extensions and adjustment of the features to be extracted (H.K. with support of C.L. and H.P). Data were extracted from included references in alphabetical (for first author) order from text and graph, alternating between 2009 and 2019 to prevent confounding by extractor learning effects. The final extraction sheet can be found in Supplementary Tables 1, 2. Relevant information from referencing to another publication were tracked and included for one level i.e., if a referenced publication referenced yet another publication the latter was not retrieved. If information was not provided in the original or directly referenced publication, it was recorded as not reported.

**TABLE 2:** Study characteristics for subset of 200 studies.

Study ID	Study ID, name of pdf-file, first author, title, year of publication, journal, issue, pages, country of origin
Study design characteristics	Number of animals per group (minimum and maximum numbers of animals per group), total number of animals used, background/purpose of craniotomy/field of research
Animal model characteristics	Species, sex, breeder, strain, age at surgery, weight at surgery, housing condition, housing temperature, housing humidity, type of cage, enrichment, light schedule, handling technique
Intervention characteristics	Duration of surgery, survival surgery, how long did animals live after surgery approximately, mortality during surgery, fate of used animals, type of surgical procedure, implantation site, insult size, trepanning size, model used, general anesthesia scheme, compound name/names, route of administration, dosage (mg/kg BW) or concentration (vol.%), how many times administered, administration interval, local anesthesia administered, compound name/names, dosage (mg/kg BW), injection volume (ml/animal), route of administration, timepoint of first administration, administered how many times in total, administration interval, pharmaceutical formulation, analgesic antipyretic agents administered, compound name/names, dosage (mg/kg BW), route of administration, timepoint of first administration, administered how many times in total, administration interval, pharmaceutical formulation, opioid administered, compound name, dosage (mg/kg BW), route of administration, timepoint of first administration, administered how many times in total, administration interval, pharmaceutical formulation, other analgesics used, if so compound name, multimodal approaches used [total number of used compound groups (analgesics and local anesthetics)], other drugs (other than analgesics) used, antibiotic agent used, compound name, route of administration, specific monitoring during surgery, peri-operative care, non-pharmacological measures for pain management, refinement measures
Outcome measures	Assessment of the efficacy of pain alleviating measures, assessment of the analgesic efficacy post-surgery, parameters testing efficacy of pain/stress reducing measures post-surgery, blinding, randomization, power analysis

Study characteristics extracted from subset of 200 studies, defined in the pre-published study protocol and additional study characteristics added during the process of designing the extraction table. Written in black: characteristics specified in the study protocol. Written in gray: characteristics documented in addition to characteristics specified in the study protocol. ID, identity; mg, milligram; BW, body weight; Vol. %, volume percent.

During data extraction, we identified a study that was erroneously included during screening (it had no craniotomy, only subcutaneous electroencephalography electrodes were implanted) (Elmorsy et al., 2019). This study was subsequently excluded, and the next number from the random number sequence of the respective year (the 101*^st^*) was substituted.

The second part of data extraction was restricted to a smaller set of key study characteristics, which was extracted from all included studies (2235 studies) (Supplementary Table 3). This second phase was later added to our original plans and has not been described in our protocol. The characteristics extracted can be found in Table 3. Data were extracted from text and graphs and recorded in the Excel spreadsheet. Relevant information from referencing to another publication was again tracked, as described above.

**TABLE 3:** Study characteristics for all included studies.

Study ID	First author, title, year of publication, journal, issue, pages
Intervention characteristics	General anesthesia scheme, analgesics or local anesthetics administered preoperatively, analgesics or local anesthetics administered intraoperatively, analgesics or local anesthetics administered postoperatively

Study characteristics extracted from all included studies. ID, identity.

Extracted data from a random subset of 5% of all included studies were quality checked by a second reviewer; ten studies for the first part by A.G., 112 for the second part by L.S. The random subsets for quality checking were again generated using random.org.

### 2.5. Data synthesis and analysis

The unit of analysis for this review was the reported analgesic and anesthetic treatment per paper; if a paper described different analgesic or anesthetic procedures for different groups of animals, these were separately included in our analyses. For our review, we separated analgesic-antipyretic agents into analgesic-antipyretic agents with and without an anti-inflammatory effect. In the following, we refer to analgesic-antipyretic agents with an anti-inflammatory effect as non-steroidal anti-inflammatory drugs (NSAIDs) and to analgesic-antipyretic agents without a relevant anti-inflammatory effect as antipyretic analgesics.

Extracted data were tabulated using Excel and Word, and evaluated for the descriptive overview in the section “3. Results.” Data were quantitatively analyzed and plotted using Excel’s Pivot table tool. Graphs and figures were created using Excel and PowerPoint.

Differences between the years were analyzed using Fisher’s exact test and Chi-square test. Two-sided testing, with confidence intervals of 95%, was performed using GraphPad PRISM (GraphPad Software version 5.04, San Diego, USA). Complete information on all calculations performed can be found in Supplementary Methods 4.

## 3. Results

### 3.1. Identification of relevant references

**FIGURE 1:**
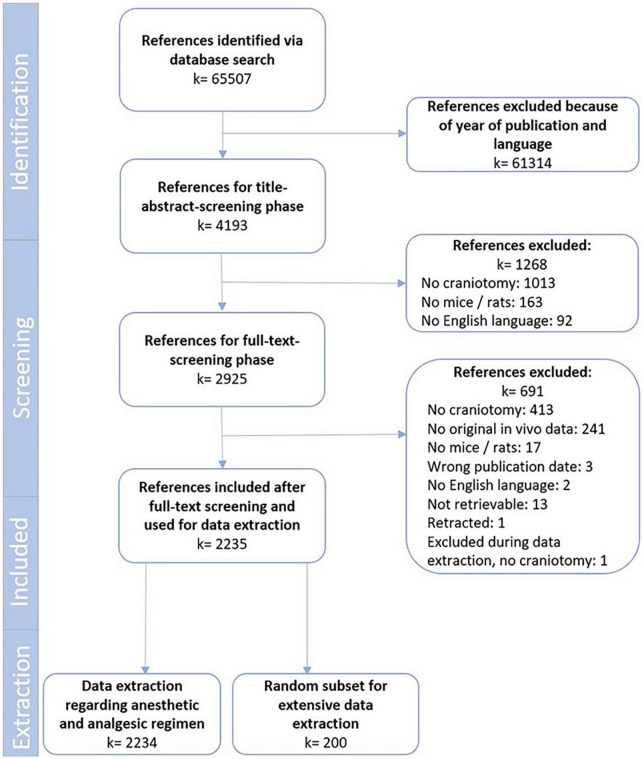
Study flow chart.

Our search identified a total of 65,507 references. After removing all references that were not published in the years of 2009 or 2019 (*k* = 61314), a total of 4,193 references remained for screening. Following title and abstract screening, 2,925 references remained that were subjected to full-text screening. In the end, full-text screening resulted in 2,234 relevant references. Information about key study characteristics was extracted from all these included references; 911 from 2009 and 1323 from 2019 (which described 2,247 treatment regimens). As per protocol, more in depth data extraction was restricted to a random subset of 200 references (100 per year). An overview of the study flow can be found in Figure 1. A list of all included references and extracted parameters can be found in Supplementary Tables 1–3.

### 3.2. Key characteristics from all 2235 references

#### 3.2.1. General study characteristics

In total, 911 references were published in 2009, and 1,324 references were published in 2019, which were included in our review. Seven of the 2019 references described multiple experimental groups in which animals received different compounds for pain management, or one group received a compound, and the other group did not. These groups were separately included in our analyses.

#### 3.2.2. Analgesia in 2009 and 2019 – key characteristics

**FIGURE 2:**
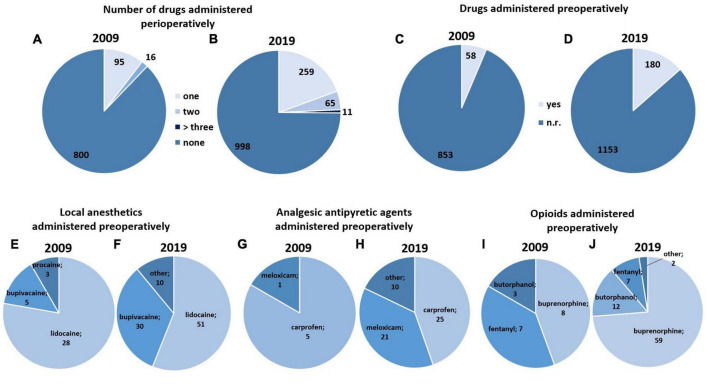
Analgesics and local anesthetics administered perioperatively and preoperatively (*k* = 2244 studies). **(A,B)** Number of drugs (analgesics and local anesthetics) administered perioperatively in 2009 **(A)** and 2019 **(B)**. **(C,D)** Drugs (analgesics and local anesthetics) administered preoperatively in 2009 **(C)** and 2019 **(D)**. **(E,F)** Local anesthetics administered preoperatively in 2009 **(E)** and 2019 **(F)**. Other = drugs used in up to four studies. **(G,H)** Analgesic antipyretic agents administered preoperatively in 2009 **(G)** and 2019 **(H)**. Other = drugs used in up to seven studies. **(I,J)** Opioids administered preoperatively in 2009 **(I)** and 2019 **(J)**. Other = drugs used in up to two studies. “Preoperatively” was defined as all timepoints before skin incision.

The perioperative use of analgesics or local anesthetics was reported in 111 of 911 studies from 2009 and in 335 of 1,333 studies from 2019 (Figures 2A, B). Perioperative administration of analgesics or local anesthetics was more frequently reported in 2019 than 2009 (*X*^2^ (1) = 56.15, *p* < 0.001).

**FIGURE 3:**
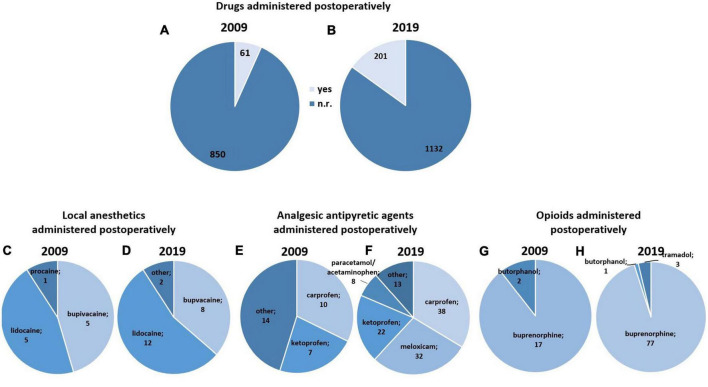
Analgesics and local anesthetics administered postoperatively (*k* = 2244 studies). **(A,B)** Drugs (analgesics and local anesthetics) administered postoperatively in 2009 **(A)** and 2019 **(B)**. **(C,D)** Local anesthetics administered postoperatively in 2009 **(C)** and 2019 **(D)**. Other = drugs used in up to two studies. **(E,F)** Analgesic antipyretic agents administered postoperatively in 2009 **(E)** and 2019 **(F)**. Other = drugs used in up to five studies. **(G,H)** Opioids administered postoperatively in 2009 **(G)** and 2019 **(H)**. “Postoperatively” was defined as all timepoints after finishing surgery and closing of incision.

The number of studies not reporting administration of analgesics or local anesthetics amounted to 87.8% (800/911) in 2009 and 74.8% (998/1333) in 2019.

While 95 of 911 studies from 2009 described the use of a single analgesic drug, only 16 of 911 studies from this year reported using two drugs. There was an increase in the relative number of studies reporting administration of more than one analgesic or local anesthetic from 2009 to 2019 (*X*^2^ (1) = 20.43, *p* < 0.001). 259 out of 1,333 studies described the use of a single drug, 65 out of 1,333 studies described use of two drugs, ten out of 1333 studies described use of three drugs, and one out of 1,333 studies described use of four drugs (Figures 2A, B). Please note that the number of drugs provided refers to the type of drug administered, and not the number of drug administrations, i.e., if the same drug was repeatedly applied, (e.g., once before and once after surgery), it was counted as one drug.

In 2009, two of 911 studies reported administering either an analgesic or local anesthetic, but the authors did not report which substance was used. The same applied to seven of 1,333 studies from 2019.

##### 3.2.2.1. Preoperative analgesia - key characteristics

The number of studies reporting administration of analgesics or local anesthetics before surgery increased from 2009 to 2019 (*X*^2^ (1) = 21.38, *p* < 0.001). In the studies describing administration of local anesthetics prior to surgery, lidocaine was the most commonly used local anesthetic (2009: 28/911 studies; 2019: 51/1333 studies), and bupivacaine the second most commonly used (2009: 5/911 studies; 2019: 30/1333 studies) (Figures 2E, F). The comparison between data sets from 2009 and 2019 indicated an increase in the use of local anesthetics before surgery (*X*^2^ (1) = 7.85, *p* = 0.005).

The two most common NSAIDs administered before surgery in both years of interest were carprofen (2009: 5/911 studies; 2019: 25/1333 studies) and meloxicam (2009: 1/911 studies; 2019: 21/1333 studies) (Figures 2G, H). The reporting of preoperative administration of NSAIDs reached higher levels in 2019 than in 2009 (*X*^2^ (1) = 23.31, *p* < 0.001). One of 1333 studies in 2019 reported use of the antipyretic analgesic metamizole/dipyrone.

The most common opioids administered before surgery were buprenorphine (2009: 8/911 studies; 2019: 59/1333 studies), fentanyl (2009: 7/911 studies; 2019: 7/1333 studies), and butorphanol (2009: 3/911 studies; 2019: 12/1333 studies) (Figures 2I, J). Preoperative opioid administration significantly increased from 2009 to 2019 (*X*^2^ (1) = 17.53, *p* < 0.001).

Full information on all drugs used for pain management before surgery is provided in Table 4 and Supplementary Table 3.

**TABLE 4:** Analgesics and local anesthetics reported in all included studies in 2009 and 2019.

Drug	2009	2009	2019	2019
	Preoperatively	Postoperatively	Preoperatively	Postoperatively
Acetaminophen	n.a.	2	n.a.	6
Acetaminophen, bupivacaine	n.a.	1	n.a.	n.a.
Bupivacaine	5	4	16	5
Bupivacaine, carprofen	n.a.	n.a.	n.a.	1
Bupivacaine, ketoprofen	n.a.	n.a.	2	n.a.
Bupivacaine, meloxicam	n.a.	n.a.	2	1
Buprenorphine	8	15	33	64
Buprenorphine, acetaminophen	n.a.	n.a.	n.a.	1
Buprenorphine, bupivacaine	n.a.	n.a.	6	n.a.
Buprenorphine, carprofen	n.a.	n.a.	5	4
Buprenorphine, ketoprofen	n.a.	n.a.	1	n.a.
Buprenorphine, meloxicam, bupivacaine	n.a.	n.a.	1	1
Butorphanol	3	2	12	1
Carprofen	4	10	14	30
Carprofen, lidocaine	1	n.a.	4	1
Diclofenac	n.a.	1	n.a.	n.a.
Fentanyl	6	n.a.	6	n.a.
Fentanyl, buprenorphine	n.a.	n.a.	1	n.a.
Fentanyl, lidocaine	1	n.a.	n.a.	n.a.
Flunixin	n.a.	3	1	3
Flunixin, meglumine	n.a.	2	n.a.	2
Ibuprofen	n.a.	3	n.a.	3
Ibuprofen, acetaminophen	n.a.	n.a.	n.a.	1
Ketoprofen	n.a.	6	4	22
Ketoprofen, buprenorphine	n.a.	1	n.a.	n.a.
Lidocaine	26	4	30	6
Lidocaine, bupivacaine	n.a.	n.a.	2	n.a.
Lidocaine, buprenorphine	n.a.	1	9	1
Lidocaine, carprofen, buprenorphine	n.a.	n.a.	n.a.	2
Lidocaine, marcaine, carprofen	n.a.	n.a.	1	n.a.
Lidocaine, meloxicam	n.a.	n.a.	2	2
Lignocaine	n.a.	n.a.	n.a.	1
Marcaine	n.a.	n.a.	1	n.a.
Marcaine, carprofen	n.a.	n.a.	1	n.a.
Meloxicam	1	2	11	23
Meloxicam, acetaminophen	n.a.	n.a.	n.a.	1
Meloxicam, bupivacaine, lidocaine	n.a.	n.a.	1	n.a.
Meloxicam, buprenorphine	n.a.	n.a.	3	4
Meloxicam, buprenorphine, lidocaine	n.a.	n.a.	1	n.a.
Metamizole	n.a.	1	1	4
n.a.	853	850	1153	1132
n.r.	n.a.	2	n.a.	7
Piritramide	n.a.	n.a.	2	n.a.
Prilocaine	n.a.	n.a.	1	n.a.
Prilocaine, lidocaine	n.a.	n.a.	1	n.a.
Procaine	3	1	1	n.a.
Ropivacaine	n.a.	n.a.	3	n.a.
Ropivacaine, lidocaine	n.a.	n.a.	n.a.	1
Ropivacaine, metamizole	n.a.	n.a.	1	n.a.
Tramadol	n.a.	n.a.	n.a.	3

Analgesics and local anesthetics reported in all included studies from 2009 (k = 911) and 2019 (k = 1333). Two studies from each year reported an intraoperative administration of analgesics or local anesthetics (2009: bupivacaine, fentanyl; 2019: buprenorphine, meloxicam). N.a., not applicable, this drug was not administered; n.r., drug administered but no information on substance used was provided; preoperatively, all timepoints before skin incision; postoperatively, all timepoints after end of surgery; intraoperatively, all timepoints between skin incision and end of surgery. Summary measures of tabulated analgesics and local anesthetics can also be found in Figures 2, 3.

##### 3.2.2.2. Intraoperative analgesia - key characteristics

Only two studies from each year reported administering analgesics or local anesthetics during surgery. The drugs administered during surgery comprised bupivacaine and fentanyl (one study each of 911 studies in 2009), as well as buprenorphine and meloxicam (one study each of 1333 studies in 2019) (Table 4). Please note that the information provided here focused on administration during the actual surgical procedure [infusion or injection from the first incision through wound closure by primary intention (edges of wound are brought and held together by e.g., suturing, gluing, or stapling)]. In this context, it should be remembered that the duration of action of some drugs administered before surgery extends into the surgical phase.

##### 3.2.2.3. Postoperative analgesia - key characteristics

The use of local anesthetics or analgesics after surgery was reported in 61 of 911 studies in 2009 and 201 of 1333 studies in 2019 (Figures 3A, B). The most commonly used local anesthetics in 2009 were bupivacaine (2009: 5/911 studies; 2019: 8/1333 studies) and lidocaine (2009: 5/911 studies; 2019: 12/1333 studies) (Figures 3C, D). We did not observe a significant change in the postoperative use of local anesthetics from 2009 to 2019 (*X*^2^ (1) = 0.46, *p* = 0.498). Among NSAIDs, the most commonly used substances in 2009 and 2019 were carprofen (2009: 10/911 studies; 2019: 38/1333 studies), ketoprofen (2009: 7/911 studies; 2019: 22/1333 studies, and meloxicam (2009: 2/911 studies; 2019: 32/1333 studies) (Figures 3E, F). Acetaminophen/paracetamol was the most frequently reported antipyretic analgesic in both years (2009: 3/911 studies; 2019: 8/1333 studies). One of 911 studies in 2009 and four of 1333 studies in 2019 reported use of metamizole/dipyrone. We found evidence for a significant increase in the postoperative use of NSAIDs from 2009 to 2019 (*X*^2^ (1) = 17.30, *p* < 0.001), but no change in the postoperative use of antipyretic analgesics (*X*^2^ (1) = 1.04, *p* = 0.308). Postsurgical administration of opioids comprised the use of buprenorphine (2009: 17/911 studies; 2019: 77/1333 studies), butorphanol (2009: 2/911 studies; 2019: 1/1333 study) and tramadol (2019: 3/1333 studies) (Figures 3G, H). The postoperative administration of opioids increased from 2009 to 2019 (*X*^2^ (1) = 19.32, *p* < 0.001).

For an overview of all drugs administered after surgery as reported in the 2009 and 2019 references, please see Table 4 and Supplementary Table 3.

#### 3.2.3. Multimodal approaches - key characteristics

Multimodal approaches (use of >2 substances) for perioperative pain management were reported in 16 of 911 studies in 2009 (two substances) and 76 of 1,333 studies (two substances: 65/1333 studies; three substances: 10/1333 studies; four substances: 1/1333 studies) in 2019 (Figures 2A, B and Table 4). The reporting of multimodal approaches increased from 2009 to 2019 (*X*^2^ (1) = 20.43, *p* < 0.001).

In 2009, six of 911 studies reported administering a local anesthetic in combination with an opioid. Another four of 911 studies reported administering an opioid and an NSAID, whereas two of 911 studies reported the administration of a local anesthetic and an NSAID and one of 911 studies reported the administration of a local anesthetic and an antipyretic analgesic. Two of 911 studies reported administering two different NSAIDs and one study reported administering two opioids.

In 2019, the most commonly reported combinations of two substances were a local anesthetic with an NSAID (24/1333 studies), followed by opioid plus NSAID (16/1333 studies), and local anesthetic plus opioid (16/1333 studies). Two further studies reported the administration of an antipyretic analgesic and an NSAID, while another study reported the administration of an antipyretic analgesic and an opioid. The majority of studies reporting the administration of three substances (7/10 studies) described using a local anesthetic, an NSAID and an opioid.

#### 3.2.4. Anesthesia in 2009 and 2019 - key characteristics

**FIGURE 4:**
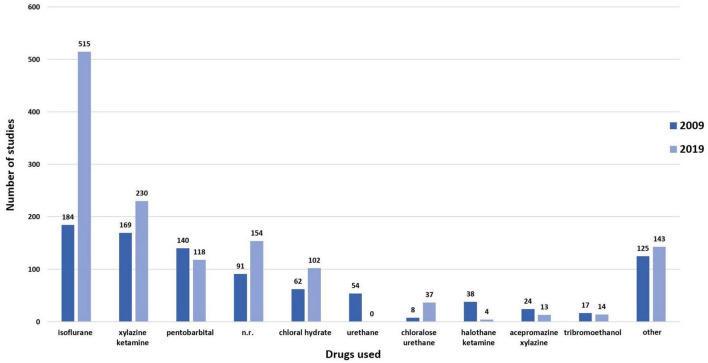
Anesthetics drugs (*k* = 2241 studies). Administration of anesthetic drugs in 2009 (*k* = 911) and 2019 (*k* = 1330). N.r., not reported. Note that one of the included papers from 2019 did not use anesthesia; the surgeries were performed on conscious animals.

The administration of anesthetic drugs was reported in all 912 studies in 2009 and in 1,329 of 1,330 studies in 2019 (Figure 4). For these analyses, experimental groups receiving different anesthetics, described within publications, were separately analyzed; the treatment regime was our unit of analysis. Because one reference in 2009 described two experimental groups, and five references in 2019 described two or more experimental groups, we now refer to 912 studies in 2009 and 1,330 studies in 2019.

In 2009, three of 912 studies reported inducing hypothermia in neonatal pups instead of using an anesthetic drug. Another study reported using either hypothermia in neonatal pups or a mixture of ketamine and xylazine. In 2019, two of 1330 publications described using hypothermia in neonatal pups. One additional manuscript reported not administering any substances to induce anesthesia; the animals remained conscious for the duration of the surgery. No explanation was provided as to why the animals remained conscious during the surgical procedure. In 2009, 91 of 912 studies did not report which drug was used to induce general anesthesia. In 2019, this was the case for 154 of 1330 studies.

The most commonly used drug to induce general anesthesia in both years of interest was isoflurane. It was administered in 184 of 912 studies in 2009 and in 515 of 1330 studies in 2019 (Figure 4). In both years, isoflurane was used in combination with ketamine and xylazine in several studies (2009: 7/912 studies; 2019: 17/1330 studies). Use of isoflurane was more frequently reported in 2019 than in 2009 (*X*^2^ (1) = 85.65, *p* < 0.001). Moreover, there was a general increase in the use of inhalational anesthesia compared to the use of injectable anesthesia (*X*^2^ (1) = 22.70, *p* < 0.001). Full information on all calculations is provided in Supplementary Methods 4.

The combination of ketamine and xylazine was the second most commonly used form for induction of general anesthesia. Use of this combination was reported in 169 of 912 studies in 2009 and 230 of 1330 studies in 2019 (Figure 4). There was no significant difference between 2009 and 2019 (*X*^2^ (1) = 0.33, *p* = 0.564). Full information on all calculations is provided in Supplementary Methods 4. Ketamine and xylazine were also combined with further drugs, the most frequently reported combination being ketamine, xylazine, and acepromazine (2009: 24/912 studies; 2019: 13/1330 studies).

The third most commonly used substance for general anesthesia in both years was pentobarbital (2009: 140/912 studies; 2019: 118/1330 studies). There was a significant decrease in the use of pentobarbital from 2009 to 2019 (*X*^2^ (1) = 21.67, *p* < 0.001). Combinations of pentobarbital with other substances were reported by 30 of 912 studies in 2009 and twelve of 1330 studies in 2019. Drugs used for combination with pentobarbital comprised chloral hydrate, diethyl ether, isoflurane, ketamine, and xylazine.

Other drugs used to induce anesthesia included chloral hydrate (2009: 62/912 studies; 2019: 102/1330 studies), halothane (2009: 38/911 studies; 2019: 4/1330 studies), urethane (2009: 54/912 studies) urethane in combination with chloralose (2009: 8/912 studies; 2019: 37/1330 studies), and tribromoethanol (2009: 17/912 studies; 2019: 7/1330 studies).

**TABLE 5:** Type of anesthesia reported in all included studies from 2009 and 2019.

Type of anesthesia	Studies in 2009 (*k* = 912)	Studies in 2019 (*k* = 1330)
Alfaxalon, diazepam	n.a.	1
Amobarbital	n.a.	1
Benzodiazepam/ketamine	n.a.	1
Chloral hydrate	62	102
Chloral hydrate/gallamine triethiodide	1	n.a.
Chloral hydrate/isoflurane/ketamine	1	n.a.
Chloralose	n.a.	1
Chloralose/halothane	1	n.a.
Desflurane	1	n.a.
Diethyl ether	1	1
Diethyl ether/pentobarbital	1	n.a.
Ethanol	n.a.	1
Ethanol/pentobarbital/chloral hydrate/propylene glycol	2	3
Ether	9	2
Ether/sevoflurane	1	n.a.
Fluanisone	1	n.a.
Halothane	38	4
Halothane/urethane	1	2
Hexenal OR chloral hydrate	1	n.a.
Hypothermia	3	2
Hypothermia/ketamine/xylazine	1	n.a.
Isoflurane	184	515
Isoflurane/chloralose	n.a.	1
Isoflurane/pentobarbital	3	n.a.
Isoflurane/dexmedetomidine	n.a.	1
Isoflurane/ketamine/medetomidine /acepromazine	n.a.	1
Isoflurane/urethane/ketamine/ xylazine	n.a.	1
Isoflurane/urethane/chloralose	1	n.a.
Isoflurane/urethane/ pancuronium bromide	1	n.a.
Isoflurane OR urethane	1	n.a.
Ketamine	5	7
Ketamine/acepromazine	3	n.a.
Ketamine/benzodiazepine	n.a.	1
Ketamine/chlorpromazine	3	n.a.
Ketamine/climazolam	1	n.a.
Ketamine/dexmedetomidine	n.a.	1
Ketamine/diazepam	3	3
Ketamine/medetomidine	13	12
Ketamine/medetomidine/ diazepam	2	n.a.
Ketamine/medetomidine/ isoflurane	n.a.	2
Ketamine/midazolam	1	n.a.
Ketamine/xylazine	169	230
Ketamine/xylazine/acepromazine	24	13
Ketamine/xylazine/acepromazine/ isoflurane	1	1
Ketamine/xylazine/chloralose	n.a.	1
Ketamine/xylazine/halothane	1	n.a.
Ketamine/xylazine/isoflurane	7	17
Ketamine/xylazine/pentobarbital	4	n.a.
Ketamine/xylazine OR isoflurane	1	1
Ketamine/xylazine OR pentobarbital OR tribromoethanol OR chloralose OR isoflurane	n.a.	1
Medetomidine/midazolam	1	9
Medetomidine/midazolam/ isoflurane	n.a.	1
Medetomidine/midazolam OR ketamine/xylazine OR pentobarbital	n.a.	1
Medetomidine/tiletamine/ zolazepam	1	n.a.
Methylbutanol/tribromoethanol	1	n.a.
Midazolam/fluanisone	4	4
Midazolam/isoflurane	n.a.	1
n.r.	91	154
None	n.a.	1
Pentobarbital	140	118
Pentobarbital/chloral hydrate	12	11
Pentobarbital/fluanisone	1	n.a.
Pentobarbital/halothane	2	n.a.
Pentobarbital/ketamine	4	1
Pentobarbital/xylazine OR isoflurane OR urethane	n.a.	1
Pentobarbital OR isoflurane	n.a.	1
Phenobarbital	1	n.a.
Propofol	n.a.	1
Sevoflurane	3	7
Sevoflurane/halothane	n.a.	2
Sevoflurane/ketamine/xylazine	n.a.	1
Thiobutabarbital	2	1
Thiopental	4	5
Thiopental/chloral hydrate/pentobarbital	1	n.a.
Tiletamine/xylazine	n.a.	1
Tiletamine/zolazepam	4	8
Tribromoethanol	17	7
Tribromoethanol/isoflurane	1	14
Tubocurarine	1	n.a.
Urethane	54	n.a.
Urethane/chloralose	8	37
Urethane/isoflurane	2	6
Urethane/ketamine	n.a.	2
Urethane/ketamine/xylazine	2	1
Urethane/medetomidine/ketamine	1	1
Xylazine	1	n.a.
Zolazepam/xylazine	n.a.	1

Type of anesthesia reported in all included studies from 2009 (k = 912) and 2019 (k = 1330); n.r., anesthetic drug not reported; None, no anesthesia reported; n.a., not applicable, this drug or combination of drugs were not administered. Summary measures of tabulated anesthetics can also be found in Figure 4.

Full information on all anesthetic drugs used is provided in Table 5 and Supplementary Table 3.

#### 3.2.5. Anesthesia and analgesia in combination - key characteristics

Conclusions about pain management need to consider that some drugs used for general anesthesia can exert analgesic effects themselves (e.g., ketamine, alpha-sympathomimetic drugs including xylazine, and urethane). Thus, we additionally analyzed the combination of specific drugs used for general anesthesia with analgesics or local anesthetics.

In 2009, 184 of 912 studies reported administering isoflurane as the only drug used to induce general anesthesia. Here, 35 studies described the additional perioperative administration of one or more drugs used for pain management. The list of added drugs comprised: lidocaine, bupivacaine, meloxicam, carprofen, ketoprofen, ibuprofen, flunixin, acetaminophen/paracetamol, buprenorphine, and butorphanol. The remaining 149 of 184 studies did not mention administering any analgesics or local anesthetics.

For 2019, 515 of 1330 studies reported administering isoflurane as the only drug used to induce general anesthesia. Here, 188 studies reported the additional perioperative administration of one or more analgesics or local anesthetics (including lidocaine, bupivacaine, ropivacaine, marcaine, meloxicam, carprofen, ketoprofen, ibuprofen, flunixin, acetaminophen/paracetamol, metamizole/dipyrone, buprenorphine, butorphanol, fentanyl, and piritramide). The remaining 327 of 515 studies using isoflurane as the only substance to induce general anesthesia did not mention administering any additional analgesics or local anesthetics.

In 2009, 169 of 912 studies reported use of a ketamine and xylazine combination to induce general anesthesia. Twenty-five of these studies reported the additional perioperative administration of one or more analgesics or local anesthetics (including lidocaine, procaine, carprofen, ketoprofen, metamizole/dipyrone, buprenorphine, and butorphanol). In 2019, 230 of 1330 studies reported use of a combination of ketamine and xylazine to induce general anesthesia. Sixty-three of these studies reported the additional perioperative administration of one or more analgesics or local anesthetics (including lidocaine, bupivacaine, procaine, ropivacaine, carprofen, meloxicam, ketoprofen, flunixin, metamizole/dipyrone, buprenorphine, butorphanol, fentanyl, and tramadol).

Among studies using pentobarbital to induce anesthesia, in 2009, three of 140 studies reported additional pre- and/or postoperative analgesia. In 2019, ten of 118 studies described pre- or postoperative administration of an analgesic.

In 2009, 62 of 912 studies reported using chloral hydrate to induce anesthesia. Only three of these studies reported an additional pre- or postoperative use of an analgesic or local anesthetic. In 2019, five of 102 studies using chloral hydrate described additional pre- and/or postoperative administration of an analgesic or local anesthetic.

Urethane was used in 54 of 912 studies in 2009. Five of these studies reported the administration of an analgesic or local anesthetic before surgery. In 2019, four studies from a total of 37 studies using urethane described the use of an analgesic drug before surgery.

Among studies using halothane (2009: 38/912 studies; 2019: 4/1330 studies) to induce anesthesia, in 2009, three studies reported administering an analgesic or local anesthetic before or following surgery. In 2019 one of the studies described administering two local anesthetics before surgery.

Please note that information on drugs used to induce anesthesia reported in 24 or fewer studies were not considered in this paragraph. Full information about all drugs and combinations used can be found in Supplementary Table 3.

### 3.3. Detailed data for the random subset of 200 references

For the purpose of feasibility, more detailed information was only collected from a random subset of 200 references (100 references/year). The additional information described below comprises study, animal and intervention characteristics, treatment protocols, outcome measures and risk of bias parameters. These additional data for the random subset allowed us to get an impression of dosage and frequency of the described anesthetics and analgesics.

Full information on all extracted parameters is provided in Supplementary Tables 1, 2.

#### 3.3.1. General study-/animal characteristics – detailed analysis of random subset of 200 studies

**FIGURE 5:**
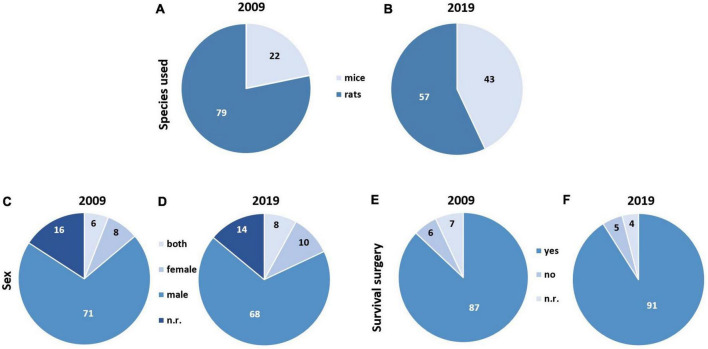
Animal model characteristics: species and sex and intervention characteristic survival surgery in the subset of 200 studies. **(A)** Studies using mice and rats in 2009 (*k* = 101 studies). **(B)** Studies using mice and rats in 2019 (*k* = 100 studies). **(C,D)** Studies reporting sex in 2009 (**C**; *k* = 101 studies) and 2019 (**D**; *k* = 100 studies). **(E,F)** Studies reporting if animals survived surgery in 2009 (**E**; *k* = 100 studies) and 2019 (**F**; *k* = 100 studies). n.r., not reported.

From the subset of 200 studies, for 2009, 22 of 100 studies using mice and 79 of 100 studies using rats were identified. In one reference from 2009 (Merkler et al., 2009), both mice and rats were used. In 2019, the number of studies using mice was 43 of 100, and 57 of 100 used rats (Figures 5A, B). The list of studies using mice indicated that the most commonly used strain was C57BL/6 in both years of interest (2009: 4/100 studies; 2019: 13/100 studies). Further information regarding the substrains were generally not reported in these studies. An exception was the C57BL/6J strain, which was specifically mentioned in selected studies from both years (2009: 2/100 studies; 2019: 5/100 studies). The list of studies using rats indicated that the most commonly used strain was Sprague–Dawley in both years of interest (2009: 36/100 studies; 2019: 33/100 studies). The second most commonly used strain was Wistar in both years (2009: 24/100 studies; 2019: 15/100 studies). Further information on strain specificity was not reported.

In both years, the majority of studies focused on male animals (2009: 71/101 studies; 2019: 68/100 studies). Female animals were used much less frequently (2009: 8/101 studies; 2019: 10/100 studies) (Figures 5C, D). Studies reporting both sexes were relatively uncommon in both years (2009: 6/101 studies; 2019: 8/100 studies). In 2009, 16 of 101 studies did not report the sex of the used animals; in 2019 this was 14 of 100 studies. Please note that we here refer to 101 studies from 2009, since one reference reported using mice and rats, but only provided information about the sex for rats.

For detailed information on further animals’ characteristics, housing, and husbandry see Supplementary Tables 1, 2.

For the purpose of this study, country of origin was defined as the country of the institute associated with the first author at the time of publication. The majority of the studies were from the United States of America (2009: 37/100 studies; 2019: 28/100 studies). There were no major differences in analgesic or anesthetic approaches between countries or continents. Full information on all countries of origin can be found in Supplementary Tables 1, 2.

#### 3.3.2. Intervention characteristics – detailed analysis of random subset of 200 studies

Deep electrode implantations were the most common surgical procedure reported in both years of interest (2009: 19/102 studies; 2019: 17/102 studies). Please note that we here refer to 102 studies from each year, since in two references from each year, two groups with different surgical procedures were reported.

For both years, the second most commonly reported surgical intervention was the transient insertion of an intracerebral injection cannula (2009: 16/102 studies; 2019: 16/102 studies). For further information on other surgical procedures, see Supplementary Tables 1, 2.

There were no major differences between years in analgesic or anesthetic approaches for different surgical interventions. Almost all studies from both years reported survival surgeries (2009: 87/100 studies; 2019: 91/100 studies) (Figures 5E, F). Further studies either reported experiments during surgery, killing the animals directly afterward, or survival for less than 24 h post-surgery.

Specific monitoring during surgery was reported in 25 of 100 studies from 2009 and 24 of 100 studies from 2019. Monitoring during surgery comprised any monitoring measure, such as vital parameters (heart rate, breathing, blood pressure, body temperature, blood oxygen), reflexes (e.g., corneal reflex, pedal reflex), movements of the animal, and ECG (Electrocardiogram).

Perioperative care was reported for 23 of 100 studies from 2009 and 30 of 100 studies from 2019. Perioperative care was defined as any measure during surgery aimed at improving animal wellbeing or the outcome of surgery. These measures comprised maintaining of body temperature and application of eye ointment.

Non-pharmacological measures for pain management (e.g., cooling of incision to aid healing, massage, physical therapy) were reported neither for the year 2009 nor for 2019.

#### 3.3.3. Pain-related outcome measures – detailed analysis of random subset of 200 studies

None of the studies published in 2009 reported an assessment of analgesic efficacy. In 2019, only one of 100 studies (Stanchi et al., 2019) reported assessing the analgesic efficacy post-surgery. Here, the well-being of mice was assessed daily by inspecting the animals. Parameters testing the efficacy of pain-reducing measures post-surgery, such as the mouse grimace score, were not reported within the sample.

#### 3.3.4. Analgesia – detailed analysis of random subset of 200 studies

The use of analgesics or local anesthetics was reported in twelve of 100 studies in 2009 and 33 of 103 studies in 2019. For the subset of 200 studies, data regarding characteristics of the analgesic and anesthetic treatment protocol, e.g., dosage, administration interval, route of application, were extracted.

##### 3.3.4.1. Local anesthetics

In 2009, six studies from the subset of 100 reported using local anesthetics (see Table 6). The substances used were lidocaine (2/100 studies), procaine (2/100 studies), and bupivacaine (1/100 studies). One study did not report which substance was used. Information about dosing was not provided in the majority (4/6 studies) of studies (Table 6 and Supplementary Tables 1, 2). For all local anesthetics, a single application was reported.

**TABLE 6:** Studies reporting use of local anesthesia from 2009 and 2019 included in the subset of 200 studies.

References	Species used	Drug	Administration route	Dosage (mg/kg)	Injection volume (ml/ animal)	Timepoint of first administration	Administered how many times in total	Administration interval (h post first administration)
Griesbach et al., 2009	Rats	Bupivacaine	s.c.	0.25	n.r.	Postsurgically	1	n.a.
Hart et al., 2009	Rats	Procaine	i.p.	300	0.3	Postsurgically	1	n.a.
Meeren et al., 2009	Rats	Lidocaine	s.c.	n.r.	n.r.	Presurgically	1	n.a.
Mohammadi et al., 2009	Rats	Lidocaine	s.c.	n.r.	0.2	Presurgically	1	n.a.
Schei et al., 2009	Rats	n.r.	n.r.	n.r.	n.r.	n.a.	1	n.a.
Tchekalarova et al., 2009	Rats	Procaine	n.r.	n.r.	n.r.	Presurgically	1	n.a.
Aldehri et al., 2019	Rats	Lidocaine	s.c.	n.r.	n.r.	Presurgically	1	n.a.
Bazzu et al., 2019	Mice	Lidocaine	n.r.	n.r.	n.r.	Presurgically	n.r.	n.r.
Bertoglio et al., 2019	Rats	Lidocaine	Applied topically	n.r.	n.r.	Postsurgically	1	n.a.
Bukhtiyarova et al., 2019	Mice	Bupivacaine + lidocaine	s.c.	n.r.	n.r.	Presurgically	1	n.a.
Christiaen et al., 2019	Rats	Lidocaine	Applied topically	n.r.	n.r.	Postsurgically	1	n.a.
Colangeli et al., 2019	Rats	Lidocaine	s.c.	n.r.	n.r.	n.r.	n.r.	n.r.
Farakhor et al., 2019	Rats	Lidocaine	n.r.	n.r.	n.r.	n.r.	n.r.	n.r.
Levata et al., 2019	Mice	Lidocaine + prilocaine	Applied topically	n.r.	n.a.	Presurgically	1	n.a.
Moller et al., 2019	Rats	Bupivacaine	Applied topically	n.r.	n.r.	Presurgically	1	n.a.
Russell et al., 2019	Rats	n.r.	n.r.	n.r.	n.r.	n.a.	1	n.a.
Sharma et al., 2019	Rats	Lignocaine	Applied topically	n.r.	n.r.	Postsurgically	4	24
Shaver et al., 2019	Rats	Bupivacaine	s.c.	n.r.	n.r.	Presurgically	1	n.a.
Slezia et al., 2019	Mice	Ropivacaine	s.c.	n.r.	0,005	Presurgically	1	n.a.
Wang et al., 2019f	Mice	Lidocaine	Applied topically	n.r.	n.r.	Postsurgically	4	24

Information on studies reporting use of local anesthetics (5 studies in 2009; 14 studies in 2019) included in the subset of 200 studies. n.r., not reported, parameter was not reported; n.a., not applicable, extraction of this parameter was not feasible; i.p., intraperitoneal; i.m., intramuscular; s.c., subcutaneous.

In 2019, 14 of 103 studies reported administration of a local anesthetic (see Table 6), with seven studies reporting administration before, four studies after surgery, and three studies not providing information on the time of administration. The most used local anesthetic was lidocaine, with eight of 103 studies reporting its use, whereas the second most used drug was bupivacaine, being used in two of 103 studies. Further local anesthetics used were ropivacaine, lignocaine, and prilocaine, all three substances being used in one study each. One study did not provide information on which local anesthetic was used and one study reported administering a combination of bupivacaine and lidocaine. None of the studies provided dosing information. Most studies (12/14) reported administering the local anesthetic once. Only two studies reported multiple applications with either lidocaine or lignocaine administered four times every 24 h.

For complete information on all drugs used, please refer to Table 6 and Supplementary Tables 1, 2.

##### 3.3.4.2. Analgesic antipyretic agents administered

The administration of analgesic antipyretic agents was reported in five of 100 studies from 2009 and 16 of 103 studies from 2019 (see Table 7). Most studies (4/5) in 2009 and all (Kaur et al., 2000) studies in 2019 reported use of NSAIDs. The remaining study in 2009 used an antipyretic analgesic. In one reference from 2019, two experimental subgroups were described with animals receiving either an NSAID or an opioid.

**TABLE 7:** Studies reporting use of analgesic antipyretic agents from 2009 (*k* = 100) and 2019 (*k* = 100) included in the subset of 200 studies.

References	Species used	Drug	Administration route	Dosage (mg/kg)	Timepoint of first administration	Administered how many times in total	Administration interval (h post first administration)
Gurevicius et al., 2009	Mice	Carprofen	i.p.	5	Postsurgically	1	n.a.
Magloire and Cattarelli, 2009	Rats	Acetaminophen	*per os*	n.r.	Postsurgically	n.a.	n.a.
Schei et al., 2009	Rats	Flunixin	s.c.	1,1	Intrasurgically	1	n.a.
Shultz et al., 2009	Rats	Ketoprofen	s.c.	n.r.	Postsurgically	1	n.a.
Topchiy et al., 2009	Rats	Flunixin	n.r.	1,1	Postsurgically	3	24
Bazzu et al., 2019	Mice	Meloxicam	s.c.	1	Presurgically	1	n.a.
Christiaen et al., 2019	Rats	Meloxicam	s.c.	1	Postsurgically	2	n.a.
Farakhor et al., 2019	Rats	Meloxicam	n.r.	0,2	Postsurgically	4	24h post-surgery
Kaefer et al., 2019	Rats	Meloxicam	n.r.	5	Intrasurgically	1	n.a.
Mastrella et al., 2019	Mice	Carprofen	i.p.	2 to 4	Presurgically	7	n.a.
Mohammad et al., 2019	Rats	Ketoprofen	s.c.	5	Presurgically	4	n.a.
Moller et al., 2019	Rats	Meloxicam	s.c.	1	Presurgically	2	24h post-surgery
O’Brien et al., 2019	Mice	Carprofen	n.r.	n.r.	Postsurgically	n.r.	n.r.
Park et al., 2019	Mice	Meloxicam + acetaminophen	Meloxicam s.c. acetaminophen *per os*	Meloxicam 1 acetaminophen n.r.	Postsurgically	1	n.a.
Sa et al., 2019	Rats	Ketoprofen	s.c.	n.r.	Postsurgically	1	n.a.
Shaver et al., 2019	Rats	Ketoprofen	s.c.	5	Presurgically	1	n.a.
Souza et al., 2019	Rats	Ketoprofen	s.c.	3 to 5	Postsurgically	4	n.a.
Sun et al., 2019	Mice	Ketoprofen	i.p.	5	Presurgically	1	n.a.
Szonyi et al., 2019	Mice	Meloxicam	i.p.	0,03 to 0,05	Postsurgically	1	n.a.
Wang et al., 2019f	Mice	Carprofen	s.c.	20	Postsurgically	4	n.a.
Wen et al., 2019	Rats	Meloxicam	n.r.	2	Presurgically	1	n.a.

Information on studies reporting use of analgesic antipyretic agents [non-steroidal anti-inflammatory drugs (NSAIDs) and antipyretic analgesics] (5 studies in 2009; 16 studies in 2019) included in the subset of 200 studies. n.r., not reported, parameter was not reported; n.a., not applicable, extraction of this parameter was not feasible; i.p., intraperitoneal; i.m., intramuscular; s.c., subcutaneous; numbers in the study ID (e.g., Souza et al., 2019) indicate animal groups within individual studies.

For 2009, one study reported administering an NSAID during, and three studies after surgery, with flunixin being administered in two of 100 studies. The dose used in both studies was 1.1 mg/kg body weight (BW). One study administered the substance once subcutaneously, whereas the second study reported administering the substance three times with an application interval of 24 h but provided no information on the administration route. Other NSAIDs used were carprofen and ketoprofen. One study reported using the antipyretic analgesic acetaminophen/paracetamol. All substances were reported once, with no information on used doses for acetaminophen and ketoprofen. Carprofen was administered intraperitoneally at a dose of 5 mg/kg BW. All three analgesics were administered once.

In 2019, seven studies reported administering the NSAID before, one study during and eight studies after surgery. The most commonly used NSAID was meloxicam. Eight of 103 studies reported using it, with doses ranging from 0.03 to 5 mg/kg BW. Two studies administered the substance twice with an interval of 24 h and one study administered it four times with an interval of 24 h. The remaining studies reported administering meloxicam once. Of these eight studies, three did not report how the NSAID was administered. Four studies reported a subcutaneous administration. Here, doses ranged from 1 to 5 mg/kg BW. One study reported intraperitoneal administration at a dose of 0.03 to 0.05 mg/kg BW. The second most commonly used NSAID was ketoprofen, being used in five of 103 studies, with two studies both reporting application four times with an interval of 24 h. The remaining studies reported administering ketoprofen once. Four studies reported a subcutaneous administration of doses ranging from 3 to 5 mg/kg BW. The fifth study reported intraperitoneal administration of a dose of 5 mg/kg BW. Carprofen was used in three of 103 studies with doses ranging from 2 to 20 mg/kg BW, being administered seven times with an interval of 12 h in one study, and four times with an interval of 24 h in another study. The third study did not provide information on how often the substance was administered. Of these three studies, one reported subcutaneous administration of 20 mg/kg BW, another study reported intraperitoneal administration of 2 to 4 mg/kg BW while the third study did not provide information on how the substance was administered.

For complete information on all drugs used, please refer to Table 7 and Supplementary Tables 1, 2.

##### 3.3.4.3. Opioids

Two of the 100 studies from 2009, and 14 of the 103 studies from 2019, reported opioid administration. In one reference from 2019, animals were allocated to two groups receiving either an NSAID or an opioid.

The opioid administered in both studies from 2009 and almost all studies from 2019 (13/14 studies) was buprenorphine (2009: doses ranged from 0.02 to 1 mg/kg BW applied subcutaneously; 2019: doses ranged from 0.01 to 2 mg/kg BW applied subcutaneously and 0.01 to 0.1 mg/kg BW applied intraperitoneally). In three of 14 studies from 2019, doses were reported without information about the route of administration. One additional study reported neither dose nor route of administration. Buprenorphine was administered once in all studies from 2009 and the majority of studies from 2019 (10/13 studies). Two studies from 2019 described repeated administration with either seven administrations every 12 h, or ten administrations every 8 h. One study reported a postoperative administration every 6–12 h but provided no information on how often the opioid was administered. Use of butorphanol was reported in one study in 2019, with an intraperitoneal administration of a dose of 2.5 mg/kg BW.

For complete information on all drugs used, please refer to Table 8 and Supplementary Tables 1, 2.

**TABLE 8:** Studies reporting use of opioids from 2009 (*k* = 100) and 2019 (*k* = 100) included in the subset of 200 studies.

References	Species used	Drug	Administration route	Dosage (mg/kg)	Timepoint of first administration	Administered how many times in total	Administration interval (h post first administration)
Holtmaat et al., 2009	Mice	Buprenorphine	s.c.	1	Postsurgically	n.r.	12
Jafri et al., 2009	Rats	Buprenorphine	s.c.	0.02 to 0.05	Presurgically	1	n.a.
Aldehri et al., 2019	Rats	Buprenorphine	s.c.	0.1	Presurgically	1	n.a.
Bertoglio et al., 2019	Rats	Buprenorphine	s.c.	0.01	Postsurgically	1	n.a.
Bukhtiyarova et al., 2019	Mice	Buprenorphine	s.c.	0.1	Presurgically	1	n.a.
Duveau et al., 2019	Rats	Buprenorphine	i.p.	0.01	Postsurgically	1	n.a.
Jackson et al., 2019	Rats	Buprenorphine	s.c.	0.05	Postsurgically	1	n.a.
Jakkamsetti et al., 2019	Mice	Buprenorphine	n.r.	0.05	Postsurgically	1	n.a.
Jermakowicz et al., 2019	Rats	Buprenorphine	s.c.	0.2	Postsurgically	7	12
Kaefer et al., 2019	Rats	Buprenorphine	n.r.	0.1	Presurgically	1	n.a.
Mazza et al., 2019	Mice	Buprenorphine	n.r.	0.05	Presurgically	1	n.a.
Okada et al., 2019	Rats	Butorphanol	i.p.	2.5	Presurgically	1	n.a.
Szonyi et al., 2019	Mice	Buprenorphine	i.p.	0.1	Postsurgically	1	n.a.
Wang et al., 2019f	Mice	Buprenorphine	s.c.	0.1	Postsurgically	10	8
Wen L. et al., 2019	Mice	Buprenorphine	s.c.	2	Intrasurgically	n.r.	6 to 12
Zhao et al., 2019	Rats	Buprenorphine	n.r.	n.r.	Presurgically	1	n.a.

Information on studies reporting use of opioids from 2009 (k = 100) and 2019 (k = 100) included in the subset of 200 studies. n.r., not reported, parameter was not reported; n.a., not applicable, extraction of this parameter was not feasible; i.p., intraperitoneal; i.m., intramuscular; s.c., subcutaneous; numbers in the study ID (e.g., Jakkamsetti et al., 2019) indicate animal groups within individual studies.

#### 3.3.5. Anesthesia – detailed analysis of random subset of 200 studies

In this section, we refer to 103 studies from 2009 and 101 studies from 2019, as several references reported forming groups with animals receiving different approaches to induce general anesthesia.

In 2009, the most frequently applied general anesthesia approach was a combination of ketamine and xylazine (25/103 studies), with the following dose ranges: 25 to 120 mg/kg BW for ketamine and 2.5 to 110 mg/kg BW for xylazine. Two studies did not report the doses used.

The second most common substance to induce general anesthesia in 2009 was isoflurane (19/103 studies). Six studies provided information about the concentrations used, which ranged from 1 to 3.5 vol.%. Another six studies reported administering different concentrations for induction (range: 2.5–5 vol.%) and maintenance (range: 1–3 vol.%) of anesthesia. Seven studies did not provide information on concentrations. Other drugs used to induce anesthesia were: pentobarbital (15/103 studies), halothane (5/103 studies), and urethane (5/103 studies).

Full information on all substances used to induce general anesthesia can be found in Table 9 and Supplementary Tables 1, 2.

**TABLE 9:** Type of anesthesia reported in studies from 2009 (*k* = 100) and 2019 (*k* = 100) included in the subset of 200 studies.

References	Species used	Type of anesthesia	Administration route	Concentration (Vol. %)	Dosage (mg/kg)	Administered how many times in total
Bartolomucci et al., 2009	Mice	Ketamine/xylazine	i.p. injection	n.a.	Ketamine 100 xylazine 5	1
Behrend et al., 2009	Rats	Ketamine/xylazine	n.r.	n.a.	Ketamine 90 xylazine 10	1
Biella et al., 2009	Rats	Isoflurane/pentobarbital	Isoflurane inhalation pentobarbital injection	Isoflurane 2,5	Pentobarbital 2,5	n.a.
Boni et al., 2009	Rats	Pentobarbital	i.p. injection	n.a.	50	Once i.p. then continuously i.v.
Bramlett et al., 2009	Rats	Halothane	Inhalation	0,5 to 1	n.a.	n.a.
Byun et al., 2009	Mice	Ether	Inhalation	n.r.	n.a.	n.a.
Caltana et al., 2009	Rats	Sevoflurane	Inhalation	8	n.a.	n.a.
Carcak et al., 2009	Rats	Ketamine/xylazine	i.p. injection	n.a.	Ketamine 100 xylazine 10	1
Cemil et al., 2009	Rats	n.r.	n.a.	n.a.	n.a.	n.a.
Chen et al., 2009	Rats	Chloral hydrate	i.p. injection	n.a.	300	1
Cifani et al., 2009	Rats	Tiletamine/zolazepam	i.m. injection	n.a.	Tiletamine 200 zolazepam 200	1
Cunningham et al., 2009	Mice	n.r.	n.a.	n.a.	n.a.	n.a.
Datta et al., 2009	Rats	Pentobarbital	i.p. injection	n.a.	40	1
Diesch et al., 2009	Rats	Halothane	Inhalation	2	n.a.	n.a.
Diguet et al., 2009	Rats	Ketamine/xylazine	i.p. injection	n.a.	Ketamine 75 xylazine 10	1
Ding et al., 2009	Rats	Isoflurane	Inhalation	2	n.a.	n.a.
Doan et al., 2009	Rats	Isoflurane	Inhalation	1	n.a.	n.a.
Doretto et al., 2009	Rats	Tribromoethanol	i.p. injection	n.a.	n.r.	1
Dux et al., 2009	Rats	Thiopental	i.p. injection	n.a.	150	n.r.
Echegoyen et al., 2009	Rats	n.r.	n.a.	n.a.	n.a.	n.a.
Ehrlichman et al., 2009	Mice	Isoflurane	Inhalation	n.r.	n.a.	n.a.
Etholm and Heggelund, 2009	Mice	n.r.	n.a.	n.a.	n.a.	n.a.
Farias et al., 2009	Rats	Isoflurane	Inhalation	3 to 3,5	n.a.	n.a.
Foti et al., 2009	Rats	Pentobarbital	i.p. injection	n.a.	50	1
Francois et al., 2009	Rats	Isoflurane	Inhalation	2 to 5	n.a.	n.a.
Francois et al., 2009	Rats	Ketamine/xylazine	i.p. injection	n.a.	Ketamine 37 xylazine 5,5	1
Fritsch et al., 2009	Rats	Ketamine/medetomidine	i.p. injection	n.a.	Ketamine 60 medetomidine 0,5	1
Good et al., 2009	Rats	Ketamine/xylazine/ acepromazine	i.m. injection	n.a.	Ketamine 50 xylazine 10 acepromazine 1	20% booster of cocktail every 45 min or as needed
Griesbach et al., 2009	Rats	Isoflurane	Inhalation	2 to 4	n.a.	n.a.
Guidine et al., 2009	Rats	Halothane	Inhalation	2 to 4	n.a.	n.a.
Gurevicius et al., 2009	Mice	Pentobarbital/chloral hydrate	i.p. injection	n.a.	Pentobarbital 50 chloral hydrate 50	1
Hart et al., 2009	Rats	Ketamine/xylazine	i.p. injection	n.a.	Ketamine 100 xylazine 20	1
Harvey et al., 2009	Mice	n.r.	n.a.	n.a.	n.a.	n.a.
Hernandez-Gonzalez et al., 2009	Rats	Pentobarbital	i.p. injection	n.a.	35	1
Ho et al., 2009	Mice	Tribromoethanol	i.p. injection	n.a.	n.r.	1
Holtmaat et al., 2009	Mice	Ketamine/xylazine	i.p. injection	n.a.	Ketamine 100 xylazine 10	1
Hrncic et al., 2009	Rats	Pentobarbital	i.p. injection	n.a.	50	1
Huguet et al., 2009	Rats	Ketamine/xylazine	i.p. injection	n.a.	Ketamine 110 xylazine n.r.	1
Ishida et al., 2009	Rats	Pentobarbital	i.p. injection	n.a.	35	1
Ito et al., 2009	Mice	Pentobarbital	i.p. injection	n.a.	35	1
Itoh et al., 2009b	Rats	Pentobarbital	i.p. injection	n.a.	50	1
Itoh et al., 2009a	Rats	Pentobarbital	i.p. injection	n.a.	50	1
Jafri et al., 2009	Rats	Ketamine/xylazine	i.p. injection	n.a.	Ketamine 40 to 80 xylazine 5 to 10	1
Kalauzi et al., 2009	Rats	Ketamine/xylazine	i.p. injection	n.a.	Ketamine 80 xylazine 5	1
Katz et al., 2009	Rats	Isoflurane	Inhalation	2	n.a.	n.a.
Kim and Ong, 2009	Rats	Ketamine/xylazine	n.r.	n.a.	Ketamine n.r. xylazine n.r.	1
Lackovic et al., 2009	Rats	Chloral hydrate	i.p. injection	n.a.	300	1
Lee et al., 2009	Mice	Ketamine/xylazine	n.r.	n.a.	Ketamine 120 xylazine 6	1
Lee and Agoston, 2009	Rats	Isoflurane	Inhalation	n.r.	n.a.	n.a.
Li et al., 2009	Rats	Diethyl ether/pentobarbital	Diethyl ether inhalation pentobarbital injection	Diethyl ether n.r.	Pentobarbital 30	n.a.
Liu et al., 2009	Rats	Ketamine/xylazine	i.p. injection	n.a.	Ketamine 75 xylazine 10	1
Lopez-Martin et al., 2009	Rats	Pentobarbital	i.p. injection	n.a.	30	1
Lu et al., 2009	Rats	Isoflurane	Inhalation	n.r.	n.a.	n.a.
Lundblad et al., 2009	Mice	Pentobarbital/ketamine	i.p. injection	n.a.	Pentobarbital 50 ketamine 50	1
Magloire and Cattarelli, 2009	Rats	Pentobarbital/chloral hydrate	i.p. injection	n.a.	Pentobarbital n.r. chloral hydrate n.r.	1
Mark et al., 2009	Rats	Ketamine/xylazine	i.p. injection	n.a.	Ketamine 91 xylazine 9,1	1
McCracken and Grace, 2009	Rats	Urethane	i.p. injection	n.a.	1,5	1
Meeren et al., 2009	Rats	Isoflurane	Inhalation	n.r.	n.a.	n.a.
Merkler et al., 2009	Mice	Urethane/chloralose	n.r.	n.a.	Urethane 1000 chloralose 400	1
Merkler et al., 2009	Rats	Isoflurane	Inhalation	n.r.	n.a.	n.a.
Mian et al., 2009	Rats	n.r.	n.a.	n.a.	n.a.	n.a.
Mohammadi et al., 2009	Rats	Urethane	i.p. injection	n.a.	1500	1
Mollazadeh et al., 2009	Rats	n.r.	n.a.	n.a.	n.a.	n.a.
Mukherjee and Simasko, 2009	Rats	Ketamine/xylazine	n.r.	n.a.	Ketamine 87 xylazine 13	1
Nehlig et al., 2009	Rats	n.r.	n.a.	n.a.	n.a.	n.a.
Nuki et al., 2009	Mice	Ketamine/xylazine	i.p. injection	n.a.	Ketamine 100 xylazine 10	1
Onyszchuk et al., 2009	Mice	Isoflurane	Inhalation	1 to 2,5	n.a.	n.a.
Oshima et al., 2009	Mice	Pentobarbital	i.p. injection	n.a.	60	1
Potts et al., 2009	Mice	Tribromoethanol	n.r.	n.a.	n.r.	1
Qing et al., 2009	Rats	Chloral hydrate	i.p. injection	n.a.	300 to 350	1
Rahim et al., 2009	Rats	Isoflurane	Inhalation	n.r.	n.a.	n.a.
Rimoli et al., 2009	Rats	Chloral hydrate	i.p. injection	n.a.	400	1
Roiko et al., 2009	Rats	Ketamine/xylazine	i.m. injection	n.a.	Ketamine 75 xylazine 7,5	1
Rudnick et al., 2009	Mice	Isoflurane	Inhalation	n.r.	n.a.	n.a.
Sahin et al., 2009	Rats	Ketamine/ chlorpromazine	i.p. injection	n.a.	Ketamine 100 chlorpromazine 1	1
Samnick et al., 2009	Rats	Ketamine/xylazine	i.p. injection	n.a.	Ketamine 70 xylazine 20	1
Sasaki et al., 2009	Rats	Halothane	Inhalation	1 to 5	n.a.	n.a.
Schei et al., 2009	Rats	Isoflurane	Inhalation	2,3 to 5	n.a.	n.a.
Schmid et al., 2009	Rats	Urethane	n.r.	n.a.	1,5	1
Sekiya et al., 2009	Rats	Ketamine/xylazine	i.p. injection	n.a.	Ketamine 100 xylazine 9	1
Sher et al., 2009	Mice	Ketamine	n.r.	n.a.	n.r.	n.r.
Shultz et al., 2009	Rats	Isoflurane	Inhalation	2 to 4	n.a.	n.a.
Silvani et al., 2009	Mice	Isoflurane	Inhalation	1 to 2	n.a.	n.a.
Sinton et al., 2009	Mice	Ketamine/xylazine	i.p. injection	n.a.	Ketamine 25 xylazine 2,5	1
Song and Poon, 2009	Rats	Urethane	i.p. injection	n.a.	1500	1
Takahashi et al., 2009	Rats	Hypothermia or isoflurane	Inhalation	n.r.	n.a.	n.a.
Takahashi et al., 2009	Rats	Ketamine/xylazine	n.r.	n.a.	Ketamine 60 xylazine 100	1
Tanida et al., 2009	Rats	Pentobarbital	i.p. injection	n.a.	35	1
Tchekalarova et al., 2009	Rats	Ketamine/xylazine	i.p. injection	n.a.	Ketamine 80 xylazine 20	1
Thomas et al., 2009	Rats	Halothane	Inhalation	n.r.	n.a.	n.a.
Topchiy et al., 2009	Rats	Ketamine/xylazine/ isoflurane	Ketamine + xylazine i.m. injection isoflurane inhalation	n.r.	Ketamine 100 xylazine 10	n.r.
Touzani et al., 2009	Rats	Ketamine/xylazine	i.p. injection	n.a.	Ketamine 63 xylazine 9,4	1
Tsanov and Manahan-Vaughan, 2009	Rats	Pentobarbital	i.p. injection	n.a.	40	1
Wagner et al., 2009	Rats	Isoflurane	Inhalation	1 to 4	n.a.	n.a.
Wan et al., 2009	Rats	Pentobarbital	i.p. injection	n.a.	45	n.r.
Wigren et al., 2009	Rats	Diazepam/medetomidine /ketamine	i.p. injection	n.a.	Diazepam 2,5 medetomidine 0,4 ketamine 60	1
Worthen et al., 2009	Rats	n.r.	n.a.	n.a.	n.a.	n.a.
Xue et al., 2009	Mice	Ketamine/xylazine	n.r.	n.a.	Ketamine n.r. xylazine n.r.	n.r.
Yoon et al., 2009	Rats	Pentobarbital	i.p. injection	n.a.	45	1
Young et al., 2009	Rats	Ketamine/xylazine	i.m. injection	n.a.	Ketamine 85 xylazine 15	1
Yu et al., 2009	Rats	Urethane	i.p. injection	n.a.	1750	1
Yurek et al., 2009	Rats	n.r.	n.a.	n.a.	n.a.	n.a.
Zeng et al., 2009	Rats	Isoflurane	Inhalation	1 to 2	n.a.	n.a.
Aldehri et al., 2019	Rats	Isoflurane	Inhalation	n.r.	n.a.	n.a.
Asan and Sahin, 2019	Rats	Isoflurane/ketamine/ xylazine	Isoflurane inhalation ketamine + xylazine i.p. injection	Isoflurane 1 to 5	Ketamine 80 xylazine 12	n.a.
Baud et al., 2019	Rats	n.r.	n.r.	n.r.	n.r.	n.r.
Bazzu et al., 2019	Mice	Isoflurane	Inhalation	1 to 3	n.a.	n.a.
Bertoglio et al., 2019	Rats	Isoflurane	Inhalation	2 to 5	n.a.	n.a.
Bleimeister et al., 2019	Rats	Isoflurane	Inhalation	2 to 4	n.a.	n.a.
Bukhtiyarova et al., 2019	Mice	Isoflurane	Inhalation	1 to 2	n.a.	n.a.
Burgdorf et al., 2019	Rats	Isoflurane	Inhalation	2 to 5	n.a.	n.a.
Casanova-Carvajal et al., 2019	Mice	n.r.	n.a.	n.a.	n.a.	n.a.
Chen G. et al., 2019	Mice	Chloral hydrate	n.r.	n.a.	350	1
Chen and Bi, 2019	Mice	Ketamine/xylazine	i.p. injection	n.a.	Ketamine 100 xylazine 10	1
Chen G. et al., 2019	Mice	n.r.	n.a.	n.a.	n.a.	n.a.
Chen G. et al., 2019	Mice	Isoflurane	Inhalation	1.5	n.a.	n.a.
Chen T. et al., 2019	Rats	Pentobarbital	i.p. injection	n.a.	50	1
Chitturi et al., 2019	Rats	Ketamine/xylazine	i.p. injection	n.a.	Ketamine 80 xylazine 10	1
Christiaen et al., 2019	Rats	Isoflurane	Inhalation	2 to 5	n.a.	n.a.
Colangeli et al., 2019	Rats	Isoflurane	Inhalation	1 to 5	n.a.	n.a.
da Silva Pacheco et al., 2019	Rats	Ketamine/xylazine/ acepromazine	s.c. injection	n.a.	Ketamine n.r. xylazine n.r. acepromazine n.r.	1
Daglas et al., 2019	Mice	Tribromoethanol	i.p. injection	n.a.	0.5	1
Dal-Pont et al., 2019	Rats	Ketamine/xylazine	i.m. injection	n.a.	Ketamine 80 xylazine 10	1
Delaney et al., 2019	Mice	n.r.	n.a.	n.a.	n.a.	n.a.
Dreier et al., 2019	Rats	Thiopental	i.p. injection	n.a.	100	1
Du et al., 2019	Rats	Isoflurane	Inhalation	2	n.a.	n.a.
Duveau et al., 2019	Rats	Isoflurane	Inhalation	2 to 2,5	n.a.	n.a.
Etter et al., 2019	Mice	Isoflurane	Inhalation	0.5 to 5	n.a.	n.a.
Ewell et al., 2019	Rats	Isoflurane	Inhalation	2 to 2.5	n.a.	n.a.
Farakhor et al., 2019	Rats	Ketamine/xylazine	n.r.	n.a.	Ketamine 100 xylazine 10	1
Farooq and Dragoi, 2019	Rats	Isoflurane	Inhalation	1 to 2	n.a.	n.a.
Fiath et al., 2019	Rats	Ketamine/xylazine	i.m. injection	n.a.	Ketamine 75 xylazine 10	n.r.
Fortress et al., 2019	Rats	Ketamine/xylazine	i.p. injection	n.a.	Ketamine 60 xylazine 7	1
Hu et al., 2019	Mice	Chloral hydrate	n.r.	n.a.	n.r.	1
Ilieva et al., 2019	Rats	Ketamine/xylazine	i.p. injection	n.a.	Ketamine 80 xylazine 20	1
Jackson et al., 2019	Rats	Isoflurane	Inhalation	2 to 5	n.a.	n.a.
Jakkamsetti et al., 2019	Mice	Ketamine/xylazine/ acepromazine	i.p. injection	n.a.	Ketamine 100 xylazine 10 acepromazine 2	n.r.
Jakkamsetti et al., 2019	Mice	Isoflurane	Inhalation	1 to 2	n.a.	n.a.
Jermakowicz et al., 2019	Rats	Isoflurane	Inhalation	1.2	n.a.	n.a.
Kaefer et al., 2019	Rats	Isoflurane	Inhalation	0.5 to 3	n.a.	n.a.
Katagiri et al., 2019	Rats	Pentobarbital	i.p. injection	n.a.	50	1
Kenny et al., 2019	Mice	Isoflurane	Inhalation	1.5 to 3	n.a.	n.a.
Kim and Narayanan, 2019	Mice	n.r.	n.a.	n.a.	n.a.	n.a.
Kunori and Takashima, 2019	Rats	Isoflurane	Inhalation	1.25 to 3	n.a.	n.a.
Kyyriainen et al., 2019	Mice	Pentobarbital	i.p. injection	n.a.	60	1
Lee et al., 2019	Rats	Ketamine/xylazine	i.p. injection	n.a.	Ketamine 7.5 to 8.75 xylazine 3 to 3.5	n.r.
Levata et al., 2019	Mice	Ketamine/xylazine	i.p. injection	n.a.	Ketamine 80 xylazine 12	1
Li F. et al., 2019	Rats	Isoflurane	Inhalation	n.r.	n.a.	n.a.
Li Y. T. et al., 2019	Rats	Urethane	n.r.	n.a.	1200	1
Li Q. et al., 2019	Rats	Urethane	i.p. injection	n.a.	n.r.	1
Luo et al., 2019	Mice	n.r.	n.a.	n.a.	n.a.	n.a.
Lv et al., 2019	Rats	Isoflurane	Inhalation	2	n.a.	n.a.
Ma et al., 2019	Mice	Isoflurane	Inhalation	2 to 3	n.a.	n.a.
Mastrella et al., 2019	Mice	Ketamine/xylazine	n.r.	n.a.	Ketamine 100 xylazine 10	1
Mazza et al., 2019	Mice	Isoflurane	Inhalation	n.r.	n.a.	n.a.
Mittal et al., 2019	Mice	Isoflurane	Inhalation	n.r.	n.a.	n.a.
Mo et al., 2019	Mice	n.r.	n.a.	n.a.	n.a.	n.a.
Mohammad et al., 2019	Rats	Isoflurane	Inhalation	2 to 5	n.a.	n.a.
Mohammadipoor-Ghasemabad et al., 2019	Rats	Ketamine/xylazine	i.p. injection	n.a.	Ketamine 80 xylazine 10	1
Mohammadpoory et al., 2019	Rats	Ketamine/xylazine	i.p. injection	n.a.	Ketamine 60 xylazine 10	1
Moller et al., 2019	Rats	Chloral hydrate	i.p. injection	n.a.	360	1
Murai et al., 2019	Rats	Isoflurane	Inhalation	n.r.	n.a.	n.a.
Njoku et al., 2019	Rats	Isoflurane	Inhalation	2 to 4	n.a.	n.a.
O’Brien et al., 2019	Mice	Isoflurane	n.a.	n.r.	n.a.	n.a.
Ogun et al., 2019	Rats	Ketamine/xylazine	i.m. injection	n.a.	Ketamine 90 xylazine 10	1
Okada et al., 2019	Rats	Medetomidine/ midazolam	i.p. injection	n.a.	Medetomidine 0.375 midazolam 2	1
Park et al., 2019	Mice	Isoflurane	Inhalation	1.5 to 3	n.a.	n.a.
Pettibone et al., 2019	Rats	n.r.	n.a.	n.a.	n.a.	n.a.
Pfluger et al., 2019	Rats	Isoflurane	Inhalation	1.5 to 4	n.a.	n.a.
Qiao et al., 2019	Mice	n.r.	n.a.	n.a.	n.a.	n.a.
Romoli et al., 2019	Mice	Tiletamine/zolazepam/ xylazine	n.r.	n.a.	Tiletamine n.r. zolazepam n.r. xylazine n.r.	1
Russell et al., 2019	Rats	Isoflurane	Inhalation	2 to 4	n.a.	n.a.
Sa et al., 2019	Rats	Ketamine/xylazine	n.r.	n.a.	Ketamine n.r. xylazine n.r.	1
Sharma et al., 2019	Rats	Ketamine	i.p. injection	n.a.	75	1
Shaver et al., 2019	Rats	Isoflurane	Inhalation	2 to 5	n.a.	n.a.
Shiuchi et al., 2019	Mice	Ketamine/xylazine	i.p. injection	n.a.	Ketamine 100 xylazine 10	1
Simader et al., 2019	Rats	Ketamine/xylazine/ isoflurane	Ketamine + xylazine i.p. injection isoflurane inhalation	Isoflurane 1.5	Ketamine 100 xylazine 10	1
Slezia et al., 2019	Mice	Ketamine/xylazine	i.p. injection	n.a.	Ketamine 100 xylazine 10	n.r.
Souza et al., 2019	Rats	Ketamine/xylazine/ acepromazine	i.m. injection	n.a.	Ketamine 75 xylazine 5 acepromazine 1	n.r.
Souza et al., 2019	Rats	Ketamine/xylazine/ acepromazine	n.r.	n.a.	Ketamine n.r. xylazine n.r. acepromazine n.r.	n.r.
Stanchi et al., 2019	Mice	Ketamine/xylazine	i.p. injection	n.a.	Ketamine 100 xylazine 10	n.r.
Stanojlovic et al., 2019	Mice	Isoflurane	Inhalation	1 to 4	n.a.	n.a.
Sun et al., 2019	Mice	Isoflurane	Inhalation	1,5 to 4	n.a.	n.a.
Suzuki et al., 2019	Mice	Isoflurane	Inhalation	1 to 3	n.a.	n.a.
Szonyi et al., 2019	Mice	Ketamine/xylazine/ isoflurane	Ketamine + xylazine i.p. injection isoflurane inhalation	Isoflurane 2	Ketamine n.r. xylazine n.r.	n.r.
Szonyi et al., 2019	Mice	Ketamine/xylazine/ isoflurane	Ketamine + xylazine i.p. injection isoflurane inhalation	Isoflurane 2	Ketamine n.r. xylazine n.r.	n.r.
Tomov et al., 2019	Rats	Isoflurane	Inhalation	2 to 4	n.a.	n.a.
Villa-Cedillo et al., 2019	Mice	Tribromoethanol	n.r.	n.a.	125 to 150	1
Villasana et al., 2019	Mice	Isoflurane	Inhalation	2	n.a.	n.a.
Wang et al., 2019f	Mice	Isoflurane	Inhalation	3	n.a.	n.a.
Wang et al., 2019g	Mice	Pentobarbital	i.p. injection	n.a.	100	1
Wang et al., 2019a	Mice	Isoflurane	Inhalation	1.5	n.a.	n.a.
Wang et al., 2019h	Mice	n.r.	n.a.	n.a.	n.a.	n.a.
Wang et al., 2019b	Rats	n.r.	n.a.	n.a.	n.a.	n.a.
Wang et al., 2019c	Rats	Chloral hydrate	i.p. injection	n.a.	n.r.	1
Wang et al., 2019d	Rats	Isoflurane	Inhalation	2 to 5	n.a.	n.a.
Wang et al., 2019e	Rats	Chloral hydrate	i.p. injection	n.a.	1	1
Wen L. et al., 2019	Mice	Ketamine/xylazine	i.p. injection	n.a.	Ketamine 80 to 100 xylazine 10	1
Wen et al., 2019	Rats	Ketamine/xylazine/ acepromazine	n.r.	n.a.	Ketamine 90 xylazine 2.7 acepromazine 0.64	1
Xu et al., 2019	Mice	Pentobarbital	i.p. injection	n.a.	65	1
Yang et al., 2019	Rats	Isoflurane	Inhalation	2 to 5	n.a.	n.a.
Yeung et al., 2019	Mice	Ketamine/medetomidine	s.c. injection	n.a.	Ketamine 75 medetomidine 1	1
Zhang et al., 2019c	Mice	Pentobarbital	n.r.	n.a.	50	1
Zhang et al., 2019b	Rats	Ketamine/xylazine	i.p. injection	n.a.	Ketamine 100 xylazine 10	1
Zhang et al., 2019a	Rats	Isoflurane	Inhalation	2 to 5	n.a.	n.a.
Zhao et al., 2019	Rats	Pentobarbital	n.r.	n.a.	35	1

Information on type of anesthesia reported in studies from 2009 (k = 100) and 2019 (k = 100) included in the subset of 200 studies. Year of pub., year of publication; n.r., not reported, parameter was not reported; n.a., not applicable, extraction of this parameter was not feasible; i.p., intraperitoneal; i.m., intramuscular; numbers in the study ID (e.g., Francois et al., 2009) indicate animal groups within individual studies.

Isoflurane was the most frequently reported substance used for anesthesia in 2019 (44/101 studies). Twenty-four studies reported a different concentration for induction (range 2.5–5 vol.%) and maintenance (range 0.5–5 vol.%) of general anesthesia, whereas 15 studies did not specify separate concentrations for induction and maintenance of general anesthesia (concentrations ranging from 0.5 to 4 vol.%). Five studies did not provide information on concentration at all.

In 2019, the second most common approach was the combination of ketamine and xylazine with 19 of 101 studies reporting this combination. The doses ranged from 7.5 to 120 mg/kg BW for ketamine and 3–100 mg/kg BW for xylazine. Further anesthetic drugs comprised pentobarbital (7/101 studies) and chloral hydrate (5/101 studies).

Full information on all substances used to induce general anesthesia can be found in Table 9 and Supplementary Tables 1, 2.

#### 3.3.6. Risk of bias – detailed analysis of random subset of 200 studies

Blinding was reported in 16 of 100 studies in 2009 (blinding of data analysis: 13 studies; during experimental procedure: two studies; during experimental procedure and data analysis: one study) and 16 of 100 studies in 2019 (of data analysis: 13 studies; during experimental procedure and data analysis: three studies). Randomization was reported in 14 of 100 studies published in 2009 (randomized group allocation: twelve studies; randomized data analysis: two studies) and 38 of 100 studies published in 2019 (group allocation: 36 studies; conducting of experimental procedure: one study; randomized data analysis: one study). In 2009, two of 100 studies reported conducting a power analysis, but none reported doing so in 2019. Reporting of doses and routes of application from studies reporting use of analgesics or local anesthetics was incomplete (no dose and/or route of application reported) in seven of eleven studies from 2009 and 23 of 32 studies from 2019. Regarding used anesthetics, 32 of 103 studies from 2009 and 30 of 101 studies from 2019 provided insufficient information on used doses or routes of application. To conclude, the majority of our sample did not report adequate blinding and randomization to prevent bias affecting their outcomes. Underreporting of doses and routes of administration was also common.

## 4. Discussion

This systematic scoping review provides representative information about common anesthetic and analgesic regimens applied perioperatively for craniotomy in mice and rats in 2009 and 2019. The extracted data inform us about the development of analgesic approaches over a decade since the report by Stokes et al. (2009), which described the lack of adequate analgesic regimens in the vast majority of mouse and rat studies with surgical interventions. While our analyses showed an increase in the application of analgesic drugs and local anesthetics in mouse and rat studies with intracranial surgery in 2019 versus 2009, the issue of undertreatment of surgical pain seems to persist, at least in the context of craniotomies. Moreover, the proportion of studies reporting multimodal analgesic regimens remains low. Please note that our data does not allow a conclusion on whether an analgesic regimen was sufficient or insufficient in specific studies, as neither a low nor high number of analgesics alone aligns with sufficient or insufficient analgesia. Aspects like dosage, administration interval and frequency of administration also have to be considered for the interpretation regarding sufficiency of the used analgesic regimen. The majority of studies from both years did not report the administration of any analgesic or local anesthetic, which suggests a lack of efforts to control postsurgical pain in the majority of experimental mice and rats undergoing craniotomy. This finding confirms recent statements by laboratory animal science experts (Jirkof, 2017; Flecknell, 2018; Foley et al., 2019). The high number of studies without description of analgesic regimen is alarming. Adequate treatment and prevention of pain is an ethical and, with most national regulations, also a legal requirement for any intervention in experimental animals (European Union, 2010; Jirkof, 2017; Prescott and Lidster, 2017). It is well known from human patients that uncontrolled pain can, among others, contribute to distress, negatively impact the affective state, disturb sleep patterns, prolong post-surgical recovery and healing phases, and increase risk of post-surgical complications and morbidity (Basali et al., 2000; Ortiz-Cardona and Bendo, 2007; Flexman et al., 2010; Dahl and Kehlet, 2011; Hansen et al., 2013). Studies focusing on rodents demonstrated that pain can for instance affect activity, behavioral patterns, circadian rhythmicity, and sleep duration (Carstens and Moberg, 2000; Jirkof et al., 2010, 2012, 2013). In the context of surgical procedures, it is of particular relevance that the uncontrolled activation of the nociceptive system can result in its sensitization at different levels, thereby contributing to prolonged and more severe post-surgical pain states with hyperalgesia and allodynia, and a risk for chronic pain (Kaur et al., 2000; Joshi and Ogunnaike, 2005; Latremoliere and Woolf, 2009; Dahl and Kehlet, 2011; Thapa and Euasobhon, 2018). Thus, the high number of publications not reporting the use of analgesics raises particular concerns regarding animal welfare. While exceptions might be defendable under very exceptional circumstances, the available multitude of analgesic drugs and local anesthetics should allow for an optimal analgesic regimen for the vast majority of studies without confounding the readout parameters (Peterson et al., 2017; Flecknell, 2018). In addition, an adjusted study design with longer post-surgical recovery phases and comparisons with substance-control groups can help to ensure data quality and correct interpretation (Moser, 2020; Jirkof and Potschka, 2021).

It can obviously not be emphasized enough that uncontrolled or insufficiently controlled pain and excessive activation of the nociceptive system exerts a multitude of effects. For instance, it can result in alterations of neurotransmitters, hormones, enzymes, metabolites, an impact on immune responses, and on sympathetic activity with a pronounced influence on cardiovascular function (Carstens and Moberg, 2000; Page, 2003; Arras et al., 2007; Jirkof, 2017; Jirkof and Potschka, 2021). These examples indicate that insufficiently controlled pain can influence a multitude of scientific readout parameters, with a detrimental impact on precision, reproducibility and external validity of the data (Jirkof, 2017; Peterson et al., 2017; Jirkof and Potschka, 2021).

As already mentioned, undertreatment of craniotomy-associated pain has also been reported in humans, and related to concerns about adverse effects comprising hypercapnia, hypertension, nausea and vomiting, sedation, and reduced blood coagulation, which might compromise neurological assessment or contribute to complications in the post-surgical phase (Leslie and Williams, 2005; Vadivelu et al., 2016). It should be taken into account that uncontrolled pain will also result in effects which can increase the risks in the post-craniotomy phase (Basali et al., 2000). The undertreatment of human patients undergoing craniotomy results from the idea that intracranial procedures are less painful than other surgeries (Dunbar et al., 1999). However, recent studies have demonstrated otherwise. Many patients reported moderate to severe pain in the early post-surgical phase (De Benedittis et al., 1996; Gottschalk et al., 2007; Mordhorst et al., 2010; Dunn et al., 2016; Bello et al., 2022), and acute post-surgical headache may persist or recur chronically in a subgroup of patients (Kaur et al., 2000; Vadivelu et al., 2016; Lutman et al., 2018). Thus, classifying the pain potentially associated with intracerebral electrode implantation in laboratory rodents as minimal to mild, as previously suggested in 2007 by ACLAM Task Force members (Kohn et al., 2007), is questionable. However, the expert group did already emphasize that the suggested categories should be considered as movable sliding scales. While previous findings from our group following implantation of electrodes in sham control groups argued against any long-term pain states and chronic issues, assessment in the early post-surgical phase confirmed the need for an efficacious analgesic regimen following craniotomy in mice and rats (Moller et al., 2018; Koska et al., 2019; Seiffert et al., 2019; Boldt et al., 2021; Buchecker et al., 2022). The need for analgesia has been further supported by Cho et al. (2019), who assessed the efficacy of meloxicam, carprofen and buprenorphine in comparison with a control group without analgesia in mice following craniotomy using the mouse grimace scale (MGS). In that study, all analgesics and routes of administration led to significant reductions in MGS scores compared to the control group.

The use of analgesic drugs increased from 2009 to 2019. There was no evidence for a preference of non-opioid analgesics versus opioid analgesics. The group of non-opioid analgesics comprised various drugs including antipyretic analgesics without a relevant anti-inflammatory effect, such as metamizole/dipyrone or acetaminophen/paracetamol, as well as NSAIDs such as carprofen, meloxicam and ketoprofen. While the choice of drugs with an anti-inflammatory effect can be advantageous in the postsurgical phase, as the limitation of inflammatory signaling will limit the activation of nociceptors, the specific risks associated with craniotomy procedures need to be taken into account. In this context, it is of interest that the additional use of paracetamol is also recommended to control post-craniotomy headache in human patients by different experts (Kotak et al., 2009; Dunn et al., 2016; Lutman et al., 2018). While NSAIDs are routinely used by different neurosurgeons, the avoidance of NSAIDs and preference of antipyretic analgesics might offer advantages concerning the risk for intracranial hemorrhage (Palmer et al., 1994; Vadivelu et al., 2016; Lutman et al., 2018). Thus, the use of antipyretic analgesics might also be justified in the context of craniotomies in mice and rats despite the lack of anti-inflammatory effects. On the other hand, the efficacy of the different drugs for different types of pain should be considered. For instance, it should be questioned whether metamizole/dipyrone achieves opioid-like efficacy in animals undergoing craniotomy, as the spasmolytic effect could contribute to a favorable efficacy profile in laparotomy procedures but may not reach an analgesic effect in craniotomy-associated pain as, for example, an opioid would.

The selection of NSAIDs used in studies from 2009 and 2019 was limited to traditional NSAIDs, without reported use of cyclooxygenase-2 inhibitors, i.e., coxibs. While these increase the risks for cardio- and cerebrovascular events in human patients (Kearney et al., 2006; Arfè et al., 2016; Fanelli et al., 2017; Gunter et al., 2017), coxibs are frequently used in veterinary medicine, and they might offer advantages for craniotomy procedures in experimental animals as they are not associated with enhanced bleeding risk (Lutman et al., 2018). Thus, it would be of particular future interest to conduct studies directly comparing traditional NSAIDs with coxibs.

The most common opioid used in mice and rats with craniotomy was buprenorphine. Other opioids administered were butorphanol, tramadol, fentanyl and piritramide. In comparison with buprenorphine, butorphanol and tramadol both have a lower efficacy. While adverse effects of opioids have limited their use in human patients with craniotomy (Leslie and Williams, 2005; Ortiz-Cardona and Bendo, 2007; Dunn et al., 2016; Vadivelu et al., 2016; Ban et al., 2019), it is nevertheless surprising to see that only one of the studies reported the use of intrasurgical administration of fentanyl, and only 14 out of 2234 studies across both years reported its presurgical administration. Likewise, only a handful of studies reported the perioperative administration of tramadol, piritramide and other full μ receptor agonists.

In laboratory rodents, one major issue with opioid use is nausea, pica behavior, loss of appetite, and weight loss (Tallett et al., 2009; Sarabia-Estrada et al., 2017). In this respect, slow-release preparations might be superior to regular formulations (Foley, 2014), which are unfortunately not yet licensed at a global level.

Intraoperative skin infiltration and scalp blockade by injecting local anesthetics into the skin surrounding the area of incision or near nerves innervating the operating field are considered as highly efficacious measures in human patients with craniotomy; their efficacy has been confirmed in several clinical studies and a meta-analysis (Guilfoyle et al., 2013; Vallapu et al., 2018). Thus, it is promising that the number of studies reporting the application of local anesthesia for intracranial surgery in mice and rats was higher in 2019 than in 2009. On the other hand, the overall rate of local anesthesia use still seems to be very low. This is unfortunate, as local anesthetics can efficaciously block transmission of nociceptive signals and can therefore prevent sensitization of the nociceptive system (Haldar et al., 2015; Vadivelu et al., 2016).

In human patients, a relatively new approach to perioperative analgesia is the administration of gabapentinoids (e.g., gabapentin, pregabalin) in addition to other opioid or non-opioid analgesics. These gabapentinoids are antiseizure medications which also exert antihyperalgesic and antinociceptive effects, and thereby decrease opioid requirements (Vadivelu et al., 2016; Tsaousi et al., 2017). In our data set, the administration of gabapentinoids for perioperative pain-management was not reported. This indicates that the use of these drugs in rodent models of craniotomy is not yet common practice.

The timing of the administration of analgesics and local anesthetics is of particular relevance for efficacy. It is strongly recommended to guarantee a continuous limitation of the activation of the nociceptive system throughout the surgical and post-surgical phase, to avoid any breakthrough pain and reduce sensitization processes contributing to hyperalgesia and allodynia (Joshi and Ogunnaike, 2005; Latremoliere and Woolf, 2009). Intrasurgical administration of opioids and non-opioid analgesics was only reported by very few studies (*k* = 4). It is emphasized that their administration during surgery can imply the risk that the nociceptive system has already been activated prior to analgesic drug exposure if no analgesic management has been applied before surgery. A preventive application of analgesics or local anesthetics can inhibit the activation of the nociceptive system, as an analgesic treatment is started before onset of the first painful stimulus, which leads to an inhibited nociceptive transmission and thereby contributes to a reduced central sensitization (Dahl and Kehlet, 2011; Vadivelu et al., 2014).

Multimodal analgesia regimens are strongly recommended in human and veterinary medicine for several reasons. In line with this, scientists have also been encouraged to implement the multimodal analgesia regimen in experimental studies (White and Kehlet, 2010; Barazanchi et al., 2018; Flecknell, 2018; Foley et al., 2019). The combination of different analgesics from different drug classes allows interference with nociceptive signaling at different levels of the nociceptive system (Kehlet and Dahl, 1993; Corletto, 2007). This renders multimodal approaches more efficacious based on additive or synergistic effects (Kehlet and Dahl, 1993; Schug, 2006; Girard et al., 2016). In addition, the doses of the different components of a multimodal regimen can often be reduced, thereby resulting in an advantageous tolerability profile of multimodal pain management approaches (White, 2008; Gritsenko et al., 2014; Rafiq et al., 2014; Foley et al., 2019). The advantage of the minimization of opioid doses by multimodal regimens has also been specifically discussed for craniotomy procedures in humans (Ban et al., 2019). Thus, it seems unfortunate that this has not yet been widely implemented for intracranial surgery in mice and rats. However, it is encouraging to see that there is an increase from 2009 to 2019 in using more than one analgesic drug.

Careful monitoring of vital functions during surgery, perioperative care comprising maintenance of body temperature and application of eye ointment, as well as non-pharmacological measures to control pain, can improve surgical outcomes and recovery, and can limit distress and pain (Schuler et al., 2009). However, the detailed assessment of a random sample of studies from 2009 and 2019 confirmed a low reporting rate for these adjunctive measures.

The design of an optimal analgesic regimen needs to go hand in hand with the selection of the anesthetic drugs. The comparison between data from 2009 and 2019 revealed an increase in the use of inhalational anesthesia, with isoflurane being the most commonly used anesthetic. Inhalational anesthesia offers several advantages compared to systemic anesthesia, including excellent controllability associated with good tolerability and low anesthesia risks (Hildebrandt et al., 2008; Lerman and Jöhr, 2009; Jedlicka et al., 2021). Unfortunately, the commonly used vaporous inhalation anesthetics do not exert relevant analgesic effects themselves. While this is not of immediate relevance during surgery, when the animals have lost consciousness and are in a tolerance state, the lack of antinociceptive impact raises the chance of sensitization, which can result in increased severity of post-surgical pain (Latremoliere and Woolf, 2009). While the same considerations apply for pentobarbital, which has still been used in some studies in 2009 and 2019, any combination comprising ketamine and an alpha2-sympathomimetic (such as xylazine or medetomidine) might benefit from the analgesic effects of the two drugs. On the other hand, these systemic combinations have clear disadvantages regarding controllability and anesthesia risks in comparison with inhalation anesthesia.

The selection of an appropriate anesthesia and analgesia regimen also needs to take the type of surgery into account. A different level of invasiveness, as well as the specific characteristics of the intervention, might result in different levels and types of post-surgical pain. For example, surgical procedures in the context of traumatic brain injury and ischemic or hemorrhagic stroke may put the animals at risk for higher levels of pain related to increases in intracranial pressure. Our data sets did not show a clear relationship between pain management approaches and interventions. However, a conclusive focused analysis would require a larger sample of studies.

Our work included an assessment of a small subset of parameters associated with study quality and the risk of bias. In line with various previous studies (Haahr and Hróbjartsson, 2006; Moja et al., 2014; Barcot et al., 2019), in our sample the risk of bias seemed to be poorly controlled, with basic principles such as blinding and randomization only reported for a small number of studies. While this is not directly relevant to our description of anesthetic and analgesic procedures, it raises concerns because there is evidence that the lack of randomization and blinding can result in inflated effect sizes (McCann et al., 2014; Saltaji et al., 2018).

As we extracted data from literature, our results are limited by the incomplete reporting by the original authors; their descriptions of the experimental methods could lack details. Thus, additional anesthetic and analgesic drugs may have been used compared to our findings. In spite of the introduction of the ARRIVE guidelines (Animal Research: Reporting of *In Vivo* Experiments), reporting of experimental details for primary studies is often far from complete. With our results, we cannot distinguish underreporting from undertreatment. Moreover, the preference for male animals is still evident in our sample of studies from 2019. Thus, the majority of the here included studies seem to ignore the National Institutes of Health recommendations from 2014 to perform studies in both sexes, as long as both are relevant for the research question (Clayton and Collins, 2014). While there is a lag between performing and publishing studies, and while recommendations cannot be applied retrospectively, we would still have expected to see some improvement in 2019. Lastly, it must be noted that we performed a scoping review; not a full systematic review. In line with the scoping approach, we only searched a single database; PubMed, and did not aim to include all relevant literature. It is likely that studies not indexed in PubMed were not retrieved by our search, but as PubMed is one of the largest medical databases, we do not expect our sample to be biased.

In conclusion, analysis of studies with craniotomy in mice and rats published in 2009 and 2019 revealed a slight increase in the reporting of perioperative administration of analgesics and local anesthetics. However, the high number of studies without any description of efforts to control pain suggests that inadequate analgesia is a persistent issue in the context of intracranial surgery in laboratory rodents. The persistently rare reporting of multimodal approaches, local anesthetic procedures, and adjunctive care measures, underscores the need for an intensified training and education of those working with animals subjected to craniotomies in the adequate application and reporting of anesthesia and analgesia.

## Data availability statement

The original contributions presented in this study are included in the article/Supplementary material, further inquiries can be directed to the corresponding author.

## Author contributions

HP, MB, and AB acquired the funding. HP supervised the review. HP, CL, MB, and AB conceptualized the review. MR wrote the review protocol and the search string under the supervision from HP and CL. HK, KA, MR, NM, AG, MB, HS, KS, LS, and PJ performed the screening. AG and LS performed the quality checks. HK performed the statistical analyses. HK, CL, and HP wrote the manuscript. All authors read and approved the manuscript.

## Footnotes

1
https://osf.io/7d4qe

2
https://randomizer.org/
