# Supplementary material for: Anesthesia and analgesia for experimental craniotomy in mice and rats: a systematic scoping review comparing the years 2009 and 2019
Source: Front Neurosci. 2023 May 3;17:1143109. doi: 10.3389/fnins.2023.1143109 (PMC10188949; doi:10.3389/fnins.2023.1143109)
Supplement: Supplementary file 1 [file Data_Sheet_1.DOCX]

**Supplementary file**

**Contents:**

- Supplementary Methods 1: Study protocol
- Supplementary Methods 2: PRISMA (Preferred Reporting Items for Systematic Reviews and Meta-Analyses) Checklist Scoping Reviews
- Supplementary Methods 3: Search strategy for Pubmed
- Supplementary table S1: List of subset of 200 studies, included studies from 2009 and evaluated parameters
- Supplementary table S2: List of subset of 200 studies, included studies from 2019 and evaluated parameters
- Supplementary table S3: List of all included studies and evaluated parameters
- Supplementary Methods 4: Results statistical analysis

**Supplementary Methods 1: Study protocol**

| 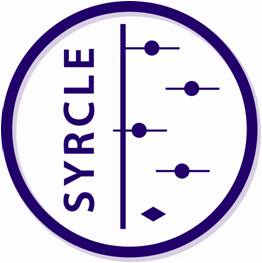 **Systematic Review Protocol for Animal Intervention Studies**  **Format by SYRCLE (**[**www.syrcle.nl**](http://www.syrcle.nl)**)**  **Version 2.0 (December 2014)** | | | |
| --- | --- | --- | --- |
| **Item #** | **Section/Subsection/Item** | **Description** | **Check for approval** |
|  | A. General | | |
| 1. | Title of the review | Anesthesia and analgesia for experimental craniotomy in mice and rats: a systematic scoping review |  |
| 2. | Authors (names, affiliations, contributions) | **Maria Reiber**  Institute of Pharmacology, Toxicology and Pharmacy  Ludwig-Maximilians-University Munich  **Cathalijn Leenaars**  Radboud University Medical Centre & Utrecht University &  Hanover Medical School  **Hannah King**  Institute of Pharmacology, Toxicology and Pharmacy  Ludwig-Maximilians-University Munich  **Helen Stirling**  Institute of Pharmacology, Toxicology and Pharmacy  Ludwig-Maximilians-University Munich  **Katharina Aulehner**  Institute of Pharmacology, Toxicology and Pharmacy  Ludwig-Maximilians-University Munich  **Paulin Jirkof**  Department Animal Welfare, University of Zurich  **Verena Buchecker**  Institute of Pharmacology, Toxicology and Pharmacy  Ludwig-Maximilians-University Munich  **Agnes Geißelmann**  University Library  Ludwig-Maximilians-University Munich  **Andre Bleich**  Institute for Laboratory Animal Science  Hanover Medical School  **Marion Bankstahl**  Institute for Laboratory Animal Science  Hanover Medical School  **Heidrun Potschka**  Institute of Pharmacology, Toxicology and Pharmacy  Ludwig-Maximilians-University Munich |  |
| 3. | Other contributors (names, affiliations, contributions) | None. |  |

| 4. | Contact person + e-mail address | **Heidrun Potschka**  [**potschka@pharmtox.vetmed.uni-muenchen.de**](mailto:potschka@pharmtox.vetmed.uni-muenchen.de) | |  |
| --- | --- | --- | --- | --- |
| 5. | Funding sources/sponsors | DFG FOR2591 | |  |
| 6. | Conflicts of interest | None | |  |
| 7. | Date and location of protocol registration | 29-10-2020, SYRF website. | |  |
| 8. | Registration number (if applicable) | - | |  |
| 9. | Stage of review at time of registration | Searches performed, screening not yet started | |  |
|  | B. Objectives | | | |
|  | Background | | | |
| 10. | What is already known about this disease/model/intervention? Why is it important to do this review? | **As one of the most common surgical procedures in neuroscience, experimental craniotomies are performed based on a variety of indications requiring direct access to the brain.**  **Contrary to the popular belief that intracranial surgeries cause less severe pain than other surgical procedures, human patients** **mostly report moderate to severe pain after brain surgeries. Recent findings indicate that, in human patients, craniotomy associated pain is often poorly treated.**  **With regard to the respective analgesic regimen in laboratory rodents, this is particularly interesting in light of the frequent oligoanalgesia and rare multimodal analgesic approaches used. Furthermore, no official guidelines exist as of yet for the specific analgesic treatment of craniotomy-associated pain in laboratory mice and rats.**  **We will conduct a systematic scoping review to provide an overview of the anesthetic and analgesic** **management of craniotomy-associated pain in laboratory mice and rats, identifying the current state, trends and improvements of the respective pain management strategies over the past two decades. We will focus on the specific pharmacological pre-, peri- and postoperative treatments (monotherapy versus multimodal approaches), on the non-pharmacological refinement measures, and on the application of post-surgical pain assessment tools. Thus, this scoping review aims to obtain information about current practices and to provide a basis for recommendations for multimodal pain management approaches for craniotomy in laboratory mice and rats.** | |  |
|  | Research question | | | |
| 11. | Specify the disease/health problem of interest | No specific disease or health problem is studied. | |  |
| 12. | Specify the population/species studied | Laboratory mouse and rat. | |  |
| 13. | Specify the intervention/exposure | Craniotomy. | |  |
| 14. | Specify the control population | Any or none. | |  |
| 15. | Specify the outcome measures | For the study selection: any.  For this SR: we will record anesthesia and analgesia (administration route and drug/compound used, specific pharmaceutical formulation (e.g. sustained release), dosage, duration of treatment, administration interval), other pain management techniques, surgical technique, duration of surgery, peri-operative care (including antibiotic use), refinement measures,  assessment method of the efficacy of pain alleviating measures. | |  |
| 16. | State your research question (based on items 11-15) | What are the common approaches to anesthetic and analgesic management for experimental craniotomy in mice and rats? | |  |
|  | C. Methods | | | |
|  | Search and study identification | | | |
| 17. | Identify literature databases to search (*e.g.* Pubmed, Embase, Web of science) | **MEDLINE via PubMed**  Web of Science  EMBASE  SCOPUS  Other, namely: Medline via OVID  Specific journal(s), namely: | |  |
| 18. | Define electronic search strategies (*e.g.* use the [step by step search guide^15^](http://www.ncbi.nlm.nih.gov/pmc/articles/PMC3265183/pdf/LA-11-087.pdf) and animal search filters[^20,^](http://www.ncbi.nlm.nih.gov/pmc/articles/PMC3104815/pdf/LA-09-117.pdf) [^21^](http://lan.sagepub.com/content/48/1/88.full.pdf+html)) | The search string can be found at the end of the document and consists of 2 search elements: the investigated population (mice and rats) and the intervention (craniotomy). | |  |
| 19. | Identify other sources for study identification | Reference lists of included studies □Books  □Reference lists of relevant reviews  □Conference proceedings, namely:  □Contacting authors/ organisations, namely:  □Other, namely: | |  |
| 20. | Define search strategy for these other sources | - | |  |
|  | Study selection | | | |
| 21. | Define screening phases (*e.g.* pre-screening based on title/abstract, full text screening, both) | 1. First screening phase: title/abstract  2. Second screening phase: full text | |  |
| 22. | Specify (a) the number of reviewers per screening phase and (b) how discrepancies will be resolved | 2. Reviewers for each phase. Discrepancies will be resolved by discussion, if needed with a third reviewer. | |  |
|  | *Define all inclusion and exclusion criteria based on:* | | | |
| 23. | Type of study (design) | Inclusion criteria: original experimental data, in vivo studies  Exclusion criteria: other study types. | |  |
| 24. | Type of animals/population (*e.g.* age, gender, disease model) | Inclusion criteria:  mice (*Mus musculus)* & rats *(Rattus norvegicus)*  Exclusion criteria: Other Species. | |  |
| 25. | Type of intervention (*e.g.* dosage, timing, frequency) | Inclusion criteria: craniotomy  Exclusion criteria: other surgeries (e.g. Peripheral neurosurgery, Medulla spinalis surgery, Denervation, Foraminotomie, Laminectomy, Laminoplasty, Nerve Transfer, Radiosurgery) | |  |
| 26. | Outcome measures | Inclusion criteria: any.  Exclusion criteria: none. | |  |
| 27. | Language restrictions | Inclusion criteria: publication in English.  Exclusion criteria: not English. | |  |
| 28. | Publication date restrictions | Inclusion criteria: year of publication 2019, 2009  Exclusion criteria: other years of publication | |  |
| 29. | Other | Screening will be completed for all papers with a publication date in 2019 or 2009.  Full inclusion and analyses will be restricted to a random subset of at least 100 papers per year of interest. | |  |
| 30. | Sort and prioritize your exclusion criteria per selection phase | Selection phase within title and abstract:   1. No English language 2. No mice and/or no rats 3. No craniotomy   Selection phase within full text:   1. No English language 2. No mice and/or no rats 3. No craniotomy 4. No original in vivo data | |  |
|  | Study characteristics to be extracted (for assessment of external validity, reporting quality) | | | |
| 31. | Study ID (*e.g.* authors, year) | First author, title, year, journal, issue, pages | |  |
| 32. | Study design characteristics (*e.g.* experimental groups, number of animals) | - Number of animals/group (for all groups) - Background/purpose of craniotomy   (specify if a project is focussed on analgesic efficacy as this can affect choice of the analgesic approach in subgroups related to use of control groups with oligoanalgesia or no analgesia) | |  |
| 33. | Animal model characteristics (*e.g.* species, gender, disease induction) | For all subgroups:   - Species - Sex - Breeder - Strain - Age - Body weight - Housing condition (temperature, type of cage, enrichment, light schedule, humidity) - Handling technique - Mortality - Duration of experiment - Fate of the used animals | |  |
| 34. | Intervention characteristics (*e.g.* intervention, timing, duration) | - Type of craniotomy procedure - Additional surgical interventions (e.g. telemetry transmitters etc) - Surgical technique - Duration of surgery - General anaesthesia and local anaesthesia - Analgesia (administration route and drug/compound used, specific pharmaceutical formulation (e.g. sustained release) dosage, duration of treatment, administration interval, number of analgesic agents used) - Other pain management techniques - Peri-operative care (including antibiotic use) - Non-pharmacological analgesic measures - Refinement measures - Antibiotic administration - Monitoring protocol | |  |
| 35. | Outcome measures | - Assessment of the efficacy of pain alleviating measures (yes/no/not reported) - Assessment of analgesic efficacy (yes/no/not reported), if yes: parameters testing efficacy of pain/stress reducing measures and use of a control group with no or limited analgesia | |  |
| 36. | Other (*e.g.* drop-outs) | - | |  |
|  | Assessment risk of bias (internal validity) or study quality | | | |
| 37. | Specify (a) the number of reviewers assessing the risk of bias/study quality in each study and (b) how discrepancies will be resolved | One reviewer will check reporting. Accuracy will be confirmed by a second reviewer using a random sample of 5% of the included papers. | |  |
| 38. | Define criteria to assess (a) the internal validity of included studies (*e.g.* selection, performance, detection and attrition bias) and/or (b) other study quality measures (*e.g.* reporting quality, power) | □By use of [SYRCLE's Risk of Bias tool^4^](http://www.biomedcentral.com/1471-2288/14/43/abstract)  □By use of SYRCLE’s Risk of Bias tool, adapted as follows:  □By use of [CAMARADES' study quality checklist, e.g ^22^](http://www.ncbi.nlm.nih.gov/pubmed/15060322)  □By use of CAMARADES' study quality checklist, adapted as follows:  Other criteria, namely:  For a scoping review of analgesia protocols, the standard tools are hardly applicable. We will estimate:   - Check if **power analysis** is reported - Check if **blinding and randomisation** are reported | |  |
|  | Collection of outcome data | | | |
| 39. | For each outcome measure, define the type of data to be extracted (*e.g.* continuous/dichotomous, unit of measurement) | Study characteristics listed in 31. to 35. will be recorded in a table and summarized qualitatively. | |  |
| 40. | Methods for data extraction/retrieval (*e.g.* first extraction from graphs using a digital screen ruler, then contacting authors) | Study characteristics and data will be extracted from text and graphs. Characteristics provided by referencing another publication will be tracked for one level. If the information is not provided in the referenced article (indirect referencing) it will be recorded as not reported. | |  |
| 41. | Specify (a) the number of reviewers extracting data and (b) how discrepancies will be resolved | One reviewer will extract data and a random sample of 5% of data will be analysed by a second reviewer. | |  |
|  | Data analysis/synthesis | | | |
| 42. | Specify (per outcome measure) how you are planning to combine/compare the data (*e.g.* descriptive summary, meta-analysis) | The results will be tabulated and used to give a descriptive overview of anesthesia and analgesia for craniotomy in laboratory mice and rats. | |  |
| 43. | Specify (per outcome measure) how it will be decided whether a meta-analysis will be performed | No meta-analysis will be performed. | |  |
|  | *If a meta-analysis seems feasible/sensible, specify (for each outcome measure):* | | | |
| 44. | The effect measure to be used (*e.g.* mean difference, standardized mean difference, risk ratio, odds ratio) | - | |  |
| 45. | The statistical model of analysis (*e.g.* random or fixed effects model) | - | |  |
| 46. | The statistical methods to assess heterogeneity (*e.g.* I^2^, Q) | - | |  |
| 47. | Which study characteristics will be examined as potential source of heterogeneity (subgroup analysis) | - | |  |
| 48. | Any sensitivity analyses you propose to perform | - | |  |
| 49. | Other details meta-analysis (*e.g.* correction for multiple testing, correction for multiple use of control group) | - | |  |
| 50. | The method for assessment of publication bias | - | |  |
|  | | | | |
| Final approval by (names, affiliations): Heidrun Potschka, Inst. of Pharmacology, Toxicology, and Pharmacy, Ludwig-Maximilians-University Munich | |  | Date: 29.10.2020 | |

**Supplementary Methods 2: PRISMA (Preferred Reporting Items for Systematic Reviews and Meta-Analyses) Checklist Scoping Reviews**

**Preferred Reporting Items for Systematic reviews and Meta-Analyses extension for Scoping Reviews (PRISMA-ScR) Checklist**

| **SECTION** | **ITEM** | **PRISMA-ScR CHECKLIST ITEM** | **REPORTED ON PAGE #** |
| --- | --- | --- | --- |
| **TITLE** | | | |
| Title | 1 | Identify the report as a scoping review. | 1 |
| **ABSTRACT** | | | |
| Structured summary | 2 | Provide a structured summary that includes (as applicable): background, objectives, eligibility criteria, sources of evidence, charting methods, results, and conclusions that relate to the review questions and objectives. | 2 |
| **INTRODUCTION** | | | |
| Rationale | 3 | Describe the rationale for the review in the context of what is already known. Explain why the review questions/objectives lend themselves to a scoping review approach. | 3-5 |
| Objectives | 4 | Provide an explicit statement of the questions and objectives being addressed with reference to their key elements (e.g., population or participants, concepts, and context) or other relevant key elements used to conceptualize the review questions and/or objectives. | 3-5 |
| **METHODS** | | | |
| Protocol and registration | 5 | Indicate whether a review protocol exists; state if and where it can be accessed (e.g., a Web address); and if available, provide registration information, including the registration number. | 6 |
| Eligibility criteria | 6 | Specify characteristics of the sources of evidence used as eligibility criteria (e.g., years considered, language, and publication status), and provide a rationale. | 7-8 |
| Information sources* | 7 | Describe all information sources in the search (e.g., databases with dates of coverage and contact with authors to identify additional sources), as well as the date the most recent search was executed. | 6 |
| Search | 8 | Present the full electronic search strategy for at least 1 database, including any limits used, such that it could be repeated. | 6-7 |
| Selection of sources of evidence† | 9 | State the process for selecting sources of evidence (i.e., screening and eligibility) included in the scoping review. | 7 |
| Data charting process‡ | 10 | Describe the methods of charting data from the included sources of evidence (e.g., calibrated forms or forms that have been tested by the team before their use, and whether data charting was done independently or in duplicate) and any processes for obtaining and confirming data from investigators. | 7-9 |
| Data items | 11 | List and define all variables for which data were sought and any assumptions and simplifications made. | 7-9 |
| Critical appraisal of individual sources of evidence§ | 12 | If done, provide a rationale for conducting a critical appraisal of included sources of evidence; describe the methods used and how this information was used in any data synthesis (if appropriate). | Click here to enter text. |
| Synthesis of results | 13 | Describe the methods of handling and summarizing the data that were charted. | 9 |
| **RESULTS** | | | |
| Selection of sources of evidence | 14 | Give numbers of sources of evidence screened, assessed for eligibility, and included in the review, with reasons for exclusions at each stage, ideally using a flow diagram. | 10 |
| Characteristics of sources of evidence | 15 | For each source of evidence, present characteristics for which data were charted and provide the citations. | 10-28 |
| Critical appraisal within sources of evidence | 16 | If done, present data on critical appraisal of included sources of evidence (see item 12). | Click here to enter text. |
| Results of individual sources of evidence | 17 | For each included source of evidence, present the relevant data that were charted that relate to the review questions and objectives. | 10-28 |
| Synthesis of results | 18 | Summarize and/or present the charting results as they relate to the review questions and objectives. | 10-28 |
| **DISCUSSION** | | | |
| Summary of evidence | 19 | Summarize the main results (including an overview of concepts, themes, and types of evidence available), link to the review questions and objectives, and consider the relevance to key groups. | 29 |
| Limitations | 20 | Discuss the limitations of the scoping review process. | 36 |
| Conclusions | 21 | Provide a general interpretation of the results with respect to the review questions and objectives, as well as potential implications and/or next steps. | 37 |
| **FUNDING** | | | |
| Funding | 22 | Describe sources of funding for the included sources of evidence, as well as sources of funding for the scoping review. Describe the role of the funders of the scoping review. | 44 |

JBI = Joanna Briggs Institute; PRISMA-ScR = Preferred Reporting Items for Systematic reviews and Meta-Analyses extension for Scoping Reviews.

* Where *sources of evidence* (see second footnote) are compiled from, such as bibliographic databases, social media platforms, and Web sites.

† A more inclusive/heterogeneous term used to account for the different types of evidence or data sources (e.g., quantitative and/or qualitative research, expert opinion, and policy documents) that may be eligible in a scoping review as opposed to only studies. This is not to be confused with *information sources* (see first footnote).

‡ The frameworks by Arksey and O’Malley (6) and Levac and colleagues (7) and the JBI guidance (4, 5) refer to the process of data extraction in a scoping review as data charting*.*

§ The process of systematically examining research evidence to assess its validity, results, and relevance before using it to inform a decision. This term is used for items 12 and 19 instead of "risk of bias" (which is more applicable to systematic reviews of interventions) to include and acknowledge the various sources of evidence that may be used in a scoping review (e.g., quantitative and/or qualitative research, expert opinion, and policy document).

*From:* Tricco AC, Lillie E, Zarin W, O'Brien KK, Colquhoun H, Levac D, et al. PRISMA Extension for Scoping Reviews (PRISMAScR): Checklist and Explanation. Ann Intern Med. 2018;169:467–473. [doi: 10.7326/M18-0850](http://annals.org/aim/fullarticle/2700389/prisma-extension-scoping-reviews-prisma-scr-checklist-explanation).

**Supplementary Methods 3: Search strategy for Pubmed**

**Medline via Pubmed**

1. **Surgery**

"neurosurgical procedures"[MeSH Terms:noexp] OR neurosurgical*[tiab] OR "anterior temporal lobectomy"[MeSH Terms] OR temporal lobectomy[tiab] OR temporal lobectomies[tiab] OR ATL[tiab] OR ATLs[tiab] OR "brain tissue transplantation"[MeSH Terms] OR brain tissue transplantation[tiab] OR brain tissue transplantations[tiab] OR brain tissue grafting[tiab] OR "cerebral decortication"[MeSH Terms] OR cerebral decortication[tiab] OR cerebral decortications[tiab] OR cerebral cortex decortication[tiab] OR "hemispherectomy"[MeSH Terms] OR hemispherectomy[tiab] OR hemispherectomies[tiab] OR "cerebrospinal fluid shunts"[MeSH Terms] OR cerebrospinal fluid shunt[tiab] OR cerebrospinal fluid shunts[tiab] OR CSF shunt[tiab] OR CSF shunts[tiab] OR CSF pressure[tiab] OR CSF pressures[tiab] OR "ventriculoperitoneal shunt"[MeSH Terms] OR ventriculoperitoneal shunt[tiab] OR ventriculoperitoneal shunts[tiab] OR ventriculo-peritoneal shunt[tiab] OR ventriculo-peritoneal shunts[tiab] OR "ventriculostomy"[MeSH Terms] OR ventriculostomy[tiab] OR ventriculostomies[tiab] OR ventriculocisternostomy[tiab] OR ventriculocisternostomies[tiab] OR "craniotomy"[MeSH Terms] OR craniotomy[tiab] OR craniotomies[tiab] OR "trephining"[MeSH Terms] OR trephining[tiab] OR trephinings[tiab] OR trephination[tiab] OR trephinations[tiab] OR trepanation[tiab] OR trepanations[tiab] OR trepanning[tiab] OR craniectomy[tiab] OR craniectomies[tiab] OR hemicraniectomy[tiab] OR hemicraniectomies[tiab] OR hippocampectomy[tiab] OR hippocampectomies[tiab] OR callostomy[tiab] OR thalamotomy[tiab] OR thalamotomies[tiab] OR subthalamotomy[tiab] OR subthalamotomies[tiab] OR cranioplasty[tiab] OR cranioplasties[tiab] OR thalamic ablation[tiab] OR thalamic ablations[tiab] OR "hypophysectomy"[MeSH Terms] OR hypophysectomy[tiab] OR hypophysectomies[tiab] OR "neuroendoscopy"[MeSH Terms] OR neuroendoscopy[tiab] OR neuroendoscopies[tiab] OR "pallidotomy"[MeSH Terms] OR pallidotomy[tiab] OR pallidotomies[tiab] OR "psychosurgery"[MeSH Terms] OR psychosurgery[tiab] OR psychosurgeries[tiab] OR lobotomy[tiab] OR lobotomies[tiab] OR gyrectomy[tiab] OR gyrectomies[tiab] OR leukotomy[tiab] OR leukotomies[tiab] OR leucotomy[tiab] OR leucotomies[tiab] OR topectomy[tiab] OR topectomies[tiab] OR "split brain procedure"[MeSH Terms] OR split brain[tiab] OR split-brain[tiab] OR "stereotaxic techniques"[mh:noexp] OR stereotaxic*[tiab] OR stereotactic*[tiab] OR stereotaxy[tiab] OR stereotaxies[tiab] OR "neuronavigation"[MeSH Terms] OR neuronavigation[tiab] OR "neurosurgery"[MeSH Terms] OR neurosurgery[tiab] OR neurosurgeries[tiab] OR microneurosurgery[tiab] OR "deep brain stimulation"[MeSH Terms] OR deep brain stimulation[tiab] OR deep brain stimulations[tiab] OR DBS[tiab] OR deep brain stimulator[tiab] OR deep brain stimulators[tiab] OR brain pacemaker[tiab] OR brain pacemakers[tiab] OR "electroencephalography"[MeSH Terms] OR electroencephalography[tiab] OR electroencephalographic[tiab] OR electroencephalographical[tiab] OR electroencephalogram[tiab] OR electroencephalograms[tiab] OR eeg[tiab] OR eegs[tiab] OR electrocorticography[tiab] OR electrocorticographical[tiab] OR electrocorticogram[tiab] OR electrocorticograms[tiab] OR ECOG[tiab] OR ECOGs[tiab] OR stereoelectroencephalography[tiab] OR "polysomnography"[MeSH Terms] OR polysomnograph*[tiab] OR sleep monitoring[tiab] OR somnograph*[tiab] OR kindling[tiab] OR kindled[tiab] OR "telemetry"[MeSH Terms] OR telemetry[tiab] OR telemetries[tiab] OR telemetric*[tiab] OR "brain injuries, traumatic"[MeSH Terms] OR traumatic brain injury[tiab] OR traumatic brain injuries[tiab] OR TBI[tiab] OR TBIs[tiab] OR traumatic encephalopathy[tiab] OR traumatic encephalopathies[tiab] OR controlled cortical impact[tiab] OR CCI[tiab] OR controlled cortical impacts[tiab] OR CCIs[tiab] OR bone flap[tiab] OR bone flaps[tiab] OR flap surgery[tiab] OR flap surgeries[tiab] OR skull trephine[tiab] OR cranial trephine[tiab] OR acoustic neuroma resection[tiab] OR acoustic neuroma resections[tiab] OR durotomy[tiab] OR durotomies[tiab] OR duratomy[tiab] OR cranial surgery[tiab] OR cranial surgeries[tiab] OR cranial procedure[tiab] OR cranial procedures[tiab] OR cranial operation[tiab] OR cranial operations[tiab] OR cranial resection[tiab] OR cranial resections[tiab] OR intracranial surgery[tiab] OR intracranial surgeries[tiab] OR intracranial procedure[tiab] OR intracranial procedures[tiab] OR intracranial operation[tiab] OR intracranial operations[tiab] OR intracranial resection[tiab] OR transcranial surgery[tiab] OR transcranial surgeries[tiab] OR transcranial procedure[tiab] OR transcranial procedures[tiab] OR transcranial operation[tiab] OR transcranial operations[tiab] OR transcranial resection[tiab] OR skull surgery[tiab] OR skull operations[tiab] OR skull resection[tiab] OR skull resections[tiab] OR base surgery[tiab] OR base surgeries[tiab] OR base procedure[tiab] OR base procedures[tiab] OR base operation[tiab] OR base operations[tiab] OR base resection[tiab] OR base resections[tiab] OR brain surgery[tiab] OR brain surgeries[tiab] OR brain procedures[tiab] OR brain operation[tiab] OR brain operations[tiab] OR brain resection[tiab] OR brain resections[tiab] OR cerebral surgery[tiab] OR cerebral procedures[tiab] OR cerebral operations[tiab] OR cerebral resection[tiab] OR cerebral resections[tiab] OR cns surgery[tiab] OR cns surgeries[tiab] OR cns procedures[tiab] OR cns operation[tiab] OR cerebrovascular surgery[tiab] OR cerebrovascular surgeries[tiab] OR cerebrovascular procedure[tiab] OR cerebrovascular procedures[tiab] OR cerebrovascular operations[tiab] OR decompression surgery[tiab] OR decompression surgeries[tiab] OR decompression procedure[tiab] OR decompression procedures[tiab] OR decompression operation[tiab] OR decompression operations[tiab] OR suboccipital surgery[tiab] OR suboccipital operation[tiab] OR suboccipital operations[tiab] OR suboccipital resection[tiab] OR transsphenoidal surgery[tiab] OR transsphenoidal surgeries[tiab] OR transsphenoidal procedure[tiab] OR transsphenoidal procedures[tiab] OR transsphenoidal operation[tiab] OR transsphenoidal operations[tiab] OR transsphenoidal resection[tiab] OR transsphenoidal resections[tiab] OR brainstem surgery[tiab] OR brain stem surgery[tiab] OR temporal lobe resection[tiab] OR temporal lobe resections[tiab] OR temporal lobe surgery[tiab] OR temporal lobe surgeries[tiab] OR cranial window[tiab] OR cranial windows[tiab] OR cranial implant[tiab] OR cranial implants[tiab] OR cranial electrode[tiab] OR cranial electrodes[tiab] OR intracranial implant[tiab] OR intracranial implants[tiab] OR intracranial electrode[tiab] OR intracranial electrodes[tiab] OR skull implant[tiab] OR skull implants[tiab] OR skull electrodes[tiab] OR cerebral implants[tiab] OR cerebral implantation[tiab] OR cerebral electrodes[tiab] OR cns implants[tiab] OR cns implantation[tiab] OR cns electrodes[tiab] OR brain implant[tiab] OR brain implants[tiab] OR brain implantation[tiab] OR brain implantations[tiab] OR brain electrode[tiab] OR brain electrodes[tiab] OR brain microelectrode[tiab] OR brainstem implant[tiab] OR brainstem implants[tiab] OR brainstem implantation[tiab] OR brainstem implantations[tiab] OR brainstem electrode[tiab] OR brain stem implant[tiab] OR brain stem implants[tiab] OR brain stem implantation[tiab] OR cerebral injection[tiab] OR cerebral injections[tiab] OR cerebral application[tiab] OR cerebral applications[tiab] OR cerebral administration[tiab] OR cns injection[tiab] OR cns injections[tiab] OR cns application[tiab] OR cns applications[tiab] OR cns administration[tiab] OR brain injection[tiab] OR brain injections[tiab] OR brain cannulation[tiab] OR brain application[tiab] OR brain applications[tiab] OR brain administration[tiab] OR brainstem injection[tiab] OR brainstem injections[tiab] OR brainstem application[tiab] OR brain stem injections[tiab] OR brain stem application[tiab] OR intraamygdala injection[tiab] OR intraamygdala injections[tiab] OR intraamygdala administration[tiab] OR intra-amygdala injection[tiab] OR intra-amygdala injections[tiab] OR intra-amygdala administration[tiab] OR ICV injection[tiab] OR ICV injections[tiab] OR ICV cannulation[tiab] OR ICV application[tiab] OR ICV administration[tiab] OR ICV administrations[tiab] OR kainate[tiab] OR kainic acid[tiab]

1. **Rodents**

"mice"[MeSH Terms] OR mice[tiab] OR mouse[tiab] OR murine[tiab] OR murines[tiab] OR muridae[tiab] OR mus[tiab] OR m. musculus[tiab] OR "rats"[MeSH Terms] OR rats[tiab] OR rat[tiab] OR rattus[tiab] OR "rodentia"[MeSH Terms] OR rodentia[tiab] OR rodent[tiab] OR rodents[tiab]

**Supplementary table S1: List of subset of 200 studies, included studies from 2009 and evaluated parameters**

Information on all evaluated parameters of studies in 2009, included in the subset of 200 studies. n.r.= not reported, parameter was not reported; n.a.= not applicable, extraction of this parameter was not feasible; i.p.= intraperitoneal; i.m.= intramuscular; s.c.= subcutaneous; tbi= traumatic brain injury; cci= controlled cortical impact.

| study ID | first author (last name) | title | year of publication (2009, 2019) | journal (in which the study was published)  (name) | issue (number or n.r.) | pages or article number | country of origin (location of the institute the first author worked for at timepoint of publication)  (name of country) |
| --- | --- | --- | --- | --- | --- | --- | --- |
| Bartolomucci et al. | Bartolomucci | Chronic intracerebroventricular injection of TLQP-21 prevents high fat diet induced weight gain in fast weight-gaining mice | 2009 | Genes Nutritional | 4 | 49 to 57 | Italy |
| Behrend et al. | Behrend | Toward feedback controlled deep brain stimulation: Dynamics of glutamate release in the subthalamic nucleus in rats | 2009 | Journal of Neuroscience Methods | 180 | 278 to 289 | USA |
| Biella et al. | Biella | Probing for local activity-related modulation of the infrared backscattering of the brain cortex | 2009 | Journal of Biophotonics | 2 | 588 to 595 | Italy |
| Boni et al. | Boni | The in vivo effect of VIP, PACAP-38 and PACAP-27 and mRNA expression of their receptors in rat middle meningeal artery | 2009 | Cephalalgia | 29 | 837 to 847 | Denmark |
| Bramlett et al. | Bramlett | Sex differences in XIAP cleavage after traumatic brain injury in the rat | 2009 | Neuroscience Letters | 461 | 49 to 53 | USA |
| Byun et al. | Byun | Kainic Acid-induced Neuronal Death is Attenuated by Aminoguanidine but Aggravated by L-NAME in Mouse Hippocampus | 2009 | Korean Journal of physiology & pharmacology | 13 | 265 to 271 | South Korea |
| Caltana et al. | Caltana | Neuronal and glial alterations due to focal cortical hypoxia induced by direct cobalt chloride (CoCl2) brain injection | 2009 | Neurotoxicity Research | 15 | 348 to 358 | Argentina |
| Carcak et al. | Carcak | Effect of stage 2 kindling on local cerebral blood flow rates in rats with genetic absence epilepsy | 2009 | Epilepsia | 50 | 33 to 43 | Turkey |
| Cemil et al. | Cemil | The effect of mitomycin C as fibrosis preventive agent during craniectomies | 2009 | British Journal of Neurosurgery | 23 | 304 to 308 | Turkey |
| Chen et al. D | Chen | GluR6-containing KA receptor mediates the activation of p38 MAP kinase in rat hippocampal CA1 region during brain ischemia injury | 2009 | Hippocampus | 19 | 79 to 89 | China |
| Cifani et al. | Cifani | Possible common central pathway for resistin and insulin in regulating food intake | 2009 | Acta Physiologica | 196 | 395 to 400 | Italy |
| Cunningham et al. | Cunningham | Microglia and the urokinase plasminogen activator receptor/uPA system in innate brain inflammation | 2009 | Glia | 57 | 1802 to 1814 | Italy |
| Datta et al. | Datta | Identification of cholinergic and non-cholinergic neurons in the pons expressing phosphorylated cyclic adenosine monophosphate response element-binding protein as a function of rapid eye movement sleep | 2009 | Neuroscience | 163 | 397 to 414 | USA |
| Diesch et al. | Diesch | Electroencephalographic responses to tail clamping in anaesthetized rat pups | 2009 | Laboratory Animals | 43 | 224 to 231 | New Zealand |
| Diguet et al. | Diguet | Normal aging modulates the neurotoxicity of mutant huntingtin | 2009 | PLoS One | 4 | 4637 | France |
| Ding et al. | Ding | Sensitization of ventral tegmental area dopamine neurons to the stimulating effects of ethanol | 2009 | Alcoholism, clinical and experimental research | 33 | 1571 to 1581 | USA |
| Doan et al. | Doan | Simultaneous two-voxel localized (1)H-observed (13)C-edited spectroscopy for in vivo MRS on rat brain at 9.4T: Application to the investigation of excitotoxic lesions | 2009 | Journal of Magnetic Resonance | 198 | 94 to 104 | France |
| Doretto et al. | Doretto | Role of the superior colliculus in the expression of acute and kindled audiogenic seizures in Wistar audiogenic rats | 2009 | Epilepsia | 50 | 2563 to 2574 | Brazil |
| Dux et al. | Dux | Involvement of capsaicin-sensitive afferent nerves in the proteinase-activated receptor 2-mediated vasodilatation in the rat dura mater | 2009 | Neuroscience | 161 | 887 to 894 | Hungary |
| Echegoyen et al. | Echegoyen | Single application of a CB1 receptor antagonist rapidly following head injury prevents long-term hyperexcitability in a rat model | 2009 | Epilepsy Research | 85 | 123 to 127 | USA |
| Ehrlichman et al. | Ehrlichman | N-methyl-d-aspartic acid receptor antagonist-induced frequency oscillations in mice recreate pattern of electrophysiological deficits in schizophrenia | 2009 | Neuroscience | 158 | 705to 712 | USA |
| Etholm et al. | Etholm | Seizure elements and seizure element transitions during tonic-clonic seizure activity in the synapsin I/II double knockout mouse: a neuroethological description | 2009 | Epilepsy & Behavior | 14 | 582 to 590 | Norway |
| Farias et al. | Farias | Injury-related production of cysteinyl leukotrienes contributes to brain damage following experimental traumatic brain injury | 2009 | Journal of Neurotrauma | 26 | 1977 to 1986 | USA |
| Foti et al. | Foti | Delivering multiple gene products in the brain from a single adeno-associated virus vector | 2009 | Gene Therapy | 16 | 1314 to 1319 | USA |
| Francois et al. | Francois | Selective reorganization of GABAergic transmission in neonatal ventral hippocampal-lesioned rats | 2009 | International Journal of Neuropsychopharmacology | 12 | 1097 to 1110 | France |
| Francois et al. | Francois | Selective reorganization of GABAergic transmission in neonatal ventral hippocampal-lesioned rats | 2009 | International Journal of Neuropsychopharmacology | 12 | 1097 to 1110 | France |
| Fritsch et al. | Fritsch | Pathological alterations in GABAergic interneurons and reduced tonic inhibition in the basolateral amygdala during epileptogenesis | 2009 | Neuroscience | 29 | 415 to 429 | USA |
| Good et al. | Good | Control of synchronization of brain dynamics leads to control of epileptic seizures in rodents | 2009 | International Journal of neural systems | 19 | 173 to 196 | USA |
| Griesbach et al. | Griesbach | Exercise-induced improvement in cognitive performance after traumatic brain injury in rats is dependent on BDNF activation | 2009 | Brain Research | 1288 | 105 to 115 | USA |
| Guidine et al. | Guidine | Electroencephalographic evidence of brainstem recruitment during scorpion envenomation | 2009 | NeuroToxicology | 30 | 90 to 96 | Brazil |
| Gurevicius et al. | Gurevicius | Genetic ablation of tenascin-C expression leads to abnormal hippocampal CA1 structure and electrical activity in vivo | 2009 | Hippocampus | 19 | 1232 to 1246 | Finland |
| Hart et al. | Hart | Systemic or intra-amygdala injection of a benzodiazepine (midazolam) impairs extinction but spares re-extinction of conditioned fear responses | 2009 | Learning Memory | 16 | 53 to 61 | Australia |
| Harvey et al. | Harvey | Intracellular dynamics of hippocampal place cells during virtual navigation | 2009 | Nature | 461 | 941 to 946 | USA |
| Hernandez-Gonzalez et al. | Hernandez-Gonzalez | Ethanol changes the electroencephalographic correlation of the ventral tegmental area and nucleus accumbens, components of the mesoaccumbens system in rats | 2009 | Pharmacology, Biochemistry and Behavior | 92 | 124 to 130 | Mexico |
| Ho et al. | Ho | EphB2 and EphA4 receptors regulate formation of the principal inter-hemispheric tracts of the mammalian forebrain | 2009 | Neuroscience | 160 | 784 to 795 | Canada |
| Holtmaat et al. | Holtmaat | Long-term, high-resolution imaging in the mouse neocortex through a chronic cranial window | 2009 | Nature protocols | 4 | 1128 to 1144 | USA |
| Hrncic et al. | Hrncic | Influence of NR2B-selective NMDA antagonist on lindane-induced seizures in rats | 2009 | Pharmacology | 84 | 234 to 239 | Serbia |
| Huguet et al. | Huguet | Intracranial self-stimulation to the lateral hypothalamus, a memory improving treatment, results in hippocampal changes in gene expression | 2009 | Neuroscience | 162 | 359 to 374 | Spain |
| Ishida et al. | Ishida | Effects of some antipsychotics and a benzodiazepine hypnotic on the sleep-wake pattern in an animal model of schizophrenia | 2009 | Journal of Pharmacological Sciences | 111 | 44 to 52 | Japan |
| Ito et al. | Ito | Acquisition of brain Na sensitivity contributes to salt-induced sympathoexcitation and cardiac dysfunction in mice with pressure overload | 2009 | Circulation Research | 104 | 1004 to 1011 | Japan |
| Itoh et al. B | Itoh | The novel free radical scavenger, edaravone, increases neural stem cell number around the area of damage following rat traumatic brain injury | 2009 | Neurotoxicity Research | 16 | 378 to 389 | Japan |
| Itoh et al. A | Itoh | Expression of amyloid precursor protein after rat traumatic brain injury | 2009 | Neurological Research | 31 | 103 to 109 | Japan |
| Jafri et al. | Jafri | Optical coherence tomography guided neurosurgical procedures in small rodents | 2009 | Journal of Neuroscience Methods | 176 | 85 to 95 | USA |
| Kalauzi et al. | Kalauzi | Cortico-pontine theta synchronization phase shift following monoaminergic lesion in rat | 2009 | Journal of Physiology and Pharmacology | 60 | 79 to 84 | Serbia |
| Katz et al. | Katz | Independence of brain and trunk temperature during hypothermic preconditioning in rats | 2009 | Journal of Neuroscience Methods | 179 | 179 to 183 | USA |
| Kim et al. A | Kim | Localization of the transcription factor, sterol regulatory element binding protein-2 (SREBP-2) in the normal rat brain and changes after kainate-induced excitotoxic injury | 2009 | Journal of Chemical Neuroanatomy | 37 | 71 to 77 | Singapore |
| Lackovic et al. | Lackovic | Single intracerebroventricular injection of botulinum toxin type A produces slow onset and long-term memory impairment in rats | 2009 | Journal of Neural Transmission | 116 | 1273 to 1280 | Croatia |
| Lee et al. B | Lee | Simultaneous recording of brain activity and functional connectivity in the mouse brain | 2009 | Annual International Conference of the IEEE Engineering in Medicine and Biology Society | n.a. | 2934 to 2936 | South Korea |
| Lee et al. A | Lee | Inhibition of VEGF receptor 2 increased cell death of dentate hilar neurons after traumatic brain injury | 2009 | Experimental Neurology | 220 | 400 to 403 | USA |
| Li et al. C | Li | Study on changes of MMP-3 expression after brain contusion in rats | 2009 | Legal Medicine | 11 | 176 to 179 | China |
| Liu et al. | Liu | Lamina-specific changes in hippocampal GABA(A)/cBZR and mossy fibre sprouting during and following amygdala kindling in the rat | 2009 | Neurobiology of Disease | 35 | 337 to 347 | Australia |
| Lopez-Martin et al. | Lopez-Martin | The action of pulse-modulated GSM radiation increases regional changes in brain activity and c-Fos expression in cortical and subcortical areas in a rat model of picrotoxin-induced seizure proneness | 2009 | Journal of Neuroscience Research | 87 | 1484 to 1499 | Spain |
| Lu et al. | Lu | NNZ-2566, a glypromate analog, attenuates brain ischemia-induced non-convulsive seizures in rats | 2009 | Journal of Cerebral Blood Flow & Metabolism | 29 | 1924 to 1932 | USA |
| Lundblad et al. | Lundblad | Hemodynamic and histological effects of traumatic brain injury in eNOS-deficient mice | 2009 | Journal of Neurotrauma | 26 | 1953 to 1962 | Sweden |
| Magloire et al. | Magloire | Delayed changes of sleep duration after rewarded olfactory discrimination learning in the rat | 2009 | Behavioral Brain Research | 205 | 568 to 571 | France |
| Mark et al. | Mark | Leptin signaling in the nucleus tractus solitarii increases sympathetic nerve activity to the kidney | 2009 | Hypertension | 53 | 375 to 380 | USA |
| McCracken et al. | McCracken | Nucleus accumbens deep brain stimulation produces region-specific alterations in local field potential oscillations and evoked responses in vivo | 2009 | The Journal of Neuroscience | 29 | 5354 to 5363 | USA |
| Meeren et al. | Meeren | Thalamic lesions in a genetic rat model of absence epilepsy: dissociation between spike-wave discharges and sleep spindles | 2009 | Experimental Neurology | 217 | 25 to 37 | Netherlands |
| Merkler et al. | Merkler | Propagation of spreading depression inversely correlates with cortical myelin content | 2009 | Annals of Neurology | 66 | 355 to 365 | Germany |
| Merkler et al. | Merkler | Propagation of spreading depression inversely correlates with cortical myelin content | 2009 | Annals of Neurology | 66 | 355 to 365 | Germany |
| Mian et al. | Mian | Postimplantation pressure testing and characterization of laser bonded glass/polyimide microjoints | 2009 | Journal of Biomedical Materials Research | 90 | 614 to 620 | USA |
| Mohammadi et al. | Mohammadi | NMDA preconditioning and neuroprotection in vivo: delayed onset of kainic acid-induced neurodegeneration and c-Fos attenuation in CA3a neurons | 2009 | Brain Research | 1256 | 162 to 172 | Kuwait |
| Mollazaedh et al. | Mollazadeh | Micropower CMOS Integrated Low-Noise Amplification, Filtering, and Digitization of Multimodal Neuropotentials | 2009 | IEEE transactions on biomedical circuits | 3 | 1 to 10 | USA |
| Mukherjee et al. | Mukherjee | Chronic alcohol treatment in rats alters sleep by fragmenting periods of vigilance cycling in the light period with extended wakenings | 2009 | Behavioral Brain Research | 198 | 113 to 124 | USA |
| Nehlig et al. | Nehlig | The ketogenic diet has no effect on the expression of spike-and-wave discharges and nutrient transporters in genetic absence epilepsy rats from Strasbourg | 2009 | Journal of Neurochemistry | 109 | 207 to 213 | France |
| Nuki et al. | Nuki | Elastase-induced intracranial aneurysms in hypertensive mice | 2009 | Hypertension | 54 | 1337 to 1344 | USA |
| Onyszchuk et al. | Onyszchuk | Post-acute pathological changes in the thalamus and internal capsule in aged mice following controlled cortical impact injury: a magnetic resonance imaging, iron histochemical, and glial immunohistochemical study | 2009 | Neuroscience Letters | 452 | 204 to 208 | USA |
| Oshima et al. | Oshima | TNF-alpha contributes to axonal sprouting and functional recovery following traumatic brain injury | 2009 | Brain Research | 1290 | 102 to 110 | Japan |
| Potts et al. | Potts | Glutathione peroxidase overexpression does not rescue impaired neurogenesis in the injured immature brain | 2009 | Journal of Neuroscience Research | 87 | 1848 to 1857 | USA |
| Qing et al. | Qing | Brain edema after intracerebral hemorrhage in rats: the role of iron overload and aquaporin 4 | 2009 | Journal of Neurosurgery | 110 | 462 to 468 | China |
| Rahim et al. | Rahim | Efficient gene delivery to the adult and fetal CNS using pseudotyped non-integrating lentiviral vectors | 2009 | Gene Therapy | 16 | 509 to 520 | UK |
| Rimoli et al. | Rimoli | T-type channel blocking properties and antiabsence activity of two imidazo[1,2-b]pyridazine derivatives structurally related to indomethacin | 2009 | Neuropharmacology | 56 | 637 to 646 | Italy |
| Roiko et al. | Roiko | Passive immunization with a nicotine-specific monoclonal antibody decreases brain nicotine levels but does not precipitate withdrawal in nicotine-dependent rats | 2009 | Pharmacology, Biochemistry and Behavior | 93 | 105 to 111 | USA |
| Rudnick et al. | Rudnick | Role of beta2-containing nicotinic acetylcholine receptors in auditory event-related potentials | 2009 | Psychopharmacology | 202 | 745 to 751 | USA |
| Sahin et al. | Sahin | Vagus nerve stimulation suppresses generalized seizure activity and seizure-triggered postictal cardiac rhythm changes in rats | 2009 | Physiological Research | 58 | 345 to 350 | Turkey |
| Samnick et al. | Samnick | Efficacy of systemic radionuclide therapy with p-131I-iodo-L-phenylalanine combined with external beam photon irradiation in treating malignant gliomas | 2009 | Journal of nuclear medicine | 50 | 2025 to 2032 | Germany |
| Sasaki et al. | Sasaki | Dynamic changes in cortical NADH fluorescence in rat focal ischemia: evaluation of the effects of hypothermia on propagation of peri-infarct depolarization by temporal and spatial analysis | 2009 | Neuroscience Letters | 449 | 61 to 65 | Japan |
| Schei et al. | Schei | State-dependent auditory evoked hemodynamic responses recorded optically with indwelling photodiodes | 2009 | Applied optics | 48 | 121-9 | USA |
| Schmid et al. | Schmid | The effects of IL-1 receptor antagonist on beta amyloid mediated depression of LTP in the rat CA1 in vivo | 2009 | Hippocampus | 19 | 670 to 676 | Switzerland |
| Sekiya et al. | Sekiya | Selective vulnerability of adult cochlear nucleus neurons to de-afferentation by mechanical compression | 2009 | Experimental Neurology | 218 | 117 to 123 | Japan |
| Sher et al. | Sher | Bioluminescence imaging of Olig2-neural stem cells reveals improved engraftment in a demyelination mouse model | 2009 | Stem Cells | 27 | 1582 to 1591 | Netherlands |
| Shultz et al. | Shultz | Intracerebroventricular injections of the enteric bacterial metabolic product propionic acid impair cognition and sensorimotor ability in the Long-Evans rat: further development of a rodent model of autism | 2009 | Behavioral Brain Research | 200 | 33 to 41 | Canada |
| Silvani et al. | Silvani | Sleep modulates hypertension in leptin-deficient obese mice | 2009 | Hypertension | 53 | 251 to 255 | Italy |
| Sinton et al. | Sinton | Validation of a novel method to interrupt sleep in the mouse | 2009 | Journal of Neuroscience Methods | 184 | 71 to 78 | USA |
| Song et al. | Song | Lateral parabrachial nucleus mediates shortening of expiration during hypoxia | 2009 | Respiratory Physiology & Neurology | 165 | 1 to 8 | USA |
| Takahashi et al. | Takahashi | Large-scale reorganization of corticofugal fibers after neonatal hemidecortication for functional restoration of forelimb movements | 2009 | European Journal of Neuroscience | 30 | 1878 to 1887 | Japan |
| Takahashi et al. | Takahashi | Large-scale reorganization of corticofugal fibers after neonatal hemidecortication for functional restoration of forelimb movements | 2009 | European Journal of Neuroscience | 30 | 1878 to 1887 | Japan |
| Tanida et al. | Tanida | Possible role of the histaminergic system in autonomic and cardiovascular responses to neuropeptide Y | 2009 | Neuropeptides | 43 | 21 to 29 | Japan |
| Tchekalarova et al. | Tchekalarova | Postnatal caffeine treatment affects differently two pentylenetetrazol seizure models in rats | 2009 | Brain Research Bulletin | 147 | 22 to 35 | Bulgaria |
| Thomas et al. | Thomas | Graft outcomes influenced by co-expression of Pax7 in graft and host tissue | 2009 | Journal of Anatomy | 214 | 396 to 405 | Australia |
| Topchiy et al. | Topchiy | Conditioned lick behavior and evoked responses using whisker twitches in head restrained rats | 2009 | Behavioral Brain Research | 197 | 16 to 23 | USA |
| Touzani et al. | Touzani | Dopamine D1-like receptor antagonism in amygdala impairs the acquisition of glucose-conditioned flavor preference in rats | 2009 | European Journal of Neuroscience | 30 | 289 to 298 | USA |
| Tsanov et al. | Tsanov | Long-term plasticity is proportional to theta-activity | 2009 | PLoS One | 4 | 5850 | Germany |
| Wagner et al. | Wagner | Controlled cortical impact injury influences methylphenidate-induced changes in striatal dopamine neurotransmission | 2009 | Journal of Neurochemistry | 110 | 801 to 810 | USA |
| Wan et al. | Wan | Preserved cerebral microcirculation during cardiogenic shock | 2009 | Critical Care Medicine | 37 | 2333 to 2337 | USA |
| Wigren et al. | Wigren | Basal forebrain lactate release and promotion of cortical arousal during prolonged waking is attenuated in aging | 2009 | The Journal of Neuroscience | 29 | 11698 to 11707 | Finland |
| Worthen et al. | Worthen | In vivo evaluation of diaminodiphenyls: anticonvulsant agents with minimal acute neurotoxicity | 2009 | Bioorganic & Medicinal Chemistry Letters | 19 | 5012 to 5015 | USA |
| Xue et al. | Xue | Relative importance of proteinase-activated receptor-1 versus matrix metalloproteinases in intracerebral hemorrhage-mediated neurotoxicity in mice | 2009 | Stroke | 40 | 2199 to 2204 | Canada |
| Yoon et al. | Yoon | Sanjoinine A isolated from Semen Zizyphi Spinosi protects against kainic acid-induced convulsions | 2009 | Archives of Pharmacal Research | 32 | 1515 to 1523 | South Korea |
| Young et al. | Young | Deep brain stimulation of the posterior hypothalamic nucleus reverses akinesia in bilaterally 6-hydroxydopamine-lesioned rat | 2009 | Neuroscience | 162 | 1 to 4 | Canada |
| Yu et al. | Yu | Effect of baclofen on neuronal activity in the medial vestibular nucleus after unilateral surgical labyrinthectomy in rats | 2009 | Acta Oto-Laryngologica | 129 | 735 to 740 | China |
| Yurek et al. | Yurek | Compacted DNA nanoparticle gene transfer of GDNF to the rat striatum enhances the survival of grafted fetal dopamine neurons | 2009 | Cell Transplantation | 18 | 1183 to 1196 | USA |
| Zeng et al. | Zeng | The mammalian target of rapamycin signaling pathway mediates epileptogenesis in a model of temporal lobe epilepsy | 2009 | The Journal of Neuroscience | 29 | 6964 to 6972 | USA |
|  |  |  |  |  |  |  |  |
|  |  |  |  |  |  |  |  |

| study ID | experimental groups formed?  If so minimum number of animals per group? (n.a. OR number OR n.r.) | if experimental groups formed:  maximum numbers of animals per group?  (number OR n.a. OR n.r.) | n total of animals used (number OR n.r.) | background/ purpose of craniotomy / field of research (is the study related to on one or more specific neurological diseases or fundamental research?  E.g. Epilepsia, Parkinson’s disease, stroke, brain tumors, traumatic brain injury, migraine)  (name of neurological disease OR fundamental research OR n.r.) | species? (mice / rats OR both) | sex reported?  (female OR male OR both OR n.r.) | commercial breeder reported? If so, specify: (name of breeder OR n.r.) | strain?  (name OR n.r.) | age at surgery reported?  (weeks OR adult OR young OR n.r.) | body weight at surgery reported?  (x-x OR n.r. OR n.a.) | housing condition (pre surgery) reported, if so what?  (housed individually OR housed in groups OR n.r.) | information about cage reported? If so, specify (description OR n.r.) | housing temperature reported, if so which temperature (°C)?  (x-x OR n.r.) | housing humidity reported, if so how high (%)?  (x-x OR n.r.) | enrichment reported? If so, specify (enrichment OR n.r.) | Light schedule reported? If so, specify  (synchronized OR reversed OR n.r. OR other) |
| --- | --- | --- | --- | --- | --- | --- | --- | --- | --- | --- | --- | --- | --- | --- | --- | --- |
| Bartolomucci et al. | 9 | 12 | 31 | fundamental research | mice | male | Charles River Calco Lecco Italy | Swiss CD1 | n.r. | n.r. | housed in groups | n.r. | 20 to 22 | n.r. | n.r. | synchronized |
| Behrend et al. | n.r. | n.r. | 14 | Parkinson’s disease | rats | n.r. | n.r. | Sprague-Dawley | n.r. | n.r. | n.r. | n.r. | 21 | n.r. | n.r. | synchronized |
| Biella et al. | n.a. | n.a. | 9 | fundamental research | rats | n.r. | Charles River Calco Lecco Italy | Sprague-Dawley | n.r. | n.r. | n.r. | n.r. | n.r. | n.r. | n.r. | other |
| Boni et al. | n.r. | n.r. | 73 | fundamental research | rats | male | n.r. | Sprague-Dawley | n.r. | n.r. | n.r. | n.r. | n.r. | n.r. | n.r. | n.r. |
| Bramlett et al. | 2 | 7 | n.r. | fundamental research | rats | both | n.r. | Sprague-Dawley | n.r. | n.r. | n.r. | n.r. | n.r. | n.r. | n.r. | synchronized |
| Byun et al. | n.r. | n.r. | n.r. | fundamental research | mice | male | Folas-International Ltd. Seoul Korea Jackson Laboratory Bar Harbour | ICR, inducible NOS knockout | n.r. | n.r. | housed in groups | n.r. | 20 to 24 | n.r. | n.r. | synchronized |
| Caltana et al. | n.r. | n.r. | 36 | brain stroke | rats | male | Animal Facility of the Pharmacy and Biochemistry School of Buenos Aires | Wistar | adult | n.r. | n.r. | n.r. | n.r. | n.r. | n.r. | synchronized |
| Carcak et al. | n.r. | n.r. | 28 | epilepsy and seizures | rats | male | n.r. | Wistar nonepileptic, GAERS | n.r. | n.r. | n.r. | n.r. | n.r. | n.r. | n.r. | synchronized |
| Cemil et al. | 10 | n.a. | 20 | fundamental research | rats | female | n.r. | Wistar | n.r. | n.r. | n.r. | n.r. | n.r. | n.r. | n.r. | n.r. |
| Chen et al. D | n.r. | n.r. | n.r. | fundamental research | rats | male | Shanghai Experimental Animal Center Chinese Academy of Science | Sprague-Dawley | adult | n.r. | n.r. | n.r. | 23 to 25 | n.r. | n.r. | synchronized |
| Cifani et al. | 7 | 18 | 61 | fundamental research | rats | male | Charles River Calco Lecco Italy | Wistar | n.r. | n.r. | housed individually | hanging stainless steel cage | 24 to 26 | 55 to 65 | n.r. | synchronized |
| Cunningham et al. | n.r. | n.r. | n.r. | fundamental research | mice | female | Harlan Bicester UK | C57BL/6 | n.r. | n.r. | housed in groups | n.r. | n.r. | n.r. | n.r. | n.r. |
| Datta et al. | 7 | 7 | 21 | fundamental research | rats | male | Charles River Wilmington MA USA | Wistar | adult | n.r. | housed individually | n.r. | 24 | n.r. | n.r. | synchronized |
| Diesch et al. | 14 | 15 | 43 | fundamental research | rats | both | Massey University | Sprague-Dawley | 1 to 3 | n.r. | n.r. | n.r. | 21-23 | n.r. | n.r. | synchronized |
| Diguet et al. | 6 | 10 | n.r. | Huntington’s disease | rats | male | Charles River | Sprague-Dawley | n.r. | n.r. | n.r. | n.r. | n.r. | n.r. | n.r. | synchronized |
| Ding et al. | 4 | 7 | n.r. | fundamental research | rats | female | Harlan Indianapolis USA | Wistar | adult | n.r. | housed in groups | n.r. | n.r. | n.r. | n.r. | synchronized |
| Doan et al. | n.a. | n.a. | 6 | fundamental research | rats | male | n.r. | Wistar | adult | n.r. | n.r. | n.r. | n.r. | n.r. | n.r. | n.r. |
| Doretto et al. | 4 | 8 | 75 | epilepsy and seizures | rats | both | Physiology Department of the Ribeirao Preto School of Medicine University of Sao Paulo | Wistar audiogenic rats (WAR), nonepileptic (resistant) Wistar rats | n.r. | n.r. | housed in groups | n.r. | 24 | n.r. | n.r. | synchronized |
| Dux et al. | 5 | 10 | n.r. | fundamental research | rats | male | n.r. | Wistar | adult | n.r. | n.r. | n.r. | n.r. | n.r. | n.r. | n.r. |
| Echegoyen et al. | 6 | 17 | 45 | epilepsy and seizures | rats | n.r. | Charles River Wilmington MA USA | Wistar | n.r. | n.r. | n.r. | n.r. | n.r. | n.r. | n.r. | n.r. |
| Ehrlichman et al. | 8 | 12 | 52 | schizophrenia | mice | male | Harlan Indianapolis USA | C57BL6/Hsd | n.r. | n.r. | housed in groups | n.r. | n.r. | n.r. | n.r. | n.r. |
| Etholm et al. | 7 | 8 | 19 | epilepsy and seizures | mice | both | n.r. | Syn-DKO | n.r. | n.r. | n.r. | Macrolon Eurostandard Type III | 21 to 25 | 45 to 65 | n.r. | synchronized |
| Farias et al. | 4 | 4 | 36 | fundamental research | rats | male | n.r. | Sprague-Dawley | adult | 250 to 300 | n.r. | n.r. | n.r. | n.r. | n.r. | n.r. |
| Foti et al. | 5 | 6 | 16 | epilepsy and seizures | rats | male | Charles River | Sprague-Dawley | n.r. | n.r. | n.r. | n.r. | n.r. | n.r. | n.r. | synchronized |
| Francois et al. | 5 | 40 | 53 | fundamental research | rats | male | Charles River L'Arbresle France | n.r. | 1 | n.r. | housed in groups | n.r. | 20 to 22 | n.r. | n.r. | synchronized |
| Francois et al. | 8 | 40 | 48 | fundamental research | rats | male | Charles River L'Arbresle France | n.r. | 12 | n.r. | housed in groups | n.r. | 20 to 22 | n.r. | n.r. | synchronized |
| Fritsch et al. | 5 | 25 | n.r. | epilepsy and seizures | rats | male | Taconic Farms Rockville MD USA | Sprague-Dawley | 6 to 7 | n.r. | housed individually | n.r. | 20 to 23 | n.r. | n.r. | synchronized |
| Good et al. | n.r. | n.r. | n.r. | epilepsy and seizures | rats | male | Harlan Labs Madison WE | Sprague-Dawley | n.r. | 350 to 400 | housed individually | n.r. | n.r. | n.r. | n.r. | n.r. |
| Griesbach et al. | n.r. | n.r. | 72 | tbi | rats | male | n.r. | Sprague-Dawley | n.r. | n.r. | n.r. | opaque plastic bin | n.r. | n.r. | n.r. | n.r. |
| Guidine et al. | n.r. | n.r. | 18 | fundamental research | rats | male | CeBIO-ICB-UFMG | Wistar | 3 | n.r. | n.r. | n.r. | 22 | n.r. | n.r. | synchronized |
| Gurevicius et al. | 5 | 9 | 28 | fundamental research | mice | male | n.r. | TNC (+/+), TNC (-/-) | 24 to 36 | n.r. | housed in groups | n.r. | 21 | n.r. | n.r. | synchronized |
| Hart et al. | 8 | 11 | 188 | fundamental research | rats | male | Gore Hill Research Laboratories | Wistar | n.r. | n.r. | housed in groups | opaque plastic bin | n.r. | n.r. | n.r. | synchronized |
| Harvey et al. | n.r. | n.r. | n.r. | fundamental research | mice | n.r. | n.r. | C57BL/6J | n.r. | n.r. | n.r. | n.r. | n.r. | n.r. | n.r. | n.r. |
| Hernandez-Gonzalez et al. | n.r. | n.r. | 20 | fundamental research | rats | male | Institute of Neurosciences University of Guadalajara | Wistar | n.r. | n.r. | housed individually | n.r. | 22 to 23 | n.r. | n.r. | reversed |
| Ho et al. | 7 | 10 | 53 | fundamental research | mice | n.r. | n.r. | EphB2, EphB3, EphB3, combinational lines | adult | n.r. | n.r. | n.r. | n.r. | n.r. | n.r. | synchronized |
| Holtmaat et al. | n.r. | n.r. | n.r. | fundamental research | mice | n.r. | n.r. | n.r. | n.r. | n.r. | n.r. | n.r. | n.r. | n.r. | n.r. | n.r. |
| Hrncic et al. | 7 | 10 | 31 | epilepsy and seizures | rats | male | Military Medical Academy Breeding Laboratory Belgrade Serbia | Wistar albino | adult | n.r. | housed individually | transparent plastic wire-covered cage | 22 to 23 | 50 to 60 | n.r. | synchronized |
| Huguet et al. | n.r. | n.r. | 59 | fundamental research | rats | male | n.r. | Wistar | 13 to 14 | n.r. | housed individually | n.r. | 21 to 23 | 40 to 70 | n.r. | synchronized |
| Ishida et al. | n.r. | n.r. | n.r. | schizophrenia | rats | male | Japan SLC Shizuoka | Wistar | n.r. | n.r. | n.r. | aluminum cage | 22 to 26 | 40 to 70 | n.r. | synchronized |
| Ito et al. | n.r. | n.r. | n.r. | fundamental research | mice | male | SLC Fukuoka Japan | Institute of Cancer Research (ICR) | 10 | n.r. | n.r. | n.r. | n.r. | n.r. | n.r. | n.r. |
| Itoh et al. B | n.a. | n.a. | 18 | tbi | rats | male | n.r. | Wistar | 10 | n.r. | n.r. | n.r. | n.r. | n.r. | n.r. | n.r. |
| Itoh et al. A | n.r. | n.r. | 75 | tbi | rats | male | n.r. | Wistar | 8 to 9 | 200 to 250 | n.r. | n.r. | n.r. | n.r. | n.r. | n.r. |
| Jafri et al. | n.r. | n.r. | n.r. | fundamental research | rats | n.r. | Charles River | Sprague-Dawley | adult | 250 to 350 | n.r. | n.r. | n.r. | n.r. | n.r. | n.r. |
| Kalauzi et al. | n.a. | n.a. | 14 | fundamental research | rats | male | n.r. | Sprague-Dawley | adult | n.r. | n.r. | n.r. | 25 | n.r. | n.r. | synchronized |
| Katz et al. | 9 | 9 | 18 | fundamental research | rats | n.r. | n.r. | Sprague-Dawley | n.r. | n.r. | n.r. | n.r. | n.r. | n.r. | n.r. | n.r. |
| Kim et al. A | 6 | 12 | 18 | epilepsy and seizures | rats | male | n.r. | Wistar | n.r. | 200 | n.r. | n.r. | n.r. | n.r. | n.r. | n.r. |
| Lackovic et al. | 5 | 11 | 55 | fundamental research | rats | male | Zagreb University School of Medicine Zagreb Croatia | Wistar | 12 | 250 to 300 | housed in groups | transparent plastic cage | 23 | n.r. | n.r. | n.r. |
| Lee et al. B | n.r. | n.r. | n.r. | fundamental research | mice | n.r. | n.r. | B6-129 | 10 | n.r. | n.r. | n.r. | n.r. | n.r. | n.r. | synchronized |
| Lee et al. A | 5 | 5 | 15 | tbi | rats | male | Taconic Farms Inc. Germantown NY USA | Sprague-Dawley | young | 270 to 330 | n.r. | n.r. | n.r. | n.r. | n.r. | n.r. |
| Li et al. C | 5 | 5 | 50 | tbi | rats | male | Experimental Animal Department of China Medical University | Sprague-Dawley | adult | 200 to 250 | n.r. | n.r. | n.r. | n.r. | n.r. | n.r. |
| Liu et al. | 5 | 6 | 35 | epilepsy and seizures | rats | male | n.r. | Wistar | 7 | 180 to 200 | n.r. | n.r. | n.r. | n.r. | n.r. | n.r. |
| Lopez-Martin et al. | n.r. | n.r. | n.r. | epilepsy and seizures | rats | male | n.r. | Sprague-Dawley | adult | n.r. | housed individually | n.r. | 22 | n.r. | n.r. | n.r. |
| Lu et al. | 9 | 14 | 69 | epilepsy and seizures | rats | male | Charles River Raleigh NC USA | Sprague-Dawley | n.r. | n.r. | housed individually | n.r. | n.r. | n.r. | n.r. | synchronized |
| Lundblad et al. | n.r. | n.r. | 101 | tbi | mice | male | Jackson Laboratory Bar Harbour | eNOS-KO, C57BL/6 wildtype | adult | n.r. | n.r. | n.r. | n.r. | n.r. | n.r. | n.r. |
| Magloire et al. | 6 | 6 | 12 | fundamental research | rats | male | n.r. | Wistar | n.r. | n.r. | housed individually | plexiglass cylinder | 22 to 23 | n.r. | n.r. | synchronized |
| Mark et al. | 4 | 7 | n.r. | fundamental research | rats | male | n.r. | Sprague-Dawley, Harlan Sprague-Dawley | n.r. | n.r. | n.r. | n.r. | n.r. | n.r. | n.r. | n.r. |
| McCracken et al. | 11 | 11 | 33 | fundamental research | rats | male | n.r. | Sprague-Dawley | n.r. | 275 to 400 | n.r. | n.r. | n.r. | n.r. | n.r. | n.r. |
| Meeren et al. | n.r. | n.r. | 13 | epilepsy and seizures | rats | male | n.r. | WAG/Rjj | 10 to 20 | 270 to 450 | housed in groups | n.r. | n.r. | n.r. | n.r. | n.r. |
| Merkler et al. | n.r. | n.r. | 64 | fundamental research | mice | n.r. | n.r. | C57BL/6 | adult | n.r. | housed in groups | n.r. | n.r. | n.r. | n.r. | n.r. |
| Merkler et al. | 5 | 27 | 32 | fundamental research | rats | female | Harlan Host Netherlands | Lewis | adult | n.r. | housed in groups | n.r. | n.r. | n.r. | n.r. | n.r. |
| Mian et al. | 1 | n.a. | 6 | fundamental research | rats | female | n.r. | Sprague Dawley | 12 | n.r. | n.r. | n.r. | n.r. | n.r. | n.r. | n.r. |
| Mohammadi et al. | 4 | 6 | 80 | fundamental research | rats | female | n.r. | Sprague -Dawley | n.r. | n.r. | n.r. | n.r. | n.r. | n.r. | n.r. | n.r. |
| Mollazaedh et al. | n.r. | n.r. | n.r. | fundamental research | rats | male | n.r. | Sprague-Dawley | n.r. | n.r. | n.r. | n.r. | n.r. | n.r. | n.r. | n.r. |
| Mukherjee et al. | 16 | 20 | 45 | fundamental research | rats | male | n.r. | Sprague-Dawley | n.r. | n.r. | housed individually | n.r. | 20 to 24 | n.r. | n.r. | n.r. |
| Nehlig et al. | n.a. | n.a. | 8 | epilepsy and seizures | rats | male | n.r. | GAERS | adult | n.r. | n.r. | n.r. | 22 | n.r. | n.r. | synchronized |
| Nuki et al. | n.r. | n.r. | 84 | fundamental research | mice | n.r. | Jackson Laboratory | C57BL/6J | 8 to 10 | n.r. | n.r. | n.r. | n.r. | n.r. | n.r. | n.r. |
| Onyszchuk et al. | 3 | 8 | 106 | tbi | mice | male | National Institute on Aging | C57BL/6 | 84 to 96 | n.r. | n.r. | n.r. | n.r. | n.r. | n.r. | n.r. |
| Oshima et al. | 5 | 7 | 26 | tbi | mice | male | n.r. | TNF-alpha-/-, C57BL/6 | 6 to 9 | 25 to 35 | n.r. | n.r. | n.r. | n.r. | n.r. | n.r. |
| Potts et al. | 3 | 7 | 59 | tbi | mice | male | n.r. | GPxTg, wildtype | 3 | n.r. | n.r. | n.r. | n.r. | n.r. | n.r. | n.r. |
| Qing et al. | 12 | 84 | 144 | fundamental research | rats | both | Animal Experimental Center of Central-South University | Sprague-Dawley | n.r. | n.r. | n.r. | n.r. | n.r. | n.r. | n.r. | n.r. |
| Rahim et al. | 4 | 5 | 29 | fundamental research | rats | n.r. | n.r. | Sprague-Dawley | adult | n.r. | n.r. | n.r. | n.r. | n.r. | n.r. | n.r. |
| Rimoli et al. | 8 | 10 | n.r. | epilepsy and seizures | rats | male | Harlan Italy Correzzana Milan | WAG/Rjj | 24 to 28 | n.r. | n.r. | n.r. | 19 to 23 | n.r. | n.r. | reversed |
| Roiko et al. | n.r. | n.r. | n.r. | fundamental research | rats | male | Harlan Indianapolis USA | Holtzman Sprague-Dawley | n.r. | 300 to 325 | housed individually | n.r. | n.r. | n.r. | n.r. | reversed |
| Rudnick et al. | 10 | 10 | 20 | fundamental research | mice | male | n.r. | C57BL/6 wildtype, ß2-KO | n.r. | n.r. | n.r. | n.r. | n.r. | n.r. | n.r. | n.r. |
| Sahin et al. | 7 | 7 | 21 | epilepsy and seizures | rats | male | n.r. | Wistar | n.r. | n.r. | housed in groups | n.r. | n.r. | n.r. | n.r. | n.r. |
| Samnick et al. | 12 | 12 | 144 | brain tumors | rats | male | Charles River Laboratories | Fischer 344, Rowett nude rat | n.r. | n.r. | n.r. | n.r. | n.r. | n.r. | n.r. | n.r. |
| Sasaki et al. | 4 | 7 | 18 | brain stroke | rats | male | Charles River Japan | spontaneously hypertensive rats | n.r. | 280 to 360 | n.r. | n.r. | n.r. | n.r. | n.r. | n.r. |
| Schei et al. | n.r. | n.r. | 4 | fundamental research | rats | female | Simonsen Laboratories Gilroy USA | Sprague-Dawley | n.r. | n.r. | n.r. | n.r. | n.r. | n.r. | n.r. | n.r. |
| Schmid et al. | 7 | 8 | 15 | Alzheimer’s disease | rats | male | Biomedical Facility University College Dublin Ireland | Wistar | n.r. | 170 to 200 | n.r. | n.r. | n.r. | n.r. | n.r. | n.r. |
| Sekiya et al. | n.a. | n.a. | 4 | fundamental research | rats | male | n.r. | Sprague-Dawley | adult | n.r. | n.r. | n.r. | n.r. | n.r. | n.r. | n.r. |
| Sher et al. | 6 | 6 | 12 | fundamental research | mice | n.r. | n.r. | FVB, FVB luciferase-GFP-actin transgenic mice | n.r. | n.r. | n.r. | n.r. | n.r. | n.r. | n.r. | n.r. |
| Shultz et al. | 9 | 11 | 50 | fundamental research | rats | male | Charles River Laboratories Quebec Canada | Long-Evans | adult | n.r. | housed in groups | acrylic cage | 20 to 22 | n.r. | n.r. | synchronized |
| Silvani et al. | n.r. | n.r. | n.r. | fundamental research | mice | male | Harlan Italy Udine | B6.V-Lepob/ob/OlyHsd, B6.V-Lep+/+/OlyHsd | n.r. | n.r. | n.r. | n.r. | 25 | n.r. | n.r. | n.r. |
| Sinton et al. | n.r. | n.r. | 5 | fundamental research | mice | male | Jackson Laboratory Bar Harbour | C57BL/6 | n.r. | n.r. | n.r. | n.r. | n.r. | n.r. | n.r. | n.r. |
| Song et al. | n.r. | n.r. | 12 | fundamental research | rats | male | Charles River Laboratories | Sprague-Dawley | adult | n.r. | n.r. | n.r. | n.r. | n.r. | n.r. | n.r. |
| Takahashi et al. | 3 | 4 | 10 | tbi | rats | n.r. | Japan SLC Hamamatsu Japan | Wistar | n.r. | n.r. | n.r. | n.r. | n.r. | n.r. | n.r. | n.r. |
| Takahashi et al. | 3 | 4 | 10 | tbi | rats | n.r. | Japan SLC Hamamatsu Japan | Wistar | n.r. | n.r. | n.r. | n.r. | n.r. | n.r. | n.r. | n.r. |
| Tanida et al. | n.r. | n.r. | 44 | fundamental research | rats | male | n.r. | Wistar | n.r. | n.r. | n.r. | n.r. | 23 to 25 | n.r. | n.r. | synchronized |
| Tchekalarova et al. | 5 | 9 | 47 | epilepsy and seizures | rats | male | Institute of Neurobiology Bulgarian Academy of Sciences | Wistar | n.r. | n.r. | housed in groups | n.r. | 17 to 23 | 40 to 50 | n.r. | synchronized |
| Thomas et al. | 5 | 5 | 15 | fundamental research | rats | n.r. | n.r. | Sprague-Dawley | n.r. | n.r. | n.r. | n.r. | n.r. | n.r. | n.r. | n.r. |
| Topchiy et al. | n.r. | n.r. | 24 | fundamental research | rats | female | n.r. | Sprague-Dawley | adult | n.r. | n.r. | n.r. | n.r. | n.r. | n.r. | n.r. |
| Touzani et al. | 15 | 28 | 91 | fundamental research | rats | male | Charles River Wilmington MA | Sprague-Dawley | adult | 408 to 522 | housed individually | n.r. | 21 | n.r. | n.r. | synchronized |
| Tsanov et al. | 6 | n.a. | 36 | fundamental research | rats | male | n.r. | Long-Evans | 7 to 8 | n.r. | n.r. | n.r. | n.r. | n.r. | n.r. | n.r. |
| Wagner et al. | n.r. | n.r. | 68 | tbi | rats | male | Hilltop Laboratories Scottsdale USA | Sprague-Dawley | adult | 275 to 300 | n.r. | n.r. | n.r. | n.r. | n.r. | n.r. |
| Wan et al. | n.r. | n.r. | 10 | fundamental research | rats | male | n.r. | Sprague-Dawley | n.r. | n.r. | n.r. | n.r. | n.r. | n.r. | n.r. | n.r. |
| Wigren et al. | 11 | 17 | 41 | fundamental research | rats | male | n.r. | Hannover Wistar | n.r. | n.r. | n.r. | n.r. | 20 to 22 | n.r. | n.r. | synchronized |
| Worthen et al. | 8 | 8 | 48 | epilepsy and seizures | rats | male | n.r. | albino Sprague-Dawley | adult | 275 to 300 | n.r. | n.r. | n.r. | n.r. | n.r. | n.r. |
| Xue et al. | 5 | 5 | 30 | fundamental research | mice | both | n.r. | C57BL/6, PAR1 | adult | n.r. | n.r. | n.r. | n.r. | n.r. | n.r. | n.r. |
| Yoon et al. | n.r. | n.r. | n.r. | epilepsy and seizures | rats | male | Samtako | Sprague-Dawley | n.r. | n.r. | n.r. | acrylic cage | 20 to 24 | n.r. | n.r. | synchronized |
| Young et al. | n.r. | n.r. | 15 | fundamental research | rats | male | Animal Care Facility University of Canada | Long-Evans | n.r. | n.r. | housed individually | n.r. | n.r. | n.r. | n.r. | n.r. |
| Yu et al. | 6 | 9 | 23 | fundamental research | rats | n.r. | n.r. | Sprague-Dawley | n.r. | n.r. | n.r. | n.r. | n.r. | n.r. | n.r. | n.r. |
| Yurek et al. | 5 | 9 | 26 | fundamental research | rats | male | Harlan Farms | Sprague-Dawley | n.r. | n.r. | n.r. | n.r. | n.r. | n.r. | n.r. | n.r. |
| Zeng et al. | 6 | 8 | n.r. | epilepsy and seizures | rats | male | Charles River Laboratories | Sprague-Dawley | n.r. | n.r. | n.r. | n.r. | n.r. | n.r. | n.r. | n.r. |

| study ID | handling technique pre surgery reported? If so, specify (handling technique OR n.r.) | duration of surgery reported?  (x minutes OR n.r.) | survival surgery?  (did animals wake up from anesthesia?)  (yes / no OR n.r.) | how long did they live after surgery approximately?  (<1 day, <1 week, <2 weeks, <4 weeks, <8 weeks, >8 weeks OR n.a. OR n.r.) | mortality during surgery reported?  (n.r. OR number) | fate of the used animals? (killed OR n.r. OR reused) | type of surgical procedure  (name) | if "other", specify:  (name OR n.a.) | if applicable:  implantation site (name OR n.a. OR n.r.) | insult size reported? (mm diameter OR n.r.) | trepanning size reported? (mm diameter OR n.r.) | no permanent implant? If permanent implant, number of anchoring screws reported?  (nonpermanent implant OR number of anchoring screws OR n.r.) | model used? if so, what kind of model?  e.g. TBI, CCI, fluid percussion injury  (model OR n.a.) |
| --- | --- | --- | --- | --- | --- | --- | --- | --- | --- | --- | --- | --- | --- |
| Bartolomucci et al. | n.r. | n.r. | yes | <2 weeks | n.r. | killed | intracerebroventricular guide cannula implantation | n.a. | n.r. | n.r. | n.r. | n.r. | n.a. |
| Behrend et al. | n.r. | n.r. | no | n.r. | n.r. | killed | deep stimulating electrode implantation | n.a. | subthalmic nucleus | n.r. | n.r. | n.r. | n.a. |
| Biella et al. | n.r. | n.r. | n.r. | n.r. | n.r. | n.r. | deep electrode implantation | n.a. | cortex | n.r. | 1 | n.r. | n.a. |
| Boni et al. | n.r. | n.r. | n.r. | n.r. | n.r. | n.r. | other | thinned cranial window | parietal bone | n.r. | n.r. | nonpermanent implant | n.a. |
| Bramlett et al. | n.r. | n.r. | yes | n.r. | n.r. | killed | TBI, fluid percussion | n.a. | parietal cortex | 4,8 | 4,8 | n.r. | n.a. |
| Byun et al. | n.r. | n.r. | yes | <1 day | n.r. | killed | intracerebral injection cannula | n.a. | n.r. | n.r. | n.r. | nonpermanent implant | n.a. |
| Caltana et al. | n.r. | n.r. | yes | <1 week | n.r. | killed | intracerebral injection cannula | n.a. | cortex | n.r. | n.r. | nonpermanent implant | n.a. |
| Carcak et al. | n.r. | n.r. | yes | <2 weeks | n.r. | killed | deep electrode implantation | n.a. | amygdala, cortex | n.r. | n.r. | n.r. | n.a. |
| Cemil et al. | n.r. | n.r. | yes | <8 weeks | n.r. | killed | sole craniotomy | n.a. | frontoparietal cortex | n.r. | 7-8 | nonpermanent implant | n.a. |
| Chen et al. D | n.r. | n.r. | yes | <8 weeks | n.r. | killed | other | EEG electrode implantation, intracerebroventricular injection cannula | n.r. | n.r. | n.r. | nonpermanent implant | n.a. |
| Cifani et al. | n.r. | n.r. | yes | <4 weeks | n.r. | n.r. | intracerebroventricular guide cannula implantation | n.a. | third cerebral ventricle | n.r. | n.r. | n.r. | n.a. |
| Cunningham et al. | n.r. | n.r. | yes | >8weeks | n.r. | killed | other | intracerebroventricular injection OR intracerebral injection | dorsal hippocampus | n.r. | n.r. | nonpermanent implant | n.a. |
| Datta et al. | n.r. | n.r. | yes | <2 weeks | n.r. | killed | surface (screw) and deep electrode implantation | n.a. | cortex, hippocampus | n.r. | n.r. | n.r. | n.a. |
| Diesch et al. | n.r. | n.r. | no | n.a. | n.r. | killed | electrode implantation | n.a. | n.r. | n.r. | n.r. | nonpermanent implant | n.a. |
| Diguet et al. | n.r. | n.r. | yes | >8weeks | n.r. | killed | intracerebral injection cannula | n.a. | striatum | n.r. | n.r. | nonpermanent implant | n.a. |
| Ding et al. | n.r. | n.r. | yes | <4 weeks | n.r. | killed | intracerebroventricular guide cannula implantation | n.a. | n.r. | n.r. | n.r. | n.r. | n.a. |
| Doan et al. | n.r. | n.r. | yes | <1 week | n.r. | killed | intracerebral guide cannula implantation | n.a. | cortex, hippocampus | n.r. | n.r. | n.r. | n.a. |
| Doretto et al. | n.r. | n.r. | yes | <4 weeks | n.r. | killed | other | section of superior colliculus, ablation of superior colliculus | superior colliculus | n.r. | n.r. | nonpermanent implant | n.a. |
| Dux et al. | n.r. | n.r. | yes | n.r. | n.r. | killed | sole craniotomy | n.a. | n.r. | n.r. | 4x6 | n.a. | n.a. |
| Echegoyen et al. | n.r. | n.r. | yes | n.r. | n.r. | n.r. | deep electrode implantation | n.a. | hippocampus | n.r. | n.r. | n.r. | n.a. |
| Ehrlichman et al. | n.r. | n.r. | yes | n.r. | n.r. | n.r. | deep electrode implantation | n.a. | hippocampus | n.r. | n.r. | n.r. | n.a. |
| Etholm et al. | n.r. | n.r. | yes | n.r. | n.r. | n.r. | deep electrode implantation | n.a. | thalamus | n.r. | n.r. | n.r. | n.a. |
| Farias et al. | n.r. | n.r. | yes | <1 day | n.r. | killed | other | Luer hub implantation | n.r. | n.r. | 3 | 2 | fluid percussion |
| Foti et al. | n.r. | n.r. | yes | <2 weeks | n.r. | killed | intracerebral injection cannula | n.a. | piriform cortex | n.r. | 0,2 | nonpermanent implant | n.a. |
| Francois et al. | n.r. | n.r. | yes | >8weeks | n.r. | killed | intracerebral injection cannula | n.a. | ventral hippocampus | n.r. | n.r. | nonpermanent implant | n.a. |
| Francois et al. | n.r. | n.r. | yes | >8weeks | n.r. | killed | electrode implantation | n.a. | cortex | n.r. | n.r. | n.r. | n.a. |
| Fritsch et al. | n.r. | n.r. | yes | <4 weeks | n.r. | killed | surface (screw) and deep electrode implantation | n.a. | n.r. | n.r. | n.r. | 5 | n.a. |
| Good et al. | n.r. | n.r. | yes | n.r. | n.r. | killed | deep electrode implantation | n.a. | cortex, hippocampus, centromedial thalamic nucleus | n.r. | 1 | n.r. | n.a. |
| Griesbach et al. | n.r. | n.r. | yes | n.r. | n.r. | n.r. | other | lateral fluid percussion injury, intracerebral microbead injection | left parietal cortex, left dorsal hippocampus | n.r. | 3 | nonpermanent implant | fluid percussion |
| Guidine et al. | n.r. | n.r. | yes | <1 week | n.r. | killed | surface (screw) and deep electrode implantation | n.a. | left parietal cortex, nucleus of the solitary tract | n.r. | n.r. | n.r. | n.a. |
| Gurevicius et al. | n.r. | n.r. | yes | <4 weeks | n.r. | killed | surface (screw) and deep electrode implantation | n.a. | hippocampus, cortical screw | 0,5 | n.r. | n.r. | n.a. |
| Hart et al. | handled daily for 3 days after surgery | n.r. | yes | <1 week | n.r. | killed | intracerebral guide cannula implantation | n.a. | basolateral amygdala | 0,405 | n.r. | 6 | n.a. |
| Harvey et al. | n.r. | n.r. | yes | n.r. | n.r. | n.r. | deep electrode implantation | n.a. | dorsal hippocampus | n.r. | 0,5 | n.r. | n.a. |
| Hernandez-Gonzalez et al. | n.r. | n.r. | yes | <4 weeks | n.r. | killed | deep electrode implantation | n.a. | Acc shell, VTA | n.r. | n.r. | n.r. | n.a. |
| Ho et al. | n.r. | n.r. | yes | <1 week | n.r. | killed | intracerebral injection cannula | n.a. | temporal cortex, olfactory bulb | n.r. | 0,5 | nonpermanent implant | n.a. |
| Holtmaat et al. | n.r. | n.r. | yes | n.r. | n.r. | killed | other | chronic cranial window | n.r. | n.r. | 3 to 7 | nonpermanent implant | n.a. |
| Hrncic et al. | handled daily for 7 days | n.r. | yes | n.r. | n.r. | n.r. | deep recording electrode implantation | n.a. | cortex | n.r. | n.r. | n.r. | n.a. |
| Huguet et al. | n.r. | n.r. | yes | n.r. | n.r. | killed | deep electrode implantation | n.a. | hypothalamus | n.r. | n.r. | n.r. | n.a. |
| Ishida et al. | n.r. | n.r. | yes | <4 weeks | n.r. | n.r. | surface (screw) and deep electrode implantation | n.a. | left frontal cortex, left frontal bone | 0,8 | n.r. | 2 | n.a. |
| Ito et al. | n.r. | n.r. | yes | <8 weeks | n.r. | n.r. | intracerebroventricular guide cannula implantation | n.a. | n.r. | n.r. | n.r. | n.r. | n.a. |
| Itoh et al. B | n.r. | n.r. | yes | <1 week | n.r. | killed | TBI | n.a. | n.r. | n.r. | n.r. | nonpermanent implant | n.a. |
| Itoh et al. A | n.r. | n.r. | yes | >8weeks | n.r. | killed | TBI | n.a. | n.r. | 1 | 2 to 2,5 | nonpermanent implant | n.a. |
| Jafri et al. | n.r. | n.r. | no | n.a. | n.r. | killed | other | intracerebral injection cannula, hippocampal lesioning | hippocampus, cortex, corpus callosum, thalamus, substantia nigra | 0,5 | n.r. | nonpermanent implant | n.a. |
| Kalauzi et al. | n.r. | n.r. | yes | n.r. | n.r. | killed | deep electrode implantation | n.a. | cortex, right pedunculopontine tegmental nucleus | n.r. | n.r. | n.r. | n.a. |
| Katz et al. | n.r. | n.r. | yes | n.r. | n.r. | n.r. | other | brain telemetric temperature probe implantation | n.r. | n.r. | n.r. | n.r. | n.a. |
| Kim et al. A | n.r. | n.r. | yes | <2 weeks | n.r. | killed | intracerebroventricular injection cannula | n.a. | right lateral brain ventricle | n.r. | n.r. | nonpermanent implant | n.a. |
| Lackovic et al. | n.r. | n.r. | yes | >8weeks | n.r. | n.r. | intracerebroventricular injection cannula | n.a. | left and right lateral ventricle | n.r. | n.r. | nonpermanent implant | n.a. |
| Lee et al. B | n.r. | n.r. | yes | n.r. | n.r. | killed | surface (screw) electrode implantation | n.a. | n.r. | n.r. | n.r. | n.r. | n.a. |
| Lee et al. A | n.r. | n.r. | yes | <2 weeks | n.r. | killed | other | fluid percussion brain injury, intracerebroventricular guide cannula implantation | n.r. | n.r. | 4,8 | n.r. | fluid percussion |
| Li et al. C | n.r. | n.r. | yes | <2 weeks | n.r. | killed | TBI, cci | n.a. | n.r. | n.r. | n.r. | nonpermanent implant | cci |
| Liu et al. | n.r. | n.r. | yes | <8 weeks | n.r. | killed | deep electrode implantation | n.a. | left basolateral amygdaloid nucleus | n.r. | n.r. | 4 | n.a. |
| Lopez-Martin et al. | n.r. | n.r. | yes | <2 weeks | n.r. | killed | surface (screw) electrode implantation | n.a. | frontal cortex, parietal cortex | n.r. | n.r. | n.r. | n.a. |
| Lu et al. | n.r. | n.r. | yes | <1 week | n.r. | killed | surface (screw) electrode implantation | n.a. | parietal cortex | n.r. | n.r. | n.r. | n.a. |
| Lundblad et al. | n.r. | n.r. | yes | n.r. | n.r. | killed | tbi, cci | n.a. | n.r. | n.r. | 5 | nonpermanent implant | cci |
| Magloire et al. | handled daily | n.r. | yes | n.r. | n.r. | n.r. | surface (screw) electrode implantation | n.a. | n.a. | 1 | n.r. | 4 | n.a. |
| Mark et al. | n.r. | n.r. | n.r. | n.r. | n.r. | killed | intracerebral injection cannula | n.a. | nucleus tractus soletarii | n.r. | n.r. | nonpermanent implant | n.a. |
| McCracken et al. | n.r. | n.r. | n.r. | n.r. | n.r. | n.r. | deep electrode implantation | n.a. | nucleus accumbens, orbitofrontal cortex, mediodorsal thalamus, medial prefrontal cortex | n.r. | n.r. | n.r. | n.a. |
| Meeren et al. | n.r. | n.r. | yes | <4 weeks | n.r. | killed | other | deep electrode implantation, intracerebral guide cannula implantation | frontal cortex, cerebellum, ventroposterior medial nucleus | n.r. | n.r. | 4 | n.a. |
| Merkler et al. | n.r. | n.r. | no | n.a. | n.r. | killed | deep electrode implantation | cortical spreading depression recording | n.r. | n.r. | 2 | nonpermanent implant | n.a. |
| Merkler et al. | n.r. | n.r. | yes | <2 weeks | n.r. | killed | intracerebral injection cannula | n.a. | cortex | n.r. | n.r. | nonpermanent implant | n.a. |
| Mian et al. | n.r. | n.r. | yes | n.r. | n.r. | n.r. | surface (screw) electrode implantation | n.a. | n.r. | 5 | n.r. | n.r. | n.a. |
| Mohammadi et al. | n.r. | n.r. | no | n.a. | n.r. | killed | intracerebroventricular injection cannula | n.a. | n.r. | 0,255 | 0,255 | nonpermanent implant | n.a. |
| Mollazaedh et al. | n.r. | n.r. | n.r. | n.r. | n.r. | n.r. | electrode implantation | n.a. | somatosensory cortex | n.r. | n.r. | n.r. | n.a. |
| Mukherjee et al. | n.r. | n.r. | yes | <8 weeks | n.r. | n.r. | deep electrode implantation | n.a. | frontal cortex, parietal cortex, occipital cortex | n.r. | n.r. | n.r. | n.a. |
| Nehlig et al. | n.r. | n.r. | yes | <4 weeks | n.r. | n.r. | deep electrode implantation | n.a. | frontoparietal cortex | n.r. | n.r. | n.r. | n.a. |
| Nuki et al. | n.r. | 30 | yes | <2 weeks | n.r. | killed | intracerebroventricular injection cannula | n.a. | right basal cistern | n.r. | n.r. | nonpermanent implant | n.a. |
| Onyszchuk et al. | n.r. | n.r. | yes | >8weeks | n.r. | killed | tbi, cci | n.a. | sensorimotor cortex | 3 | 3,5 | nonpermanent implant | cci |
| Oshima et al. | n.r. | n.r. | yes | <4 weeks | n.r. | killed | other | tbi controlled cortical impact, intracerebral injection | n.r. | 3 | 5 | nonpermanent implant | cci |
| Potts et al. | n.r. | n.r. | yes | <4 weeks | n.r. | killed | tbi, cci | n.a. | parietal cortex | 3 | 4 | nonpermanent implant | cci |
| Qing et al. | n.r. | n.r. | yes | <2 weeks | n.r. | killed | intracerebral injection cannula | n.a. | right caudate | n.r. | 1 | nonpermanent implant | n.a. |
| Rahim et al. | n.r. | n.r. | yes | <2 weeks | n.r. | killed | intracerebral injection cannula | n.a. | striatum, motor cortex, red nucleus | n.r. | n.r. | nonpermanent implant | n.a. |
| Rimoli et al. | n.r. | n.r. | yes | n.r. | n.r. | n.r. | surface (screw) electrode implantation | n.a. | over frontal cortex parietal cortex cerebellum | n.r. | n.r. | n.r. | n.a. |
| Roiko et al. | n.r. | n.r. | yes | n.r. | n.r. | n.r. | deep electrode implantation | n.a. | lateral hypothalamus | n.r. | n.r. | n.r. | n.a. |
| Rudnick et al. | n.r. | n.r. | yes | n.r. | n.r. | n.r. | deep recording electrode implantation | n.a. | CA3 | n.r. | n.r. | n.r. | n.a. |
| Sahin et al. | n.r. | n.r. | yes | n.r. | n.r. | n.r. | deep electrode implantation | n.a. | n.r. | n.r. | n.r. | 8 | n.a. |
| Samnick et al. | n.r. | n.r. | yes | n.r. | n.r. | killed | intracerebral injection cannula | n.a. | n.r. | n.r. | n.r. | nonpermanent implant | n.a. |
| Sasaki et al. | n.r. | n.r. | yes | <1 day | n.r. | killed | electrode implantation | n.a. | anterior and posterior cortex | n.r. | 9x11 | n.r. | n.a. |
| Schei et al. | n.r. | n.r. | yes | n.r. | n.r. | n.r. | other | screw electrode implantation, photodiode implantation, LED implantation | n.r. | n.r. | 0,9 to 1,4 | n.r. | n.a. |
| Schmid et al. | n.r. | n.r. | n.r. | n.r. | n.r. | n.r. | other | intracerebroventricular guide cannula implantation, deep reporting electrode implantation | hippocampus, cortex, lateral ventricle | n.r. | n.r. | n.r. | n.a. |
| Sekiya et al. | n.r. | n.r. | yes | <8 weeks | n.r. | killed | other | compression recording electrode implantation | 7th and 8th cranial nerve trunks | n.r. | n.r. | n.r. | n.a. |
| Sher et al. | n.r. | n.r. | yes | <8 weeks | n.r. | killed | intracerebral injection cannula | n.a. | corpus callosum | n.r. | n.r. | nonpermanent implant | n.a. |
| Shultz et al. | n.r. | n.r. | yes | n.r. | n.r. | killed | intracerebroventricular guide cannula implantation | n.a. | right lateral brain ventricle | 0,57 | n.r. | 4 | n.a. |
| Silvani et al. | n.r. | n.r. | yes | n.r. | n.r. | n.r. | surface (screw) electrode implantation | n.a. | n.r. | n.r. | n.r. | n.r. | n.a. |
| Sinton et al. | n.r. | n.r. | yes | n.r. | n.r. | killed | surface (screw) electrode implantation | n.a. | n.r. | n.r. | 0,75 | n.r. | n.a. |
| Song et al. | n.r. | n.r. | yes | n.r. | n.r. | n.r. | intracerebral injection cannula | n.a. | dorsolateral pons | n.r. | 5 | nonpermanent implant | n.a. |
| Takahashi et al. | n.r. | n.r. | yes | >8weeks | n.r. | killed | other | hemidecortication, intracerebral injection cannula | sensorimotor cortex | n.r. | n.r. | nonpermanent implant | n.a. |
| Takahashi et al. | n.r. | n.r. | yes | >8weeks | n.r. | killed | other | hemidecortication, intracerebral injection cannula | sensorimotor cortex | n.r. | n.r. | nonpermanent implant | n.a. |
| Tanida et al. | n.r. | n.r. | yes | n.r. | n.r. | n.r. | intracerebroventricular injection cannula | n.a. | third cerebral ventricle | n.r. | n.r. | nonpermanent implant | n.a. |
| Tchekalarova et al. | n.r. | n.r. | yes | <8 weeks | n.r. | killed | surface (screw) electrode implantation | n.a. | frontal cortex, parietal cortex | n.r. | n.r. | n.r. | n.a. |
| Thomas et al. | n.r. | n.r. | yes | n.r. | n.r. | killed | intracerebral injection cannula | n.a. | superior colliculus | n.r. | n.r. | nonpermanent implant | n.a. |
| Topchiy et al. | handled for 2-3 weeks | n.r. | yes | n.r. | n.r. | n.r. | other | screw electrode implantation, deep electrode implantation | lambda midline, parietal cortex, frontal cortex, barrel cortex | n.r. | 5x5 | n.r. | n.a. |
| Touzani et al. | n.r. | n.r. | yes | n.r. | n.r. | killed | intracerebral guide cannula implantation | n.a. | amygdala | n.r. | n.r. | n.r. | n.a. |
| Tsanov et al. | n.r. | n.r. | yes | <4 weeks | n.r. | n.r. | other | intracerebroventricular guide cannula implantation, deep reporting electrode implantation | dentate gyrus, ipsilateral cerebral ventricle | n.r. | n.r. | n.r. | n.a. |
| Wagner et al. | n.r. | n.r. | yes | n.r. | n.r. | n.r. | tbi, cci | n.a. | left parietal cortex | n.r. | n.r. | nonpermanent implant | cci |
| Wan et al. | n.r. | n.r. | no | n.a. | n.r. | killed | sole craniotomy | n.a. | left parietal cortex | n.r. | 4x6 | nonpermanent implant | n.a. |
| Wigren et al. | handled daily | n.r. | yes | <4 weeks | n.r. | killed | other | screw electrode implantation, intracerebral guide cannula implantation | n.r. | n.r. | n.r. | n.r. | n.a. |
| Worthen et al. | n.r. | n.r. | yes | n.r. | n.r. | n.r. | deep electrode implantation | n.a. | ventral hippocampus | n.r. | n.r. | n.r. | n.a. |
| Xue et al. | n.r. | n.r. | yes | <1 day | n.r. | killed | intracerebral injection cannula | n.a. | striatum | 0,4 | n.r. | nonpermanent implant | n.a. |
| Yoon et al. | n.r. | n.r. | yes | n.r. | n.r. | killed | electrode implantation | n.a. | cortex | n.r. | n.r. | n.r. | n.a. |
| Young et al. | n.r. | n.r. | yes | n.r. | n.r. | killed | other | deep electrode implantation, intracerebral guide cannula implantation | posterior hypothalamic nucleus, striatum, hippocampus | n.r. | n.r. | n.r. | n.a. |
| Yu et al. | n.r. | n.r. | n.r. | n.r. | n.r. | n.r. | deep electrode implantation | n.a. | cerebellum | n.r. | n.r. | n.r. | n.a. |
| Yurek et al. | n.r. | n.r. | yes | >8weeks | n.r. | killed | intracerebral injection cannula | n.a. | medial forebrain bundle, rostral pars compacta of substantia nigra | n.r. | n.r. | nonpermanent implant | n.a. |
| Zeng et al. | n.r. | n.r. | yes | <8 weeks | n.r. | n.r. | surface (screw) electrode implantation | n.a. | n.r. | n.r. | n.r. | n.r. | n.a. |

| study ID | general anesthesia, neuroleptics and hypnotics administered? If so, specify scheme (compound names OR n.r.) | drug / compound 1 (name OR n.a. OR n.r.) | administration route inhalation? (yes / no OR n.a.) | if so: volume percent  (Vol % OR n.a.) | if reported, induction:  (Vol% OR n.r. OR n.a.) | if reported, maintenance: (Vol% OR n.r. OR n.a.) | administration route injection?  (yes / no OR n.a.) | if so: injection route (s.c. OR i.p. OR i.v. OR n.a. OR n.r.) | if so:  dosage  (mg/kg OR n.a.) | administered how many times in total?  (number OR n.a. OR n.r.) | if so: administration interval (h post first administration OR n.r. OR n.a.) |
| --- | --- | --- | --- | --- | --- | --- | --- | --- | --- | --- | --- |
| Bartolomucci et al. | ketamine / xylazine | ketamine | no | n.a. | n.a. | n.a. | yes | i.p. | 100 | 1 | n.a. |
| Behrend et al. | ketamine / xylazine | ketamine | no | n.a. | n.a. | n.a. | yes | n.r. | 90 | 1 | n.a. |
| Biella et al. | isoflurane / pentobarbital | isoflurane | yes | 2,5 | n.r. | n.r. | no | n.a. | n.a. | n.a. | n.a. |
| Boni et al. | pentobarbital | pentobarbital | no | n.a. | n.a. | n.a. | yes | i.p. | 50 | once i.p. continuously i.v. | n.a. |
| Bramlett et al. | halothane | halothane | yes | 0,5 to 1 | n.r. | n.r. | no | n.a. | n.a. | n.a. | n.a. |
| Byun et al. | ether | ether | yes | n.r. | n.r. | n.r. | no | n.a. | n.a. | n.a. | n.a. |
| Caltana et al. | sevoflurane | sevoflurane | yes | 8 | n.r. | n.r. | no | n.a. | n.a. | n.a. | n.a. |
| Carcak et al. | ketamine / xylazine | ketamine | no | n.a. | n.a. | n.a. | yes | i.p. | 100 | 1 | n.a. |
| Cemil et al. | n.r. | n.r. | n.a. | n.a. | n.a. | n.a. | n.a. | n.a. | n.a. | n.a. | n.a. |
| Chen et al. D | chloral hydrate | chloral hydrate | no | n.a. | n.a. | n.a. | yes | i.p. | 300 | 1 | n.a. |
| Cifani et al. | tiletamine / zolazepam | tiletamine | no | n.a. | n.a. | n.a. | yes | i.m. | 200 | 1 | n.a. |
| Cunningham et al. | n.r. | n.r. | n.a. | n.a. | n.a. | n.a. | n.a. | n.a. | n.a. | n.a. | n.a. |
| Datta et al. | pentobarbital | pentobarbital | no | n.a. | n.a. | n.a. | yes | i.p. | 40 | 1 | n.a. |
| Diesch et al. | halothane | halothane | yes | n.a. | n.r. | 2 | no | n.a. | n.a. | n.a. | n.a. |
| Diguet et al. | ketamine / xylazine | ketamine | no | n.a. | n.a. | n.a. | yes | i.p. | 75 | 1 | n.a. |
| Ding et al. | isoflurane | isoflurane | yes | 2 | n.r. | n.r. | no | n.a. | n.a. | n.a. | n.a. |
| Doan et al. | isoflurane | isoflurane | yes | 1 | n.r. | n.r. | no | n.a. | n.a. | n.a. | n.a. |
| Doretto et al. | tribromoethanol | tribromoethanol | no | n.a. | n.a. | n.a. | yes | i.p. | n.r. | 1 | n.a. |
| Dux et al. | thiopental | thiopental | no | n.a. | n.a. | n.a. | yes | i.p. | 150 | n.r. | n.r. |
| Echegoyen et al. | n.r. | n.r. | n.a. | n.a. | n.a. | n.a. | n.a. | n.a. | n.a. | n.a. | n.a. |
| Ehrlichman et al. | isoflurane | isoflurane | yes | n.r. | n.r. | n.r. | no | n.a. | n.a. | n.a. | n.a. |
| Etholm et al. | n.r. | n.r. | n.a. | n.a. | n.a. | n.a. | n.a. | n.a. | n.a. | n.a. | n.a. |
| Farias et al. | isoflurane | isoflurane | yes | 3 to 3,5 | n.r. | n.r. | no | n.a. | n.a. | n.a. | n.a. |
| Foti et al. | pentobarbital | pentobarbital | no | n.a. | n.a. | n.a. | yes | i.p. | 50 | 1 | n.a. |
| Francois et al. | isoflurane | isoflurane | yes | n.a. | 5 | 2 to 3 | no | n.a. | n.a. | n.a. | n.a. |
| Francois et al. | ketamine / xylazine | ketamine | no | n.a. | n.a. | n.a. | yes | i.p. | 37 | 1 | n.a. |
| Fritsch et al. | ketamine / medetomidine | ketamine | no | n.a. | n.a. | n.a. | yes | i.p. | 60 | 1 | n.a. |
| Good et al. | ketamine / xylazine / acepromazine | ketamine | no | n.a. | n.a. | n.a. | yes | i.m. | 50 | preoperatively, 20% booster of cocktail every 45 min or as needed | n.r. |
| Griesbach et al. | isoflurane | isoflurane | yes | n.r. | 4 | 2 | no | n.a. | n.a. | n.a. | n.a. |
| Guidine et al. | halothane | halothane | yes | n.r. | 4 | 2 | no | n.a. | n.a. | n.a. | n.a. |
| Gurevicius et al. | pentobarbital / chloral hydrate | pentobarbital | no | n.a. | n.a. | n.a. | yes | i.p. | 50 | 1 | n.a. |
| Hart et al. | ketamine / xylazine | ketamine | no | n.a. | n.a. | n.a. | yes | i.p. | 100 | 1 | n.a. |
| Harvey et al. | n.r. | n.r. | n.a. | n.a. | n.a. | n.a. | n.a. | n.a. | n.a. | n.a. | n.a. |
| Hernandez-Gonzalez et al. | pentobarbital | pentobarbital | no | n.a. | n.a. | n.a. | yes | i.p. | 35 | 1 | n.a. |
| Ho et al. | tribromoethanol | tribromoethanol | no | n.a. | n.a. | n.a. | yes | i.p. | n.r. | 1 | n.a. |
| Holtmaat et al. | ketamine / xylazine | ketamine | no | n.a. | n.a. | n.a. | yes | i.p. | 100 | 1 | n.a. |
| Hrncic et al. | pentobarbital | pentobarbital | no | n.a. | n.a. | n.a. | yes | i.p. | 50 | 1 | n.a. |
| Huguet et al. | ketamine / xylazine | ketamine | no | n.a. | n.a. | n.a. | yes | i.p. | 110 | 1 | n.a. |
| Ishida et al. | pentobarbital | pentobarbital | no | n.a. | n.a. | n.a. | yes | i.p. | 35 | 1 | n.a. |
| Ito et al. | pentobarbital | pentobarbital | no | n.a. | n.a. | n.a. | yes | i.p. | 35 | 1 | n.a. |
| Itoh et al. B | pentobarbital | pentobarbital | no | n.a. | n.a. | n.a. | yes | i.p. | 50 | 1 | n.a. |
| Itoh et al. A | pentobarbital | pentobarbital | no | n.a. | n.a. | n.a. | yes | i.p. | 50 | 1 | n.a. |
| Jafri et al. | ketamine / xylazine | ketamine | no | n.a. | n.a. | n.a. | yes | i.p. | 40 to 80 | 1 | n.a. |
| Kalauzi et al. | ketamine / xylazine | ketamine | no | n.a. | n.a. | n.a. | yes | i.p. | 80 | 1 | n.a. |
| Katz et al. | isoflurane | isoflurane | yes | 2 | n.r. | n.r. | no | n.a. | n.a. | n.a. | n.a. |
| Kim et al. A | ketamine / xylazine | ketamine | no | n.a. | n.a. | n.a. | yes | n.r. | n.r. | 1 | n.r. |
| Lackovic et al. | chloral hydrate | chloral hydrate | no | n.a. | n.a. | n.a. | yes | i.p. | 300 | 1 | n.a. |
| Lee et al. B | ketamine / xylazine | ketamine | no | n.a. | n.a. | n.a. | yes | n.r. | 120 | 1 | n.r. |
| Lee et al. A | isoflurane | isoflurane | yes | n.r. | n.r. | n.r. | no | n.a. | n.a. | n.a. | n.a. |
| Li et al. C | diethyl ether / pentobarbital | diethyl ether | yes | n.r. | n.r. | n.r. | no | n.a. | n.a. | n.a. | n.a. |
| Liu et al. | ketamine / xylazine | ketamine | no | n.a. | n.a. | n.a. | yes | i.p. | 75 | 1 | n.a. |
| Lopez-Martin et al. | pentobarbital | pentobarbital | no | n.a. | n.a. | n.a. | yes | i.p. | 30 | 1 | n.a. |
| Lu et al. | isoflurane | isoflurane | yes | n.r. | n.r. | n.r. | no | n.a. | n.a. | n.a. | n.a. |
| Lundblad et al. | pentobarbital / ketamine | pentobarbital | no | n.a. | n.a. | n.a. | yes | i.p. | 50 | 1 | n.a. |
| Magloire et al. | pentobarbital / chloral hydrate | pentobarbital | no | n.a. | n.a. | n.a. | yes | i.p. | n.r. | 1 | n.a. |
| Mark et al. | ketamine / xylazine | ketamine | no | n.a. | n.a. | n.a. | yes | i.p. | 91 | 1 | n.a. |
| McCracken et al. | urethane | urethane | no | n.a. | n.a. | n.a. | yes | i.p. | 1,5 | 1 | n.a. |
| Meeren et al. | isoflurane | isoflurane | yes | n.r. | n.r. | n.r. | no | n.a. | n.a. | n.a. | n.a. |
| Merkler et al. | urethane / chloralose | urethane | no | n.a. | n.a. | n.a. | yes | n.r. | 1000 | 1 | n.a. |
| Merkler et al. | isoflurane | isoflurane | yes | n.r. | n.r. | n.r. | no | n.a. | n.a. | n.a. | n.a. |
| Mian et al. | n.r. | n.r. | n.a. | n.a. | n.a. | n.a. | n.a. | n.a. | n.a. | n.a. | n.a. |
| Mohammadi et al. | urethane | urethane | no | n.a. | n.a. | n.a. | yes | i.p. | 1500 | 1 | n.a. |
| Mollazaedh et al. | n.r. | n.r. | n.a. | n.a. | n.a. | n.a. | n.a. | n.a. | n.a. | n.a. | n.a. |
| Mukherjee et al. | ketamine / xylazine | ketamine | no | n.a. | n.a. | n.a. | yes | n.r. | 87 | 1 | n.a. |
| Nehlig et al. | n.r. | n.r. | n.a. | n.a. | n.a. | n.a. | n.a. | n.a. | n.a. | n.a. | n.a. |
| Nuki et al. | ketamine / xylazine | ketamine | no | n.a. | n.a. | n.a. | yes | i.p. | 100 | 1 | n.r. |
| Onyszchuk et al. | isoflurane | isoflurane | yes | n.r. | 2,5 | 1 | no | n.a. | n.a. | n.a. | n.a. |
| Oshima et al. | pentobarbital | pentobarbital | no | n.a. | n.a. | n.a. | yes | i.p. | 60 | 1 | n.a. |
| Potts et al. | tribromoethanol | tribromoethanol | no | n.a. | n.a. | n.a. | yes | n.r. | n.r. | 1 | n.a. |
| Qing et al. | chloral hydrate | chloral hydrate | no | n.a. | n.a. | n.a. | yes | i.p. | 300 to 350 | 1 | n.a. |
| Rahim et al. | isoflurane | isoflurane | yes | n.r. | n.r. | n.r. | no | n.a. | n.a. | n.a. | n.a. |
| Rimoli et al. | chloral hydrate | chloral hydrate | no | n.a. | n.a. | n.a. | yes | i.p. | 400 | 1 | n.a. |
| Roiko et al. | ketamine / xylazine | ketamine | no | n.a. | n.a. | n.a. | yes | i.m. | 75 | 1 | n.a. |
| Rudnick et al. | isoflurane | isoflurane | yes | n.r. | n.r. | n.r. | no | n.a. | n.a. | n.a. | n.a. |
| Sahin et al. | ketamine / chlorpromazine | ketamine | no | n.a. | n.a. | n.a. | yes | i.p. | 100 | 1 | n.a. |
| Samnick et al. | ketamine / xylazine | ketamine | no | n.a. | n.a. | n.a. | yes | i.p. | 70 | 1 | n.a. |
| Sasaki et al. | halothane | halothane | yes | n.a. | 5 | 1 | no | n.a. | n.a. | n.a. | n.a. |
| Schei et al. | isoflurane | isoflurane | yes | n.r. | 5 | 2,3 to 2,7 | no | n.a. | n.a. | n.a. | n.a. |
| Schmid et al. | urethane | urethane | no | n.a. | n.a. | n.a. | yes | n.r. | 1,5 | 1 | n.a. |
| Sekiya et al. | ketamine / xylazine | ketamine | no | n.a. | n.a. | n.a. | yes | i.p. | 100 | 1 | n.a. |
| Sher et al. | ketamine | ketamine | no | n.a. | n.a. | n.a. | yes | n.r. | n.r. | n.r. | n.r. |
| Shultz et al. | isoflurane | isoflurane | yes | n.r. | 4 | 2 | no | n.a. | n.a. | n.a. | n.a. |
| Silvani et al. | isoflurane | isoflurane | yes | 1 to 2 | n.r. | n.r. | no | n.a. | n.a. | n.a. | n.a. |
| Sinton et al. | ketamine / xylazine | ketamine | no | n.a. | n.a. | n.a. | yes | i.p. | 25 | 1 | n.r. |
| Song et al. | urethane | urethane | no | n.a. | n.a. | n.a. | yes | i.p. | 1500 | 1 | n.a. |
| Takahashi et al. | hypothermia OR isoflurane | isoflurane | yes | n.r. | n.r. | n.r. | no | n.a. | n.a. | n.a. | n.a. |
| Takahashi et al. | ketamine / xylazine | ketamine | no | n.a. | n.a. | n.a. | yes | n.r. | 60 | 1 | n.r. |
| Tanida et al. | pentobarbital | pentobarbital | no | n.a. | n.a. | n.a. | yes | i.p. | 35 | 1 | n.a. |
| Tchekalarova et al. | ketamine / xylazine | ketamine | no | n.a. | n.a. | n.a. | yes | i.p. | 80 | 1 | n.a. |
| Thomas et al. | halothane | halothane | yes | n.r. | n.r. | n.r. | no | n.a. | n.a. | n.a. | n.a. |
| Topchiy et al. | ketamine / xylazine / isoflurane | ketamine | no | n.a. | n.a. | n.a. | yes | i.m. | 100 | n.r. | n.r. |
| Touzani et al. | ketamine / xylazine | ketamine | no | n.a. | n.a. | n.a. | yes | i.p. | 63 | 1 | n.a. |
| Tsanov et al. | pentobarbital | pentobarbital | no | n.a. | n.a. | n.a. | yes | i.p. | 40 | 1 | n.a. |
| Wagner et al. | isoflurane | isoflurane | yes | n.r. | 4 | 1 to 1,5 | no | n.a. | n.a. | n.a. | n.a. |
| Wan et al. | pentobarbital | pentobarbital | no | n.a. | n.a. | n.a. | yes | i.p. | 45 | n.r. | n.r. |
| Wigren et al. | diazepam / medetomidine / ketamine | diazepam | no | n.a. | n.a. | n.a. | yes | i.p. | 2,5 | 1 | n.a. |
| Worthen et al. | n.r. | n.r. | n.a. | n.a. | n.a. | n.a. | n.a. | n.a. | n.a. | n.a. | n.a. |
| Xue et al. | ketamine / xylazine | ketamine | no | n.a. | n.a. | n.a. | yes | n.r. | n.r. | n.r. | n.r. |
| Yoon et al. | pentobarbital | pentobarbital | no | n.a. | n.a. | n.a. | yes | i.p. | 45 | 1 | n.a. |
| Young et al. | ketamine / xylazine | ketamine | no | n.a. | n.a. | n.a. | yes | i.m. | 85 | 1 | n.a. |
| Yu et al. | urethane | urethane | no | n.a. | n.a. | n.a. | yes | i.p. | 1750 | 1 | n.a. |
| Yurek et al. | n.r. | n.r. | n.a. | n.a. | n.a. | n.a. | n.a. | n.a. | n.a. | n.a. | n.a. |
| Zeng et al. | isoflurane | Isoflurane | 1 to 2 | n.r. | n.r. | n.a. | n.a. | n.a. | n.a. | n.a. | n.a. |

| study ID | drug / compound 2 (name OR n.a.) | administration route injection?  (yes / no OR n.a.) | if so:  administration route (s.c. OR i.p. OR i.v. OR n.a. OR n.r.) | if so:  dosage  (mg/kg OR n.a.) | administered how many times in total?  (number OR n.a. OR n.r.) | drug / compound 3 (name OR n.a.) | administration route inhalation? (yes / no OR n.a.) | if so: volume percent  (Vol % OR n.a.) | administration route injection?  (yes / no OR n.a.) | if so:  administration route (s.c. OR i.p. OR i.v. OR n.a. OR n.r.) | if so:  dosage  (mg/kg OR n.a.) | administered how many times in total?  (number OR n.a. OR n.r.) |
| --- | --- | --- | --- | --- | --- | --- | --- | --- | --- | --- | --- | --- |
| Bartolomucci et al. | xylazine | yes | i.p. | 5 | n.a. | n.a. | n.a. | n.a. | n.a. | n.a. | n.a. | n.a. |
| Behrend et al. | xylazine | yes | n.r. | 10 | 1 | n.a. | n.a. | n.a. | n.a. | n.a. | n.a. | n.a. |
| Biella et al. | pentobarbital | yes | i.p. | 2,5 | n.r. | n.a. | n.a. | n.a. | n.a. | n.a. | n.a. | n.a. |
| Boni et al. | n.a. | n.a. | n.a. | n.a. | n.a. | n.a. | n.a. | n.a. | n.a. | n.a. | n.a. | n.a. |
| Bramlett et al. | n.a. | n.a. | n.a. | n.a. | n.a. | n.a. | n.a. | n.a. | n.a. | n.a. | n.a. | n.a. |
| Byun et al. | n.a. | n.a. | n.a. | n.a. | n.a. | n.a. | n.a. | n.a. | n.a. | n.a. | n.a. | n.a. |
| Caltana et al. | n.a. | n.a. | n.a. | n.a. | n.a. | n.a. | n.a. | n.a. | n.a. | n.a. | n.a. | n.a. |
| Carcak et al. | xylazine | yes | i.p. | 10 | 1 | n.a. | n.a. | n.a. | n.a. | n.a. | n.a. | n.a. |
| Cemil et al. | n.a. | n.a. | n.a. | n.a. | n.a. | n.a. | n.a. | n.a. | n.a. | n.a. | n.a. | n.a. |
| Chen et al. D | n.a. | n.a. | n.a. | n.a. | n.a. | n.a. | n.a. | n.a. | n.a. | n.a. | n.a. | n.a. |
| Cifani et al. | zolazepam | yes | i.m. | 200 | 1 | n.a. | n.a. | n.a. | n.a. | n.a. | n.a. | n.a. |
| Cunningham et al. | n.a. | n.a. | n.a. | n.a. | n.a. | n.a. | n.a. | n.a. | n.a. | n.a. | n.a. | n.a. |
| Datta et al. | n.a. | n.a. | n.a. | n.a. | n.a. | n.a. | n.a. | n.a. | n.a. | n.a. | n.a. | n.a. |
| Diesch et al. | n.a. | n.a. | n.a. | n.a. | n.a. | n.a. | n.a. | n.a. | n.a. | n.a. | n.a. | n.a. |
| Diguet et al. | xylazine | yes | i.p. | 10 | 1 | n.a. | n.a. | n.a. | n.a. | n.a. | n.a. | n.a. |
| Ding et al. | n.a. | n.a. | n.a. | n.a. | n.a. | n.a. | n.a. | n.a. | n.a. | n.a. | n.a. | n.a. |
| Doan et al. | n.a. | n.a. | n.a. | n.a. | n.a. | n.a. | n.a. | n.a. | n.a. | n.a. | n.a. | n.a. |
| Doretto et al. | n.a. | n.a. | n.a. | n.a. | n.a. | n.a. | n.a. | n.a. | n.a. | n.a. | n.a. | n.a. |
| Dux et al. | n.a. | n.a. | n.a. | n.a. | n.a. | n.a. | n.a. | n.a. | n.a. | n.a. | n.a. | n.a. |
| Echegoyen et al. | n.a. | n.a. | n.a. | n.a. | n.a. | n.a. | n.a. | n.a. | n.a. | n.a. | n.a. | n.a. |
| Ehrlichman et al. | n.a. | n.a. | n.a. | n.a. | n.a. | n.a. | n.a. | n.a. | n.a. | n.a. | n.a. | n.a. |
| Etholm et al. | n.r. | n.a. | n.a. | n.a. | n.a. | n.a. | n.a. | n.a. | n.a. | n.a. | n.a. | n.a. |
| Farias et al. | n.a. | n.a. | n.a. | n.a. | n.a. | n.a. | n.a. | n.a. | n.a. | n.a. | n.a. | n.a. |
| Foti et al. | n.a. | n.a. | n.a. | n.a. | n.a. | n.a. | n.a. | n.a. | n.a. | n.a. | n.a. | n.a. |
| Francois et al. | n.a. | n.a. | n.a. | n.a. | n.a. | n.a. | n.a. | n.a. | n.a. | n.a. | n.a. | n.a. |
| Francois et al. | xylazine | yes | i.p. | 5,5 | 1 | n.a. | n.a. | n.a. | n.a. | n.a. | n.a. | n.a. |
| Fritsch et al. | medetomidine | yes | i.p. | 0,5 | 1 | n.a. | n.a. | n.a. | n.a. | n.a. | n.a. | n.a. |
| Good et al. | xylazine | yes | i.m. | 10 | preoperatively, 20% booster of cocktail every 45 min or as needed | acepromazine | no | n.a. | yes |  | 1 | 1 |
| Griesbach et al. | n.a. | n.a. | n.a. | n.a. | n.a. | n.a. | n.a. | n.a. | n.a. | n.a. | n.a. | n.a. |
| Guidine et al. | n.a. | n.a. | n.a. | n.a. | n.a. | n.a. | n.a. | n.a. | n.a. | n.a. | n.a. | n.a. |
| Gurevicius et al. | chloral hydrate | yes | i.p. | 50 | 1 | n.a. | n.a. | n.a. | n.a. | n.a. | n.a. | n.a. |
| Hart et al. | xylazine | yes | i.p. | 20 | 1 | n.a. | n.a. | n.a. | n.a. | n.a. | n.a. | n.a. |
| Harvey et al. | n.a. | n.a. | n.a. | n.a. | n.a. | n.a. | n.a. | n.a. | n.a. | n.a. | n.a. | n.a. |
| Hernandez-Gonzalez et al. | n.a. | n.a. | n.a. | n.a. | n.a. | n.a. | n.a. | n.a. | n.a. | n.a. | n.a. | n.a. |
| Ho et al. | n.a. | n.a. | n.a. | n.a. | n.a. | n.a. | n.a. | n.a. | n.a. | n.a. | n.a. | n.a. |
| Holtmaat et al. | xylazine | yes | i.p. | 10 | 1 | n.a. | n.a. | n.a. | n.a. | n.a. | n.a. | n.a. |
| Hrncic et al. | n.a. | n.a. | n.a. | n.a. | n.a. | n.a. | n.a. | n.a. | n.a. | n.a. | n.a. | n.a. |
| Huguet et al. | xylazine | yes | i.p. | n.r. | 1 | n.a. | n.a. | n.a. | n.a. | n.a. | n.a. | n.a. |
| Ishida et al. | n.a. | n.a. | n.a. | n.a. | n.a. | n.a. | n.a. | n.a. | n.a. | n.a. | n.a. | n.a. |
| Ito et al. | n.a. | n.a. | n.a. | n.a. | n.a. | n.a. | n.a. | n.a. | n.a. | n.a. | n.a. | n.a. |
| Itoh et al. B | n.a. | n.a. | n.a. | n.a. | n.a. | n.a. | n.a. | n.a. | n.a. | n.a. | n.a. | n.a. |
| Itoh et al. A | n.a. | n.a. | n.a. | n.a. | n.a. | n.a. | n.a. | n.a. | n.a. | n.a. | n.a. | n.a. |
| Jafri et al. | xylazine | yes | i.p. | 5 to 10 | 1 | n.a. | n.a. | n.a. | n.a. | n.a. | n.a. | n.a. |
| Kalauzi et al. | xylazine | yes | i.p. | 5 | 1 | n.a. | n.a. | n.a. | n.a. | n.a. | n.a. | n.a. |
| Katz et al. | n.a. | n.a. | n.a. | n.a. | n.a. | n.a. | n.a. | n.a. | n.a. | n.a. | n.a. | n.a. |
| Kim et al. A | xylazine | yes | n.r. | n.r. | n.r. | n.a. | n.a. | n.a. | n.a. | n.a. | n.a. | n.a. |
| Lackovic et al. | n.a. | n.a. | n.a. | n.a. | n.a. | n.a. | n.a. | n.a. | n.a. | n.a. | n.a. | n.a. |
| Lee et al. B | xylazine | yes | n.r. | 6 | 1 | n.a. | n.a. | n.a. | n.a. | n.a. | n.a. | n.a. |
| Lee et al. A | n.a. | n.a. | n.a. | n.a. | n.a. | n.a. | n.a. | n.a. | n.a. | n.a. | n.a. | n.a. |
| Li et al. C | pentobarbital | yes | n.r. | 30 | 1 | n.a. | n.a. | n.a. | n.a. | n.a. | n.a. | n.a. |
| Liu et al. | xylazine | yes | i.p. | 10 | 1 | n.a. | n.a. | n.a. | n.a. | n.a. | n.a. | n.a. |
| Lopez-Martin et al. | n.a. | n.a. | n.a. | n.a. | n.a. | n.a. | n.a. | n.a. | n.a. | n.a. | n.a. | n.a. |
| Lu et al. | n.a. | n.a. | n.a. | n.a. | n.a. | n.a. | n.a. | n.a. | n.a. | n.a. | n.a. | n.a. |
| Lundblad et al. | ketamine | yes | i.p. | 50 | 1 | n.a. | n.a. | n.a. | n.a. | n.a. | n.a. | n.a. |
| Magloire et al. | chloral hydrate | yes | i.p. | n.r. | 1 | n.a. | n.a. | n.a. | n.a. | n.a. | n.a. | n.a. |
| Mark et al. | xylazine | yes | i.p. | 9,1 | 1 | n.a. | n.a. | n.a. | n.a. | n.a. | n.a. | n.a. |
| McCracken et al. | n.a. | n.a. | n.a. | n.a. | n.a. | n.a. | n.a. | n.a. | n.a. | n.a. | n.a. | n.a. |
| Meeren et al. | n.a. | n.a. | n.a. | n.a. | n.a. | n.a. | n.a. | n.a. | n.a. | n.a. | n.a. | n.a. |
| Merkler et al. | chloralose | yes | n.r. | 400 | 1 | n.a. | n.a. | n.a. | n.a. | n.a. | n.a. | n.a. |
| Merkler et al. | n.a. | n.a. | n.a. | n.a. | n.a. | n.a. | n.a. | n.a. | n.a. | n.a. | n.a. | n.a. |
| Mian et al. | n.a. | n.a. | n.a. | n.a. | n.a. | n.a. | n.a. | n.a. | n.a. | n.a. | n.a. | n.a. |
| Mohammadi et al. | n.a. | n.a. | n.a. | n.a. | n.a. | n.a. | n.a. | n.a. | n.a. | n.a. | n.a. | n.a. |
| Mollazaedh et al. | n.a. | n.a. | n.a. | n.a. | n.a. | n.a. | n.a. | n.a. | n.a. | n.a. | n.a. | n.a. |
| Mukherjee et al. | xylazine | yes | n.r. | 13 | 1 | n.a. | n.a. | n.a. | n.a. | n.a. | n.a. | n.a. |
| Nehlig et al. | n.a. | n.a. | n.a. | n.a. | n.a. | n.a. | n.a. | n.a. | n.a. | n.a. | n.a. | n.a. |
| Nuki et al. | xylazine | yes | i.p. | 10 | 1 | n.a. | n.a. | n.a. | n.a. | n.a. | n.a. | n.a. |
| Onyszchuk et al. | n.a. | n.a. | n.a. | n.a. | n.a. | n.a. | n.a. | n.a. | n.a. | n.a. | n.a. | n.a. |
| Oshima et al. | n.a. | n.a. | n.a. | n.a. | n.a. | n.a. | n.a. | n.a. | n.a. | n.a. | n.a. | n.a. |
| Potts et al. | n.a. | n.a. | n.a. | n.a. | n.a. | n.a. | n.a. | n.a. | n.a. | n.a. | n.a. | n.a. |
| Qing et al. | n.a. | n.a. | n.a. | n.a. | n.a. | n.a. | n.a. | n.a. | n.a. | n.a. | n.a. | n.a. |
| Rahim et al. | n.a. | n.a. | n.a. | n.a. | n.a. | n.a. | n.a. | n.a. | n.a. | n.a. | n.a. | n.a. |
| Rimoli et al. | n.a. | n.a. | n.a. | n.a. | n.a. | n.a. | n.a. | n.a. | n.a. | n.a. | n.a. | n.a. |
| Roiko et al. | xylazine | yes | i.m. | 7,5 | 1 | n.a. | n.a. | n.a. | n.a. | n.a. | n.a. | n.a. |
| Rudnick et al. | n.a. | n.a. | n.a. | n.a. | n.a. | n.a. | n.a. | n.a. | n.a. | n.a. | n.a. | n.a. |
| Sahin et al. | chlorpromazine | yes | i.p. | 1 | 1 | n.a. | n.a. | n.a. | n.a. | n.a. | n.a. | n.a. |
| Samnick et al. | xylazine | yes | i.p. | 20 | 1 | n.a. | n.a. | n.a. | n.a. | n.a. | n.a. | n.a. |
| Sasaki et al. | n.a. | n.a. | n.a. | n.a. | n.a. | n.a. | n.a. | n.a. | n.a. | n.a. | n.a. | n.a. |
| Schei et al. | n.a. | n.a. | n.a. | n.a. | n.a. | n.a. | n.a. | n.a. | n.a. | n.a. | n.a. | n.a. |
| Schmid et al. | n.a. | n.a. | n.a. | n.a. | n.a. | n.a. | n.a. | n.a. | n.a. | n.a. | n.a. | n.a. |
| Sekiya et al. | xylazine | yes | i.p. | 9 | 1 | n.a. | n.a. | n.a. | n.a. | n.a. | n.a. | n.a. |
| Sher et al. | n.a. | n.a. | n.a. | n.a. | n.a. | n.a. | n.a. | n.a. | n.a. | n.a. | n.a. | n.a. |
| Shultz et al. | n.a. | n.a. | n.a. | n.a. | n.a. | n.a. | n.a. | n.a. | n.a. | n.a. | n.a. | n.a. |
| Silvani et al. | n.a. | n.a. | n.a. | n.a. | n.a. | n.a. | n.a. | n.a. | n.a. | n.a. | n.a. | n.a. |
| Sinton et al. | xylazine | yes | i.p. | 2,5 | 1 | n.a. | n.a. | n.a. | n.a. | n.a. | n.a. | n.a. |
| Song et al. | n.a. | n.a. | n.a. | n.a. | n.a. | n.a. | n.a. | n.a. | n.a. | n.a. | n.a. | n.a. |
| Takahashi et al. | hypothermia | no | n.a. | n.a. | n.a. | n.a. | n.a. | n.a. | n.a. | n.a. | n.a. | n.a. |
| Takahashi et al. | xylazine | yes | n.r. | 100 | 1 | n.a. | n.a. | n.a. | n.a. | n.a. | n.a. | n.a. |
| Tanida et al. | n.a. | n.a. | n.a. | n.a. | n.a. | n.a. | n.a. | n.a. | n.a. | n.a. | n.a. | n.a. |
| Tchekalarova et al. | xylazine | yes | i.p. | 20 | 1 | n.a. | n.a. | n.a. | n.a. | n.a. | n.a. | n.a. |
| Thomas et al. | n.a. | n.a. | n.a. | n.a. | n.a. | n.a. | n.a. | n.a. | n.a. | n.a. | n.a. | n.a. |
| Topchiy et al. | xylazine | yes | i.m. | 10 | n.r. | isoflurane | yes | n.r. | no | n.a. | n.a. | n.a. |
| Touzani et al. | xylazine | yes | i.p. | 9,4 | 1 | n.a. | n.a. | n.a. | no | n.a. | n.a. | n.a. |
| Tsanov et al. | n.a. | n.a. | n.a. | n.a. | n.a. | n.a. | n.a. | n.a. | n.a. | n.a. | n.a. | n.a. |
| Wagner et al. | n.a. | n.a. | n.a. | n.a. | n.a. | n.a. | n.a. | n.a. | n.a. | n.a. | n.a. | n.a. |
| Wan et al. | n.a. | n.a. | n.a. | n.a. | n.a. | n.a. | n.a. | n.a. | n.a. | n.a. | n.a. | n.a. |
| Wigren et al. | medetomidine | yes | i.p. | 0,4 | 1 | ketamine | no | n.a. | yes | i.p. | 60 | 1 |
| Worthen et al. | n.a. | n.a. | n.a. | n.a. | n.a. | n.a. | n.a. | n.a. | n.a. | n.a. | n.a. | n.a. |
| Xue et al. | xylazine | yes | n.r. | n.r. | n.r. | n.a. | n.a. | n.a. | n.a. | n.a. | n.a. | n.a. |
| Yoon et al. | n.a. | n.a. | n.a. | n.a. | n.a. | n.a. | n.a. | n.a. | n.a. | n.a. | n.a. | n.a. |
| Young et al. | xylazine | yes | i.m. | 15 | 1 | n.a. | n.a. | n.a. | n.a. | n.a. | n.a. | n.a. |
| Yu et al. | n.a. | n.a. | n.a. | n.a. | n.a. | n.a. | n.a. | n.a. | n.a. | n.a. | n.a. | n.a. |
| Yurek et al. | n.a. | n.a. | n.a. | n.a. | n.a. | n.a. | n.a. | n.a. | n.a. | n.a. | n.a. | n.a. |
| Zeng et al. | n.a. | n.a. | n.a. | n.a. | n.a. | n.a. | n.a. | n.a. | n.a. | n.a. | n.a. | n.a. |

| study ID | local anesthesia administered?  (yes / no OR n.r.) | drug / compound 1   (name OR n.a.) | administration route   (s.c. OR applied topically OR n.a.) | dosage   (mg/kg) OR n.r. OR n.a. | injection volume   (ml/animal OR n.r. OR n.a.) | timepoint of first administration   (pre-, intra-, postsurgically OR n.a.) | if reported:  immediately before surgery OR immediately after surgery OR n.r. OR n.a. | administered how many times in total?   (number OR n.a.) | if so: administration interval   (h post first administration Or n.r. OR n.a.) | if reported: drug concentration   (mg/ml OR n.r. OR n.a.) | if reported:  pharmaceutical formulation  (e.g. solution OR n.r. OR n.a.) |
| --- | --- | --- | --- | --- | --- | --- | --- | --- | --- | --- | --- |
| Bartolomucci et al. | no | n.a. | n.a. | n.a. | n.a. | n.a. | n.a. | n.a. | n.a. | n.a. | n.a. |
| Behrend et al. | no | n.a. | n.a. | n.a. | n.a. | n.a. | n.a. | n.a. | n.a. | n.a. | n.a. |
| Biella et al. | no | n.a. | n.a. | n.a. | n.a. | n.a. | n.a. | n.a. | n.a. | n.a. | n.a. |
| Boni et al. | no | n.a. | n.a. | n.a. | n.a. | n.a. | n.a. | n.a. | n.a. | n.a. | n.a. |
| Bramlett et al. | no | n.a. | n.a. | n.a. | n.a. | n.a. | n.a. | n.a. | n.a. | n.a. | n.a. |
| Byun et al. | no | n.a. | n.a. | n.a. | n.a. | n.a. | n.a. | n.a. | n.a. | n.a. | n.a. |
| Caltana et al. | no | n.a. | n.a. | n.a. | n.a. | n.a. | n.a. | n.a. | n.a. | n.a. | n.a. |
| Carcak et al. | no | n.a. | n.a. | n.a. | n.a. | n.a. | n.a. | n.a. | n.a. | n.a. | n.a. |
| Cemil et al. | no | n.a. | n.a. | n.a. | n.a. | n.a. | n.a. | n.a. | n.a. | n.a. | n.a. |
| Chen et al. D | no | n.a. | n.a. | n.a. | n.a. | n.a. | n.a. | n.a. | n.a. | n.a. | n.a. |
| Cifani et al. | no | n.a. | n.a. | n.a. | n.a. | n.a. | n.a. | n.a. | n.a. | n.a. | n.a. |
| Cunningham et al. | no | n.a. | n.a. | n.a. | n.a. | n.a. | n.a. | n.a. | n.a. | n.a. | n.a. |
| Datta et al. | no | n.a. | n.a. | n.a. | n.a. | n.a. | n.a. | n.a. | n.a. | n.a. | n.a. |
| Diesch et al. | no | n.a. | n.a. | n.a. | n.a. | n.a. | n.a. | n.a. | n.a. | n.a. | n.a. |
| Diguet et al. | no | n.a. | n.a. | n.a. | n.a. | n.a. | n.a. | n.a. | n.a. | n.a. | n.a. |
| Ding et al. | no | n.a. | n.a. | n.a. | n.a. | n.a. | n.a. | n.a. | n.a. | n.a. | n.a. |
| Doan et al. | no | n.a. | n.a. | n.a. | n.a. | n.a. | n.a. | n.a. | n.a. | n.a. | n.a. |
| Doretto et al. | no | n.a. | n.a. | n.a. | n.a. | n.a. | n.a. | n.a. | n.a. | n.a. | n.a. |
| Dux et al. | no | n.a. | n.a. | n.a. | n.a. | n.a. | n.a. | n.a. | n.a. | n.a. | n.a. |
| Echegoyen et al. | no | n.a. | n.a. | n.a. | n.a. | n.a. | n.a. | n.a. | n.a. | n.a. | n.a. |
| Ehrlichman et al. | no | n.a. | n.a. | n.a. | n.a. | n.a. | n.a. | n.a. | n.a. | n.a. | n.a. |
| Etholm et al. | no | n.a. | n.a. | n.a. | n.a. | n.a. | n.a. | n.a. | n.a. | n.a. | n.a. |
| Farias et al. | no | n.a. | n.a. | n.a. | n.a. | n.a. | n.a. | n.a. | n.a. | n.a. | n.a. |
| Foti et al. | no | n.a. | n.a. | n.a. | n.a. | n.a. | n.a. | n.a. | n.a. | n.a. | n.a. |
| Francois et al. | no | n.a. | n.a. | n.a. | n.a. | n.a. | n.a. | n.a. | n.a. | n.a. | n.a. |
| Francois et al. | no | n.a. | n.a. | n.a. | n.a. | n.a. | n.a. | n.a. | n.a. | n.a. | n.a. |
| Fritsch et al. | no | n.a. | n.a. | n.a. | n.a. | n.a. | n.a. | n.a. | n.a. | n.a. | n.a. |
| Good et al. | no | n.a. | n.a. | n.a. | n.a. | n.a. | n.a. | n.a. | n.a. | n.a. | n.a. |
| Griesbach et al. | yes | bupivacaine | s.c. | 0,25 | n.r. | postsurgically | immediately after surgery | 1 | n.a. | n.r. | n.r. |
| Guidine et al. | no | n.a. | n.a. | n.a. | n.a. | n.a. | n.a. | n.a. | n.a. | n.a. | n.a. |
| Gurevicius et al. | no | n.a. | n.a. | n.a. | n.a. | n.a. | n.a. | n.a. | n.a. | n.a. | n.a. |
| Hart et al. | yes | procaine | i.p. | 300 | 0,3 | postsurgically | immediately after surgery | 1 | n.a. | n.a. | solution |
| Harvey et al. | no | n.a. | n.a. | n.a. | n.a. | n.a. | n.a. | n.a. | n.a. | n.a. | n.a. |
| Hernandez-Gonzalez et al. | no | n.a. | n.a. | n.a. | n.a. | n.a. | n.a. | n.a. | n.a. | n.a. | n.a. |
| Ho et al. | no | n.a. | n.a. | n.a. | n.a. | n.a. | n.a. | n.a. | n.a. | n.a. | n.a. |
| Holtmaat et al. | No | n.a. | n.a. | n.a. | n.a. | n.a. | n.a. | n.a. | n.a. | n.a. | n.r. |
| Hrncic et al. | no | n.a. | n.a. | n.a. | n.a. | n.a. | n.a. | n.a. | n.a. | n.a. | n.a. |
| Huguet et al. | no | n.a. | n.a. | n.a. | n.a. | n.a. | n.a. | n.a. | n.a. | n.a. | n.a. |
| Ishida et al. | no | n.a. | n.a. | n.a. | n.a. | n.a. | n.a. | n.a. | n.a. | n.a. | n.a. |
| Ito et al. | no | n.a. | n.a. | n.a. | n.a. | n.a. | n.a. | n.a. | n.a. | n.a. | n.a. |
| Itoh et al. B | no | n.a. | n.a. | n.a. | n.a. | n.a. | n.a. | n.a. | n.a. | n.a. | n.a. |
| Itoh et al. A | no | n.a. | n.a. | n.a. | n.a. | n.a. | n.a. | n.a. | n.a. | n.a. | n.a. |
| Jafri et al. | no | n.a. | n.a. | n.a. | n.a. | n.a. | n.a. | n.a. | n.a. | n.a. | n.a. |
| Kalauzi et al. | no | n.a. | n.a. | n.a. | n.a. | n.a. | n.a. | n.a. | n.a. | n.a. | n.a. |
| Katz et al. | no | n.a. | n.a. | n.a. | n.a. | n.a. | n.a. | n.a. | n.a. | n.a. | n.a. |
| Kim et al. A | no | n.a. | n.a. | n.a. | n.a. | n.a. | n.a. | n.a. | n.a. | n.a. | n.a. |
| Lackovic et al. | no | n.a. | n.a. | n.a. | n.a. | n.a. | n.a. | n.a. | n.a. | n.a. | n.a. |
| Lee et al. B | no | n.a. | n.a. | n.a. | n.a. | n.a. | n.a. | n.a. | n.a. | n.a. | n.a. |
| Lee et al. A | no | n.a. | n.a. | n.a. | n.a. | n.a. | n.a. | n.a. | n.a. | n.a. | n.a. |
| Li et al. C | no | n.a. | n.a. | n.a. | n.a. | n.a. | n.a. | n.a. | n.a. | n.a. | n.a. |
| Liu et al. | no | n.a. | n.a. | n.a. | n.a. | n.a. | n.a. | n.a. | n.a. | n.a. | n.a. |
| Lopez-Martin et al. | no | n.a. | n.a. | n.a. | n.a. | n.a. | n.a. | n.a. | n.a. | n.a. | n.a. |
| Lu et al. | no | n.a. | n.a. | n.a. | n.a. | n.a. | n.a. | n.a. | n.a. | n.a. | n.a. |
| Lundblad et al. | no | n.a. | n.a. | n.a. | n.a. | n.a. | n.a. | n.a. | n.a. | n.a. | n.a. |
| Magloire et al. | no | n.a. | n.a. | n.a. | n.a. | n.a. | n.a. | n.a. | n.a. | n.a. | n.a. |
| Mark et al. | no | n.a. | n.a. | n.a. | n.a. | n.a. | n.a. | n.a. | n.a. | n.a. | n.a. |
| McCracken et al. | no | n.a. | n.a. | n.a. | n.a. | n.a. | n.a. | n.a. | n.a. | n.a. | n.a. |
| Meeren et al. | yes | lidocaine | s.c. | n.r. | n.r. | presurgically | n.r. | 1 | n.a. | 0,02 | n.r. |
| Merkler et al. | no | n.a. | n.a. | n.a. | n.a. | n.a. | n.a. | n.a. | n.a. | n.a. | n.a. |
| Merkler et al. | no | n.a. | n.a. | n.a. | n.a. | n.a. | n.a. | n.a. | n.a. | n.a. | n.a. |
| Mian et al. | no | n.a. | n.a. | n.a. | n.a. | n.a. | n.a. | n.a. | n.a. | n.a. | n.a. |
| Mohammadi et al. | yes | lidocaine | s.c. | n.r. | 0,2 | presurgically | immediately before surgery | 1 | n.a. | n.r. | n.r. |
| Mollazaedh et al. | no | n.a. | n.a. | n.a. | n.a. | n.a. | n.a. | n.a. | n.a. | n.a. | n.a. |
| Mukherjee et al. | no | n.a. | n.a. | n.a. | n.a. | n.a. | n.a. | n.a. | n.a. | n.a. | n.a. |
| Nehlig et al. | no | n.a. | n.a. | n.a. | n.a. | n.a. | n.a. | n.a. | n.a. | n.a. | n.a. |
| Nuki et al. | no | n.a. | n.a. | n.a. | n.a. | n.a. | n.a. | n.a. | n.a. | n.a. | n.a. |
| Onyszchuk et al. | no | n.a. | n.a. | n.a. | n.a. | n.a. | n.a. | n.a. | n.a. | n.a. | n.a. |
| Oshima et al. | no | n.a. | n.a. | n.a. | n.a. | n.a. | n.a. | n.a. | n.a. | n.a. | n.a. |
| Potts et al. | no | n.a. | n.a. | n.a. | n.a. | n.a. | n.a. | n.a. | n.a. | n.a. | n.a. |
| Qing et al. | no | n.a. | n.a. | n.a. | n.a. | n.a. | n.a. | n.a. | n.a. | n.a. | n.a. |
| Rahim et al. | no | n.a. | n.a. | n.a. | n.a. | n.a. | n.a. | n.a. | n.a. | n.a. | n.a. |
| Rimoli et al. | no | n.a. | n.a. | n.a. | n.a. | n.a. | n.a. | n.a. | n.a. | n.a. | n.a. |
| Roiko et al. | no | n.a. | n.a. | n.a. | n.a. | n.a. | n.a. | n.a. | n.a. | n.a. | n.a. |
| Rudnick et al. | no | n.a. | n.a. | n.a. | n.a. | n.a. | n.a. | n.a. | n.a. | n.a. | n.a. |
| Sahin et al. | no | n.a. | n.a. | n.a. | n.a. | n.a. | n.a. | n.a. | n.a. | n.a. | n.a. |
| Samnick et al. | no | n.a. | n.a. | n.a. | n.a. | n.a. | n.a. | n.a. | n.a. | n.a. | n.a. |
| Sasaki et al. | no | n.a. | n.a. | n.a. | n.a. | n.a. | n.a. | n.a. | n.a. | n.a. | n.a. |
| Schei et al. | yes | n.r. | n.r. | n.r. | n.r. | n.a. | n.r. | 1 | n.a. | n.r. | n.r. |
| Schmid et al. | no | n.a. | n.a. | n.a. | n.a. | n.a. | n.a. | n.a. | n.a. | n.a. | n.a. |
| Sekiya et al. | no | n.a. | n.a. | n.a. | n.a. | n.a. | n.a. | n.a. | n.a. | n.a. | n.a. |
| Sher et al. | no | n.a. | n.a. | n.a. | n.a. | n.a. | n.a. | n.a. | n.a. | n.a. | n.a. |
| Shultz et al. | no | n.a. | n.a. | n.a. | n.a. | n.a. | n.a. | n.a. | n.a. | n.a. | n.a. |
| Silvani et al. | no | n.a. | n.a. | n.a. | n.a. | n.a. | n.a. | n.a. | n.a. | n.a. | n.a. |
| Sinton et al. | no | n.a. | n.a. | n.a. | n.a. | n.a. | n.a. | n.a. | n.a. | n.a. | n.a. |
| Song et al. | no | n.a. | n.a. | n.a. | n.a. | n.a. | n.a. | n.a. | n.a. | n.a. | n.a. |
| Takahashi et al. | no | n.a. | n.a. | n.a. | n.a. | n.a. | n.a. | n.a. | n.a. | n.a. | n.a. |
| Takahashi et al. | no | n.a. | n.a. | n.a. | n.a. | n.a. | n.a. | n.a. | n.a. | n.a. | n.a. |
| Tanida et al. | no | n.a. | n.a. | n.a. | n.a. | n.a. | n.a. | n.a. | n.a. | n.a. | n.a. |
| Tchekalarova et al. | yes | procaine | n.r. | n.r. | n.r. | presurgically | immediately before surgery | 1 | n.a. | n.r. | n.r. |
| Thomas et al. | no | n.a. | n.a. | n.a. | n.a. | n.a. | n.a. | n.a. | n.a. | n.a. | n.a. |
| Topchiy et al. | no | n.a. | n.a. | n.a. | n.a. | n.a. | n.a. | n.a. | n.a. | n.a. | n.a. |
| Touzani et al. | no | n.a. | n.a. | n.a. | n.a. | n.a. | n.a. | n.a. | n.a. | n.a. | n.a. |
| Tsanov et al. | no | n.a. | n.a. | n.a. | n.a. | n.a. | n.a. | n.a. | n.a. | n.a. | n.a. |
| Wagner et al. | no | n.a. | n.a. | n.a. | n.a. | n.a. | n.a. | n.a. | n.a. | n.a. | n.a. |
| Wan et al. | no | n.a. | n.a. | n.a. | n.a. | n.a. | n.a. | n.a. | n.a. | n.a. | n.a. |
| Wigren et al. | no | n.a. | n.a. | n.a. | n.a. | n.a. | n.a. | n.a. | n.a. | n.a. | n.a. |
| Worthen et al. | no | n.a. | n.a. | n.a. | n.a. | n.a. | n.a. | n.a. | n.a. | n.a. | n.a. |
| Xue et al. | no | n.a. | n.a. | n.a. | n.a. | n.a. | n.a. | n.a. | n.a. | n.a. | n.a. |
| Yoon et al. | no | n.a. | n.a. | n.a. | n.a. | n.a. | n.a. | n.a. | n.a. | n.a. | n.a. |
| Young et al. | no | n.a. | n.a. | n.a. | n.a. | n.a. | n.a. | n.a. | n.a. | n.a. | n.a. |
| Yu et al. | no | n.a. | n.a. | n.a. | n.a. | n.a. | n.a. | n.a. | n.a. | n.a. | n.a. |
| Yurek et al. | no | n.a. | n.a. | n.a. | n.a. | n.a. | n.a. | n.a. | n.a. | n.a. | n.a. |
| Zeng et al. | no | n.a. | n.a. | n.a. | n.a. | n.a. | n.a. | n.a. | n.a. | n.a. | n.a. |

| study ID | drug / compound 2  (name OR n.a.) | administration route  (s.c. OR applied topically OR n.a.) | dosage  (mg/kg) OR n.r. OR n.a. | injection volume  (ml/animal OR n.r. OR n.a.) | timepoint of first administration  (pre-, intra-, post surgically OR n.a.) | if reported: immediately before surgery OR immediately after surgery OR n.r. OR n.a. | administered how many times in total?  (number OR n.a.) | if so: administration interval  (h post first administration OR n.r. OR n.a.) | if reported: drug concentration  (mg/ml OR n.r. OR n.a.) | analgesia NSAID administered? (yes / no OR n.a.) | drug / compound 1 (name OR n.a.) | administration route  (s.c. OR i.m. OR i.p. OR per os OR n.a.) | dosage  (mg/kg) OR n.r. OR n.a. | timepoint of first administration  (pre-, intra-, postsurgically OR n.a.) | if reported: immediately before surgery OR immediately after surgery OR n.r. OR n.a. |
| --- | --- | --- | --- | --- | --- | --- | --- | --- | --- | --- | --- | --- | --- | --- | --- |
| Bartolomucci et al. | n.a. | n.a. | n.a. | n.a. | n.a. | n.a. | n.a. | n.a. | n.a. | no | n.a. | n.a. | n.a. | n.a. | n.a. |
| Behrend et al. | n.a. | n.a. | n.a. | n.a. | n.a. | n.a. | n.a. | n.a. | n.a. | no | n.a. | n.a. | n.a. | n.a. | n.a. |
| Biella et al. | n.a. | n.a. | n.a. | n.a. | n.a. | n.a. | n.a. | n.a. | n.a. | no | n.a. | n.a. | n.a. | n.a. | n.a. |
| Boni et al. | n.a. | n.a. | n.a. | n.a. | n.a. | n.a. | n.a. | n.a. | n.a. | no | n.a. | n.a. | n.a. | n.a. | n.a. |
| Bramlett et al. | n.a. | n.a. | n.a. | n.a. | n.a. | n.a. | n.a. | n.a. | n.a. | no | n.a. | n.a. | n.a. | n.a. | n.a. |
| Byun et al. | n.a. | n.a. | n.a. | n.a. | n.a. | n.a. | n.a. | n.a. | n.a. | no | n.a. | n.a. | n.a. | n.a. | n.a. |
| Caltana et al. | n.a. | n.a. | n.a. | n.a. | n.a. | n.a. | n.a. | n.a. | n.a. | no | n.a. | n.a. | n.a. | n.a. | n.a. |
| Carcak et al. | n.a. | n.a. | n.a. | n.a. | n.a. | n.a. | n.a. | n.a. | n.a. | no | n.a. | n.a. | n.a. | n.a. | n.a. |
| Cemil et al. | n.a. | n.a. | n.a. | n.a. | n.a. | n.a. | n.a. | n.a. | n.a. | no | n.a. | n.a. | n.a. | n.a. | n.a. |
| Chen et al. D | n.a. | n.a. | n.a. | n.a. | n.a. | n.a. | n.a. | n.a. | n.a. | no | n.a. | n.a. | n.a. | n.a. | n.a. |
| Cifani et al. | n.a. | n.a. | n.a. | n.a. | n.a. | n.a. | n.a. | n.a. | n.a. | no | n.a. | n.a. | n.a. | n.a. | n.a. |
| Cunningham et al. | n.a. | n.a. | n.a. | n.a. | n.a. | n.a. | n.a. | n.a. | n.a. | no | n.a. | n.a. | n.a. | n.a. | n.a. |
| Datta et al. | n.a. | n.a. | n.a. | n.a. | n.a. | n.a. | n.a. | n.a. | n.a. | no | n.a. | n.a. | n.a. | n.a. | n.a. |
| Diesch et al. | n.a. | n.a. | n.a. | n.a. | n.a. | n.a. | n.a. | n.a. | n.a. | no | n.a. | n.a. | n.a. | n.a. | n.a. |
| Diguet et al. | n.a. | n.a. | n.a. | n.a. | n.a. | n.a. | n.a. | n.a. | n.a. | no | n.a. | n.a. | n.a. | n.a. | n.a. |
| Ding et al. | n.a. | n.a. | n.a. | n.a. | n.a. | n.a. | n.a. | n.a. | n.a. | no | n.a. | n.a. | n.a. | n.a. | n.a. |
| Doan et al. | n.a. | n.a. | n.a. | n.a. | n.a. | n.a. | n.a. | n.a. | n.a. | no | n.a. | n.a. | n.a. | n.a. | n.a. |
| Doretto et al. | n.a. | n.a. | n.a. | n.a. | n.a. | n.a. | n.a. | n.a. | n.a. | no | n.a. | n.a. | n.a. | n.a. | n.a. |
| Dux et al. | n.a. | n.a. | n.a. | n.a. | n.a. | n.a. | n.a. | n.a. | n.a. | no | n.a. | n.a. | n.a. | n.a. | n.a. |
| Echegoyen et al. | n.a. | n.a. | n.a. | n.a. | n.a. | n.a. | n.a. | n.a. | n.a. | no | n.a. | n.a. | n.a. | n.a. | n.a. |
| Ehrlichman et al. | n.a. | n.a. | n.a. | n.a. | n.a. | n.a. | n.a. | n.a. | n.a. | no | n.a. | n.a. | n.a. | n.a. | n.a. |
| Etholm et al. | n.a. | n.a. | n.a. | n.a. | n.a. | n.a. | n.a. | n.a. | n.a. | no | n.a. | n.a. | n.a. | n.a. | n.a. |
| Farias et al. | n.a. | n.a. | n.a. | n.a. | n.a. | n.a. | n.a. | n.a. | n.a. | no | n.a. | n.a. | n.a. | n.a. | n.a. |
| Foti et al. | n.a. | n.a. | n.a. | n.a. | n.a. | n.a. | n.a. | n.a. | n.a. | no | n.a. | n.a. | n.a. | n.a. | n.a. |
| Francois et al. | n.a. | n.a. | n.a. | n.a. | n.a. | n.a. | n.a. | n.a. | n.a. | no | n.a. | n.a. | n.a. | n.a. | n.a. |
| Francois et al. | n.a. | n.a. | n.a. | n.a. | n.a. | n.a. | n.a. | n.a. | n.a. | no | n.a. | n.a. | n.a. | n.a. | n.a. |
| Fritsch et al. | n.a. | n.a. | n.a. | n.a. | n.a. | n.a. | n.a. | n.a. | n.a. | no | n.a. | n.a. | n.a. | n.a. | n.a. |
| Good et al. | n.a. | n.a. | n.a. | n.a. | n.a. | n.a. | n.a. | n.a. | n.a. | no | n.a. | n.a. | n.a. | n.a. | n.a. |
| Griesbach et al. | n.a. | n.a. | n.a. | n.a. | n.a. | n.a. | n.a. | n.a. | n.a. | no | n.a. | n.a. | n.a. | n.a. | n.a. |
| Guidine et al. | n.a. | n.a. | n.a. | n.a. | n.a. | n.a. | n.a. | n.a. | n.a. | no | n.a. | n.a. | n.a. | n.a. | n.a. |
| Gurevicius et al. | n.a. | n.a. | n.a. | n.a. | n.a. | n.a. | n.a. | n.a. | n.a. | yes | carprofen | i.p. | 5 | postsurgically | immediately after surgery |
| Hart et al. | n.a. | n.a. | n.a. | n.a. | n.a. | n.a. | n.a. | n.a. | n.a. | no | n.a. | n.a. | n.a. | n.a. | n.a. |
| Harvey et al. | n.a. | n.a. | n.a. | n.a. | n.a. | n.a. | n.a. | n.a. | n.a. | no | n.a. | n.a. | n.a. | n.a. | n.a. |
| Hernandez-Gonzalez et al. | n.a. | n.a. | n.a. | n.a. | n.a. | n.a. | n.a. | n.a. | n.a. | no | n.a. | n.a. | n.a. | n.a. | n.a. |
| Ho et al. | n.a. | n.a. | n.a. | n.a. | n.a. | n.a. | n.a. | n.a. | n.a. | no | n.a. | n.a. | n.a. | n.a. | n.a. |
| Holtmaat et al. | n.a. | n.a. | n.a. | n.a. | n.a. | n.a. | n.a. | n.a. | n.a. | no | n.a. | n.a. | n.a. | n.a. | n.a. |
| Hrncic et al. | n.a. | n.a. | n.a. | n.a. | n.a. | n.a. | n.a. | n.a. | n.a. | no | n.a. | n.a. | n.a. | n.a. | n.a. |
| Huguet et al. | n.a. | n.a. | n.a. | n.a. | n.a. | n.a. | n.a. | n.a. | n.a. | no | n.a. | n.a. | n.a. | n.a. | n.a. |
| Ishida et al. | n.a. | n.a. | n.a. | n.a. | n.a. | n.a. | n.a. | n.a. | n.a. | no | n.a. | n.a. | n.a. | n.a. | n.a. |
| Ito et al. | n.a. | n.a. | n.a. | n.a. | n.a. | n.a. | n.a. | n.a. | n.a. | no | n.a. | n.a. | n.a. | n.a. | n.a. |
| Itoh et al. B | n.a. | n.a. | n.a. | n.a. | n.a. | n.a. | n.a. | n.a. | n.a. | no | n.a. | n.a. | n.a. | n.a. | n.a. |
| Itoh et al. A | n.a. | n.a. | n.a. | n.a. | n.a. | n.a. | n.a. | n.a. | n.a. | no | n.a. | n.a. | n.a. | n.a. | n.a. |
| Jafri et al. | n.a. | n.a. | n.a. | n.a. | n.a. | n.a. | n.a. | n.a. | n.a. | no | n.a. | n.a. | n.a. | n.a. | n.a. |
| Kalauzi et al. | n.a. | n.a. | n.a. | n.a. | n.a. | n.a. | n.a. | n.a. | n.a. | no | n.a. | n.a. | n.a. | n.a. | n.a. |
| Katz et al. | n.a. | n.a. | n.a. | n.a. | n.a. | n.a. | n.a. | n.a. | n.a. | no | n.a. | n.a. | n.a. | n.a. | n.a. |
| Kim et al. A | n.a. | n.a. | n.a. | n.a. | n.a. | n.a. | n.a. | n.a. | n.a. | no | n.a. | n.a. | n.a. | n.a. | n.a. |
| Lackovic et al. | n.a. | n.a. | n.a. | n.a. | n.a. | n.a. | n.a. | n.a. | n.a. | no | n.a. | n.a. | n.a. | n.a. | n.a. |
| Lee et al. B | n.a. | n.a. | n.a. | n.a. | n.a. | n.a. | n.a. | n.a. | n.a. | no | n.a. | n.a. | n.a. | n.a. | n.a. |
| Lee et al. A | n.a. | n.a. | n.a. | n.a. | n.a. | n.a. | n.a. | n.a. | n.a. | no | n.a. | n.a. | n.a. | n.a. | n.a. |
| Li et al. C | n.a. | n.a. | n.a. | n.a. | n.a. | n.a. | n.a. | n.a. | n.a. | no | n.a. | n.a. | n.a. | n.a. | n.a. |
| Liu et al. | n.a. | n.a. | n.a. | n.a. | n.a. | n.a. | n.a. | n.a. | n.a. | no | n.a. | n.a. | n.a. | n.a. | n.a. |
| Lopez-Martin et al. | n.a. | n.a. | n.a. | n.a. | n.a. | n.a. | n.a. | n.a. | n.a. | no | n.a. | n.a. | n.a. | n.a. | n.a. |
| Lu et al. | n.a. | n.a. | n.a. | n.a. | n.a. | n.a. | n.a. | n.a. | n.a. | no | n.a. | n.a. | n.a. | n.a. | n.a. |
| Lundblad et al. | n.a. | n.a. | n.a. | n.a. | n.a. | n.a. | n.a. | n.a. | n.a. | no | n.a. | n.a. | n.a. | n.a. | n.a. |
| Magloire et al. | n.a. | n.a. | n.a. | n.a. | n.a. | n.a. | n.a. | n.a. | n.a. | yes | acetaminophen | per os | n.r. | postsurgically | n.a. |
| Mark et al. | n.a. | n.a. | n.a. | n.a. | n.a. | n.a. | n.a. | n.a. | n.a. | no | n.a. | n.a. | n.a. | n.a. | n.a. |
| McCracken et al. | n.a. | n.a. | n.a. | n.a. | n.a. | n.a. | n.a. | n.a. | n.a. | no | n.a. | n.a. | n.a. | n.a. | n.a. |
| Meeren et al. | n.a. | n.a. | n.a. | n.a. | n.a. | n.a. | n.a. | n.a. | n.a. | no | n.a. | n.a. | n.a. | n.a. | n.a. |
| Merkler et al. | n.a. | n.a. | n.a. | n.a. | n.a. | n.a. | n.a. | n.a. | n.a. | no | n.a. | n.a. | n.a. | n.a. | n.a. |
| Merkler et al. | n.a. | n.a. | n.a. | n.a. | n.a. | n.a. | n.a. | n.a. | n.a. | no | n.a. | n.a. | n.a. | n.a. | n.a. |
| Mian et al. | n.a. | n.a. | n.a. | n.a. | n.a. | n.a. | n.a. | n.a. | n.a. | no | n.a. | n.a. | n.a. | n.a. | n.a. |
| Mohammadi et al. | n.a. | n.a. | n.a. | n.a. | n.a. | n.a. | n.a. | n.a. | n.a. | no | n.a. | n.a. | n.a. | n.a. | n.a. |
| Mollazaedh et al. | n.a. | n.a. | n.a. | n.a. | n.a. | n.a. | n.a. | n.a. | n.a. | no | n.a. | n.a. | n.a. | n.a. | n.a. |
| Mukherjee et al. | n.a. | n.a. | n.a. | n.a. | n.a. | n.a. | n.a. | n.a. | n.a. | no | n.a. | n.a. | n.a. | n.a. | n.a. |
| Nehlig et al. | n.a. | n.a. | n.a. | n.a. | n.a. | n.a. | n.a. | n.a. | n.a. | no | n.a. | n.a. | n.a. | n.a. | n.a. |
| Nuki et al. | n.a. | n.a. | n.a. | n.a. | n.a. | n.a. | n.a. | n.a. | n.a. | no | n.a. | n.a. | n.a. | n.a. | n.a. |
| Onyszchuk et al. | n.a. | n.a. | n.a. | n.a. | n.a. | n.a. | n.a. | n.a. | n.a. | no | n.a. | n.a. | n.a. | n.a. | n.a. |
| Oshima et al. | n.a. | n.a. | n.a. | n.a. | n.a. | n.a. | n.a. | n.a. | n.a. | no | n.a. | n.a. | n.a. | n.a. | n.a. |
| Potts et al. | n.a. | n.a. | n.a. | n.a. | n.a. | n.a. | n.a. | n.a. | n.a. | no | n.a. | n.a. | n.a. | n.a. | n.a. |
| Qing et al. | n.a. | n.a. | n.a. | n.a. | n.a. | n.a. | n.a. | n.a. | n.a. | no | n.a. | n.a. | n.a. | n.a. | n.a. |
| Rahim et al. | n.a. | n.a. | n.a. | n.a. | n.a. | n.a. | n.a. | n.a. | n.a. | no | n.a. | n.a. | n.a. | n.a. | n.a. |
| Rimoli et al. | n.a. | n.a. | n.a. | n.a. | n.a. | n.a. | n.a. | n.a. | n.a. | no | n.a. | n.a. | n.a. | n.a. | n.a. |
| Roiko et al. | n.a. | n.a. | n.a. | n.a. | n.a. | n.a. | n.a. | n.a. | n.a. | no | n.a. | n.a. | n.a. | n.a. | n.a. |
| Rudnick et al. | n.a. | n.a. | n.a. | n.a. | n.a. | n.a. | n.a. | n.a. | n.a. | no | n.a. | n.a. | n.a. | n.a. | n.a. |
| Sahin et al. | n.a. | n.a. | n.a. | n.a. | n.a. | n.a. | n.a. | n.a. | n.a. | no | n.a. | n.a. | n.a. | n.a. | n.a. |
| Samnick et al. | n.a. | n.a. | n.a. | n.a. | n.a. | n.a. | n.a. | n.a. | n.a. | no | n.a. | n.a. | n.a. | n.a. | n.a. |
| Sasaki et al. | n.a. | n.a. | n.a. | n.a. | n.a. | n.a. | n.a. | n.a. | n.a. | no | n.a. | n.a. | n.a. | n.a. | n.a. |
| Schei et al. | n.a. | n.a. | n.a. | n.a. | n.a. | n.a. | n.a. | n.a. | n.a. | yes | flunixin | s.c. | 1,1 | intrasurgically | n.a. |
| Schmid et al. | n.a. | n.a. | n.a. | n.a. | n.a. | n.a. | n.a. | n.a. | n.a. | no | n.a. | n.a. | n.a. | n.a. | n.a. |
| Sekiya et al. | n.a. | n.a. | n.a. | n.a. | n.a. | n.a. | n.a. | n.a. | n.a. | no | n.a. | n.a. | n.a. | n.a. | n.a. |
| Sher et al. | n.a. | n.a. | n.a. | n.a. | n.a. | n.a. | n.a. | n.a. | n.a. | no | n.a. | n.a. | n.a. | n.a. | n.a. |
| Shultz et al. | n.a. | n.a. | n.a. | n.a. | n.a. | n.a. | n.a. | n.a. | n.a. | yes | ketoprofen | s.c. | n.r. | postsurgically | immediately after surgery |
| Silvani et al. | n.a. | n.a. | n.a. | n.a. | n.a. | n.a. | n.a. | n.a. | n.a. | no | n.a. | n.a. | n.a. | n.a. | n.a. |
| Sinton et al. | n.a. | n.a. | n.a. | n.a. | n.a. | n.a. | n.a. | n.a. | n.a. | no | n.a. | n.a. | n.a. | n.a. | n.a. |
| Song et al. | n.a. | n.a. | n.a. | n.a. | n.a. | n.a. | n.a. | n.a. | n.a. | no | n.a. | n.a. | n.a. | n.a. | n.a. |
| Takahashi et al. | n.a. | n.a. | n.a. | n.a. | n.a. | n.a. | n.a. | n.a. | n.a. | no | n.a. | n.a. | n.a. | n.a. | n.a. |
| Takahashi et al. | n.a. | n.a. | n.a. | n.a. | n.a. | n.a. | n.a. | n.a. | n.a. | no | n.a. | n.a. | n.a. | n.a. | n.a. |
| Tanida et al. | n.a. | n.a. | n.a. | n.a. | n.a. | n.a. | n.a. | n.a. | n.a. | no | n.a. | n.a. | n.a. | n.a. | n.a. |
| Tchekalarova et al. | n.a. | n.a. | n.a. | n.a. | n.a. | n.a. | n.a. | n.a. | n.a. | no | n.a. | n.a. | n.a. | n.a. | n.a. |
| Thomas et al. | n.a. | n.a. | n.a. | n.a. | n.a. | n.a. | n.a. | n.a. | n.a. | no | n.a. | n.a. | n.a. | n.a. | n.a. |
| Topchiy et al. | n.a. | n.a. | n.a. | n.a. | n.a. | n.a. | n.a. | n.a. | n.a. | yes | flunixin | n.r. | 1,1 | postsurgically | immediately after surgery |
| Touzani et al. | n.a. | n.a. | n.a. | n.a. | n.a. | n.a. | n.a. | n.a. | n.a. | no | n.a. | n.a. | n.a. | n.a. | n.a. |
| Tsanov et al. | n.a. | n.a. | n.a. | n.a. | n.a. | n.a. | n.a. | n.a. | n.a. | no | n.a. | n.a. | n.a. | n.a. | n.a. |
| Wagner et al. | n.a. | n.a. | n.a. | n.a. | n.a. | n.a. | n.a. | n.a. | n.a. | no | n.a. | n.a. | n.a. | n.a. | n.a. |
| Wan et al. | n.a. | n.a. | n.a. | n.a. | n.a. | n.a. | n.a. | n.a. | n.a. | no | n.a. | n.a. | n.a. | n.a. | n.a. |
| Wigren et al. | n.a. | n.a. | n.a. | n.a. | n.a. | n.a. | n.a. | n.a. | n.a. | no | n.a. | n.a. | n.a. | n.a. | n.a. |
| Worthen et al. | n.a. | n.a. | n.a. | n.a. | n.a. | n.a. | n.a. | n.a. | n.a. | no | n.a. | n.a. | n.a. | n.a. | n.a. |
| Xue et al. | n.a. | n.a. | n.a. | n.a. | n.a. | n.a. | n.a. | n.a. | n.a. | no | n.a. | n.a. | n.a. | n.a. | n.a. |
| Yoon et al. | n.a. | n.a. | n.a. | n.a. | n.a. | n.a. | n.a. | n.a. | n.a. | no | n.a. | n.a. | n.a. | n.a. | n.a. |
| Young et al. | n.a. | n.a. | n.a. | n.a. | n.a. | n.a. | n.a. | n.a. | n.a. | no | n.a. | n.a. | n.a. | n.a. | n.a. |
| Yu et al. | n.a. | n.a. | n.a. | n.a. | n.a. | n.a. | n.a. | n.a. | n.a. | no | n.a. | n.a. | n.a. | n.a. | n.a. |
| Yurek et al. | n.a. | n.a. | n.a. | n.a. | n.a. | n.a. | n.a. | n.a. | n.a. | no | n.a. | n.a. | n.a. | n.a. | n.a. |
| Zeng et al. | n.a. | n.a. | n.a. | n.a. | n.a. | n.a. | n.a. | n.a. | n.a. | no | n.a. | n.a. | n.a. | n.a. | n.a. |

| study ID | administered how many times in total?  (number OR n.a. OR n.r.) | if reported: administration interval  (morning after surgery OR 24h post surgery OR n.r. OR n.a.) | if reported: administration interval  (h post first administration OR n.r. OR n.a.) | if reported:  pharmaceutical formulation (e.g. solution OR n.r. OR n.a.) | drug / compound 2 (name OR n.a.) | administration route  (s.c. OR i.m. OR i.p. OR per os OR n.a.) | dosage  (mg/kg) OR n.r. OR n.a. | timepoint of first administration  (pre-, intra-, postsurgically OR n.a.) | if reported: immediately before surgery OR immediately after surgery OR n.r. OR n.a. | administered how many times in total?  (number OR n.a. OR n.r.) | if reported: administration interval  (morning after surgery OR 24h post surgery OR n.r. OR n.a.) | if reported: administration interval  (h post first administration OR n.r. OR n.a.) | if reported:  pharmaceutical formulation (e.g. solution OR n.r. OR n.a.) |
| --- | --- | --- | --- | --- | --- | --- | --- | --- | --- | --- | --- | --- | --- |
| Bartolomucci et al. | n.a. | n.a. | n.a. | n.a. | n.a. | n.a. | n.a. | n.a. | n.a. | n.a. | n.a. | n.a. | n.a. |
| Behrend et al. | n.a. | n.a. | n.a. | n.a. | n.a. | n.a. | n.a. | n.a. | n.a. | n.a. | n.a. | n.a. | n.a. |
| Biella et al. | n.a. | n.a. | n.a. | n.a. | n.a. | n.a. | n.a. | n.a. | n.a. | n.a. | n.a. | n.a. | n.a. |
| Boni et al. | n.a. | n.a. | n.a. | n.a. | n.a. | n.a. | n.a. | n.a. | n.a. | n.a. | n.a. | n.a. | n.a. |
| Bramlett et al. | n.a. | n.a. | n.a. | n.a. | n.a. | n.a. | n.a. | n.a. | n.a. | n.a. | n.a. | n.a. | n.a. |
| Byun et al. | n.a. | n.a. | n.a. | n.a. | n.a. | n.a. | n.a. | n.a. | n.a. | n.a. | n.a. | n.a. | n.a. |
| Caltana et al. | n.a. | n.a. | n.a. | n.a. | n.a. | n.a. | n.a. | n.a. | n.a. | n.a. | n.a. | n.a. | n.a. |
| Carcak et al. | n.a. | n.a. | n.a. | n.a. | n.a. | n.a. | n.a. | n.a. | n.a. | n.a. | n.a. | n.a. | n.a. |
| Cemil et al. | n.a. | n.a. | n.a. | n.a. | n.a. | n.a. | n.a. | n.a. | n.a. | n.a. | n.a. | n.a. | n.a. |
| Chen et al. D | n.a. | n.a. | n.a. | n.a. | n.a. | n.a. | n.a. | n.a. | n.a. | n.a. | n.a. | n.a. | n.a. |
| Cifani et al. | n.a. | n.a. | n.a. | n.a. | n.a. | n.a. | n.a. | n.a. | n.a. | n.a. | n.a. | n.a. | n.a. |
| Cunningham et al. | n.a. | n.a. | n.a. | n.a. | n.a. | n.a. | n.a. | n.a. | n.a. | n.a. | n.a. | n.a. | n.a. |
| Datta et al. | n.a. | n.a. | n.a. | n.a. | n.a. | n.a. | n.a. | n.a. | n.a. | n.a. | n.a. | n.a. | n.a. |
| Diesch et al. | n.a. | n.a. | n.a. | n.a. | n.a. | n.a. | n.a. | n.a. | n.a. | n.a. | n.a. | n.a. | n.a. |
| Diguet et al. | n.a. | n.a. | n.a. | n.a. | n.a. | n.a. | n.a. | n.a. | n.a. | n.a. | n.a. | n.a. | n.a. |
| Ding et al. | n.a. | n.a. | n.a. | n.a. | n.a. | n.a. | n.a. | n.a. | n.a. | n.a. | n.a. | n.a. | n.a. |
| Doan et al. | n.a. | n.a. | n.a. | n.a. | n.a. | n.a. | n.a. | n.a. | n.a. | n.a. | n.a. | n.a. | n.a. |
| Doretto et al. | n.a. | n.a. | n.a. | n.a. | n.a. | n.a. | n.a. | n.a. | n.a. | n.a. | n.a. | n.a. | n.a. |
| Dux et al. | n.a. | n.a. | n.a. | n.a. | n.a. | n.a. | n.a. | n.a. | n.a. | n.a. | n.a. | n.a. | n.a. |
| Echegoyen et al. | n.a. | n.a. | n.a. | n.a. | n.a. | n.a. | n.a. | n.a. | n.a. | n.a. | n.a. | n.a. | n.a. |
| Ehrlichman et al. | n.a. | n.a. | n.a. | n.a. | n.a. | n.a. | n.a. | n.a. | n.a. | n.a. | n.a. | n.a. | n.a. |
| Etholm et al. | n.a. | n.a. | n.a. | n.a. | n.a. | n.a. | n.a. | n.a. | n.a. | n.a. | n.a. | n.a. | n.a. |
| Farias et al. | n.a. | n.a. | n.a. | n.a. | n.a. | n.a. | n.a. | n.a. | n.a. | n.a. | n.a. | n.a. | n.a. |
| Foti et al. | n.a. | n.a. | n.a. | n.a. | n.a. | n.a. | n.a. | n.a. | n.a. | n.a. | n.a. | n.a. | n.a. |
| Francois et al. | n.a. | n.a. | n.a. | n.a. | n.a. | n.a. | n.a. | n.a. | n.a. | n.a. | n.a. | n.a. | n.a. |
| Francois et al. | n.a. | n.a. | n.a. | n.a. | n.a. | n.a. | n.a. | n.a. | n.a. | n.a. | n.a. | n.a. | n.a. |
| Fritsch et al. | n.a. | n.a. | n.a. | n.a. | n.a. | n.a. | n.a. | n.a. | n.a. | n.a. | n.a. | n.a. | n.a. |
| Good et al. | n.a. | n.a. | n.a. | n.a. | n.a. | n.a. | n.a. | n.a. | n.a. | n.a. | n.a. | n.a. | n.a. |
| Griesbach et al. | n.a. | n.a. | n.a. | n.a. | n.a. | n.a. | n.a. | n.a. | n.a. | n.a. | n.a. | n.a. | n.a. |
| Guidine et al. | n.a. | n.a. | n.a. | n.a. | n.a. | n.a. | n.a. | n.a. | n.a. | n.a. | n.a. | n.a. | n.a. |
| Gurevicius et al. | 1 | n.a. | n.a. | n.r. | n.a. | n.a. | n.a. | n.a. | n.a. | n.a. | n.a. | n.a. | n.a. |
| Hart et al. | n.a. | n.a. | n.a. | n.a. | n.a. | n.a. | n.a. | n.a. | n.a. | n.a. | n.a. | n.a. | n.a. |
| Harvey et al. | n.a. | n.a. | n.a. | n.a. | n.a. | n.a. | n.a. | n.a. | n.a. | n.a. | n.a. | n.a. | n.a. |
| Hernandez-Gonzalez et al. | n.a. | n.a. | n.a. | n.a. | n.a. | n.a. | n.a. | n.a. | n.a. | n.a. | n.a. | n.a. | n.a. |
| Ho et al. | n.a. | n.a. | n.a. | n.a. | n.a. | n.a. | n.a. | n.a. | n.a. | n.a. | n.a. | n.a. | n.a. |
| Holtmaat et al. | n.a. | n.a. | n.a. | n.a. | n.a. | n.a. | n.a. | n.a. | n.a. | n.a. | n.a. | n.a. | n.a. |
| Hrncic et al. | n.a. | n.a. | n.a. | n.a. | n.a. | n.a. | n.a. | n.a. | n.a. | n.a. | n.a. | n.a. | n.a. |
| Huguet et al. | n.a. | n.a. | n.a. | n.a. | n.a. | n.a. | n.a. | n.a. | n.a. | n.a. | n.a. | n.a. | n.a. |
| Ishida et al. | n.a. | n.a. | n.a. | n.a. | n.a. | n.a. | n.a. | n.a. | n.a. | n.a. | n.a. | n.a. | n.a. |
| Ito et al. | n.a. | n.a. | n.a. | n.a. | n.a. | n.a. | n.a. | n.a. | n.a. | n.a. | n.a. | n.a. | n.a. |
| Itoh et al. B | n.a. | n.a. | n.a. | n.a. | n.a. | n.a. | n.a. | n.a. | n.a. | n.a. | n.a. | n.a. | n.a. |
| Itoh et al. A | n.a. | n.a. | n.a. | n.a. | n.a. | n.a. | n.a. | n.a. | n.a. | n.a. | n.a. | n.a. | n.a. |
| Jafri et al. | n.a. | n.a. | n.a. | n.a. | n.a. | n.a. | n.a. | n.a. | n.a. | n.a. | n.a. | n.a. | n.a. |
| Kalauzi et al. | n.a. | n.a. | n.a. | n.a. | n.a. | n.a. | n.a. | n.a. | n.a. | n.a. | n.a. | n.a. | n.a. |
| Katz et al. | n.a. | n.a. | n.a. | n.a. | n.a. | n.a. | n.a. | n.a. | n.a. | n.a. | n.a. | n.a. | n.a. |
| Kim et al. A | n.a. | n.a. | n.a. | n.a. | n.a. | n.a. | n.a. | n.a. | n.a. | n.a. | n.a. | n.a. | n.a. |
| Lackovic et al. | n.a. | n.a. | n.a. | n.a. | n.a. | n.a. | n.a. | n.a. | n.a. | n.a. | n.a. | n.a. | n.a. |
| Lee et al. B | n.a. | n.a. | n.a. | n.a. | n.a. | n.a. | n.a. | n.a. | n.a. | n.a. | n.a. | n.a. | n.a. |
| Lee et al. A | n.a. | n.a. | n.a. | n.a. | n.a. | n.a. | n.a. | n.a. | n.a. | n.a. | n.a. | n.a. | n.a. |
| Li et al. C | n.a. | n.a. | n.a. | n.a. | n.a. | n.a. | n.a. | n.a. | n.a. | n.a. | n.a. | n.a. | n.a. |
| Liu et al. | n.a. | n.a. | n.a. | n.a. | n.a. | n.a. | n.a. | n.a. | n.a. | n.a. | n.a. | n.a. | n.a. |
| Lopez-Martin et al. | n.a. | n.a. | n.a. | n.a. | n.a. | n.a. | n.a. | n.a. | n.a. | n.a. | n.a. | n.a. | n.a. |
| Lu et al. | n.a. | n.a. | n.a. | n.a. | n.a. | n.a. | n.a. | n.a. | n.a. | n.a. | n.a. | n.a. | n.a. |
| Lundblad et al. | n.a. | n.a. | n.a. | n.a. | n.a. | n.a. | n.a. | n.a. | n.a. | n.a. | n.a. | n.a. | n.a. |
| Magloire et al. | n.a. | n.a. | n.a. | solution | n.a. | n.a. | n.a. | n.a. | n.a. | n.a. | n.a. | n.a. | n.a. |
| Mark et al. | n.a. | n.a. | n.a. | n.a. | n.a. | n.a. | n.a. | n.a. | n.a. | n.a. | n.a. | n.a. | n.a. |
| McCracken et al. | n.a. | n.a. | n.a. | n.a. | n.a. | n.a. | n.a. | n.a. | n.a. | n.a. | n.a. | n.a. | n.a. |
| Meeren et al. | n.a. | n.a. | n.a. | n.a. | n.a. | n.a. | n.a. | n.a. | n.a. | n.a. | n.a. | n.a. | n.a. |
| Merkler et al. | n.a. | n.a. | n.a. | n.a. | n.a. | n.a. | n.a. | n.a. | n.a. | n.a. | n.a. | n.a. | n.a. |
| Merkler et al. | n.a. | n.a. | n.a. | n.a. | n.a. | n.a. | n.a. | n.a. | n.a. | n.a. | n.a. | n.a. | n.a. |
| Mian et al. | n.a. | n.a. | n.a. | n.a. | n.a. | n.a. | n.a. | n.a. | n.a. | n.a. | n.a. | n.a. | n.a. |
| Mohammadi et al. | n.a. | n.a. | n.a. | n.a. | n.a. | n.a. | n.a. | n.a. | n.a. | n.a. | n.a. | n.a. | n.a. |
| Mollazaedh et al. | n.a. | n.a. | n.a. | n.a. | n.a. | n.a. | n.a. | n.a. | n.a. | n.a. | n.a. | n.a. | n.a. |
| Mukherjee et al. | n.a. | n.a. | n.a. | n.a. | n.a. | n.a. | n.a. | n.a. | n.a. | n.a. | n.a. | n.a. | n.a. |
| Nehlig et al. | n.a. | n.a. | n.a. | n.a. | n.a. | n.a. | n.a. | n.a. | n.a. | n.a. | n.a. | n.a. | n.a. |
| Nuki et al. | n.a. | n.a. | n.a. | n.a. | n.a. | n.a. | n.a. | n.a. | n.a. | n.a. | n.a. | n.a. | n.a. |
| Onyszchuk et al. | n.a. | n.a. | n.a. | n.a. | n.a. | n.a. | n.a. | n.a. | n.a. | n.a. | n.a. | n.a. | n.a. |
| Oshima et al. | n.a. | n.a. | n.a. | n.a. | n.a. | n.a. | n.a. | n.a. | n.a. | n.a. | n.a. | n.a. | n.a. |
| Potts et al. | n.a. | n.a. | n.a. | n.a. | n.a. | n.a. | n.a. | n.a. | n.a. | n.a. | n.a. | n.a. | n.a. |
| Qing et al. | n.a. | n.a. | n.a. | n.a. | n.a. | n.a. | n.a. | n.a. | n.a. | n.a. | n.a. | n.a. | n.a. |
| Rahim et al. | n.a. | n.a. | n.a. | n.a. | n.a. | n.a. | n.a. | n.a. | n.a. | n.a. | n.a. | n.a. | n.a. |
| Rimoli et al. | n.a. | n.a. | n.a. | n.a. | n.a. | n.a. | n.a. | n.a. | n.a. | n.a. | n.a. | n.a. | n.a. |
| Roiko et al. | n.a. | n.a. | n.a. | n.a. | n.a. | n.a. | n.a. | n.a. | n.a. | n.a. | n.a. | n.a. | n.a. |
| Rudnick et al. | n.a. | n.a. | n.a. | n.a. | n.a. | n.a. | n.a. | n.a. | n.a. | n.a. | n.a. | n.a. | n.a. |
| Sahin et al. | n.a. | n.a. | n.a. | n.a. | n.a. | n.a. | n.a. | n.a. | n.a. | n.a. | n.a. | n.a. | n.a. |
| Samnick et al. | n.a. | n.a. | n.a. | n.a. | n.a. | n.a. | n.a. | n.a. | n.a. | n.a. | n.a. | n.a. | n.a. |
| Sasaki et al. | n.a. | n.a. | n.a. | n.a. | n.a. | n.a. | n.a. | n.a. | n.a. | n.a. | n.a. | n.a. | n.a. |
| Schei et al. | 1 | n.a. | n.a. | n.r. | n.a. | n.a. | n.a. | n.a. | n.a. | n.a. | n.a. | n.a. | n.a. |
| Schmid et al. | n.a. | n.a. | n.a. | n.a. | n.a. | n.a. | n.a. | n.a. | n.a. | n.a. | n.a. | n.a. | n.a. |
| Sekiya et al. | n.a. | n.a. | n.a. | n.a. | n.a. | n.a. | n.a. | n.a. | n.a. | n.a. | n.a. | n.a. | n.a. |
| Sher et al. | n.a. | n.a. | n.a. | n.a. | n.a. | n.a. | n.a. | n.a. | n.a. | n.a. | n.a. | n.a. | n.a. |
| Shultz et al. | 1 | n.a. | n.a. | n.r. | n.a. | n.a. | n.a. | n.a. | n.a. | n.a. | n.a. | n.a. | n.a. |
| Silvani et al. | n.a. | n.a. | n.a. | n.a. | n.a. | n.a. | n.a. | n.a. | n.a. | n.a. | n.a. | n.a. | n.a. |
| Sinton et al. | n.a. | n.a. | n.a. | n.a. | n.a. | n.a. | n.a. | n.a. | n.a. | n.a. | n.a. | n.a. | n.a. |
| Song et al. | n.a. | n.a. | n.a. | n.a. | n.a. | n.a. | n.a. | n.a. | n.a. | n.a. | n.a. | n.a. | n.a. |
| Takahashi et al. | n.a. | n.a. | n.a. | n.a. | n.a. | n.a. | n.a. | n.a. | n.a. | n.a. | n.a. | n.a. | n.a. |
| Takahashi et al. | n.a. | n.a. | n.a. | n.a. | n.a. | n.a. | n.a. | n.a. | n.a. | n.a. | n.a. | n.a. | n.a. |
| Tanida et al. | n.a. | n.a. | n.a. | n.a. | n.a. | n.a. | n.a. | n.a. | n.a. | n.a. | n.a. | n.a. | n.a. |
| Tchekalarova et al. | n.a. | n.a. | n.a. | n.a. | n.a. | n.a. | n.a. | n.a. | n.a. | n.a. | n.a. | n.a. | n.a. |
| Thomas et al. | n.a. | n.a. | n.a. | n.a. | n.a. | n.a. | n.a. | n.a. | n.a. | n.a. | n.a. | n.a. | n.a. |
| Topchiy et al. | 3 | n.r. | 24 | n.r. | n.a. | n.a. | n.a. | n.a. | n.a. | n.a. | n.a. | n.a. | n.a. |
| Touzani et al. | n.a. | n.a. | n.a. | n.a. | n.a. | n.a. | n.a. | n.a. | n.a. | n.a. | n.a. | n.a. | n.a. |
| Tsanov et al. | n.a. | n.a. | n.a. | n.a. | n.a. | n.a. | n.a. | n.a. | n.a. | n.a. | n.a. | n.a. | n.a. |
| Wagner et al. | n.a. | n.a. | n.a. | n.a. | n.a. | n.a. | n.a. | n.a. | n.a. | n.a. | n.a. | n.a. | n.a. |
| Wan et al. | n.a. | n.a. | n.a. | n.a. | n.a. | n.a. | n.a. | n.a. | n.a. | n.a. | n.a. | n.a. | n.a. |
| Wigren et al. | n.a. | n.a. | n.a. | n.a. | n.a. | n.a. | n.a. | n.a. | n.a. | n.a. | n.a. | n.a. | n.a. |
| Worthen et al. | n.a. | n.a. | n.a. | n.a. | n.a. | n.a. | n.a. | n.a. | n.a. | n.a. | n.a. | n.a. | n.a. |
| Xue et al. | n.a. | n.a. | n.a. | n.a. | n.a. | n.a. | n.a. | n.a. | n.a. | n.a. | n.a. | n.a. | n.a. |
| Yoon et al. | n.a. | n.a. | n.a. | n.a. | n.a. | n.a. | n.a. | n.a. | n.a. | n.a. | n.a. | n.a. | n.a. |
| Young et al. | n.a. | n.a. | n.a. | n.a. | n.a. | n.a. | n.a. | n.a. | n.a. | n.a. | n.a. | n.a. | n.a. |
| Yu et al. | n.a. | n.a. | n.a. | n.a. | n.a. | n.a. | n.a. | n.a. | n.a. | n.a. | n.a. | n.a. | n.a. |
| Yurek et al. | n.a. | n.a. | n.a. | n.a. | n.a. | n.a. | n.a. | n.a. | n.a. | n.a. | n.a. | n.a. | n.a. |
| Zeng et al. | n.a. | n.a. | n.a. | n.a. | n.a. | n.a. | n.a. | n.a. | n.a. | n.a. | n.a. | n.a. | n.a. |

| study ID | analgesia opioid administered? (yes / no OR n.r.) | drug / compound (name OR n.a.) | administration route  (s.c. OR i.m. OR i.p. OR per os OR n.a.) | dosage  (mg/kg) OR n.r. OR n.a. | timepoint of first administration  (pre-, intra-, postsurgically OR n.a.) | if reported: immediately before surgery OR immediately after surgery OR n.r. OR n.a. | administered how many times in total?  (number OR n.a.) | if reported: administration interval  (morning after surgery OR 24h after surgery OR n.r. OR n.a.) | if reported: administration interval  (h post first administration OR n.r. OR n.a.) | if reported:  pharmaceutical formulation (e.g. solution OR n.r. OR n.a.) | other analgesics used?  E.g. metamizol (dipyrone) (yes / no OR n.r.) | if so: drug / compound  (name OR n.a.) | multimodal approaches  (total number of used compound groups (analgesics and local anesthetics))  (excluding agents used for general anesthesia; if e.g. two local anesthetics administered it is counted as one since only one substance group)  (number) | other drugs (other than analgesics) used?  If so: compound (n.a. OR name) |
| --- | --- | --- | --- | --- | --- | --- | --- | --- | --- | --- | --- | --- | --- | --- |
| Bartolomucci et al. | no | n.a. | n.a. | n.a. | n.a. | n.a. | n.a. | n.a. | n.a. | n.a. | no | n.a. | 0 | n.a. |
| Behrend et al. | no | n.a. | n.a. | n.a. | n.a. | n.a. | n.a. | n.a. | n.a. | n.a. | no | n.a. | 0 | n.a. |
| Biella et al. | no | n.a. | n.a. | n.a. | n.a. | n.a. | n.a. | n.a. | n.a. | n.a. | no | n.a. | 0 | gallamine triethiodide |
| Boni et al. | no | n.a. | n.a. | n.a. | n.a. | n.a. | n.a. | n.a. | n.a. | n.a. | no | n.a. | 0 | n.a. |
| Bramlett et al. | no | n.a. | n.a. | n.a. | n.a. | n.a. | n.a. | n.a. | n.a. | n.a. | no | n.a. | 0 | gallamine triethiodide |
| Byun et al. | no | n.a. | n.a. | n.a. | n.a. | n.a. | n.a. | n.a. | n.a. | n.a. | no | n.a. | 0 | n.a. |
| Caltana et al. | no | n.a. | n.a. | n.a. | n.a. | n.a. | n.a. | n.a. | n.a. | n.a. | no | n.a. | 0 | n.a. |
| Carcak et al. | no | n.a. | n.a. | n.a. | n.a. | n.a. | n.a. | n.a. | n.a. | n.a. | no | n.a. | 0 | n.a. |
| Cemil et al. | no | n.a. | n.a. | n.a. | n.a. | n.a. | n.a. | n.a. | n.a. | n.a. | no | n.a. | 0 | n.a. |
| Chen et al. D | no | n.a. | n.a. | n.a. | n.a. | n.a. | n.a. | n.a. | n.a. | n.a. | no | n.a. | 0 | n.a. |
| Cifani et al. | no | n.a. | n.a. | n.a. | n.a. | n.a. | n.a. | n.a. | n.a. | n.a. | no | n.a. | 0 | n.a. |
| Cunningham et al. | no | n.a. | n.a. | n.a. | n.a. | n.a. | n.a. | n.a. | n.a. | n.a. | no | n.a. | 0 | n.a. |
| Datta et al. | no | n.a. | n.a. | n.a. | n.a. | n.a. | n.a. | n.a. | n.a. | n.a. | no | n.a. | 0 | n.a. |
| Diesch et al. | no | n.a. | n.a. | n.a. | n.a. | n.a. | n.a. | n.a. | n.a. | n.a. | no | n.a. | 0 | n.a. |
| Diguet et al. | no | n.a. | n.a. | n.a. | n.a. | n.a. | n.a. | n.a. | n.a. | n.a. | no | n.a. | 0 | n.a. |
| Ding et al. | no | n.a. | n.a. | n.a. | n.a. | n.a. | n.a. | n.a. | n.a. | n.a. | no | n.a. | 0 | n.a. |
| Doan et al. | no | n.a. | n.a. | n.a. | n.a. | n.a. | n.a. | n.a. | n.a. | n.a. | no | n.a. | 0 | n.a. |
| Doretto et al. | no | n.a. | n.a. | n.a. | n.a. | n.a. | n.a. | n.a. | n.a. | n.a. | no | n.a. | 0 | n.a. |
| Dux et al. | no | n.a. | n.a. | n.a. | n.a. | n.a. | n.a. | n.a. | n.a. | n.a. | no | n.a. | 0 | n.a. |
| Echegoyen et al. | no | n.a. | n.a. | n.a. | n.a. | n.a. | n.a. | n.a. | n.a. | n.a. | no | n.a. | 0 | n.a. |
| Ehrlichman et al. | no | n.a. | n.a. | n.a. | n.a. | n.a. | n.a. | n.a. | n.a. | n.a. | no | n.a. | 0 | n.a. |
| Etholm et al. | no | n.a. | n.a. | n.a. | n.a. | n.a. | n.a. | n.a. | n.a. | n.a. | no | n.a. | 0 | n.a. |
| Farias et al. | no | n.a. | n.a. | n.a. | n.a. | n.a. | n.a. | n.a. | n.a. | n.a. | no | n.a. | 0 | n.a. |
| Foti et al. | no | n.a. | n.a. | n.a. | n.a. | n.a. | n.a. | n.a. | n.a. | n.a. | no | n.a. | 0 | n.a. |
| Francois et al. | no | n.a. | n.a. | n.a. | n.a. | n.a. | n.a. | n.a. | n.a. | n.a. | no | n.a. | 0 | n.a. |
| Francois et al. | no | n.a. | n.a. | n.a. | n.a. | n.a. | n.a. | n.a. | n.a. | n.a. | no | n.a. | 0 | n.a. |
| Fritsch et al. | no | n.a. | n.a. | n.a. | n.a. | n.a. | n.a. | n.a. | n.a. | n.a. | no | n.a. | 0 | atipamezole |
| Good et al. | no | n.a. | n.a. | n.a. | n.a. | n.a. | n.a. | n.a. | n.a. | n.a. | no | n.a. | 0 | n.a. |
| Griesbach et al. | no | n.a. | n.a. | n.a. | n.a. | n.a. | n.a. | n.a. | n.a. | n.a. | no | n.a. | 0 | n.a. |
| Guidine et al. | no | n.a. | n.a. | n.a. | n.a. | n.a. | n.a. | n.a. | n.a. | n.a. | no | n.a. | 0 | n.a. |
| Gurevicius et al. | no | n.a. | n.a. | n.a. | n.a. | n.a. | n.a. | n.a. | n.a. | n.a. | no | n.a. | 1 | n.a. |
| Hart et al. | no | n.a. | n.a. | n.a. | n.a. | n.a. | n.a. | n.a. | n.a. | n.a. | no | n.a. | 0 | n.a. |
| Harvey et al. | no | n.a. | n.a. | n.a. | n.a. | n.a. | n.a. | n.a. | n.a. | n.a. | no | n.a. | 0 | n.a. |
| Hernandez-Gonzalez et al. | no | n.a. | n.a. | n.a. | n.a. | n.a. | n.a. | n.a. | n.a. | n.a. | no | n.a. | 0 | n.a. |
| Ho et al. | no | n.a. | n.a. | n.a. | n.a. | n.a. | n.a. | n.a. | n.a. | n.a. | no | n.a. | 0 | n.a. |
| Holtmaat et al. | yes | buprenorphine | s.c. | 1 | postsurgically | immediately after surgery | n.r. | n.r. | 12 | n.r. | no | n.a. | 1 | dexamethasone |
| Hrncic et al. | no | n.a. | n.a. | n.a. | n.a. | n.a. | n.a. | n.a. | n.a. | n.a. | no | n.a. | 0 | n.a. |
| Huguet et al. | no | n.a. | n.a. | n.a. | n.a. | n.a. | n.a. | n.a. | n.a. | n.a. | no | n.a. | 0 | n.a. |
| Ishida et al. | no | n.a. | n.a. | n.a. | n.a. | n.a. | n.a. | n.a. | n.a. | n.a. | no | n.a. | 0 | n.a. |
| Ito et al. | no | n.a. | n.a. | n.a. | n.a. | n.a. | n.a. | n.a. | n.a. | n.a. | no | n.a. | 0 | n.a. |
| Itoh et al. B | no | n.a. | n.a. | n.a. | n.a. | n.a. | n.a. | n.a. | n.a. | n.a. | no | n.a. | 0 | n.a. |
| Itoh et al. A | no | n.a. | n.a. | n.a. | n.a. | n.a. | n.a. | n.a. | n.a. | n.a. | no | n.a. | 0 | n.a. |
| Jafri et al. | yes | buprenorphine | s.c. | 0,02 to 0,05 | presurgically | n.r. | 1 | n.a. | n.a. | n.r. | no | n.a. | 1 | n.a. |
| Kalauzi et al. | no | n.a. | n.a. | n.a. | n.a. | n.a. | n.a. | n.a. | n.a. | n.a. | no | n.a. | 0 | n.a. |
| Katz et al. | no | n.a. | n.a. | n.a. | n.a. | n.a. | n.a. | n.a. | n.a. | n.a. | no | n.a. | 0 | n.a. |
| Kim et al. A | no | n.a. | n.a. | n.a. | n.a. | n.a. | n.a. | n.a. | n.a. | n.a. | no | n.a. | 0 | n.a. |
| Lackovic et al. | no | n.a. | n.a. | n.a. | n.a. | n.a. | n.a. | n.a. | n.a. | n.a. | no | n.a. | 0 | n.a. |
| Lee et al. B | no | n.a. | n.a. | n.a. | n.a. | n.a. | n.a. | n.a. | n.a. | n.a. | no | n.a. | 0 | n.a. |
| Lee et al. A | no | n.a. | n.a. | n.a. | n.a. | n.a. | n.a. | n.a. | n.a. | n.a. | no | n.a. | 0 | n.a. |
| Li et al. C | no | n.a. | n.a. | n.a. | n.a. | n.a. | n.a. | n.a. | n.a. | n.a. | no | n.a. | 0 | n.a. |
| Liu et al. | no | n.a. | n.a. | n.a. | n.a. | n.a. | n.a. | n.a. | n.a. | n.a. | no | n.a. | 0 | n.a. |
| Lopez-Martin et al. | no | n.a. | n.a. | n.a. | n.a. | n.a. | n.a. | n.a. | n.a. | n.a. | no | n.a. | 0 | n.a. |
| Lu et al. | no | n.a. | n.a. | n.a. | n.a. | n.a. | n.a. | n.a. | n.a. | n.a. | no | n.a. | 0 | n.a. |
| Lundblad et al. | no | n.a. | n.a. | n.a. | n.a. | n.a. | n.a. | n.a. | n.a. | n.a. | no | n.a. | 0 | n.a. |
| Magloire et al. | no | n.a. | n.a. | n.a. | n.a. | n.a. | n.a. | n.a. | n.a. | n.a. | no | n.a. | 1 | n.a. |
| Mark et al. | no | n.a. | n.a. | n.a. | n.a. | n.a. | n.a. | n.a. | n.a. | n.a. | no | n.a. | 0 | alpha-chloralose |
| McCracken et al. | no | n.a. | n.a. | n.a. | n.a. | n.a. | n.a. | n.a. | n.a. | n.a. | no | n.a. | 0 | n.a. |
| Meeren et al. | no | n.a. | n.a. | n.a. | n.a. | n.a. | n.a. | n.a. | n.a. | n.a. | no | n.a. | 0 | n.a. |
| Merkler et al. | no | n.a. | n.a. | n.a. | n.a. | n.a. | n.a. | n.a. | n.a. | n.a. | no | n.a. | 0 | n.a. |
| Merkler et al. | no | n.a. | n.a. | n.a. | n.a. | n.a. | n.a. | n.a. | n.a. | n.a. | no | n.a. | 0 | n.a. |
| Mian et al. | no | n.a. | n.a. | n.a. | n.a. | n.a. | n.a. | n.a. | n.a. | n.a. | no | n.a. | 0 | n.a. |
| Mohammadi et al. | no | n.a. | n.a. | n.a. | n.a. | n.a. | n.a. | n.a. | n.a. | n.a. | no | n.a. | 0 | n.a. |
| Mollazaedh et al. | no | n.a. | n.a. | n.a. | n.a. | n.a. | n.a. | n.a. | n.a. | n.a. | no | n.a. | 0 | n.a. |
| Mukherjee et al. | no | n.a. | n.a. | n.a. | n.a. | n.a. | n.a. | n.a. | n.a. | n.a. | no | n.a. | 0 | n.a. |
| Nehlig et al. | no | n.a. | n.a. | n.a. | n.a. | n.a. | n.a. | n.a. | n.a. | n.a. | no | n.a. | 0 | n.a. |
| Nuki et al. | no | n.a. | n.a. | n.a. | n.a. | n.a. | n.a. | n.a. | n.a. | n.a. | no | n.a. | 0 | n.a. |
| Onyszchuk et al. | no | n.a. | n.a. | n.a. | n.a. | n.a. | n.a. | n.a. | n.a. | n.a. | no | n.a. | 0 | n.a. |
| Oshima et al. | no | n.a. | n.a. | n.a. | n.a. | n.a. | n.a. | n.a. | n.a. | n.a. | no | n.a. | 0 | n.a. |
| Potts et al. | no | n.a. | n.a. | n.a. | n.a. | n.a. | n.a. | n.a. | n.a. | n.a. | no | n.a. | 0 | n.a. |
| Qing et al. | no | n.a. | n.a. | n.a. | n.a. | n.a. | n.a. | n.a. | n.a. | n.a. | no | n.a. | 0 | n.a. |
| Rahim et al. | no | n.a. | n.a. | n.a. | n.a. | n.a. | n.a. | n.a. | n.a. | n.a. | no | n.a. | 0 | n.a. |
| Rimoli et al. | no | n.a. | n.a. | n.a. | n.a. | n.a. | n.a. | n.a. | n.a. | n.a. | no | n.a. | 0 | n.a. |
| Roiko et al. | no | n.a. | n.a. | n.a. | n.a. | n.a. | n.a. | n.a. | n.a. | n.a. | no | n.a. | 0 | n.a. |
| Rudnick et al. | no | n.a. | n.a. | n.a. | n.a. | n.a. | n.a. | n.a. | n.a. | n.a. | no | n.a. | 0 | n.a. |
| Sahin et al. | no | n.a. | n.a. | n.a. | n.a. | n.a. | n.a. | n.a. | n.a. | n.a. | no | n.a. | 0 | n.a. |
| Samnick et al. | no | n.a. | n.a. | n.a. | n.a. | n.a. | n.a. | n.a. | n.a. | n.a. | no | n.a. | 0 | n.a. |
| Sasaki et al. | no | n.a. | n.a. | n.a. | n.a. | n.a. | n.a. | n.a. | n.a. | n.a. | no | n.a. | 0 | n.a. |
| Schei et al. | no | n.a. | n.a. | n.a. | n.a. | n.a. | n.a. | n.a. | n.a. | n.a. | no | n.a. | 1 | n.a. |
| Schmid et al. | no | n.a. | n.a. | n.a. | n.a. | n.a. | n.a. | n.a. | n.a. | n.a. | no | n.a. | 0 | n.a. |
| Sekiya et al. | no | n.a. | n.a. | n.a. | n.a. | n.a. | n.a. | n.a. | n.a. | n.a. | no | n.a. | 0 | n.a. |
| Sher et al. | no | n.a. | n.a. | n.a. | n.a. | n.a. | n.a. | n.a. | n.a. | n.a. | no | n.a. | 0 | n.a. |
| Shultz et al. | no | n.a. | n.a. | n.a. | n.a. | n.a. | n.a. | n.a. | n.a. | n.a. | no | n.a. | 1 | n.a. |
| Silvani et al. | no | n.a. | n.a. | n.a. | n.a. | n.a. | n.a. | n.a. | n.a. | n.a. | no | n.a. | 0 | n.a. |
| Sinton et al. | no | n.a. | n.a. | n.a. | n.a. | n.a. | n.a. | n.a. | n.a. | n.a. | no | n.a. | 0 | n.a. |
| Song et al. | no | n.a. | n.a. | n.a. | n.a. | n.a. | n.a. | n.a. | n.a. | n.a. | no | n.a. | 0 | n.a. |
| Takahashi et al. | no | n.a. | n.a. | n.a. | n.a. | n.a. | n.a. | n.a. | n.a. | n.a. | no | n.a. | 0 | n.a. |
| Takahashi et al. | no | n.a. | n.a. | n.a. | n.a. | n.a. | n.a. | n.a. | n.a. | n.a. | no | n.a. | 0 | n.a. |
| Tanida et al. | no | n.a. | n.a. | n.a. | n.a. | n.a. | n.a. | n.a. | n.a. | n.a. | no | n.a. | 0 | n.a. |
| Tchekalarova et al. | no | n.a. | n.a. | n.a. | n.a. | n.a. | n.a. | n.a. | n.a. | n.a. | no | n.a. | 0 | n.a. |
| Thomas et al. | no | n.a. | n.a. | n.a. | n.a. | n.a. | n.a. | n.a. | n.a. | n.a. | no | n.a. | 0 | n.a. |
| Topchiy et al. | no | n.a. | n.a. | n.a. | n.a. | n.a. | n.a. | n.a. | n.a. | n.a. | no | n.a. | 1 | n.a. |
| Touzani et al. | no | n.a. | n.a. | n.a. | n.a. | n.a. | n.a. | n.a. | n.a. | n.a. | no | n.a. | 0 | n.a. |
| Tsanov et al. | no | n.a. | n.a. | n.a. | n.a. | n.a. | n.a. | n.a. | n.a. | n.a. | no | n.a. | 0 | n.a. |
| Wagner et al. | no | n.a. | n.a. | n.a. | n.a. | n.a. | n.a. | n.a. | n.a. | n.a. | no | n.a. | 0 | n.a. |
| Wan et al. | no | n.a. | n.a. | n.a. | n.a. | n.a. | n.a. | n.a. | n.a. | n.a. | no | n.a. | 0 | n.a. |
| Wigren et al. | no | n.a. | n.a. | n.a. | n.a. | n.a. | n.a. | n.a. | n.a. | n.a. | no | n.a. | 0 | n.a. |
| Worthen et al. | no | n.a. | n.a. | n.a. | n.a. | n.a. | n.a. | n.a. | n.a. | n.a. | no | n.a. | 0 | n.a. |
| Xue et al. | no | n.a. | n.a. | n.a. | n.a. | n.a. | n.a. | n.a. | n.a. | n.a. | no | n.a. | 0 | n.a. |
| Yoon et al. | no | n.a. | n.a. | n.a. | n.a. | n.a. | n.a. | n.a. | n.a. | n.a. | no | n.a. | 0 | n.a. |
| Young et al. | no | n.a. | n.a. | n.a. | n.a. | n.a. | n.a. | n.a. | n.a. | n.a. | no | n.a. | 0 | n.a. |
| Yu et al. | no | n.a. | n.a. | n.a. | n.a. | n.a. | n.a. | n.a. | n.a. | n.a. | no | n.a. | 0 | n.a. |
| Yurek et al. | no | n.a. | n.a. | n.a. | n.a. | n.a. | n.a. | n.a. | n.a. | n.a. | no | n.a. | 0 | n.a. |
| Zeng et al. | no | n.a. | n.a. | n.a. | n.a. | n.a. | n.a. | n.a. | n.a. | n.a. | no | n.a. | 0 | n.a. |

| study ID | antibiotic agent  used? If so, list drug/ compound (name OR unclear OR no OR n.r.) | administration route  (s.c. OR i.m. OR topically OR per os OR n.a.) | specific monitoring reported? (heartrate, breathing, body temperature) (yes / no) | if so, specify: e.g. heartbeat, pulse, breathing, body temperature OR n.a. | peri-operative care reported?  (yes / no) | if so, specify:  e.g. body temperature maintained, saline drip OR n.a. | non-pharmacological measures for pain management reported? (probably not) (yes / no) | if so, specify: e.g. cooling of incision, non-pharmacological substances applied OR n.a. | refinement measures reported?  (yes / no) | if so, specify:  e.g. recovery on heating pad, bolus of saline, housed individually after surgery OR n.a. |
| --- | --- | --- | --- | --- | --- | --- | --- | --- | --- | --- |
| Bartolomucci et al. | unclear | n.a. | no | n.a. | no | n.a. | no | n.a. | no | n.a. |
| Behrend et al. | unclear | n.a. | no | n.a. | no | n.a. | no | n.a. | no | n.a. |
| Biella et al. | unclear | n.a. | yes | breathing, body temperature | yes | body temperature maintained | no | n.a. | no | n.a. |
| Boni et al. | unclear | n.a. | yes | arterial blood pressure, breathing | no | n.a. | no | n.a. | no | n.a. |
| Bramlett et al. | unclear | n.a. | yes | breathing, blood gases, body temperature | no | n.a. | no | n.a. | no | n.a. |
| Byun et al. | unclear | n.a. | no | n.a. | no | n.a. | no | n.a. | no | n.a. |
| Caltana et al. | unclear | n.a. | yes | body temperature | yes | body temperature maintained | no | n.a. | yes | housed individually |
| Carcak et al. | unclear | n.a. | no | n.a. | no | n.a. | no | n.a. | no | n.a. |
| Cemil et al. | unclear | n.a. | no | n.a. | no | n.a. | no | n.a. | no | n.a. |
| Chen et al. D | unclear | n.a. | no | n.a. | no | n.a. | no | n.a. | no | n.a. |
| Cifani et al. | penicillin / streptomycin | i.m. | no | n.a. | no | n.a. | no | n.a. | no | n.a. |
| Cunningham et al. | unclear | n.a. | no | n.a. | no | n.a. | no | n.a. | no | n.a. |
| Datta et al. | unclear | n.a. | no | n.a. | no | n.a. | no | n.a. | no | n.a. |
| Diesch et al. | unclear | n.a. | yes | body temperature | yes | body temperature maintained | no | n.a. | no | n.a. |
| Diguet et al. | unclear | n.a. | no | n.a. | no | n.a. | no | n.a. | no | n.a. |
| Ding et al. | unclear | n.a. | no | n.a. | no | n.a. | no | n.a. | yes | housed individually |
| Doan et al. | unclear | n.a. | yes | physiological parameters, body temperature | no | n.a. | no | n.a. | no | n.a. |
| Doretto et al. | unclear | n.a. | no | n.a. | no | n.a. | no | n.a. | no | n.a. |
| Dux et al. | unclear | n.a. | yes | systemic blood pressure, body temperature, breathing | yes | body temperature maintained | no | n.a. | no | n.a. |
| Echegoyen et al. | unclear | n.a. | no | n.a. | no | n.a. | no | n.a. | no | n.a. |
| Ehrlichman et al. | unclear | n.a. | no | n.a. | no | n.a. | no | n.a. | yes | housed individually |
| Etholm et al. | unclear | n.a. | no | n.a. | no | n.a. | no | n.a. | no | n.a. |
| Farias et al. | antibiotic ointment | topically | no | n.a. | no | n.a. | no | n.a. | no | n.a. |
| Foti et al. | unclear | n.a. | no | n.a. | no | n.a. | no | n.a. | no | n.a. |
| Francois et al. | unclear | n.a. | yes | body temperature | yes | body temperature maintained | no | n.a. | yes | recovery on heating pad |
| Francois et al. | unclear | n.a. | no | n.a. | no | n.a. | no | n.a. | no | n.a. |
| Fritsch et al. | unclear | n.a. | no | n.a. | no | n.a. | no | n.a. | no | n.a. |
| Good et al. | unclear | n.a. | no | n.a. | no | n.a. | no | n.a. | no | n.a. |
| Griesbach et al. | triple antibiotic | n.a. | yes | breathing, muscular relaxation, corneal reflex, pedal reflex, body temperature | yes | body temperature maintained | no | n.a. | no | n.a. |
| Guidine et al. | enrofloxacine | s.c. | no | n.a. | no | n.a. | no | n.a. | yes | housed individually |
| Gurevicius et al. | unclear | n.a. | no | n.a. | no | n.a. | no | n.a. | no | n.a. |
| Hart et al. | penicillin | i.p. | no | n.a. | no | n.a. | no | n.a. | yes | weighed and handled daily |
| Harvey et al. | unclear | n.a. | no | n.a. | no | n.a. | no | n.a. | no | n.a. |
| Hernandez-Gonzalez et al. | unclear | n.a. | no | n.a. | no | n.a. | no | n.a. | no | n.a. |
| Ho et al. | unclear | n.a. | no | n.a. | no | n.a. | no | n.a. | no | n.a. |
| Holtmaat et al. | enrofloxacine | s.c. | yes | body temperature | yes | body temperature maintained, eye ointment applied | no | n.a. | yes | lactated ringer's solution, recovery on heating pad, wet food |
| Hrncic et al. | unclear | n.a. | no | n.a. | no | n.a. | no | n.a. | no | n.a. |
| Huguet et al. | unclear | n.a. | no | n.a. | no | n.a. | no | n.a. | yes | weighed and handled daily |
| Ishida et al. | unclear | n.a. | no | n.a. | no | n.a. | no | n.a. | no | n.a. |
| Ito et al. | unclear | n.a. | no | n.a. | no | n.a. | no | n.a. | no | n.a. |
| Itoh et al. B | unclear | n.a. | no | n.a. | no | n.a. | no | n.a. | no | n.a. |
| Itoh et al. A | unclear | n.a. | no | n.a. | no | n.a. | no | n.a. | yes | recovery on heating pad |
| Jafri et al. | unclear | n.a. | yes | body temperature | yes | body temperature maintained, eye ointment applied | no | n.a. | no | n.a. |
| Kalauzi et al. | unclear | n.a. | no | n.a. | no | n.a. | no | n.a. | no | n.a. |
| Katz et al. | unclear | n.a. | no | n.a. | no | n.a. | no | n.a. | yes | housed individually |
| Kim et al. A | unclear | n.a. | no | n.a. | no | n.a. | no | n.a. | no | n.a. |
| Lackovic et al. | unclear | n.a. | no | n.a. | no | n.a. | no | n.a. | no | n.a. |
| Lee et al. B | doxycycline | n.a. | no | n.a. | no | n.a. | no | n.a. | no | n.a. |
| Lee et al. A | unclear | n.a. | no | n.a. | no | n.a. | no | n.a. | no | n.a. |
| Li et al. C | unclear | n.a. | no | n.a. | no | n.a. | no | n.a. | yes | housed individually |
| Liu et al. | unclear | n.a. | no | n.a. | no | n.a. | no | n.a. | no | n.a. |
| Lopez-Martin et al. | unclear | n.a. | no | n.a. | no | n.a. | no | n.a. | no | n.a. |
| Lu et al. | unclear | n.a. | yes | body temperature | yes | body temperature maintained | no | n.a. | no | n.a. |
| Lundblad et al. | unclear | n.a. | yes | body temperature | yes | body temperature maintained | no | n.a. | yes | recovery on heating pad |
| Magloire et al. | unclear | n.a. | yes | body temperature | yes | body temperature maintained | no | n.a. | no | n.a. |
| Mark et al. | unclear | n.a. | yes | arterial pressure, heart rate, body temperature | yes | body temperature maintained | no | n.a. | no | n.a. |
| McCracken et al. | unclear | n.a. | no | n.a. | no | n.a. | no | n.a. | no | n.a. |
| Meeren et al. | unclear | n.a. | no | n.a. | no | n.a. | no | n.a. | yes | housed individually |
| Merkler et al. | unclear | n.a. | yes | body temperature | yes | body temperature maintained | no | n.a. | no | n.a. |
| Merkler et al. | unclear | n.a. | no | n.a. | no | n.a. | no | n.a. | no | n.a. |
| Mian et al. | unclear | n.a. | no | n.a. | no | n.a. | no | n.a. | no | n.a. |
| Mohammadi et al. | unclear | n.a. | yes | breathing, body temperature | yes | body temperature maintained | no | n.a. | no | n.a. |
| Mollazaedh et al. | unclear | n.a. | no | n.a. | no | n.a. | no | n.a. | no | n.a. |
| Mukherjee et al. | unclear | n.a. | no | n.a. | no | n.a. | no | n.a. | no | n.a. |
| Nehlig et al. | unclear | n.a. | no | n.a. | no | n.a. | no | n.a. | yes | handled daily twice |
| Nuki et al. | cefalexin | n.a. | no | n.a. | no | n.a. | no | n.a. | no | n.a. |
| Onyszchuk et al. | unclear | n.a. | no | n.a. | no | n.a. | no | n.a. | no | n.a. |
| Oshima et al. | unclear | n.a. | no | n.a. | no | n.a. | no | n.a. | yes | n.a. |
| Potts et al. | unclear | n.a. | yes | body temperature | yes | body temperature maintained | no | n.a. | yes | 1 ml saline s.c. |
| Qing et al. | unclear | n.a. | no | n.a. | no | n.a. | no | n.a. | no | n.a. |
| Rahim et al. | unclear | n.a. | no | n.a. | no | n.a. | no | n.a. | no | n.a. |
| Rimoli et al. | unclear | n.a. | no | n.a. | no | n.a. | no | n.a. | yes | handled daily |
| Roiko et al. | enrofloxacine | i.m. | no | n.a. | no | n.a. | no | n.a. | no | n.a. |
| Rudnick et al. | unclear | n.a. | no | n.a. | no | n.a. | no | n.a. | no | n.a. |
| Sahin et al. | unclear | n.a. | no | n.a. | no | n.a. | no | n.a. | no | n.a. |
| Samnick et al. | unclear | n.a. | no | n.a. | yes | eye ointment applied | no | n.a. | no | n.a. |
| Sasaki et al. | unclear | n.a. | yes | breathing, body temperature, blood pressure | yes | body temperature maintained | no | n.a. | no | n.a. |
| Schei et al. | n.r. | n.r. | yes | body temperature, heart rate, breathing | yes | body temperature maintained | no | n.a. | no | n.a. |
| Schmid et al. | unclear | n.a. | yes | body temperature | yes | body temperature maintained | no | n.a. | no | n.a. |
| Sekiya et al. | unclear | n.a. | no | n.a. | no | n.a. | no | n.a. | no | n.a. |
| Sher et al. | unclear | n.a. | no | n.a. | no | n.a. | no | n.a. | no | n.a. |
| Shultz et al. | unclear | n.a. | no | n.a. | no | n.a. | no | n.a. | no | n.a. |
| Silvani et al. | unclear | n.a. | yes | body temperature | yes | body temperature maintained | no | n.a. | no | n.a. |
| Sinton et al. | n.r. | topically | no | n.a. | no | n.a. | no | n.a. | yes | recovery on heating pad |
| Song et al. | unclear | n.a. | yes | body temperature, heart rate, breathing, reflexes | yes | body temperature maintained | no | n.a. | no | n.a. |
| Takahashi et al. | unclear | n.a. | no | n.a. | no | n.a. | no | n.a. | no | n.a. |
| Takahashi et al. | unclear | n.a. | no | n.a. | no | n.a. | no | n.a. | no | n.a. |
| Tanida et al. | unclear | n.a. | no | n.a. | no | n.a. | no | n.a. | no | n.a. |
| Tchekalarova et al. | unclear | n.a. | no | n.a. | no | n.a. | no | n.a. | no | n.a. |
| Thomas et al. | unclear | n.a. | no | n.a. | no | n.a. | no | n.a. | no | n.a. |
| Topchiy et al. | gentamycin | n.r. | yes | body temperature, ecg rate | yes | body temperature maintained, eye ointment applied | no | n.a. | yes | bolus of lactated Ringers solution |
| Touzani et al. | penicillin | i.m. | no | n.a. | no | n.a. | no | n.a. | no | n.a. |
| Tsanov et al. | unclear | n.a. | no | n.a. | no | n.a. | no | n.a. | no | n.a. |
| Wagner et al. | unclear | n.a. | no | n.a. | no | n.a. | no | n.a. | no | n.a. |
| Wan et al. | unclear | n.a. | yes | breathing, mean atrial pressure, body temperature | yes | body temperature maintained, dural surface perfused with saline | no | n.a. | no | n.a. |
| Wigren et al. | unclear | n.a. | no | n.a. | no | n.a. | no | n.a. | yes | housed individually |
| Worthen et al. | unclear | n.a. | no | n.a. | no | n.a. | no | n.a. | no | n.a. |
| Xue et al. | unclear | n.a. | no | n.a. | no | n.a. | no | n.a. | no | n.a. |
| Yoon et al. | unclear | n.a. | no | n.a. | no | n.a. | no | n.a. | no | n.a. |
| Young et al. | unclear | n.a. | no | n.a. | no | n.a. | no | n.a. | no | n.a. |
| Yu et al. | unclear | n.a. | no | n.a. | no | n.a. | no | n.a. | no | n.a. |
| Yurek et al. | unclear | n.a. | no | n.a. | no | n.a. | no | n.a. | no | n.a. |
| Zeng et al. | unclear | n.a. | no | n.a. | no | n.a. | no | n.a. | no | n.a. |

| study ID | assessment of  analgesic efficacy post surgery reported?  (yes / no) | parameters testing efficacy of pain/stress reducing  measures post surgery reported?  (e.g. Mouse Grimace Scale) (yes / no) | if so:  which parameters are reported?  (name of method OR n.a.) | blinding reported?  (yes / no) | randomization reported?  (yes / no) | Power Analysis reported? (yes / no) | if so: Power Analysis reported in detail? (yes / no OR n.a.) |
| --- | --- | --- | --- | --- | --- | --- | --- |
| Bartolomucci et al. | no | no | n.a. | no | no | no | n.a. |
| Behrend et al. | no | no | n.a. | no | no | no | n.a. |
| Biella et al. | no | no | n.a. | no | no | no | n.a. |
| Boni et al. | no | no | n.a. | no | no | no | n.a. |
| Bramlett et al. | no | no | n.a. | no | no | no | n.a. |
| Byun et al. | no | no | n.a. | no | no | no | n.a. |
| Caltana et al. | no | no | n.a. | no | no | no | n.a. |
| Carcak et al. | no | no | n.a. | no | yes | no | n.a. |
| Cemil et al. | no | no | n.a. | no | yes | no | n.a. |
| Chen et al. D | no | no | n.a. | no | no | no | n.a. |
| Cifani et al. | no | no | n.a. | no | no | no | n.a. |
| Cunningham et al. | no | no | n.a. | no | no | no | n.a. |
| Datta et al. | no | no | n.a. | no | yes | no | n.a. |
| Diesch et al. | no | no | n.a. | no | no | no | n.a. |
| Diguet et al. | no | no | n.a. | yes | no | no | n.a. |
| Ding et al. | no | no | n.a. | no | no | no | n.a. |
| Doan et al. | no | no | n.a. | no | no | no | n.a. |
| Doretto et al. | no | no | n.a. | no | no | no | n.a. |
| Dux et al. | no | no | n.a. | no | no | no | n.a. |
| Echegoyen et al. | no | no | n.a. | no | no | no | n.a. |
| Ehrlichman et al. | no | no | n.a. | no | no | no | n.a. |
| Etholm et al. | no | no | n.a. | no | no | no | n.a. |
| Farias et al. | no | no | n.a. | yes | no | no | n.a. |
| Foti et al. | no | no | n.a. | no | no | no | n.a. |
| Francois et al. | no | no | n.a. | no | no | no | n.a. |
| Francois et al. | no | no | n.a. | no | no | no | n.a. |
| Fritsch et al. | no | no | n.a. | no | no | no | n.a. |
| Good et al. | no | no | n.a. | no | no | no | n.a. |
| Griesbach et al. | no | no | n.a. | yes | yes | no | n.a. |
| Guidine et al. | no | no | n.a. | no | no | no | n.a. |
| Gurevicius et al. | no | no | n.a. | no | no | no | n.a. |
| Hart et al. | no | no | n.a. | yes | no | yes | no |
| Harvey et al. | no | no | n.a. | no | no | no | n.a. |
| Hernandez-Gonzalez et al. | no | no | n.a. | no | no | no | n.a. |
| Ho et al. | no | no | n.a. | no | no | no | n.a. |
| Holtmaat et al. | no | no | n.a. | no | no | no | n.a. |
| Hrncic et al. | no | no | n.a. | no | no | no | n.a. |
| Huguet et al. | no | no | n.a. | no | no | no | n.a. |
| Ishida et al. | no | no | n.a. | no | no | no | n.a. |
| Ito et al. | no | no | n.a. | no | no | no | n.a. |
| Itoh et al. B | no | no | n.a. | no | yes | no | n.a. |
| Itoh et al. A | no | no | n.a. | no | no | no | n.a. |
| Jafri et al. | no | no | n.a. | no | no | no | n.a. |
| Kalauzi et al. | no | no | n.a. | no | no | no | n.a. |
| Katz et al. | no | no | n.a. | no | yes | no | n.a. |
| Kim et al. A | no | no | n.a. | no | no | no | n.a. |
| Lackovic et al. | no | no | n.a. | no | yes | no | n.a. |
| Lee et al. B | no | no | n.a. | no | no | no | n.a. |
| Lee et al. A | no | no | n.a. | no | no | no | n.a. |
| Li et al. C | no | no | n.a. | no | no | no | n.a. |
| Liu et al. | no | no | n.a. | yes | yes | no | n.a. |
| Lopez-Martin et al. | no | no | n.a. | yes | no | no | n.a. |
| Lu et al. | no | no | n.a. | yes | no | no | n.a. |
| Lundblad et al. | no | no | n.a. | yes | no | no | n.a. |
| Magloire et al. | no | no | n.a. | no | no | no | n.a. |
| Mark et al. | no | no | n.a. | yes | no | no | n.a. |
| McCracken et al. | no | no | n.a. | no | no | no | n.a. |
| Meeren et al. | no | no | n.a. | no | no | no | n.a. |
| Merkler et al. | no | no | n.a. | yes | yes | no | n.a. |
| Merkler et al. | no | no | n.a. | yes | yes | no | n.a. |
| Mian et al. | no | no | n.a. | no | no | no | n.a. |
| Mohammadi et al. | no | no | n.a. | no | no | no | n.a. |
| Mollazaedh et al. | no | no | n.a. | no | no | no | n.a. |
| Mukherjee et al. | no | no | n.a. | no | no | no | n.a. |
| Nehlig et al. | no | no | n.a. | no | no | no | n.a. |
| Nuki et al. | no | no | n.a. | yes | no | no | n.a. |
| Onyszchuk et al. | no | no | n.a. | no | no | no | n.a. |
| Oshima et al. | no | no | n.a. | no | no | no | n.a. |
| Potts et al. | no | no | n.a. | yes | no | no | n.a. |
| Qing et al. | no | no | n.a. | no | no | no | n.a. |
| Rahim et al. | no | no | n.a. | yes | no | no | n.a. |
| Rimoli et al. | no | no | n.a. | no | no | no | n.a. |
| Roiko et al. | no | no | n.a. | yes | no | no | n.a. |
| Rudnick et al. | no | no | n.a. | no | no | no | n.a. |
| Sahin et al. | no | no | n.a. | no | no | no | n.a. |
| Samnick et al. | no | no | n.a. | no | yes | no | n.a. |
| Sasaki et al. | no | no | n.a. | yes | yes | no | n.a. |
| Schei et al. | no | no | n.a. | no | no | no | n.a. |
| Schmid et al. | no | no | n.a. | no | no | no | n.a. |
| Sekiya et al. | no | no | n.a. | no | no | no | n.a. |
| Sher et al. | no | no | n.a. | no | no | no | n.a. |
| Shultz et al. | no | no | n.a. | yes | yes | no | n.a. |
| Silvani et al. | no | no | n.a. | yes | no | no | n.a. |
| Sinton et al. | no | no | n.a. | no | no | no | n.a. |
| Song et al. | no | no | n.a. | no | no | no | n.a. |
| Takahashi et al. | no | no | n.a. | no | no | no | n.a. |
| Takahashi et al. | no | no | n.a. | no | no | no | n.a. |
| Tanida et al. | no | no | n.a. | no | no | no | n.a. |
| Tchekalarova et al. | no | no | n.a. | no | no | no | n.a. |
| Thomas et al. | no | no | n.a. | no | no | no | n.a. |
| Topchiy et al. | no | no | n.a. | no | no | no | n.a. |
| Touzani et al. | no | no | n.a. | no | no | no | n.a. |
| Tsanov et al. | no | no | n.a. | no | no | yes | n.a. |
| Wagner et al. | no | no | n.a. | no | no | no | n.a. |
| Wan et al. | no | no | n.a. | no | yes | no | n.a. |
| Wigren et al. | no | no | n.a. | no | no | no | n.a. |
| Worthen et al. | no | no | n.a. | no | no | no | n.a. |
| Xue et al. | no | no | n.a. | yes | no | no | n.a. |
| Yoon et al. | no | no | n.a. | no | no | no | n.a. |
| Young et al. | no | no | n.a. | no | no | no | n.a. |
| Yu et al. | no | no | n.a. | no | no | no | n.a. |
| Yurek et al. | no | no | n.a. | no | no | no | n.a. |
| Zeng et al. | no | no | n.a. | yes | no | no | n.a. |

**Supplementary table S2: List of subset of 200 studies, included studies from 2019 and evaluated parameters**

Information on all evaluated parameters of studies in 2019, included in the subset of 200 studies. n.r.= not reported, parameter was not reported; n.a.= not applicable, extraction of this parameter was not feasible; i.p.= intraperitoneal; i.m.= intramuscular; s.c.= subcutaneous; tbi= traumatic brain injury; cci= controlled cortical impact.

| study ID | first author (last name) | title | year of publication (2009, 2019) | journal (in which the study was published)  (name) | issue (number or n.r.) | pages or article number (x - x) | country of origin (location of the institute the first author worked for at timepoint of publication)  (name of country) |
| --- | --- | --- | --- | --- | --- | --- | --- |
| Aldehri et al. | Aldehri | Fornix deep brain stimulation induces reduction of hippocampal synaptophysin levels | 2019 | Journal of Chemical Neuroanatomy | 96 | 34 to 40 | Netherlands |
| Asan et al. | Asan | Modulation of Multiunit Spike Activity by Transcranial AC Stimulation (tACS) in the Rat Cerebellar Cortex | 2019 | Annual International Conference of the IEEE Engineering in Medicine and Biology Society | n.a. | 5192 to 5195 | USA |
| Baud et al. | Baud | Endogenous multidien rhythm of epilepsy in rats | 2019 | Experimental Neurology | 315 | 82 to 87 | Switzerland |
| Bazzu et al. | Bazzu | Monitoring deep brain stimulation by measuring regional brain oxygen responses in freely moving mice | 2019 | Journal of Neuroscience Methods | 317 | 20 to 28 | Italy |
| Bertoglio et al. | Bertoglio | In vivo measurement of brain network connectivity reflects progression and intrinsic disease severity in a model of temporal lobe epilepsy | 2019 | Neurobiology of Disease | 127 | 45 to 52 | Belgium |
| Bleimeister et al. | Bleimeister | Environmental enrichment and amantadine confer individual but nonadditive enhancements in motor and spatial learning after controlled cortical impact injury | 2019 | Brain Research | 1714 | 227 to 233 | USA |
| Bukhtiyarova et al. | Bukhtiyarova | Slow wave detection in sleeping mice: Comparison of traditional and machine learning methods | 2019 | Journal of Neuroscience Methods | 316 | 35 to 45 | Canada |
| Burgdorf et al. | Burgdorf | A translational EEG-based approach to assess modulation of long-lasting NMDAR-dependent synaptic plasticity | 2019 | Psychopharmacology | 236 | 3687 to 3693 | USA |
| Casanova-Carvajal et al. | Casanova-Carvajal | Slowdown intracranial glioma progression by optical hyperthermia therapy: study on a CT-2A mouse astrocytoma model | 2019 | Nanotechnology | 30 | 355101 | Spain |
| Chen et al. C | Chen | Neuroprotective effect of mogrol against Abeta1-42 -induced memory impairment neuroinflammation and apoptosis in mice | 2019 | Journal of Pharmacy and Pharmacology | 71 | 869 to 877 | China |
| Chen et al. E | Chen | Optogenetic Long-Term Depression Induction in the PVT-CeL Circuitry Mediates Decreased Fear Memory | 2019 | Molecular Neurobiology | 56 | 4855 to 4865 | China |
| Chen et al. A | Chen | Astrocyte-Specific Deletion of Sox2 Promotes Functional Recovery After Traumatic Brain Injury | 2019 | Cerebral Cortex | 29 | 54 to 69 | USA |
| Chen et al. B | Chen | Intracerebroventricular Delivery of Recombinant NAMPT Deters Inflammation and Protects Against Cerebral Ischemia | 2019 | Translational Stroke Research | 10 | 719 to 728 | USA |
| Chen et al. F | Chen | The Potassium SK Channel Activator NS309 Protects Against Experimental Traumatic Brain Injury Through Anti-Inflammatory and Immunomodulatory Mechanisms | 2019 | Frontiers in Pharmacology | 10 | 1432 | China |
| Chitturi et al. | Chitturi | Beneficial Effects of Kaempferol after Developmental Traumatic Brain Injury Is through Protection of Mitochondrial Function, Oxidative Metabolism, and Neural Viability | 2019 | Journal of Neurotrauma | 36 | 1264 to 1278 | USA |
| Christiaen et al. | Christiaen | Alterations in the functional brain network in a rat model of epileptogenesis: A longitudinal resting state fMRI study | 2019 | NeuroImage | 202 | 116144 | Belgium |
| Colangeli et al. | Colangeli | Synergistic action of CB1 and 5-HT2B receptors in preventing pilocarpine-induced status epilepticus in rats | 2019 | Neurobiology of Disease | 125 | 135 to 145 | Malta |
| da Silva Pacheco et al. | da Silva Pacheco | Neurons expressing estrogen receptor alpha differentially innervate the periaqueductal gray matter of female rats | 2019 | Journal of Chemical Neuroanatomy | 97 | 33 to 42 | Brazil |
| Daglas et al. | Daglas | Activated CD8(+) T Cells Cause Long-Term Neurological Impairment after Traumatic Brain Injury in Mice | 2019 | Cell Reports | 29 | 1178 to 1191 | Australia |
| Dal-Pont et al. | Dal-Pont | Tamoxifen has an anti-manic effect but not protect the brain against oxidative stress in an animal model of mania induced by ouabain | 2019 | Journal of psychiatric research | 113 | 181 to 189 | Brazil |
| Delaney et al. | Delaney | Breast Cancer Brain Metastasis Response to Radiation After Microbubble Oxygen Delivery in a Murine Model | 2019 | Journal of Ultrasound in Medicine | 38 | 3221 to 3228 | USA |
| Dreier et al. | Dreier | Correlates of Spreading Depolarization, Spreading Depression, and Negative Ultraslow Potential in Epidural Versus Subdural Electrocorticography | 2019 | Frontiers in Neuroscience | 13 | 373 | Germany |
| Du et al. | Du | PET imaging of metabolic changes after neural stem cells and GABA progenitor cells transplantation in a rat model of temporal lobe epilepsy | 2019 | European Journal of Nuclear Medicine and Molecular Imaging | 46 | 2392 to 2397 | China |
| Duveau et al. | Duveau | Pronounced antiepileptic activity of the subtype-selective GABAA -positive allosteric modulator PF-06372865 in the GAERS absence epilepsy model | 2019 | CNS Neuroscience & Therapeutics | 25 | 255 to 260 | France |
| Etter et al. | Etter | Optogenetic gamma stimulation rescues memory impairments in an Alzheimer's disease mouse model | 2019 | nature communications | 10 | 5322 | Canada |
| Ewell et al. | Ewell | The impact of pathological high-frequency oscillations on hippocampal network activity in rats with chronic epilepsy | 2019 | Elife | 8 | 42148 | USA |
| Farakhor et al. | Farakhor | Adaptation effects of medial forebrain bundle micro-electrical stimulation | 2019 | BIOENGINEERED | 10 | 78 to 86 | Iran |
| Farooq et al. | Farooq | Emergence of preconfigured and plastic time-compressed sequences in early postnatal development | 2019 | Neuroscience | 363 | 168 to 173 | USA |
| Fiath et al. | Fiath | Fine-scale mapping of cortical laminar activity during sleep slow oscillations using high-density linear silicon probes | 2019 | Journal of Neuroscience Methods | 316 | 58 to 70 | Hungary |
| Fortress et al. | Fortress | Experimental traumatic brain injury results in estrous cycle disruption, neurobehavioral deficits, and impaired GSK3beta/beta-catenin signaling in female rats | 2019 | Experimental Neurology | 315 | 42 to 51 | USA |
| Hu et al. | Hu | Effects of cellular prion protein on rapid eye movement sleep deprivation-induced spatial memory impairment | 2019 | Journal of integrative neuroscience | 18 | 439 to 444 | China |
| Ilieva et al. | Ilieva | Antidepressant agomelatine attenuates behavioral deficits and concomitant pathology observed in streptozotocin-induced model of Alzheimer's disease in male rats | 2019 | Hormones and Behavior | 107 | 11 to 19 | Bulgaria |
| Jackson et al. | Jackson | Intracerebroventricular Ghrelin Administration Increases Depressive-Like Behavior in Male Juvenile Rats | 2019 | frontiers in behavioral neuroscience | 13 | 77 | USA |
| Jakkamsetti et al. | Jakkamsetti | Brain metabolism modulates neuronal excitability in a mouse model of pyruvate dehydrogenase deficiency | 2019 | Science translational medicine | 11 | 457 | USA |
| Jakkamsetti et al. | Jakkamsetti | Brain metabolism modulates neuronal excitability in a mouse model of pyruvate dehydrogenase deficiency | 2019 | Science translational medicine | 11 | 457 | USA |
| Jermakowicz et al. | Jermakowicz | Cellular Changes in Injured Rat Spinal Cord Following Electrical Brainstem Stimulation | 2019 | brain sciences | 9 | 124 | USA |
| Kaefer et al. | Kaefer | Disrupted-in-schizophrenia 1 overexpression disrupts hippocampal coding and oscillatory synchronization | 2019 | Hippocampus | 29 | 802 to 816 | Austria |
| Katagiri et al. | Katagiri | Anti-seizure effect and neuronal activity change in the genetic-epileptic model rat with acute and chronic vagus nerve stimulation | 2019 | Epilepsy Research | 155 | 106159 | Japan |
| Kenny et al. | Kenny | Ferroptosis Contributes to Neuronal Death and Functional Outcome After Traumatic Brain Injury | 2019 | Critical Care Medicine | 47 | 410 to 418 | USA |
| Kim et al. B | Kim | Prefrontal D1 Dopamine-Receptor Neurons and Delta Resonance in Interval Timing | 2019 | Cerebral Cortex | 29 | 2051 to 2060 | USA |
| Kunori et al. | Kunori | An Implantable Cranial Window Using a Collagen Membrane for Chronic Voltage-Sensitive Dye Imaging | 2019 | Micromachines | 10 | 789 | Japan |
| Kyyriainen et al. | Kyyriainen | Deficiency of urokinase-type plasminogen activator and its receptor affects social behavior and increases seizure susceptibility | 2019 | Epilepsy Research | 151 | 67 to 74 | Finland |
| Lee et al. C | Lee | Fabrication of Convex PDMS-Parylene Microstructures for Conformal Contact of Planar Micro-Electrode Array | 2019 | polymers | 11 | 1436 | South Korea |
| Levata et al. | Levata | Nesfatin-1 Acts Centrally to Induce Sympathetic Activation of Brown Adipose Tissue and Non-Shivering Thermogenesis | 2019 | Hormone and Metabolica Research | 51 | 678 to 685 | Germany |
| Li et al. A | Li | Breviscapine provides a neuroprotective effect after traumatic brain injury by modulating the Nrf2 signaling pathway | 2019 | Journal of Cellular Biochemistry | 120 | 14899 to 14907 | China |
| Li et al. D | Li | Designing and Implementing a Novel Transcranial Electrostimulation System for Neuroplastic Applications: A Preliminary Study | 2019 | IEEE transactions on neural systems and rehabilitation engineering | 27 | 805 to 813 | Taiwan |
| Li et al. B | Li | Arginine vasopressin attenuates dysfunction of hippocampal theta and gamma oscillations in chronic cerebral hypoperfusion via V1a receptor | 2019 | Brain Research Bulletin | 153 | 84 to 92 | China |
| Luo et al. | Luo | Preso regulates NMDA receptor-mediated excitotoxicity via modulating nitric oxide and calcium responses after traumatic brain injury | 2019 | Cell Death and Disease | 10 | 496 | China |
| Lv et al. | Lv | Activation of the primary motor cortex using fully-implanted electrical sciatic nerve stimulation | 2019 | Experimental and therapeutic medicine | 18 | 3357 to 3364 | China |
| Ma et al. | Ma | Lactobacillus acidophilus Exerts Neuroprotective Effects in Mice with Traumatic Brain Injury | 2019 | The Journal of Nutrition and Disease | 149 | 1543 to 1552 | China |
| Mastrella et al. | Mastrella | Targeting APLN/APLNR Improves Antiangiogenic Efficiency and Blunts Proinvasive Side Effects of VEGFA/VEGFR2 Blockade in Glioblastoma | 2019 | Cancer Research | 79 | 2298 to 2313 | Germany |
| Mazza et al. | Mazza | Hampering brain tumor proliferation and migration using peptide nanofiber:siPLK1/MMP2 complexes | 2019 | Nanomedicine | 14 | 3127 to 3142 | UK |
| Mittal et al. | Mittal | CD4 T Cells Induce A Subset of MHCII-Expressing Microglia that Attenuates Alzheimer Pathology | 2019 | iScience | 16 | 298 to 311 | Israel |
| Mo et al. | Mo | Microglial P2Y12 Receptor Regulates Seizure-Induced Neurogenesis and Immature Neuronal Projections | 2019 | Neurobiology of Disease | 39 | 9453 to 9464 | USA |
| Mohammad et al. | Mohammad | Perampanel but Not Amantadine Prevents Behavioral Alterations and Epileptogenesis in Pilocarpine Rat Model of Status Epilepticus | 2019 | Molecular Neurobiology | 56 | 2508 to 2523 | Canada |
| Mohammadipoor-Ghasemabad et al. | Mohammadipoor-Ghasemabad | Hippocampal microRNA-191a-5p Regulates BDNF Expression and Shows Correlation with Cognitive Impairment Induced by Paradoxical Sleep Deprivation | 2019 | Neuroscience | 414 | 49 to 59 | Iran |
| Mohammadpoory et al. | Mohammadpoory | Complex network based models of ECoG signals for detection of induced epileptic seizures in rats | 2019 | Cognitive Neurodynamics | 13 | 325 to 339 | Iran |
| Moller et al. | Moller | Impact of repeated kindled seizures on heart rate rhythms, heart rate variability, and locomotor activity in rats | 2019 | Epilepsy & Behavior | 92 | 36 to 44 | Germany |
| Murai et al. | Murai | A novel GABAB receptor positive allosteric modulator, ASP8062, exerts analgesic effects in a rat model of fibromyalgia | 2019 | European Journal of Pharmacology | 865 | 17250 | Japan |
| Njoku et al. | Njoku | Chronic treatment with galantamine rescues reversal learning in an attentional set-shifting test after experimental brain trauma | 2019 | Experimental Neurology | 315 | 32 to 41 | USA |
| O'Brien et al. | O'Brien | Computer-based Multitaper Spectrogram Program for Electroencephalographic Data | 2019 | Journal of Visualized Experiments | 153 | 60333 | USA |
| Ogun et al. | Ogun | The effect of vortioxetine on penicillin-induced epileptiform activity in rats | 2019 | Arquivos de neuro-psiquiatria | 77 | 412 to 417 | Turkey |
| Okada et al. | Okada | Functional involvement of nucleus tractus solitarii neurons projecting to the parabrachial nucleus in trigeminal neuropathic pain | 2019 | Journal of Oral Sciences | 61 | 370 to 378 | Japan |
| Park et al. | Park | Longitudinal study of hemodynamics and dendritic membrane potential changes in the mouse cortex following a soft cranial window installation | 2019 | Neurophotonics | 6 | 15006 | South Korea |
| Pettibone et al. | Pettibone | Knock-In Rat Lines with Cre Recombinase at the Dopamine D1 and Adenosine 2a Receptor Loci | 2019 | eNeuro | 6 | n.r. | USA |
| Pflüger et al. | Pflüger | Chronically Implanted Microelectrodes Cause c-fos Expression Along Their Trajectory | 2019 | Frontiers in Neuroscience | 13 | 1367 | Germany |
| Qiao et al. | Qiao | Long-term characterization of activated microglia/macrophages facilitating the development of experimental brain metastasis through intravital microscopic imaging | 2019 | Journal of Neuroinflammation | 16 | 4 | China |
| Romoli et al. | Romoli | Hippocampal epileptogenesis in autoimmune encephalitis | 2019 | Annals of Clinical and Translational Neurology | 6 | 2261 to 2269 | Italy |
| Russell et al. | Russell | Time-dependent hemeoxygenase-1, lipocalin-2 and ferritin induction after non-contusion traumatic brain injury | 2019 | Brain Research | 1725 | 146466 | USA |
| Sa et al. | Sa | Cardiovascular and hidroelectrolytic changes in rats fed with high-fat diet | 2019 | Behavioral Brain Research | 373 | 112075 | Brazil |
| Sharma et al. | Sharma | Neuroprotective potential of solanesol in intracerebroventricular propionic acid induced experimental model of autism: Insights from behavioral and biochemical evidence | 2019 | Toxicology Reports | 6 | 1164 to 1175 | India |
| Shaver et al. | Shaver | Long-term deficits in risky decision-making after traumatic brain injury on a rat analog of the Iowa gambling task | 2019 | Brain Research | 1704 | 103 to 113 | USA |
| Shiuchi et al. | Shiuchi | Role of orexin in exercise-induced leptin sensitivity in the mediobasal hypothalamus of mice | 2019 | Biochemical and biophysical research communications | 514 | 166 to 172 | Japan |
| Simader et al. | Simader | Subarachnoid hemorrhage in rats - Visualizing blood distribution in vivo using gadolinium-enhanced magnetic resonance imaging: Technical note | 2019 | Journal of Neuroscience Methods | 325 | 108370 | Austria |
| Slezia et al. | Slezia | Electrophoretic Delivery of gamma-aminobutyric Acid (GABA) into Epileptic Focus Prevents Seizures in Mice | 2019 | Journal of Visualized Experiments | 147 | n.r. | France |
| Souza et al. | Souza | Contribution of the Retrotrapezoid Nucleus and Carotid Bodies to Hypercapnia- and Hypoxia-induced Arousal from Sleep | 2019 | The Journal of Neuroscience | 39 | 9725 to 9737 | USA |
| Souza et al. | Souza | Contribution of the Retrotrapezoid Nucleus and Carotid Bodies to Hypercapnia- and Hypoxia-induced Arousal from Sleep | 2019 | The Journal of Neuroscience | 39 | 9725 to 9737 | USA |
| Stanchi et al. | Stanchi | Imaging Glioma Progression by Intravital Microscopy | 2019 | Methods in Molecular Biology | 1862 | 227 to 243 | Belgium |
| Stanojlovic et al. | Stanojlovic | Chemogenetic activation of orexin/hypocretin neurons ameliorates aging-induced changes in behavior and energy expenditure | 2019 | American Journal of Physiology, regulatory, integrative and comparative physiology | 316 | 571 to 583 | USA |
| Sun et al. | Sun | In vivo Two-Photon Imaging of Anesthesia-Specific Alterations in Microglial Surveillance and Photodamage-Directed Motility in Mouse Cortex | 2019 | Frontiers in Neuroscience | 13 | 421 | Germany |
| Suzuki et al. | Suzuki | Noninvasive Vagus Nerve Stimulation Prevents Ruptures and Improves Outcomes in a Model of Intracranial Aneurysm in Mice | 2019 | Stroke | 50 | 1216 to 1223 | USA |
| Szonyi et al. | Szonyi | Median raphe controls acquisition of negative experience in the mouse | 2019 | Neuroscience | 366 | 6469 | Hungary |
| Szonyi et al. | Szonyi | Median raphe controls acquisition of negative experience in the mouse | 2019 | Neuroscience | 366 | 6469 | Hungary |
| Tomov et al. | Tomov | Roscovitine, an experimental CDK5 inhibitor, causes delayed suppression of microglial, but not astroglial recruitment around intracerebral dopaminergic grafts | 2019 | Experimental Neurology | 318 | 135 to 144 | Germany |
| Villa-Cedillo et al. | Villa-Cedillo | The mRVG-9R peptide as a potential therapeutic vector to the central nervous system cells | 2019 | Cell Biology International | 43 | 809 to 819 | Mexico |
| Villasana et al. | Villasana | Diazepam Inhibits Post-Traumatic Neurogenesis and Blocks Aberrant Dendritic Development | 2019 | Journal of Neurotrauma | 36 | 2454 to 2467 | USA |
| Wang et al. F | Wang | Transition from status epilepticus to interictal spiking in a rodent model of mesial temporal epilepsy | 2019 | Epilepsy Research | 152 | 73 to 76 | Canada |
| Wang et al. G | Wang | Early-life stress alters sleep structure and the excitatory-inhibitory balance in the nucleus accumbens in aged mice | 2019 | Chinese Medical Journal | 132 | 1582 to 1590 | China |
| Wang et al. A | Wang | Neuroprotective effect of l-serine against white matter demyelination by harnessing and modulating inflammation in mice | 2019 | Neuropharmacology | 146 | 39 to 49 | China |
| Wang et al. H | Wang | TRPV1 translocated to astrocytic membrane to promote migration and inflammatory infiltration thus promotes epilepsy after hypoxic ischemia in immature brain | 2019 | Journal of Neuroinflammation | 16 | 214 | China |
| Wang et al. B | Wang | Non-equilibrium critical dynamics of bursts in theta and delta rhythms as fundamental characteristic of sleep and wake micro-architecture | 2019 | PLoS Computational Biology | 15 | 1007268 | USA |
| Wang et al. D | Wang | Hippocampal low-frequency stimulation improves cognitive function in pharmacoresistant epileptic rats | 2019 | Epilepsy Research | 168 | 106194 | China |
| Wang et al. C | Wang | MiR-181b inhibits P38/JNK signaling pathway to attenuate autophagy and apoptosis in juvenile rats with kainic acid-induced epilepsy via targeting TLR4 | 2019 | CNS Neuroscience & Therapeutics | 25 | 112 to 122 | China |
| Wang et al. E | Wang | Levetiracetam Protects Against Cognitive Impairment of Subthreshold Convulsant Discharge Model Rats by Activating Protein Kinase C (PKC)-Growth-Associated Protein 43 (GAP-43)-Calmodulin-Dependent Protein Kinase (CaMK) Signal Transduction Pathway | 2019 | Medical Science Monitor | 25 | 4627 to 4638 | China |
| Wen et al. A | Wen | Investigating Alterations in Caecum Microbiota After Traumatic Brain Injury in Mice | 2019 | Journal of Visualized Experiments | 151 | n.r. | China |
| Wen et al. B | Wen | Inhibitory effects of glucagon-like peptide-1 receptor on epilepsy | 2019 | Biochemical and biophysical research communications | 511 | 79 to 86 | China |
| Xu et al. | Xu | Acute histopathological responses and long-term behavioral outcomes in mice with graded controlled cortical impact injury | 2019 | Neural Regeneration Research | 6 | 997 to 1003 | China |
| Yang et al. | Yang | Serum-Based Phospho-Neurofilament-Heavy Protein as Theranostic Biomarker in Three Models of Traumatic Brain Injury: An Operation Brain Trauma Therapy Study | 2019 | Journal of Neurotrauma | 36 | 348 to 359 | USA |
| Yeung et al. | Yeung | The Acute Effects of Amyloid-Beta1-42 on Glutamatergic Receptor and Transporter Expression in the Mouse Hippocampus | 2019 | Frontiers in Neuroscience | 13 | 1427 | New Zealand |
| Zhang et al. C | Zhang | Activation of noradrenergic terminals in the reticular thalamus delays arousal from propofol anesthesia in mice | 2019 | The FASEB Journal | 33 | 7252 to 7260 | China |
| Zhang et al. B | Zhang | A Small Molecule Spinogenic Compound Enhances Functional Outcome and Dendritic Spine Plasticity in a Rat Model of Traumatic Brain Injury | 2019 | Journal of Neurotrauma | 36 | 589 to 600 | USA |
| Zhang et al. A | Zhang | Cross-Species Investigation on Resting State Electroencephalogram | 2019 | Brain Topography | 32 | 808 to 824 | China |
| Zhao et al. | Zhao | Glial response in early stages of traumatic brain injury | 2019 | Neuroscience Letters | 708 | 134335 | China |

| study ID | experimental groups formed?  If so minimum number of animals per group? (n.a. OR number OR n.r.) | if experimental groups formed:  maximum numbers of animals per group?  (number OR n.a. OR n.r.) | n total of animals used (number OR n.r.) | background/ purpose of craniotomy / field of research (is the study related to on one or more specific neurological diseases or fundamental research?  E.g. Epilepsia, Parkinson’s disease, stroke, brain tumors, traumatic brain injury, migraine)  (name of neurological disease OR fundamental research OR n.r.) | species? (mice / rats OR both) | sex reported?  (female OR male OR both OR n.r.) | commercial breeder reported? If so, specify: (name of breeder OR n.r.) | strain?  (name OR n.r.) | age at surgery reported?  (weeks OR adult OR young OR n.r.) | body weight at surgery reported?  (x-x OR n.r. OR n.a.) | housing condition (pre surgery) reported, if so what?  (housed individually OR housed in groups OR n.r.) | information about cage reported? If so, specify (description OR n.r.) | housing temperature reported, if so which temperature (°C)?  (x-x OR n.r.) | housing humidity reported, if so how high (%)?  (x-x OR n.r.) | enrichment reported? If so, specify (enrichment OR n.r.) | Light schedule reported? If so, specify  (synchronized OR reversed OR n.r. OR other) |
| --- | --- | --- | --- | --- | --- | --- | --- | --- | --- | --- | --- | --- | --- | --- | --- | --- |
| Aldehri et al. | 7 | 10 | 17 | fundamental research | rats | n.r. | Charles River Sulzbach Germany | Sprague-Dawley | n.r. | 280 to 300 | housed in groups | n.r. | 20 to 22 | n.r. | n.r. | reversed |
| Asan et al. | n.a. | n.a. | 2 | fundamental research | rats | n.r. | n.r. | Sprague-Dawley | n.r. | n.r. | n.r. | n.r. | n.r. | n.r. | n.r. | n.r. |
| Baud et al. | 5 | 5 | 15 | epilepsy and seizures | rats | male | n.r. | Sprague-Dawley | n.r. | n.r. | n.r. | n.r. | n.r. | n.r. | n.r. | n.r. |
| Bazzu et al. | n.a. | n.a. | 8 | fundamental research | mice | male | Harlan Netherlands | C57BL/6 | n.r. | n.r. | n.r. | n.r. | 21 | n.r. | n.r. | reversed |
| Bertoglio et al. | 11 | 12 | 37 | epilepsy and seizures | rats | male | Charles River Laboratories France | Sprague-Dawley | 6 | n.r. | housed individually | n.r. | n.r. | n.r. | n.r. | synchronized |
| Bleimeister et al. | 5 | 10 | 60 | fundamental research | rats | male | Envigo RMS Inc. Indianapolis | Sprague-Dawley | adult | 300 to 325 | housed in groups | ventilated polycarbonate rat cage | 20 to 22 | n.r. | n.r. | synchronized |
| Bukhtiyarova et al. | n.r. | n.r. | 5 | fundamental research | mice | n.r. | n.r. | C57/BL6 | n.r. | n.r. | housed individually | n.r. | n.r. | n.r. | n.r. | synchronized |
| Burgdorf et al. | 9 | 10 | n.r. | epilepsy and seizures | rats | male | Envigo USA | Sprague-Dawley | n.r. | n.r. | n.r. | Lucite cage | n.r. | n.r. | n.r. | synchronized |
| Casanova-Carvajal et al. | 9 | 18 | 27 | brain tumors | mice | n.r. | no | C57BL/6 | adult | 34,25 to 36,27 | n.r. | n.r. | n.r. | n.r. | n.r. | synchronized |
| Chen et al. C | 10 | n.a. | 60 | fundamental research | mice | male | Yangzhou University Medical Center | ICR | n.r. | n.r. | n.r. | n.r. | n.r. | n.r. | n.r. | n.r. |
| Chen et al. E | 15 | 15 | 30 | fundamental research | mice | male | n.r. | C57BL/6 | n.r. | n.r. | housed in groups | n.r. | 21 to 25 | n.r. | n.r. | synchronized |
| Chen et al. A | n.r. | n.r. | n.r. | fundamental research | mice | both | Jackson Laboratory, MMRRC | Sox2f/f Aldh1l1-EGFP hGFAP-CreERt2 mGfap-Cre Rosa-tdTomato Rosa-YFP wildtype C57BL/6J | adult | n.r. | n.r. | n.r. | n.r. | n.r. | n.r. | synchronized |
| Chen et al. B | n.r. | n.r. | n.r. | brain stroke | mice | male | Jackson Laboratory Bar Harbour | C57BL/6 | 10 | n.r. | n.r. | n.r. | n.r. | n.r. | n.r. | n.r. |
| Chen et al. F | 6 | 6 | 216 | fundamental research | rats | male | Animal Experimental Center of Anhui Medical University | Sprague-Dawley | n.r. | n.r. | n.r. | n.r. | 20-22 | n.r. | n.r. | synchronized |
| Chitturi et al. | 6 | 10 | 36 | fundamental research | rats | male | Charles River Wilmington MA USA | Sprague-Dawley | 4,5 | n.r. | n.r. | n.r. | n.r. | n.r. | n.r. | n.r. |
| Christiaen et al. | 5 | 12 | 27 | fundamental research | rats | male | Envigo the netherlands | Sprague-Dawley | adult | n.r. | housed individually | type III H cage | 20 to 23 | 40 to 60 | n.r. | synchronized |
| Colangeli et al. | 7 | 25 | n.r. | epilepsy and seizures | rats | male | Charles River Italy | Sprague-Dawley | adult | n.r. | n.r. | n.r. | n.r. | n.r. | n.r. | synchronized |
| da Silva Pacheco et al. | n.r. | n.r. | 40 | fundamental research | rats | female | n.r. | Wistar | adult | n.r. | housed in groups | n.r. | n.r. | n.r. | n.r. | synchronized |
| Daglas et al. | 5 | 27 | n.r. | tbi | mice | male | Animal Resources Centre Western Australia, Australian Phenomics Facility Canberra, Monash Animal Research Platform | C57BL/6J, µ-MT, ß2m-/- | n.r. | n.r. | housed in groups | n.r. | n.r. | n.r. | n.r. | synchronized |
| Dal-Pont et al. | 8 | 8 | 48 | fundamental research | rats | male | n.r. | Wistar | n.r. | n.r. | housed in groups | n.r. | 21 to 23 | 45 to 55 | n.r. | synchronized |
| Delaney et al. | 3 | 3 | 15 | brain tumors | mice | female | Charles River Wilmington MA USA | immunodeficient nude | n.r. | n.r. | n.r. | n.r. | n.r. | n.r. | n.r. | n.r. |
| Dreier et al. | n.a. | n.a. | 8 | fundamental research | rats | male | Charles River Germany | Wistar | n.r. | 250 to 400 | n.r. | n.r. | n.r. | n.r. | n.r. | n.r. |
| Du et al. | 5 | 8 | n.r. | epilepsy and seizures | rats | male | n.r. | Sprague-Dawley | adult | n.r. | n.r. | n.r. | n.r. | n.r. | n.r. | n.r. |
| Duveau et al. | n.r. | n.r. | n.r. | epilepsy and seizures | rats | male | INSERM Grenoble Institute of Neurosciences Grenoble France | GAERS | n.r. | n.r. | housed in groups | n.r. | 20 to 24 | n.r. | n.r. | synchronized |
| Etter et al. | 6 | 31 | 87 | Alzheimer’s disease | mice | male | n.r. | PVJ20 | n.r. | n.r. | n.r. | n.r. | n.r. | n.r. | n.r. | n.r. |
| Ewell et al. | 4 | 4 | 8 | epilepsy and seizures | rats | male | Charles River Labs | Wistar | n.r. | n.r. | housed individually | n.r. | n.r. | n.r. | n.r. | reversed |
| Farakhor et al. | n.r. | n.r. | 6 | fundamental research | rats | male | n.r. | Wistar | adult | n.r. | housed individually | n.r. | n.r. | n.r. | n.r. | n.r. |
| Farooq et al. | 3 | n.r. | 22 | fundamental research | rats | male | n.r. | Long-Evans | 2 to 3,5  adult | n.r. | housed in groups | n.r. | n.r. | n.r. | n.r. | synchronized |
| Fiath et al. | n.r. | n.r. | 6 | fundamental research | rats | n.r. | n.r. | Wistar | n.r. | 270 to 650 | n.r. | n.r. | n.r. | n.r. | n.r. | n.r. |
| Fortress et al. | 3 | 7 | 22 | tbi | rats | female | Charles River Raleigh NC USA | Sprague-Dawley | n.r. | n.r. | housed individually | n.r. | n.r. | n.r. | PVC tube | synchronized |
| Hu et al. | n.r. | n.r. | n.r. | fundamental research | mice | male | n.r. | C57BL/6 | n.r. | n.r. | housed in groups | plexiglass cage | 22 to 24 | 50 to 60 | n.r. | synchronized |
| Ilieva et al. | 11 | 13 | 40 | Alzheimer’s disease | rats | male | Institute of Neurobiology Bulgarian Academy of Sciences | Sprague-Dawley | n.r. | n.r. | housed in groups | n.r. | 20 | 50 to 60 | n.r. | synchronized |
| Jackson et al. | 6 | 16 | 72 | fundamental research | rats | male | n.r. | Sprague-Dawley | 3 | n.r. | housed individually | n.r. | 20 to 22 | 46 | n.r. | synchronized |
| Jakkamsetti et al. | 4 | 8 | 36 | fundamental research | mice | male | n.r. | PDHD, control | 3 to 4 | n.r. | n.r. | n.r. | n.r. | n.r. | n.r. | n.r. |
| Jakkamsetti et al. | 4 | 8 | 36 | fundamental research | mice | male | n.r. | PDHD, control | 3 to 4 | n.r. | n.r. | n.r. | n.r. | n.r. | n.r. | n.r. |
| Jermakowicz et al. | 10 | 11 | 21 | fundamental research | rats | female | n.r. | Sprague-Dawley | young | n.r. | n.r. | n.r. | n.r. | n.r. | n.r. | n.r. |
| Kaefer et al. | 2 | 2 | 8 | schizophrenia | rats | male | n.r. | tgDISC1, wildtype | n.r. | n.r. | n.r. | n.r. | n.r. | n.r. | n.r. | n.r. |
| Katagiri et al. | n.r. | n.r. | 24 | epilepsy and seizures | rats | both | National Bioresource Project-Rat Kyoto University Kyoto Japan | Noda Epileptic rat (NER) | n.r. | n.r. | n.r. | n.r. | n.r. | 55 | n.r. | n.r. |
| Kenny et al. | 3 | 10 | n.r. | tbi | mice | male | Jackson Laboratory Bar Harbour | C57BL/6 | adult | n.r. | n.r. | n.r. | n.r. | n.r. | n.r. | n.r. |
| Kim et al. B | 6 | 6 | 24 | fundamental research | mice | n.r. | n.r. | D1-Cre+ | n.r. | n.r. | housed individually | n.r. | n.r. | n.r. | n.r. | synchronized |
| Kunori et al. | n.a. | n.a. | 7 | fundamental research | rats | male | SLC Inc. Tokyo Japan | Wistar | n.r. | 300 to 350 | housed in groups | n.r. | n.r. | n.r. | n.r. | n.r. |
| Kyyriainen et al. | 10 | 13 | 23 | epilepsy and seizures | mice | both | n.r. | Plau/Plaur-double-knockout, wildtype | adult | n.r. | n.r. | n.r. | 21 to 23 | n.r. | n.r. | synchronized |
| Lee et al. C | n.a. | n.a. | 4 | fundamental research | rats | male | n.r. | Sprague-Dawley | n.r. | 300 to 350 | n.r. | n.r. | n.r. | n.r. | n.r. | n.r. |
| Levata et al. | n.r. | n.r. | n.r. | fundamental research | mice | male | Charles River Sulzfeld Germany | C57BL/6J | n.r. | n.r. | n.r. | individually ventilated cage | 21 | n.r. | n.r. | synchronized |
| Li et al. A | 6 | 18 | 237 | tbi | rats | male | Experimental Animal Center of Nanjing Medical University | Sprague-Dawley | n.r. | n.r. | n.r. | n.r. | 23,5 to 24,5 | n.r. | n.r. | n.r. |
| Li et al. D | 7 | 7 | 14 | fundamental research | rats | male | n.r. | Wistar | n.r. | 300 to 350 | n.r. | n.r. | n.r. | n.r. | n.r. | n.r. |
| Li et al. B | n.r. | n.r. | n.r. | fundamental research | rats | male | Experimental Animal Center of the Chinese Academy Medical Scinces Beijing China | Wistar | adult | n.r. | housed in groups | n.r. | 23 to 25 | 50 to 60 | n.r. | n.r. |
| Luo et al. | 10 | 12 | 42 | tbi | mice | n.r. | Experimental Center of Fourth Military Medical University | C57BL/6J | 11 to 13 | n.r. | n.r. | n.r. | 27 | n.r. | n.r. | n.r. |
| Lv et al. | n.a. | n.a. | 13 | fundamental research | rats | male | n.r. | Sprague-Dawley | n.r. | n.r. | n.r. | n.r. | 30 | 55,3 | n.r. | synchronized |
| Ma et al. | n.r. | n.r. | n.r. | tbi | mice | male | n.r. | C57BL/6 | n.r. | n.r. | n.r. | n.r. | n.r. | n.r. | n.r. | n.r. |
| Mastrella et al. | n.r. | n.r. | n.r. | brain tumors | mice | n.r. | n.r. |  | n.r. | n.r. | n.r. | n.r. | n.r. | n.r. | n.r. | n.r. |
| Mazza et al. | 4 | 5 | 9 | brain tumors | mice | n.r. | Harlan UK | Athymic nude | 4 | n.r. | n.r. | n.r. | n.r. | n.r. | n.r. | n.r. |
| Mittal et al. | 4 | 7 | 39 | fundamental research | mice | female | n.r. | 5XFAD/MHCII-/- | n.r. | n.r. | n.r. | n.r. | n.r. | n.r. | n.r. | n.r. |
| Mo et al. | 12 | n.r. | n.r. | epilepsy and seizures | mice | male | Jackson Laboratory | C57B/6, CX3CR1-GFP +/-, P2Y12Rfl/fl | 8 to 10 | n.r. | n.r. | n.r. | n.r. | n.r. | n.r. | n.r. |
| Mohammad et al. | 8 | 38 | 139 | epilepsy and seizures | rats | male | n.r. | Sprague-Dawley | adult | n.r. | housed in groups | polypropylene | 21 | n.r. | n.r. | n.r. |
| Mohammadipoor-Ghasemabad et al. | 5 | 7 | 114 | fundamental research | rats | female | n.r. | Wistar | 16 to 20 | n.r. | housed in groups | n.r. | 22 to 24 | n.r. | n.r. | n.r. |
| Mohammadpoory et al. | 12 | 15 | 27 | epilepsy and seizures | rats | male | n.r. | Wistar | n.r. | n.r. | n.r. | n.r. | 21 to 23 | n.r. | n.r. | synchronized |
| Moller et al. | 6 | 6 | 13 | epilepsy and seizures | rats | female | Envigo the Netherlands | Sprague-Dawley | n.r. | n.r. | housed individually | Macrolon Eurostandard Type III | 20 to 24 | 45 to 65 | embedding, nesting material | synchronized |
| Murai et al. | 7 | 8 | n.r. | fundamental research | rats | male | Japan SLC Hamamatsu, Charles River Laboratories Japan Yokohama Japan | Sprague-Dawley | n.r. | n.r. | housed in groups | n.r. | 21 to 25 | 40 to 70 | n.r. | n.r. |
| Njoku et al. | n.r. | n.r. | 59 | tbi | rats | male | Harlan Indianapolis USA | Sprague-Dawley | n.r. | 300 to 325 | n.r. | plexiglass cage | 20 to 22 | n.r. | n.r. | synchronized |
| O'Brien et al. | n.r. | n.r. | n.r. | fundamental research | mice | n.r. | n.r. | n.r. | n.r. | n.r. | n.r. | n.r. | n.r. | n.r. | n.r. | n.r. |
| Ogun et al. | 9 | 9 | 27 | epilepsy and seizures | rats | male | Abant Izzet Baysal University Experimental Animals Research Center Bolu Turkey | Wistar | n.r. | n.r. | housed in groups | n.r. | 20 to 24 | n.r. | n.r. | synchronized |
| Okada et al. | 4 | 4 | 21 | fundamental research | rats | male | n.r. | Sprague-Dawley | adult | n.r. | n.r. | n.r. | 23 | n.r. | n.r. | synchronized |
| Park et al. | n.a. | n.a. | 80 | fundamental research | mice | male | n.r. | C57BL/6 | n.r. | n.r. | n.r. | n.r. | n.r. | n.r. | n.r. | n.r. |
| Pettibone et al. | n.a. | n.a. | 4 | fundamental research | rats | both | n.r. | Adora2a, Drd1a | n.r. | n.r. | n.r. | n.r. | n.r. | n.r. | n.r. | n.r. |
| Pflüger et al. | n.a. | n.a. | 15 | fundamental research | rats | female | n.r. | Sprague-Dawley | n.r. | 290 to 330 | n.r. | n.r. | 22 | 40 | n.r. | synchronized |
| Qiao et al. | 6 | 7 | n.r. | brain tumors | mice | female | Jackson Laboratory Bar Harbour USA, Hunan Slack King of Laboratory Animal Co. Changsha China | CX3CR1-GFP, C57BL/6 | n.r. | n.r. | n.r. | n.r. | n.r. | n.r. | n.r. | n.r. |
| Romoli et al. | n.r. | n.r. | n.r. | epilepsy and seizures | mice | male | n.r. | C57BL/6J | n.r. | n.r. | n.r. | n.r. | n.r. | n.r. | n.r. | n.r. |
| Russell et al. | n.r. | n.r. | 47 | tbi | rats | male | Harlan Laboratories | Sprague-Dawley | n.r. | n.r. | housed in groups | n.r. | 22 | 66 | n.r. | n.r. |
| Sa et al. | 5 | 8 | n.r. | fundamental research | rats | male | UNESP Araraqara | Holtzman Sprague-Dawley | adult | n.r. | housed individually | n.r. | 21 to 25 | 45 to 65 | n.r. | synchronized |
| Sharma et al. | 7 | 7 | 59 | fundamental research | rats | both | Central Animal House ISF College of Pharmacy Moga Punjab India | Wistar | n.r. | n.r. | n.r. | polyacrylic cage | n.r. | n.r. | soft bedding | n.r. |
| Shaver et al. | 23 | 24 | 47 | tbi | rats | male | Charles River Wilmington MA USA | Long-Evans | 16 | n.r. | housed individually | n.r. | n.r. | n.r. | n.r. | reversed |
| Shiuchi et al. | 3 | 3 | n.r. | fundamental research | mice | male | Japan SLC Shizuoka | C57BL/6J | n.r. | n.r. | housed individually | n.r. | 22 to 24 | n.r. | n.r. | synchronized |
| Simader et al. | 1 | 7 | 11 | fundamental research | rats | male | Department of Biomedical Research Medical University of Vienna Himberg Austria | Sprague-Dawley | adult | 300 to 400 | n.r. | n.r. | n.r. | n.r. | n.r. | n.r. |
| Slezia et al. | n.r. | n.r. | 17 | epilepsy and seizures | mice | male | n.r. | OF1 | adult | n.r. | n.r. | n.r. | n.r. | n.r. | n.r. | n.r. |
| Souza et al. | n.r. | n. | 46 | fundamental research | rats | male | Taconic Biosciences, Horizon Discovery | Sprague-Dawley, Th-cre rats | adult | 275 to 300 | n.r. | n.r. | 23 to 24 | n.r. | n.r. | n.r. |
| Souza et al. | n.r. | n.r. | 46 | fundamental research | rats | male | Taconic Biosciences, Horizon Discovery | Sprague-Dawley, Th-cre rats | adult | 275 to 300 | n.r. | n.r. | 23 to 24 | n.r. | n.r. | n.r. |
| Stanchi et al. | n.r. | n.r. | n.r. | brain tumors | mice | n.r. | n.r. | n.r. | n.r. | n.r. | n.r. | n.r. | n.r. | n.r. | n.r. | n.r. |
| Stanojlovic et al. | 8 | 10 | 35 | fundamental research | mice | female | in house | C57BL6/6J, orexin/Cre | adult | n.r. | n.r. | n.r. | n.r. | n.r. | n.r. | n.r. |
| Sun et al. | n.r. | n.r. | 7 | fundamental research | mice | male | Jackson Laboratory | CX3CRI-GFP heterozygous C57BL/6CRL | n.r. | n.r. | housed individually | n.r. | n.r. | n.r. | n.r. | n.r. |
| Suzuki et al. | 10 | 14 | 50 | fundamental research | mice | male | Charles River Wilmington MA | C57BL/6 | n.r. | n.r. | n.r. | n.r. | n.r. | n.r. | n.r. | n.r. |
| Szonyi et al. | 2 | 14 | n.r. | fundamental research | mice | both | n.r. | vGluT2-iRES-Cre, vGAT-iRES-Cre, BAC-vGluT3/iCre, TpH2/iCre-ERT2, C57BL/6 | adult | n.r. | housed in groups | n.r. | n.r. | n.r. | n.r. | n.r. |
| Szonyi et al. | 2 | 14 | n.r. | fundamental research | mice | both | n.r. | vGluT2-iRES-Cre, vGAT-iRES-Cre, BAC-vGluT3/iCre, TpH2/iCre-ERT2, C57BL/6 | adult | n.r. | housed in groups | n.r. | n.r. | n.r. | n.r. | n.r. |
| Tomov et al. | 8 | 8 | 64 | fundamental research | rats | male | Charles River Laboratories Germany | Sprague-Dawley | adult | n.r. | n.r. | n.r. | 20 to 24 | n.r. | n.r. | n.r. |
| Villa-Cedillo et al. | n.r. | n.r. | 42 | fundamental research | mice | n.r. | n.r. | C57BL/6 | 10 | 23 to 27 | n.r. | n.r. | n.r. | n.r. | n.r. | n.r. |
| Villasana et al. | 6 | 16 | 131 | tbi | mice | both | n.r. | C57BL/6J, POMC-GFP | n.r. | n.r. | n.r. | n.r. | n.r. | n.r. | n.r. | n.r. |
| Wang et al. F | n.a. | n.a. | 5 | epilepsy and seizures | mice | n.r. | Jackson Laboratory | C57BL/6 | 8 to 14 | n.r. | n.r. | n.r. | 20 to 24 | n.r. | n.r. | n.r. |
| Wang et al. G | n.a. | n.a. | 10 | fundamental research | mice | male | n.r. | C57BL/6N | 64 to 68 | n.r. | n.r. | n.r. | 22 to 24 | n.r. | n.r. | synchronized |
| Wang et al. A | 12 | 12 | 60 | fundamental research | mice | male | Experimental Animal Center of Nantong University | C57/BL6 | 10 to 12 | n.r. | housed in groups | n.r. | n.r. | n.r. | n.r. | n.r. |
| Wang et al. H | 28 | 28 | 112 | fundamental research | mice | n.r. | Jackson Laboratory | B6.129X1-TRPV1 KO | 1,5 | n.r. | n.r. | n.r. | n.r. | n.r. | n.r. | n.r. |
| Wang et al. B | 5 | 10 | 20 | fundamental research | rats | male | Harlan | Sprague-Dawley | adult | n.r. | n.r. | n.r. | n.r. | n.r. | n.r. | n.r. |
| Wang et al. D | 6 | 12 | 100 | epilepsy and seizures | rats | male | n.r. | Sprague-Dawley | adult | n.r. | housed individually | galvanized mesh cage | n.r. | n.r. | n.r. | synchronized |
| Wang et al. C | n.r. | n.r. | 47 | epilepsy and seizures | rats | both | Shanghai SLAC Laboratory Animal Co Ltd. Shanghai China | Wistar | n.r. | n.r. | n.r. | n.r. | 22 to 25 | n.r. | n.r. | n.r. |
| Wang et al. E | n.r. | n.r. | 64 | epilepsy and seizures | rats | male | Experimental Animal Center of Chongqing Medical University China | specific-pathogen-free Sprague-Dawley | n.r. | n.r. | n.r. | n.r. | n.r. | n.r. | n.r. | n.r. |
| Wen et al. A | n.r. | n.r. | n.r. | tbi | mice | male | n.r. | C57BL/6J | 5 to 6 | 20 to 25 | n.r. | n.r. | n.r. | n.r. | n.r. | synchronized |
| Wen et al. B | 6 | 10 | n.r. | epilepsy and seizures | rats | male | Experimental Animal Center of Chongqing Medical University China | Sprague-Dawley | adult | n.r. | n.r. | n.r. | n.r. | n.r. | n.r. | synchronized |
| Xu et al. | 18 | 18 | 152 | tbi | mice | male | Shanghai Research Center for Model Organisms China | C57BL/6 | n.r. | n.r. | housed in groups | n.r. | 24 | 50 | n.r. | synchronized |
| Yang et al. | 20 | 25 | 184 | tbi | rats | male | Charles River Wilmington MA Harlan (Indianapolis IN) | Sprague-Dawley | n.r. | n.r. | n.r. | n.r. | 22 | n.r. | n.r. | synchronized |
| Yeung et al. | 6 | 12 | 30 | Alzheimer’s disease | mice | male | n.r. | C57BL/6 | 64 | n.r. | n.r. | n.r. | n.r. | n.r. | n.r. | n.r. |
| Zhang et al. C | 10 | 10 | n.r. | fundamental research | mice | male | Shanghai Model Organisms Center Shanghai China | C57BL/6J, TH-IHRES-Cre, Vgat-IHRES-Cre | n.r. | n.r. | n.r. | n.r. | 22 to 25 | n.r. | n.r. | n.r. |
| Zhang et al. B | 7 | 7 | 14 | tbi | rats | male | Charles River Wilmington MA | Wistar | n.r. | n.r. | n.r. | n.r. | n.r. | n.r. | n.r. | n.r. |
| Zhang et al. A | n.a. | n.a. | 8 | fundamental research | rats | male | n.r. | Sprague-Dawley | adult | 300 to 400 | housed individually | n.r. | n.r. | n.r. | n.r. | reversed |
| Zhao et al. | n.r. | n.r. | 24 | tbi | rats | male | Experimental Animal Center of Xi'an Jiaotong University | Sprague-Dawley | n.r. | n.r. | n.r. | n.r. | 20-25 | n.r. | n.r. | synchronized |

| study ID | handling technique pre surgery reported? If so, specify (handling technique OR n.r.) | duration of surgery reported?  (x minutes OR n.r.) | survival surgery?  (did animals wake up from anesthesia?)  (yes / no OR n.r.) | how long did they live after surgery approximately?  (<1 day, <1 week, <2 weeks, <4 weeks, <8 weeks, >8 weeks OR n.a. OR n.r.) | mortality during surgery reported?  (n.r. OR number) | fate of the used animals? (killed OR n.r. OR reused) | type of surgical procedure  (name) | if "other", specify:  (name OR n.a.) | if applicable:  implantation site (name OR n.a. OR n.r.) | insult size reported? (mm diameter OR n.r.) | trepanning size reported? (mm diameter OR n.r.) | no permanent implant? If permanent implant, number of anchoring screws reported?  (nonpermanent implant OR number of anchoring screws OR n.r.) | model used? if so, what kind of model?  e.g. TBI, CCI, fluid percussion injury  (model OR n.a.) |
| --- | --- | --- | --- | --- | --- | --- | --- | --- | --- | --- | --- | --- | --- |
| Aldehri et al. | n.r. | n.r. | yes | <8 weeks | n.r. | killed | deep electrode implantation | n.a. | fornix | n.r. | n.r. | n.r. | n.a. |
| Asan et al. | n.r. | n.r. | n.r. | n.r. | n.r. | n.r. | deep electrode implantation | n.a. | cortex | n.r. | 2x2 | n.r. | n.a. |
| Baud et al. | n.r. | n.r. | yes | >8weeks | n.r. | n.r. | surface (screw) electrode implantation | n.a. | hippocampus | n.r. | n.r. | n.r. | n.a. |
| Bazzu et al. | handled daily for 8 days with plastic tube | n.r. | yes | <2 weeks | n.r. | killed | other | oxygen microsensor implantation, stimulating electrode implantation | cortex | n.r. | n.r. | 3 | n.a. |
| Bertoglio et al. | n.r. | n.r. | yes | >8weeks | n.r. | killed | surface (screw) and deep electrode implantation | n.a. | cortex | n.r. | n.r. | n.r. | n.a. |
| Bleimeister et al. | n.r. | n.r. | yes | <4 weeks | n.r. | killed | TBI, cci | n.a. | n.r. | 6 | 6 | nonpermanent implant | cci |
| Bukhtiyarova et al. | n.r. | n.r. | yes | <4 weeks | n.r. | n.r. | deep electrode implantation | n.a. | cortex | n.r. | n.r. | n.r. | n.a. |
| Burgdorf et al. | n.r. | 15-20 | yes | <2 weeks | n.r. | killed | surface (screw) electrode implantation | n.a. | n.r. | n.r. | n.r. | n.r. | n.a. |
| Casanova-Carvajal et al. | n.r. | n.r. | yes | <8 weeks | n.r. | killed | intracerebral injection cannula | n.a. | cortex | n.r. | n.r. | nonpermanent implant | n.a. |
| Chen et al. C | n.r. | n.r. | yes | <4 weeks | n.r. | killed | intracerebral injection cannula | n.a. | dentate gyrus | n.r. | n.r. | nonpermanent implant | n.a. |
| Chen et al. E | n.r. | n.r. | yes | <8 weeks | n.r. | killed | other | intracerebroventricular injection, fiber-optic cannula implantation | paraventricular nucleus of the thalamus, amygdala | n.r. | n.r. | n.r. | n.a. |
| Chen et al. A | n.r. | n.r. | yes | <8 weeks | n.r. | killed | TBI, cci | n.a. | cortex | 2 | 2,2 | nonpermanent implant | cci |
| Chen et al. B | n.r. | n.r. | yes | <2 weeks | n.r. | killed | intracerebroventricular guide cannula implantation | n.a. | left lateral ventricle | n.r. | n.r. | n.r. | n.a. |
| Chen et al. F | n.r. | n.r. | yes | <4 weeks | n.r. | killed | other | tbi controlled cortical impact, intracerebral injection | n.a. | 4,5 | 7 | nonpermanent implant | cci |
| Chitturi et al. | n.r. | n.r. | yes | <1 week | 4 | killed | TBI, fluid percussion | n.a. | n.r. | n.r. | 3 | n.r. | fluid percussion |
| Christiaen et al. | n.r. | n.r. | yes | n.r. | n.r. | n.r. | electrode implantation | n.a. | hippocampus | n.r. | n.r. | n.r. | n.a. |
| Colangeli et al. | n.r. | n.r. | yes | n.r. | n.r. | killed | surface (screw) electrode implantation | n.a. | n.r. | n.r. | n.r. | n.r. | n.a. |
| da Silva Pacheco et al. | n.r. | n.r. | yes | <4 weeks | n.r. | killed | intracerebral injection cannula | n.a. | periaqueductal grey matter | n.r. | n.r. | nonpermanent implant | n.a. |
| Daglas et al. | n.r. | n.r. | yes | n.r. | n.r. | killed | TBI, cci | n.a. | n.r. | n.r. | 5 | nonpermanent implant | cci |
| Dal-Pont et al. | n.r. | n.r. | yes | <1 week | n.r. | killed | intracerebroventricular guide cannula implantation | n.a. | lateral brain ventricle | n.r. | 0,36 | n.r. | n.a. |
| Delaney et al. | n.r. | n.r. | yes | n.r. | n.r. | killed | intracerebral injection cannula | n.a. | right hemisphere | n.r. | n.r. | nonpermanent implant | n.a. |
| Dreier et al. | n.r. | n.r. | no | n.a. | n.r. | killed | electrode implantation | n.a. | cortex | n.r. | n.r. | n.r. | n.a. |
| Du et al. | n.r. | n.r. | yes | <8 weeks | n.r. | killed | other | double intracerebral guide cannula implantation, intracerebral injection | dorsal hippocampus | n.r. | n.r. | n.r. | n.a. |
| Duveau et al. | n.r. | n.r. | yes | <2 weeks | n.r. | killed | surface (screw) electrode implantation | n.a. | n.r. | n.r. | 1 | n.r. | n.a. |
| Etter et al. | n.r. | n.r. | yes | n.r. | n.r. | killed | other | intracerebral injection cannula, fiber optic implantation | medial septum, hippocampus | 0,2 | n.r. | n.r. | n.a. |
| Ewell et al. | n.r. | n.r. | yes | n.r. | n.r. | n.r. | electrode implantation | n.a. | right hippocampus, cortex | n.r. | n.r. | 16 | n.a. |
| Farakhor et al. | n.r. | n.r. | yes | n.r. | n.r. | killed | surface (screw) and deep electrode implantation | n.a. | cortex, lateral hypothalamus | 1 | n.r. | n.r. | n.a. |
| Farooq et al. | n.r. | n.r. | yes | n.r. | n.r. | n.r. | deep electrode implantation | n.a. | hippocampus | n.r. | n.r. | n.r. | n.a. |
| Fiath et al. | n.r. | n.r. | no | n.a. | n.r. | killed | deep electrode implantation | n.a. | neocortex | n.r. | 3x3 | n.r. | n.a. |
| Fortress et al. | n.r. | n.r. | yes | >8weeks | 3 | killed | TBI, fluid percussion | n.a. | parietal cortex | n.r. | 4 | n.r. | fluid percussion |
| Hu et al. | n.r. | n.r. | yes | n.r. | n.r. | killed | intracerebral injection cannula | n.a. | hippocampus | n.r. | n.r. | nonpermanent implant | n.a. |
| Ilieva et al. | n.r. | n.r. | yes | <8 weeks | n.r. | killed | intracerebroventricular guide cannula implantation | n.a. | lateral brain ventricle | n.r. | n.r. | n.r. | n.a. |
| Jackson et al. | n.r. | n.r. | yes | <4 weeks | n.r. | killed | intracerebroventricular injection cannula | n.a. | lateral brain ventricle | n.r. | n.r. | nonpermanent implant | n.a. |
| Jakkamsetti et al. | n.r. | n.r. | yes | n.r. | n.r. | n.r. | deep electrode implantation | n.a. | barrel cortex | n.r. | n.r. | n.r. | n.a. |
| Jakkamsetti et al. | n.r. | n.r. | yes | n.r. | n.r. | n.r. | surface (screw) and deep electrode implantation | n.a. | n.r. | n.r. | n.r. | n.r. | n.a. |
| Jermakowicz et al. | n.r. | n.r. | yes | <1 week | n.r. | killed | deep electrode implantation | n.a. | nucleus raphe magnus | n.r. | n.r. | n.r. | n.a. |
| Kaefer et al. | n.r. | n.r. | yes | n.r. | n.r. | n.r. | deep electrode implantation | n.a. | right dorsal CA1 region | n.r. | n.r. | 7 to 8 | n.a. |
| Katagiri et al. | n.r. | n.r. | yes | n.r. | n.r. | killed | electrode implantation | n.a. | right dorsal hippocampus, skull surface | n.r. | n.r. | n.r. | n.a. |
| Kenny et al. | n.r. | n.r. | yes | n.r. | n.r. | killed | TBI, cci | n.a. | right cortex | 3 | n.r. | nonpermanent implant | cci |
| Kim et al. B | n.r. | n.r. | yes | n.r. | n.r. | killed | other | intracerebral injection, optical fiber cannula implantation | medial frontal cortex | n.r. | n.r. | n.r. | n.a. |
| Kunori et al. | n.r. | n.r. | n.r. | n.a. | n.r. | killed | other | cranial window device implantation | over secondary motor cortex | n.r. | 3,5 | n.r. | n.a. |
| Kyyriainen et al. | n.r. | n.r. | yes | <4 weeks | 3 | killed | surface (screw) and deep electrode implantation | n.a. | parietal cortex | 1 | n.r. | n.r. | n.a. |
| Lee et al. C | n.r. | n.r. | n.r. | n.a. | n.r. | n.r. | sole craniotomy | n.a. | olfactory bulb | n.r. | n.r. | nonpermanent implant | n.a. |
| Levata et al. | n.r. | n.r. | yes | n.r. | n.r. | n.r. | intracerebroventricular guide cannula implantation | n.a. | lateral brain ventricle | 0,4 | 0,4 | n.r. | n.a. |
| Li et al. A | n.r. | n.r. | yes | <2 weeks | 27 | killed | TBI | n.a. | left parietal cortex | n.r. | 6 | nonpermanent implant | tbi |
| Li et al. D | n.r. | n.r. | n.r. | n.r. | n.r. | n.r. | deep electrode implantation | n.a. | primary motor cortex | n.r. | n.r. | n.r. | n.a. |
| Li et al. B | n.r. | n.r. | no | n.a. | n.r. | killed | deep recording electrode implantation | n.a. | hippocampus | n.r. | n.r. | nonpermanent implant | n.a. |
| Luo et al. | n.r. | n.r. | yes | n.r. | n.r. | killed | intracerebral injection cannula | n.a. | right hemisphere | n.r. | n.r. | nonpermanent implant | cci |
| Lv et al. | n.r. | n.r. | yes | <8 weeks | n.r. | n.r. | electrode implantation | n.a. | n.r. | n.r. | n.r. | n.r. | n.a. |
| Ma et al. | n.r. | n.r. | yes | <2 weeks | n.r. | killed | tbi | n.a. | n.r. | 3 | 2x2 | nonpermanent implant | tbi |
| Mastrella et al. | n.r. | n.r. | yes | n.r. | n.r. | killed | other | intracerebral injection cannula, intracerebroventricular guide cannula implantation | n.r. | n.r. | n.r. | n.r. | n.a. |
| Mazza et al. | n.r. | n.r. | yes | n.r. | n.r. | n.r. | intracerebral injection cannula | n.a. | striatum | n.r. | n.r. | nonpermanent implant | n.a. |
| Mittal et al. | n.r. | n.r. | yes | <4 weeks | n.r. | killed | intracerebroventricular injection cannula | n.a. | lateral brain ventricle | n.r. | n.r. | nonpermanent implant | n.a. |
| Mo et al. | n.r. | n.r. | yes | n.r. | n.r. | killed | intracerebroventricular injection cannula | n.a. | n.r. | n.r. | n.r. | nonpermanent implant | n.a. |
| Mohammad et al. | n.r. | n.r. | yes | <8 weeks | n.r. | killed | deep electrode implantation | n.a. | hippocampus, cortex, cerebellum | 0,28 | n.r. | n.r. | n.a. |
| Mohammadipoor-Ghasemabad et al. | n.r. | n.r. | yes | <2 weeks | n.r. | killed | intracerebroventricular guide cannula implantation | n.a. | left brain ventricle, right brain ventricle | 0,64 | n.r. | n.r. | n.a. |
| Mohammadpoory et al. | handled gently | n.r. | yes | n.r. | n.r. | n.r. | surface (screw) electrode implantation | n.a. | parietal cortex, occipital cortex | n.r. | n.r. | 3 | n.a. |
| Moller et al. | n.r. | n.r. | yes | <4 weeks | 2 | killed | deep electrode implantation | n.a. | basolateral amygdala | n.r. | n.r. | n.r. | n.a. |
| Murai et al. | n.r. | n.r. | yes | n.r. | n.r. | n.r. | deep recording electrode implantation | n.a. | frontal cortex, parietal cortex | n.r. | n.r. | n.r. | n.a. |
| Njoku et al. | n.r. | n.r. | yes | <8 weeks | n.r. | killed | tbi | n.a. | parietal cortex | 6 | n.r. | nonpermanent implant | tbi |
| O'Brien et al. | n.r. | n.r. | yes | n.r. | n.r. | n.r. | deep electrode implantation | n.a. | cortex | n.r. | n.r. | n.r. | n.a. |
| Ogun et al. | n.r. | n.r. | no | n.a. | n.r. | killed | deep recording electrode implantation | n.a. | n.r. | n.r. | n.r. | nonpermanent implant | n.a. |
| Okada et al. | n.r. | n.r. | yes | <2 weeks | n.r. | killed | intracerebral injection cannula | n.a. | parabrachial nucleus | n.r. | 3 | nonpermanent implant | n.a. |
| Park et al. | n.r. | n.r. | yes | <8 weeks | n.r. | killed | other | electrocorticographic recording, cranial window implantation | olfactory bulb | n.r. | 1,2, 4 | n.r. | n.a. |
| Pettibone et al. | n.r. | n.r. | yes | <4 weeks | n.r. | killed | other | intracerebral injection cannula, deep electrode implantation | dorso-ventral trajectory, ventral striatum | n.r. | n.r. | n.r. | n.a. |
| Pflüger et al. | handling for several days | n.r. | yes | >8weeks | n.r. | killed | deep electrode implantation | n.a. | left dorsolateral striatum | n.r. | n.r. | 10 | n.a. |
| Qiao et al. | n.r. | n.r. | yes | <4 weeks | n.r. | killed | other | intracerebral injection cannula, cranial window | n.r. | n.r. | n.r. | n.r. | n.a. |
| Romoli et al. | n.r. | n.r. | yes | <1 week | n.r. | killed | intracerebral injection cannula | n.a. | dorsal CA3-CA1 | n.r. | n.r. | nonpermanent implant | n.a. |
| Russell et al. | n.r. | n.r. | yes | <4 weeks | n.r. | killed | tbi | n.a. | n.r. | n.r. | n.r. | nonpermanent implant | n.a. |
| Sa et al. | n.r. | n.r. | yes | <8 weeks | n.r. | killed | intracerebral guide cannula implantation | n.a. | lateral brain ventricle | 0,6 | n.r. | n.r. | n.a. |
| Sharma et al. | n.r. | n.r. | yes | <8 weeks | n.r. | killed | intracerebroventricular guide cannula implantation | n.a. | lateral brain ventricle | n.r. | n.r. | n.r. | n.a. |
| Shaver et al. | n.r. | 12 to 55 | yes | >8weeks | n.r. | killed | TBI, cci | n.a. | n.a. | 5 | 6 | nonpermanent implant | cci |
| Shiuchi et al. | n.r. | n.r. | yes | <1 day | n.r. | killed | other | intracerebral guide cannula implantation, intracerebroventricular guide cannula implantation | mediobasal hypothalamus, lateral brain ventricle | n.r. | n.r. | n.r. | n.a. |
| Simader et al. | n.r. | n.r. | yes | <1 week | n.r. | killed | intracerebral guide cannula implantation | n.a. | n.r. | 0,36 | n.r. | n.r. | n.a. |
| Slezia et al. | n.r. | n.r. | no | n.a. | n.r. | killed | other | multichannel silicon probe implantation | n.r. | n.r. | 0,4 | n.r. | n.a. |
| Souza et al. | n.r. | n.r. | yes | <8 weeks | n.r. | killed | intracerebral injection cannula | n.a. | retrotrapezoid nucleus | n.r. | n.r. | nonpermanent implant | n.a. |
| Souza et al. | n.r. | n.r. | yes | <8 weeks | n.r. | killed | surface (screw) electrode implantation | n.a. | frontal cortex, parietal cortex | n.r. | n.r. | n.r. | n.a. |
| Stanchi et al. | n.r. | n.r. | yes | n.r. | n.r. | n.r. | other | intracerebral injection cannula, cranial window | n.r. | n.r. | n.r. | n.r. | n.a. |
| Stanojlovic et al. | n.r. | n.r. | yes | <4 weeks | n.r. | killed | intracerebral injection cannula | n.a. | lateral hypothalamus | n.r. | n.r. | nonpermanent implant | n.a. |
| Sun et al. | n.r. | n.r. | yes | n.r. | n.r. | n.r. | other | chronic cranial window | n.r. | n.r. | 5 | n.r. | n.a. |
| Suzuki et al. | n.r. | n.r. | yes | <4 weeks | n.r. | killed | intracerebral injection cannula | n.a. | right basal cistern | n.r. | n.r. | nonpermanent implant | n.a. |
| Szonyi et al. | n.r. | n.r. | yes | <8 weeks | n.r. | killed | other | intracerebral injection cannula, optic fibre implantation | n.r. | n.r. | n.r. | n.r. | n.a. |
| Szonyi et al. | n.r. | n.r. | yes | <8 weeks | n.r. | killed | other | intracerebral injection cannula, optic fibre implantation | n.r. | n.r. | n.r. | n.r. | n.a. |
| Tomov et al. | n.r. | n.r. | yes | <4 weeks | n.r. | killed | intracerebral injection cannula | n.a. | right medial forebrain bundle, right striatum | n.r. | n.r. | nonpermanent implant | n.a. |
| Villa-Cedillo et al. | n.r. | n.r. | yes | <4 weeks | n.r. | killed | intracerebral injection cannula | n.a. | n.r. | n.r. | n.r. | nonpermanent implant | n.a. |
| Villasana et al. | n.r. | n.r. | yes | <4 weeks | n.r. | killed | TBI, cci | n.a. | n.r. | n.r. | n.r. | nonpermanent implant | cci |
| Wang et al. F | n.r. | n.r. | yes | n.r. | n.r. | n.r. | deep electrode implantation | n.a. | hippocampus, cortex | n.r. | n.r. | n.r. | n.a. |
| Wang et al. G | n.r. | n.r. | yes | <2 weeks | n.r. | n.r. | surface (screw) electrode implantation | n.a. | cerebellum | n.r. | n.r. | n.r. | n.a. |
| Wang et al. A | n.r. | n.r. | yes | <4 weeks | n.r. | killed | intracerebral injection cannula | n.a. | lateral brain ventricle | 0,18 | n.r. | nonpermanent implant | n.a. |
| Wang et al. H | n.r. | n.r. | yes | n.r. | n.r. | n.r. | deep electrode implantation | n.a. | hippocampus | n.r. | n.r. | n.r. | n.a. |
| Wang et al. B | n.r. | n.r. | yes | <4 weeks | n.r. | killed | other | intracerebral injection cannula, screw electrode implantation | parietal cortex, parietal cortex | n.r. | n.r. | n.r. | n.a. |
| Wang et al. D | n.r. | n.r. | yes | n.r. | n.r. | n.r. | deep electrode implantation | n.a. | hippocampus | n.r. | n.r. | n.r. | n.a. |
| Wang et al. C | n.r. | n.r. | yes | n.r. | 3 | killed | other | intracerebroventricular injection cannula, screw electrode implantation | temporal cortex | n.r. | n.r. | n.r. | n.a. |
| Wang et al. E | n.r. | n.r. | yes | <8 weeks | n.r. | killed | deep electrode implantation | n.a. | hippocampus, cortex | n.r. | n.r. | n.r. | n.a. |
| Wen et al. A | n.r. | 20 | yes | <2 weeks | n.r. | killed | TBI, fluid percussion | n.a. | parietal area | 2,5 | 3 | n.r. | fluid percussion |
| Wen et al. B | n.r. | n.r. | yes | <8 weeks | n.r. | killed | intracerebroventricular guide cannula implantation | n.a. | lateral brain ventricle | n.r. | n.r. | n.r. | n.a. |
| Xu et al. | n.r. | n.r. | yes | <1 week | n.r. | killed | tbi, cci | n.a. | n.r. | 3 | 4 | nonpermanent implant | n.a. |
| Yang et al. | n.r. | n.r. | yes | n.r. | n.r. | n.r. | other | fluid percussion brain injury OR controlled cortical impact OR penetrating ballistic like brain injury | n.a. | n.r. | 4-8 | nonpermanent implant | tbi |
| Yeung et al. | n.r. | n.r. | yes | <1 week | n.r. | killed | intracerebral injection cannula | n.a. | hippocampus | n.r. | n.r. | nonpermanent implant | n.a. |
| Zhang et al. C | n.r. | n.r. | yes | <8 weeks | n.r. | killed | other | intracerebral injection cannula, intracerebral guide cannula implantation, deep electrode implantation | thalamic reticular nucleus, locus coeruleus, prefrontal cortex | n.r. | n.r. | n.r. | n.a. |
| Zhang et al. B | n.r. | n.r. | yes | <8 weeks | n.r. | killed | tbi, cci | n.a. | cortex | 6 | 10 | nonpermanent implant | cci |
| Zhang et al. A | n.r. | n.r. | yes | n.r. | n.r. | n.r. | surface (screw) electrode implantation | n.a. | n.r. | 1 | n.r. | 16 | n.a. |
| Zhao et al. | n.r. | n.r. | yes | n.r. | n.r. | killed | TBI | n.a. | parietal bone | n.r. | 5 | nonpermanent implant | tbi |

| study ID | general anesthesia, neuroleptics and hypnotics administered? If so, specify scheme (compound names OR n.r.) | drug / compound 1 (name OR n.a. OR n.r.) | administration route inhalation? (yes / no OR n.a.) | if so: volume percent  (Vol % OR n.a.) | if reported, induction:  (Vol% OR n.r. OR n.a.) | if reported, maintenance: (Vol% OR n.r. OR n.a.) | administration route injection?  (yes / no OR n.a.) | if so: injection route (s.c. OR i.p. OR i.v. OR n.a. OR n.r.) | if so:  dosage  (mg/kg OR n.a.) | administered how many times in total?  (number OR n.a. OR n.r.) | if so: administration interval (h post first administration OR n.r. OR n.a.) |
| --- | --- | --- | --- | --- | --- | --- | --- | --- | --- | --- | --- |
| Aldehri et al. | isoflurane | isoflurane | yes | n.r. | n.r. | n.r. | no | n.a. | n.a. | n.a. | n.a. |
| Asan et al. | Isoflurane / ketamine / xylazine | isoflurane | yes | n.a. | 5 | 1-3 | no | n.a. | n.a. | n.a. | n.a. |
| Baud et al. | n.r. | n.r. | n.r. | n.r. | n.r. | n.r. | n.r. | n.r. | n.r. | n.r. | n.r. |
| Bazzu et al. | isoflurane | isoflurane | yes | 1 to 3 | n.r. | n.r. | no | n.a. | n.a. | n.a. | n.a. |
| Bertoglio et al. | isoflurane | isoflurane | yes | n.a. | 5 | 2-2,5 | no | n.a. | n.a. | n.a. | n.a. |
| Bleimeister et al. | isoflurane | isoflurane | yes | n.a. | 4 | 2 | no | n.a. | n.a. | n.a. | n.a. |
| Bukhtiyarova et al. | isoflurane | isoflurane | yes | 1 to 2 | n.r. | n.r. | no | n.a. | n.a. | n.a. | n.a. |
| Burgdorf et al. | isoflurane | isoflurane | yes | n.a. | 5 | 2-3 | no | n.a. | n.a. | n.a. | n.a. |
| Casanova-Carvajal et al. | n.r. | n.r. | n.a. | n.a. | n.a. | n.a. | n.a. | n.a. | n.a. | n.a. | n.a. |
| Chen et al. C | chloral hydrate | chloral hydrate | no | n.a. | n.a. | n.a. | yes | n.r. | 350 | 1 | n.a. |
| Chen et al. E | ketamine / xylazine | ketamine | no | n.a. | n.a. | n.a. | yes | i.p. | 100 | 1 | n.a. |
| Chen et al. A | n.r. | n.r. | n.a. | n.a. | n.a. | n.a. | n.a. | n.a. | n.a. | n.a. | n.a. |
| Chen et al. B | isoflurane | isoflurane | yes | 1,5 | n.r. | n.r. | no | n.a. | n.a. | n.a. | n.a. |
| Chen et al. F | pentobarbital | pentobarbital | no | n.a. | n.a. | n.a. | yes | i.p. | 50 | 1 | n.a. |
| Chitturi et al. | ketamine / xylazine | ketamine | no | n.a. | n.a. | n.a. | yes | i.p. | 80 | 1 | n.a. |
| Christiaen et al. | isoflurane | isoflurane | yes | n.a. | 5 | 2 | no | n.a. | n.a. | n.a. | n.a. |
| Colangeli et al. | isoflurane | isoflurane | yes | n.a. | 5 | 1-2 | no | n.a. | n.a. | n.a. | n.a. |
| da Silva Pacheco et al. | ketamine / xylazine / acepromazine | ketamine | no | n.a. | n.a. | n.a. | yes | s.c. | n.r. | 1 | n.a. |
| Daglas et al. | tribromoethanol | tribromoethanol | no | n.a. | n.a. | n.a. | yes | i.p. | 0,5 | 1 | n.a. |
| Dal-Pont et al. | ketamine / xylazine | ketamine | no | n.a. | n.a. | n.a. | yes | i.m. | 80 | 1 | n.a. |
| Delaney et al. | n.r. | n.r. | n.a. | n.a. | n.a. | n.a. | n.a. | n.a. | n.a. | n.a. | n.a. |
| Dreier et al. | thiopental | thiopental | no | n.a. | n.a. | n.a. | yes | i.p. | 100 | 1 | n.a. |
| Du et al. | isoflurane | isoflurane | yes | 2 | n.r. | n.r. | no | n.a. | n.a. | n.a. | n.a. |
| Duveau et al. | isoflurane | isoflurane | yes | 2 to 2,5 | n.r. | n.r. | no | n.a. | n.a. | n.a. | n.a. |
| Etter et al. | isoflurane | isoflurane | yes | n.r. | 5 | 0,5 to 2 | no | n.a. | n.a. | n.a. | n.a. |
| Ewell et al. | isoflurane | isoflurane | yes | 2 to 2,5 | n.r. | n.r. | no | n.a. | n.a. | n.a. | n.a. |
| Farakhor et al. | ketamine / xylazine | xylazine | no | n.a. | n.a. | n.a. | yes | n.r. | 10 | 1 | n.a. |
| Farooq et al. | isoflurane | isoflurane | yes | 1 to 2 | n.r. | n.r. | no | n.a. | n.a. | n.a. | n.a. |
| Fiath et al. | ketamine / xylazine | ketamine | no | n.a. | n.a. | n.a. | yes | i.m. | 75 | n.r. | n.r. |
| Fortress et al. | ketamine / xylazine | ketamine | no | n.a. | n.a. | n.a. | yes | i.p. | 60 | 1 | n.a. |
| Hu et al. | chloral hydrate | chloral hydrate | no | n.a. | n.a. | n.a. | yes | n.r. | n.r. | 1 | n.a. |
| Ilieva et al. | ketamine / xylazine | ketamine | no | n.a. | n.a. | n.a. | yes | i.p. | 80 | 1 | n.a. |
| Jackson et al. | isoflurane | isoflurane | yes | n.r. | 5 | 2 | no | n.a. | n.a. | n.a. | n.a. |
| Jakkamsetti et al. | ketamine / xylazine / acepromazine | ketamine | no | n.a. | n.a. | n.a. | yes | i.p. | 100 | n.r. | n.r. |
| Jakkamsetti et al. | isoflurane | isoflurane | yes | 1 to 2 | n.r. | n.r. | no | n.a. | n.a. | n.a. | n.a. |
| Jermakowicz et al. | isoflurane | isoflurane | yes | 1,2 | n.r. | n.r. | no | n.a. | n.a. | n.a. | n.a. |
| Kaefer et al. | isoflurane | isoflurane | yes | 0,5 to 3 | n.r. | n.r. | no | n.a. | n.a. | n.a. | n.a. |
| Katagiri et al. | pentobarbital | pentobarbital | no | n.a. | n.a. | n.a. | yes | i.p. | 50 | 1 | n.a. |
| Kenny et al. | isoflurane | isoflurane | yes | n.a. | 3 | 1,5 | no | n.a. | n.a. | n.a. | n.a. |
| Kim et al. B | n.r. | n.r. | n.a. | n.a. | n.a. | n.a. | n.a. | n.a. | n.a. | n.a. | n.a. |
| Kunori et al. | isoflurane | isoflurane | yes | n.r. | 3 | 1,25 to 1,5 | no | n.a. | n.a. | n.a. | n.a. |
| Kyyriainen et al. | pentobarbital | pentobarbital | no | n.a. | n.a. | n.a. | yes | i.p. | 60 | 1 | n.a. |
| Lee et al. C | ketamine / xylazine | ketamine | no | n.a. | n.a. | n.a. | yes | i.p. | 7,5 to 8,75 | n.r. | n.r. |
| Levata et al. | ketamine / xylazine | ketamine | no | n.a. | n.a. | n.a. | yes | i.p. | 80 | 1 | n.a. |
| Li et al. A | isoflurane | isoflurane | yes | n.r. | n.r. | n.r. | no | n.a. | n.a. | n.a. | n.a. |
| Li et al. D | urethane | urethane | no | n.a. | n.a. | n.a. | yes | n.r. | 1200 | 1 | n.a. |
| Li et al. B | urethane | urethane | no | n.a. | n.a. | n.a. | yes | i.p. | n.r. | 1 | n.a. |
| Luo et al. | n.r. | n.r. | n.a. | n.a. | n.a. | n.a. | n.a. | n.a. | n.a. | n.a. | n.a. |
| Lv et al. | isoflurane | isoflurane | yes | 2 | n.r. | n.r. | no | n.a. | n.a. | n.a. | n.a. |
| Ma et al. | isoflurane | isoflurane | yes | n.a. | 3 | 2 | no | n.a. | n.a. | n.a. | n.a. |
| Mastrella et al. | ketamine / xylazine | ketamine | no | n.a. | n.a. | n.a. | yes | n.r. | 100 | 1 | n.a. |
| Mazza et al. | isoflurane | isoflurane | yes | n.r. | n.r. | n.r. | no | n.a. | n.a. | n.a. | n.a. |
| Mittal et al. | isoflurane | isoflurane | yes | n.r. | n.r. | n.r. | n.a. | n.a. | n.a. | n.a. | n.a. |
| Mo et al. | n.r. | n.r. | n.a. | n.a. | n.a. | n.a. | n.a. | n.a. | n.a. | n.a. | n.a. |
| Mohammad et al. | isoflurane | isoflurane | yes | n.r. | 5 | 2 | no | n.a. | n.a. | n.a. | n.a. |
| Mohammadipoor-Ghasemabad et al. | ketamine / xylazine | ketamine | no | n.a. | n.a. | n.a. | yes | i.p. | 80 | 1 | n.a. |
| Mohammadpoory et al. | ketamine / xylazine | ketamine | no | n.a. | n.a. | n.a. | yes | i.p. | 60 | 1 | n.a. |
| Moller et al. | chloral hydrate | chloral hydrate | no | n.a. | n.a. | n.a. | yes | i.p. | 360 | 1 | n.a. |
| Murai et al. | isoflurane | isoflurane | yes | n.r. | n.r. | n.r. | no | n.a. | n.a. | n.a. | n.a. |
| Njoku et al. | isoflurane | isoflurane | yes | n.r. | 4 | 2 | no | n.a. | n.a. | n.a. | n.a. |
| O'Brien et al. | isoflurane | isoflurane | yes | n.r. | 2,5 to 3 | 1,7 | no | n.a. | n.a. | n.a. | n.a. |
| Ogun et al. | ketamine / xylazine | ketamine | no | n.a. | n.a. | n.a. | yes | i.m. | 90 | 1 | n.a. |
| Okada et al. | medetomidine / midazolam | medetomidine | no | n.a. | n.a. | n.a. | yes | i.p. | 0,375 | 1 | n.a. |
| Park et al. | isoflurane | isoflurane | yes | n.a. | 3 | 1,5 | no | n.a. | n.a. | n.a. | n.a. |
| Pettibone et al. | n.r. | n.r. | n.a. | n.a. | n.a. | n.a. | n.a. | n.a. | n.a. | n.a. | n.a. |
| Pflüger et al. | isoflurane | isoflurane | yes | n.a. | 4 | 1,5 | no | n.a. | n.a. | n.a. | n.a. |
| Qiao et al. | n.r. | n.r. | n.a. | n.a. | n.a. | n.a. | n.a. | n.a. | n.a. | n.a. | n.a. |
| Romoli et al. | tiletamine / zolazepam / xylazine | tiletamine | no | n.a. | n.a. | n.a. | yes | n.r. | n.r. | 1 | n.a. |
| Russell et al. | isoflurane | isoflurane | yes | n.r. | 4 | 2 | no | n.a. | n.a. | n.a. | n.a. |
| Sa et al. | ketamine / xylazine | ketamine | no | n.a. | n.a. | n.a. | yes | n.r. | n.r. | 1 | n.a. |
| Sharma et al. | ketamine | ketamine | no | n.a. | n.a. | n.a. | yes | i.p. | 75 | 1 | n.a. |
| Shaver et al. | isoflurane | isoflurane | yes | n.a. | 5 | 2-4 | no | n.a. | n.a. | n.a. | n.a. |
| Shiuchi et al. | ketamine / xylazine | ketamine | no | n.a. | n.a. | n.a. | yes | i.p. | 100 | 1 | n.a. |
| Simader et al. | ketamine / xylazine / isoflurane | ketamine | no | n.a. | n.a. | n.a. | yes | i.p. | 100 | 1 | n.a. |
| Slezia et al. | ketamine / xylazine | ketamine | no | n.a. | n.a. | n.a. | yes | i.p. | 100 | n.r. | n.r. |
| Souza et al. | ketamine / xylazine / acepromazine | ketamine | no | n.a. | n.a. | n.a. | yes | i.m. | 75 | n.r. | n.r. |
| Souza et al. | ketamine / xylazine / acepromazine | ketamine | no | n.a. | n.a. | n.a. | yes | n.r. | n.r. | n.r. | n.r. |
| Stanchi et al. | ketamine / xylazine | ketamine | no | n.a. | n.a. | n.a. | yes | i.p. | 100 | n.r. | n.r. |
| Stanojlovic et al. | isoflurane | isoflurane | yes | 1 to 4 | n.r. | n.r. | no | n.a. | n.a. | n.a. | n.a. |
| Sun et al. | isoflurane | isoflurane | yes | n.r. | 4 | 1,5 to 2 | no | n.a. | n.a. | n.a. | n.a. |
| Suzuki et al. | isoflurane | isoflurane | yes | n.r. | 3 | 1 | no | n.a. | n.a. | n.a. | n.a. |
| Szonyi et al. | ketamine / xylazine / isoflurane | ketamine | no | n.a. | n.a. | n.a. | yes | i.p. | n.r. | n.r. | n.r. |
| Szonyi et al. | ketamine / xylazine / isoflurane | ketamine | no | n.a. | n.a. | n.a. | yes | i.p. | n.r. | n.r. | n.r. |
| Tomov et al. | isoflurane | isoflurane | yes | n.r. | 4 | 2 | no | n.a. | n.a. | n.a. | n.a. |
| Villa-Cedillo et al. | tribromoethanol | tribromoethanol | no | n.a. | n.a. | n.a. | yes | n.r. | 125 to 150 | 1 | n.a. |
| Villasana et al. | isoflurane | isoflurane | yes | 2 | n.r. | n.r. | no | n.a. | n.a. | n.a. | n.a. |
| Wang et al. F | isoflurane | isoflurane | yes | 3 | n.r. | n.r. | no | n.a. | n.a. | n.a. | n.a. |
| Wang et al. G | pentobarbital | pentobarbital | no | n.a. | n.a. | n.a. | yes | i.p. | 100 | 1 | n.a. |
| Wang et al. A | isoflurane | isoflurane | yes | 1,5 | n.r. | n.r. | no | n.a. | n.a. | n.a. | n.a. |
| Wang et al. H | n.r. | n.r. | n.a. | n.a. | n.a. | n.a. | n.a. | n.a. | n.a. | n.a. | n.a. |
| Wang et al. B | n.r. | n.r. | n.a. | n.a. | n.a. | n.a. | n.a. | n.a. | n.a. | n.a. | n.a. |
| Wang et al. D | chloral hydrate | chloral hydrate | no | n.a. | n.a. | n.a. | yes | i.p. | n.r. | 1 | n.a. |
| Wang et al. C | isoflurane | isoflurane | yes | n.r. | 5 | 2 | no | n.a. | n.a. | n.a. | n.a. |
| Wang et al. E | chloral hydrate | chloral hydrate | no | n.a. | n.a. | n.a. | yes | i.p. | 1 | 1 | n.a. |
| Wen et al. A | ketamine / xylazine | ketamine | no | n.a. | n.a. | n.a. | yes | i.p. | 80 to 100 | 1 | n.a. |
| Wen et al. B | ketamine / xylazine / acepromazine | ketamine | no | n.a. | n.a. | n.a. | yes | n.r. | 90 | 1 | n.a. |
| Xu et al. | pentobarbital | pentobarbital | no | n.a. | n.a. | n.a. | yes | i.p. | 65 | 1 | n.a. |
| Yang et al. | isoflurane | isoflurane | yes | n.a. | 4 | 2-5 | no | n.a. | n.a. | n.a. | n.a. |
| Yeung et al. | ketamine / medetomidine | ketamine | no | n.a. | n.a. | n.a. | yes | s.c. | 75 | 1 | n.a. |
| Zhang et al. C | pentobarbital | pentobarbital | no | n.a. | n.a. | n.a. | yes | n.r. | 50 | 1 | n.a. |
| Zhang et al. B | ketamine / xylazine | ketamine | no | n.a. | n.a. | n.a. | yes | i.p. | 100 | 1 | n.a. |
| Zhang et al. A | isoflurane | isoflurane | yes | n.r. | 5 | 2 | no | n.a. | n.a. | n.a. | n.a. |
| Zhao et al. | pentobarbital | pentobarbital | no | n.a. | n.a. | n.a. | yes | n.r. | 35 | 1 | n.a. |

| study ID | drug / compound 2 (name OR n.a.) | administration route injection?  (yes / no OR n.a.) | if so:  administration route (s.c. OR i.p. OR i.v. OR n.a. OR n.r.) | if so:  dosage  (mg/kg OR n.a.) | administered how many times in total?  (number OR n.a. OR n.r.) | drug / compound 3 (name OR n.a.) | administration route inhalation? (yes / no OR n.a.) | if so: volume percent  (Vol % OR n.a.) | administration route injection?  (yes / no OR n.a.) | if so:  administration route (s.c. OR i.p. OR i.v. OR n.a. OR n.r.) | if so:  dosage  (mg/kg OR n.a.) | administered how many times in total?  (number OR n.a. OR n.r.) |
| --- | --- | --- | --- | --- | --- | --- | --- | --- | --- | --- | --- | --- |
| Aldehri et al. | n.a. | n.a. | n.a. | n.a. | n.a. | n.a. | n.a. | n.a. | n.a. | n.a. | n.a. | n.a. |
| Asan et al. | ketamine | yes | i.p. | 80 | n.r. | xylazine | no | n.a. | yes | i.p. | 12 | n.r. |
| Baud et al. | n.r. | n.r. | n.r. | n.r. | n.r. | n.r. | n.a. | n.a. | n.r. | n.r. | n.r. | n.r. |
| Bazzu et al. | n.a. | n.a. | n.a. | n.a. | n.a. | n.a. | n.a. | n.a. | n.a. | n.a. | n.a. | n.a. |
| Bertoglio et al. | n.a. | n.a. | n.a. | n.a. | n.a. | n.a. | n.a. | n.a. | n.a. | n.a. | n.a. | n.a. |
| Bleimeister et al. | n.a. | n.a. | n.a. | n.a. | n.a. | n.a. | n.a. | n.a. | n.a. | n.a. | n.a. | n.a. |
| Bukhtiyarova et al. | n.a. | n.a. | n.a. | n.a. | n.a. | n.a. | n.a. | n.a. | n.a. | n.a. | n.a. | n.a. |
| Burgdorf et al. | n.a. | n.a. | n.a. | n.a. | n.a. | n.a. | n.a. | n.a. | n.a. | n.a. | n.a. | n.a. |
| Casanova-Carvajal et al. | n.a. | n.a. | n.a. | n.a. | n.a. | n.a. | n.a. | n.a. | n.a. | n.a. | n.a. | n.a. |
| Chen et al. C | n.a. | n.a. | n.a. | n.a. | n.a. | n.a. | n.a. | n.a. | n.a. | n.a. | n.a. | n.a. |
| Chen et al. E | xylazine | yes | i.p. | 10 | 1 | n.a. | n.a. | n.a. | n.a. | n.a. | n.a. | n.a. |
| Chen et al. A | n.a. | n.a. | n.a. | n.a. | n.a. | n.a. | n.a. | n.a. | n.a. | n.a. | n.a. | n.a. |
| Chen et al. B | n.a. | n.a. | n.a. | n.a. | n.a. | n.a. | n.a. | n.a. | n.a. | n.a. | n.a. | n.a. |
| Chen et al. F | n.a. | n.a. | n.a. | n.a. | n.a. | n.a. | n.a. | n.a. | n.a. | n.a. | n.a. | n.a. |
| Chitturi et al. | xylazine | yes | i.p. | 10 | 1 | n.a. | n.a. | n.a. | n.a. | n.a. | n.a. | n.a. |
| Christiaen et al. | n.a. | n.a. | n.a. | n.a. | n.a. | n.a. | n.a. | n.a. | n.a. | n.a. | n.a. | n.a. |
| Colangeli et al. | n.a. | n.a. | n.a. | n.a. | n.a. | n.a. | n.a. | n.a. | n.a. | n.a. | n.a. | n.a. |
| da Silva Pacheco et al. | xylazine | yes | s.c. | n.r. | 1 | acepromazine | no | n.a. | yes | s.c. | n.r. | 1 |
| Daglas et al. | n.a. | n.a. | n.a. | n.a. | n.a. | n.a. | n.a. | n.a. | n.a. | n.a. | n.a. | n.a. |
| Dal-Pont et al. | xylazine | yes | i.m. | 10 | 1 | n.a. | n.a. | n.a. | n.a. | n.a. | n.a. | n.a. |
| Delaney et al. | n.a. | n.a. | n.a. | n.a. | n.a. | n.a. | n.a. | n.a. | n.a. | n.a. | n.a. | n.a. |
| Dreier et al. | n.a. | n.a. | n.a. | n.a. | n.a. | n.a. | n.a. | n.a. | n.a. | n.a. | n.a. | n.a. |
| Du et al. | n.a. | n.a. | n.a. | n.a. | n.a. | n.a. | n.a. | n.a. | n.a. | n.a. | n.a. | n.a. |
| Duveau et al. | n.a. | n.a. | n.a. | n.a. | n.a. | n.a. | n.a. | n.a. | n.a. | n.a. | n.a. | n.a. |
| Etter et al. | n.a. | n.a. | n.a. | n.a. | n.a. | n.a. | n.a. | n.a. | n.a. | n.a. | n.a. | n.a. |
| Ewell et al. | n.a. | n.a. | n.a. | n.a. | n.a. | n.a. | n.a. | n.a. | n.a. | n.a. | n.a. | n.a. |
| Farakhor et al. | ketamine | yes | n.r. | 100 | 1 | n.a. | n.a. | n.a. | n.a. | n.a. | n.a. | n.a. |
| Farooq et al. | n.a. | n.a. | n.a. | n.a. | n.a. | n.a. | n.a. | n.a. | n.a. | n.a. | n.a. | n.a. |
| Fiath et al. | xylazine | yes | i.m. | 10 | n.r. | n.a. | n.a. | n.a. | n.a. | n.a. | n.a. | n.a. |
| Fortress et al. | xylazine | yes | i.p. | 7 | 1 | n.a. | n.a. | n.a. | n.a. | n.a. | n.a. | n.a. |
| Hu et al. | n.a. | n.a. | n.a. | n.a. | n.a. | n.a. | n.a. | n.a. | n.a. | n.a. | n.a. | n.a. |
| Ilieva et al. | xylazine | yes | i.p. | 20 | 1 | n.a. | n.a. | n.a. | n.a. | n.a. | n.a. | n.a. |
| Jackson et al. | n.a. | n.a. | n.a. | n.a. | n.a. | n.a. | n.a. | n.a. | n.a. | n.a. | n.a. | n.a. |
| Jakkamsetti et al. | xylazine | yes | i.p. | 10 | n.r. | acepromazine | no | n.a. | yes | i.p. | 2 | n.r. |
| Jakkamsetti et al. | n.a. | n.a. | n.a. | n.a. | n.a. | n.a. | n.a. | n.a. | n.a. | n.a. | n.a. | n.a. |
| Jermakowicz et al. | n.a. | n.a. | n.a. | n.a. | n.a. | n.a. | n.a. | n.a. | n.a. | n.a. | n.a. | n.a. |
| Kaefer et al. | n.a. | n.a. | n.a. | n.a. | n.a. | n.a. | n.a. | n.a. | n.a. | n.a. | n.a. | n.a. |
| Katagiri et al. | n.a. | n.a. | n.a. | n.a. | n.a. | n.a. | n.a. | n.a. | n.a. | n.a. | n.a. | n.a. |
| Kenny et al. | n.a. | n.a. | n.a. | n.a. | n.a. | n.a. | n.a. | n.a. | n.a. | n.a. | n.a. | n.a. |
| Kim et al. B | n.a. | n.a. | n.a. | n.a. | n.a. | n.a. | n.a. | n.a. | n.a. | n.a. | n.a. | n.a. |
| Kunori et al. | n.a. | n.a. | n.a. | n.a. | n.a. | n.a. | n.a. | n.a. | n.a. | n.a. | n.a. | n.a. |
| Kyyriainen et al. | n.a. | n.a. | n.a. | n.a. | n.a. | n.a. | n.a. | n.a. | n.a. | n.a. | n.a. | n.a. |
| Lee et al. C | xylazine | yes | i.p. | 3 to 3,5 | n.r. | n.a. | n.a. | n.a. | n.a. | n.a. | n.a. | n.a. |
| Levata et al. | xylazine | yes | i.p. | 12 | 1 | n.a. | n.a. | n.a. | n.a. | n.a. | n.a. | n.a. |
| Li et al. A | n.a. | n.a. | n.a. | n.a. | n.a. | n.a. | n.a. | n.a. | n.a. | n.a. | n.a. | n.a. |
| Li et al. D | n.a. | n.a. | n.a. | n.a. | n.a. | n.a. | n.a. | n.a. | n.a. | n.a. | n.a. | n.a. |
| Li et al. B | n.a. | n.a. | n.a. | n.a. | n.a. | n.a. | n.a. | n.a. | n.a. | n.a. | n.a. | n.a. |
| Luo et al. | n.a. | n.a. | n.a. | n.a. | n.a. | n.a. | n.a. | n.a. | n.a. | n.a. | n.a. | n.a. |
| Lv et al. | n.a. | n.a. | n.a. | n.a. | n.a. | n.a. | n.a. | n.a. | n.a. | n.a. | n.a. | n.a. |
| Ma et al. | n.a. | n.a. | n.a. | n.a. | n.a. | n.a. | n.a. | n.a. | n.a. | n.a. | n.a. | n.a. |
| Mastrella et al. | xylazine | yes | n.r. | 10 | 1 | n.a. | n.a. | n.a. | n.a. | n.a. | n.a. | n.a. |
| Mazza et al. | n.a. | n.a. | n.a. | n.a. | n.a. | n.a. | n.a. | n.a. | n.a. | n.a. | n.a. | n.a. |
| Mittal et al. | n.a. | n.a. | n.a. | n.a. | n.a. | n.a. | n.a. | n.a. | n.a. | n.a. | n.a. | n.a. |
| Mo et al. | n.a. | n.a. | n.a. | n.a. | n.a. | n.a. | n.a. | n.a. | n.a. | n.a. | n.a. | n.a. |
| Mohammad et al. | n.a. | n.a. | n.a. | n.a. | n.a. | n.a. | n.a. | n.a. | n.a. | n.a. | n.a. | n.a. |
| Mohammadipoor-Ghasemabad et al. | xylazine | yes | i.p. | 10 | 1 | n.a. | n.a. | n.a. | n.a. | n.a. | n.a. | n.a. |
| Mohammadpoory et al. | xylazine | yes | i.p. | 10 | 1 | n.a. | n.a. | n.a. | n.a. | n.a. | n.a. | n.a. |
| Moller et al. | n.a. | n.a. | n.a. | n.a. | n.a. | n.a. | n.a. | n.a. | n.a. | n.a. | n.a. | n.a. |
| Murai et al. | n.a. | n.a. | n.a. | n.a. | n.a. | n.a. | n.a. | n.a. | n.a. | n.a. | n.a. | n.a. |
| Njoku et al. | n.a. | n.a. | n.a. | n.a. | n.a. | n.a. | n.a. | n.a. | n.a. | n.a. | n.a. | n.a. |
| O'Brien et al. | n.a. | n.a. | n.a. | n.a. | n.a. | n.a. | n.a. | n.a. | n.a. | n.a. | n.a. | n.a. |
| Ogun et al. | xylazine | yes | i.m. | 10 | 1 | n.a. | n.a. | n.a. | n.a. | n.a. | n.a. | n.a. |
| Okada et al. | midazolam | yes | i.p. | 2 | 1 | n.a. | n.a. | n.a. | n.a. | n.a. | n.a. | n.a. |
| Park et al. | n.a. | n.a. | n.a. | n.a. | n.a. | n.a. | n.a. | n.a. | n.a. | n.a. | n.a. | n.a. |
| Pettibone et al. | n.a. | n.a. | n.a. | n.a. | n.a. | n.a. | n.a. | n.a. | n.a. | n.a. | n.a. | n.a. |
| Pflüger et al. | n.a. | n.a. | n.a. | n.a. | n.a. | n.a. | n.a. | n.a. | n.a. | n.a. | n.a. | n.a. |
| Qiao et al. | n.a. | n.a. | n.a. | n.a. | n.a. | n.a. | n.a. | n.a. | n.a. | n.a. | n.a. | n.a. |
| Romoli et al. | zolazepam | yes | n.r. | n.r. | 1 | xylazine | no | n.a. | y | n.r. | n.r. | 1 |
| Russell et al. | n.a. | n.a. | n.a. | n.a. | n.a. | n.a. | n.a. | n.a. | n.a. | n.a. | n.a. | n.a. |
| Sa et al. | xylazine | yes | n.r. | n.r. | 1 | n.a. | n.a. | n.a. | n.a. | n.a. | n.a. | n.a. |
| Sharma et al. | n.a. | n.a. | n.a. | n.a. | n.a. | n.a. | n.a. | n.a. | n.a. | n.a. | n.a. | n.a. |
| Shaver et al. | n.a. | n.a. | n.a. | n.a. | n.a. | n.a. | n.a. | n.a. | n.a. | n.a. | n.a. | n.a. |
| Shiuchi et al. | xylazine | yes | i.p. | 10 | 1 | n.a. | n.a. | n.a. | n.a. | n.a. | n.a. | n.a. |
| Simader et al. | xylazine | yes | i.p. | 10 | 1 | isoflurane | yes | 1,5 | no | n.a. | n.a. | n.a. |
| Slezia et al. | xylazine | yes | i.p. | 10 | n.r. | n.a. | n.a. | n.a. | n.a. | n.a. | n.a. | n.a. |
| Souza et al. | xylazine | yes | i.m. | 5 | n.r. | acepromazine | no | n.a. | yes | i.m. | 1 | n.r. |
| Souza et al. | xylazine | yes | n.r. | n.r. | n.r. | acepromazine | no | n.a. | yes | n.r. | n.r. | n.r. |
| Stanchi et al. | xylazine | yes | i.p. | 10 | n.r. | n.a. | n.a. | n.a. | n.a. | n.a. | n.a. | n.a. |
| Stanojlovic et al. | n.a. | n.a. | n.a. | n.a. | n.a. | n.a. | n.a. | n.a. | n.a. | n.a. | n.a. | n.a. |
| Sun et al. | n.a. | n.a. | n.a. | n.a. | n.a. | n.a. | n.a. | n.a. | n.a. | n.a. | n.a. | n.a. |
| Suzuki et al. | n.a. | n.a. | n.a. | n.a. | n.a. | n.a. | n.a. | n.a. | n.a. | n.a. | n.a. | n.a. |
| Szonyi et al. | xylazine | yes | i.p. | n.r. | n.r. | isoflurane | yes | 2 | no | n.a. | n.a. | n.a. |
| Szonyi et al. | xylazine | yes | i.p. | n.r. | n.r. | isoflurane | yes | 2 | no | n.a. | n.a. | n.a. |
| Tomov et al. | n.a. | n.a. | n.a. | n.a. | n.a. | n.a. | n.a. | n.a. | n.a. | n.a. | n.a. | n.a. |
| Villa-Cedillo et al. | n.a. | n.a. | n.a. | n.a. | n.a. | n.a. | n.a. | n.a. | n.a. | n.a. | n.a. | n.a. |
| Villasana et al. | n.a. | n.a. | n.a. | n.a. | n.a. | n.a. | n.a. | n.a. | n.a. | n.a. | n.a. | n.a. |
| Wang et al. F | n.a. | n.a. | n.a. | n.a. | n.a. | n.a. | n.a. | n.a. | n.a. | n.a. | n.a. | n.a. |
| Wang et al. G | n.a. | n.a. | n.a. | n.a. | n.a. | n.a. | n.a. | n.a. | n.a. | n.a. | n.a. | n.a. |
| Wang et al. A | n.a. | n.a. | n.a. | n.a. | n.a. | n.a. | n.a. | n.a. | n.a. | n.a. | n.a. | n.a. |
| Wang et al. H | n.a. | n.a. | n.a. | n.a. | n.a. | n.a. | n.a. | n.a. | n.a. | n.a. | n.a. | n.a. |
| Wang et al. B | n.a. | n.a. | n.a. | n.a. | n.a. | n.a. | n.a. | n.a. | n.a. | n.a. | n.a. | n.a. |
| Wang et al. D | n.a. | n.a. | n.a. | n.a. | n.a. | n.a. | n.a. | n.a. | n.a. | n.a. | n.a. | n.a. |
| Wang et al. C | n.a. | n.a. | n.a. | n.a. | n.a. | n.a. | n.a. | n.a. | n.a. | n.a. | n.a. | n.a. |
| Wang et al. E | n.a. | n.a. | n.a. | n.a. | n.a. | n.a. | n.a. | n.a. | n.a. | n.a. | n.a. | n.a. |
| Wen et al. A | xylazine | yes | i.p. | 10 | 1 | n.a. | n.a. | n.a. | n.a. | n.a. | n.a. | n.a. |
| Wen et al. B | xylazine | yes | n.r. | 2,7 | 1 | acepromazine | no | n.a. | yes | n.r. | 0,64 | 1 |
| Xu et al. | n.a. | n.a. | n.a. | n.a. | n.a. | n.a. | n.a. | n.a. | n.a. | n.a. | n.a. | n.a. |
| Yang et al. | n.a. | n.a. | n.a. | n.a. | n.a. | n.a. | n.a. | n.a. | n.a. | n.a. | n.a. | n.a. |
| Yeung et al. | medetomidine | yes | s.c. | 1 | 1 | n.a. | n.a. | n.a. | n.a. | n.a. | n.a. | n.a. |
| Zhang et al. C | n.a. | n.a. | n.a. | n.a. | n.a. | n.a. | n.a. | n.a. | n.a. | n.a. | n.a. | n.a. |
| Zhang et al. B | xylazine | yes | i.p. | 10 | 1 | n.a. | n.a. | n.a. | n.a. | n.a. | n.a. | n.a. |
| Zhang et al. A | n.a. | n.a. | n.a. | n.a. | n.a. | n.a. | n.a. | n.a. | n.a. | n.a. | n.a. | n.a. |
| Zhao et al. | n.a. | n.a. | n.a. | n.a. | n.a. | n.a. | n.a. | n.a. | n.a. | n.a. | n.a. | n.a. |

| study ID | local anesthesia administered?  (yes / no OR n.r.) | drug / compound 1   (name OR n.a.) | administration route   (s.c. OR applied topically OR n.a.) | dosage   (mg/kg) OR n.r. OR n.a. | injection volume   (ml/animal OR n.r. OR n.a.) | timepoint of first administration   (pre-, intra-, postsurgically OR n.a.) | if reported:  immediately before surgery OR immediately after surgery OR n.r. OR n.a. | administered how many times in total?   (number OR n.a.) | if so: administration interval   (h post first administration Or n.r. OR n.a.) | if reported: drug concentration   (mg/ml OR n.r. OR n.a.) | if reported:  pharmaceutical formulation  (e.g. solution OR n.r. OR n.a.) |
| --- | --- | --- | --- | --- | --- | --- | --- | --- | --- | --- | --- |
| Aldehri et al. | yes | lidocaine | s.c. | n.r. | n.r. | presurgically | n.a. | 1 | n.a. | 0,005 to 0,01 | n.r. |
| Asan et al. | no | n.a. | n.a. | n.a. | n.a. | n.a. | n.a. | n.a. | n.a. | n.a. | n.a. |
| Baud et al. | no | n.a. | n.a. | n.a. | n.a. | n.a. | n.a. | n.a. | n.a. | n.a. | n.a. |
| Bazzu et al. | yes | lidocaine | n.r. | n.r. | n.r. | presurgically | immediately before surgery | n.r. | n.r. | n.r. | solution |
| Bertoglio et al. | yes | lidocaine | applied topically | n.r. | n.r. | postsurgically | immediately after surgery | 1 | n.a. | n.a. | gel |
| Bleimeister et al. | no | n.a. | n.a. | n.a. | n.a. | n.a. | n.a. | n.a. | n.a. | n.a. | n.a. |
| Bukhtiyarova et al. | yes | bupivacaine | s.c. | n.r. | n.r. | presurgically | immediately before surgery | 1 | n.a. | 0,0025 | n.r. |
| Burgdorf et al. | no | n.a. | n.a. | n.a. | n.a. | n.a. | n.a. | n.a. | n.a. | n.a. | n.a. |
| Casanova-Carvajal et al. | no | n.a. | n.a. | n.a. | n.a. | n.a. | n.a. | n.a. | n.a. | n.a. | n.a. |
| Chen et al. C | no | n.a. | n.a. | n.a. | n.a. | n.a. | n.a. | n.a. | n.a. | n.a. | n.a. |
| Chen et al. E | no | n.a. | n.a. | n.a. | n.a. | n.a. | n.a. | n.a. | n.a. | n.a. | n.a. |
| Chen et al. A | no | n.a. | n.a. | n.a. | n.a. | n.a. | n.a. | n.a. | n.a. | n.a. | n.a. |
| Chen et al. B | no | n.a. | n.a. | n.a. | n.a. | n.a. | n.a. | n.a. | n.a. | n.a. | n.a. |
| Chen et al. F | no | n.a. | n.a. | n.a. | n.a. | n.a. | n.a. | n.a. | n.a. | n.a. | n.a. |
| Chitturi et al. | no | n.a. | n.a. | n.a. | n.a. | n.a. | n.a. | n.a. | n.a. | n.a. | n.a. |
| Christiaen et al. | yes | lidocaine | applied topically | n.r. | n.r. | postsurgically | immediately after surgery | 1 | n.a. | n.r. | gel |
| Colangeli et al. | yes | lidocaine | s.c. | n.r. | n.r. | n.r. | n.r. | n.r. | n.r. | n.r. | n.r. |
| da Silva Pacheco et al. | no | n.a. | n.a. | n.a. | n.a. | n.a. | n.a. | n.a. | n.a. | n.a. | n.a. |
| Daglas et al. | no | n.a. | n.a. | n.a. | n.a. | n.a. | n.a. | n.a. | n.a. | n.a. | n.a. |
| Dal-Pont et al. | no | n.a. | n.a. | n.a. | n.a. | n.a. | n.a. | n.a. | n.a. | n.a. | n.a. |
| Delaney et al. | no | n.a. | n.a. | n.a. | n.a. | n.a. | n.a. | n.a. | n.a. | n.a. | n.a. |
| Dreier et al. | no | n.a. | n.a. | n.a. | n.a. | n.a. | n.a. | n.a. | n.a. | n.a. | n.a. |
| Du et al. | no | n.a. | n.a. | n.a. | n.a. | n.a. | n.a. | n.a. | n.a. | n.a. | n.a. |
| Duveau et al. | no | n.a. | n.a. | n.a. | n.a. | n.a. | n.a. | n.a. | n.a. | n.a. | n.a. |
| Etter et al. | no | n.a. | n.a. | n.a. | n.a. | n.a. | n.a. | n.a. | n.a. | n.a. | n.a. |
| Ewell et al. | no | n.a. | n.a. | n.a. | n.a. | n.a. | n.a. | n.a. | n.a. | n.a. | n.a. |
| Farakhor et al. | yes | lidocaine | n.r. | n.r. | n.r. | n.r. | n.r. | n.r. | n.r. | n.r. | n.r. |
| Farooq et al. | no | n.a. | n.a. | n.a. | n.a. | n.a. | n.a. | n.a. | n.a. | n.a. | n.a. |
| Fiath et al. | no | n.a. | n.a. | n.a. | n.a. | n.a. | n.a. | n.a. | n.a. | n.a. | n.a. |
| Fortress et al. | no | n.a. | n.a. | n.a. | n.a. | n.a. | n.a. | n.a. | n.a. | n.a. | n.a. |
| Hu et al. | no | n.a. | n.a. | n.a. | n.a. | n.a. | n.a. | n.a. | n.a. | n.a. | n.a. |
| Ilieva et al. | no | n.a. | n.a. | n.a. | n.a. | n.a. | n.a. | n.a. | n.a. | n.a. | n.a. |
| Jackson et al. | no | n.a. | n.a. | n.a. | n.a. | n.a. | n.a. | n.a. | n.a. | n.a. | n.a. |
| Jakkamsetti et al. | no | n.a. | n.a. | n.a. | n.a. | n.a. | n.a. | n.a. | n.a. | n.a. | n.a. |
| Jakkamsetti et al. | no | n.a. | n.a. | n.a. | n.a. | n.a. | n.a. | n.a. | n.a. | n.a. | n.a. |
| Jermakowicz et al. | no | n.a. | n.a. | n.a. | n.a. | n.a. | n.a. | n.a. | n.a. | n.a. | n.a. |
| Kaefer et al. | no | n.a. | n.a. | n.a. | n.a. | n.a. | n.a. | n.a. | n.a. | n.a. | n.a. |
| Katagiri et al. | no | n.a. | n.a. | n.a. | n.a. | n.a. | n.a. | n.a. | n.a. | n.a. | n.a. |
| Kenny et al. | no | n.a. | n.a. | n.a. | n.a. | n.a. | n.a. | n.a. | n.a. | n.a. | n.a. |
| Kim et al. B | no | n.a. | n.a. | n.a. | n.a. | n.a. | n.a. | n.a. | n.a. | n.a. | n.a. |
| Kunori et al. | no | n.a. | n.a. | n.a. | n.a. | n.a. | n.a. | n.a. | n.a. | n.a. | n.a. |
| Kyyriainen et al. | no | n.a. | n.a. | n.a. | n.a. | n.a. | n.a. | n.a. | n.a. | n.a. | n.a. |
| Lee et al. C | no | n.a. | n.a. | n.a. | n.a. | n.a. | n.a. | n.a. | n.a. | n.a. | n.a. |
| Levata et al. | yes | lidocaine | applied topically | n.r. | n.a. | presurgically | immediately before surgery | 1 | n.a. | n.r. | cream |
| Li et al. A | no | n.a. | n.a. | n.a. | n.a. | n.a. | n.a. | n.a. | n.a. | n.a. | n.a. |
| Li et al. D | no | n.a. | n.a. | n.a. | n.a. | n.a. | n.a. | n.a. | n.a. | n.a. | n.a. |
| Li et al. B | no | n.a. | n.a. | n.a. | n.a. | n.a. | n.a. | n.a. | n.a. | n.a. | n.a. |
| Luo et al. | no | n.a. | n.a. | n.a. | n.a. | n.a. | n.a. | n.a. | n.a. | n.a. | n.a. |
| Lv et al. | no | n.a. | n.a. | n.a. | n.a. | n.a. | n.a. | n.a. | n.a. | n.a. | n.a. |
| Ma et al. | no | n.a. | n.a. | n.a. | n.a. | n.a. | n.a. | n.a. | n.a. | n.a. | n.a. |
| Mastrella et al. | no | n.a. | n.a. | n.a. | n.a. | n.a. | n.a. | n.a. | n.a. | n.a. | n.a. |
| Mazza et al. | no | n.a. | n.a. | n.a. | n.a. | n.a. | n.a. | n.a. | n.a. | n.a. | n.a. |
| Mittal et al. | no | n.a. | n.a. | n.a. | n.a. | n.a. | n.a. | n.a. | n.a. | n.a. | n.a. |
| Mo et al. | no | n.a. | n.a. | n.a. | n.a. | n.a. | n.a. | n.a. | n.a. | n.a. | n.a. |
| Mohammad et al. | no | n.a. | n.a. | n.a. | n.a. | n.a. | n.a. | n.a. | n.a. | n.a. | n.a. |
| Mohammadipoor-Ghasemabad et al. | no | n.a. | n.a. | n.a. | n.a. | n.a. | n.a. | n.a. | n.a. | n.a. | n.a. |
| Mohammadpoory et al. | no | n.a. | n.a. | n.a. | n.a. | n.a. | n.a. | n.a. | n.a. | n.a. | n.a. |
| Moller et al. | yes | bupivacaine | applied topically | n.r. | n.r. | presurgically | immediately before surgery | 1 | n.a. | n.a. | n.r. |
| Murai et al. | no | n.a. | n.a. | n.a. | n.a. | n.a. | n.a. | n.a. | n.a. | n.a. | n.a. |
| Njoku et al. | no | n.a. | n.a. | n.a. | n.a. | n.a. | n.a. | n.a. | n.a. | n.a. | n.a. |
| O'Brien et al. | no | n.a. | n.a. | n.a. | n.a. | n.a. | n.a. | n.a. | n.a. | n.a. | n.a. |
| Ogun et al. | no | n.a. | n.a. | n.a. | n.a. | n.a. | n.a. | n.a. | n.a. | n.a. | n.a. |
| Okada et al. | no | n.a. | n.a. | n.a. | n.a. | n.a. | n.a. | n.a. | n.a. | n.a. | n.a. |
| Park et al. | no | n.a. | n.a. | n.a. | n.a. | n.a. | n.a. | n.a. | n.a. | n.a. | n.a. |
| Pettibone et al. | no | n.a. | n.a. | n.a. | n.a. | n.a. | n.a. | n.a. | n.a. | n.a. | n.a. |
| Pflüger et al. | no | n.a. | n.a. | n.a. | n.a. | n.a. | n.a. | n.a. | n.a. | n.a. | n.a. |
| Qiao et al. | no | n.a. | n.a. | n.a. | n.a. | n.a. | n.a. | n.a. | n.a. | n.a. | n.a. |
| Romoli et al. | no | n.a. | n.a. | n.a. | n.a. | n.a. | n.a. | n.a. | n.a. | n.a. | n.a. |
| Russell et al. | yes | n.r. | n.r. | n.r. | n.r. | n.a. | n.r. | 1 | n.a. | n.r. | n.r. |
| Sa et al. | no | n.a. | n.a. | n.a. | n.a. | n.a. | n.a. | n.a. | n.a. | n.a. | n.a. |
| Sharma et al. | yes | lignocaine | applied topically | n.r. | n.r. | postsurgically | n.a. | 4 | 24 | n.r. | powder |
| Shaver et al. | yes | bupivacaine | s.c. | n.r. | n.r. | presurgically | immediately before surgery | 1 | n.a. | n.a. | solution |
| Shiuchi et al. | no | n.a. | n.a. | n.a. | n.a. | n.a. | n.a. | n.a. | n.a. | n.a. | n.a. |
| Simader et al. | no | n.a. | n.a. | n.a. | n.a. | n.a. | n.a. | n.a. | n.a. | n.a. | n.a. |
| Slezia et al. | yes | ropivacaine | s.c. | n.r. | 0,005 | presurgically | immediately before surgery | 1 | n.a. | n.a. | n.r. |
| Souza et al. | no | n.a. | n.a. | n.a. | n.a. | n.a. | n.a. | n.a. | n.a. | n.a. | n.a. |
| Souza et al. | no | n.a. | n.a. | n.a. | n.a. | n.a. | n.a. | n.a. | n.a. | n.a. | n.a. |
| Stanchi et al. | no | n.a. | n.a. | n.a. | n.a. | n.a. | n.a. | n.a. | n.a. | n.a. | n.a. |
| Stanojlovic et al. | no | n.a. | n.a. | n.a. | n.a. | n.a. | n.a. | n.a. | n.a. | n.a. | n.a. |
| Sun et al. | no | n.a. | n.a. | n.a. | n.a. | n.a. | n.a. | n.a. | n.a. | n.a. | n.a. |
| Suzuki et al. | no | n.a. | n.a. | n.a. | n.a. | n.a. | n.a. | n.a. | n.a. | n.a. | n.a. |
| Szonyi et al. | no | n.a. | n.a. | n.a. | n.a. | n.a. | n.a. | n.a. | n.a. | n.a. | n.a. |
| Szonyi et al. | no | n.a. | n.a. | n.a. | n.a. | n.a. | n.a. | n.a. | n.a. | n.a. | n.a. |
| Tomov et al. | no | n.a. | n.a. | n.a. | n.a. | n.a. | n.a. | n.a. | n.a. | n.a. | n.a. |
| Villa-Cedillo et al. | no | n.a. | n.a. | n.a. | n.a. | n.a. | n.a. | n.a. | n.a. | n.a. | n.a. |
| Villasana et al. | no | n.a. | n.a. | n.a. | n.a. | n.a. | n.a. | n.a. | n.a. | n.a. | n.a. |
| Wang et al. F | yes | lidocaine | applied topically | n.r. | n.r. | postsurgically | n.a. | 4 | 24 | 5% | n.r. |
| Wang et al. G | no | n.a. | n.a. | n.a. | n.a. | n.a. | n.a. | n.a. | n.a. | n.a. | n.a. |
| Wang et al. A | no | n.a. | n.a. | n.a. | n.a. | n.a. | n.a. | n.a. | n.a. | n.a. | n.a. |
| Wang et al. H | no | n.a. | n.a. | n.a. | n.a. | n.a. | n.a. | n.a. | n.a. | n.a. | n.a. |
| Wang et al. B | no | n.a. | n.a. | n.a. | n.a. | n.a. | n.a. | n.a. | n.a. | n.a. | n.a. |
| Wang et al. D | no | n.a. | n.a. | n.a. | n.a. | n.a. | n.a. | n.a. | n.a. | n.a. | n.a. |
| Wang et al. C | no | n.a. | n.a. | n.a. | n.a. | n.a. | n.a. | n.a. | n.a. | n.a. | n.a. |
| Wang et al. E | no | n.a. | n.a. | n.a. | n.a. | n.a. | n.a. | n.a. | n.a. | n.a. | n.a. |
| Wen et al. A | no | n.a. | n.a. | n.a. | n.a. | n.a. | n.a. | n.a. | n.a. | n.a. | n.a. |
| Wen et al. B | no | n.a. | n.a. | n.a. | n.a. | n.a. | n.a. | n.a. | n.a. | n.a. | n.a. |
| Xu et al. | no | n.a. | n.a. | n.a. | n.a. | n.a. | n.a. | n.a. | n.a. | n.a. | n.a. |
| Yang et al. | no | n.a. | n.a. | n.a. | n.a. | n.a. | n.a. | n.a. | n.a. | n.a. | n.a. |
| Yeung et al. | no | n.a. | n.a. | n.a. | n.a. | n.a. | n.a. | n.a. | n.a. | n.a. | n.a. |
| Zhang et al. C | no | n.a. | n.a. | n.a. | n.a. | n.a. | n.a. | n.a. | n.a. | n.a. | n.a. |
| Zhang et al. B | no | n.a. | n.a. | n.a. | n.a. | n.a. | n.a. | n.a. | n.a. | n.a. | n.a. |
| Zhang et al. A | no | n.a. | n.a. | n.a. | n.a. | n.a. | n.a. | n.a. | n.a. | n.a. | n.a. |
| Zhao et al. | no | n.a. | n.a. | n.a. | n.a. | n.a. | n.a. | n.a. | n.a. | n.a. | n.a. |

| study ID | drug / compound 2  (name OR n.a.) | administration route  (s.c. OR applied topically OR n.a.) | dosage  (mg/kg) OR n.r. OR n.a. | injection volume  (ml/animal OR n.r. OR n.a.) | timepoint of first administration  (pre-, intra-, post surgically OR n.a.) | if reported: immediately before surgery OR immediately after surgery OR n.r. OR n.a. | administered how many times in total?  (number OR n.a.) | if so: administration interval  (h post first administration OR n.r. OR n.a.) | if reported: drug concentration  (mg/ml OR n.r. OR n.a.) | analgesia NSAID administered? (yes / no OR n.a.) | drug / compound 1 (name OR n.a.) | administration route  (s.c. OR i.m. OR i.p. OR per os OR n.a.) | dosage  (mg/kg) OR n.r. OR n.a. | timepoint of first administration  (pre-, intra-, postsurgically OR n.a.) | if reported: immediately before surgery OR immediately after surgery OR n.r. OR n.a. |
| --- | --- | --- | --- | --- | --- | --- | --- | --- | --- | --- | --- | --- | --- | --- | --- |
| Aldehri et al. | n.a. | n.a. | n.a. | n.a. | n.a. | n.a. | n.a. | n.a. | n.a. | no | n.a. | n.a. | n.a. | n.a. | n.a. |
| Asan et al. | n.a. | n.a. | n.a. | n.a. | n.a. | n.a. | n.a. | n.a. | n.a. | no | n.a. | n.a. | n.a. | n.a. | n.a. |
| Baud et al. | n.a. | n.a. | n.a. | n.a. | n.a. | n.a. | n.a. | n.a. | n.a. | no | n.a. | n.a. | n.a. | n.a. | n.a. |
| Bazzu et al. | n.a. | n.a. | n.a. | n.a. | n.a. | n.a. | n.a. | n.a. | n.a. | yes | meloxicam | s.c. | 1 | presurgically | immediately before surgery |
| Bertoglio et al. | n.a. | n.a. | n.a. | n.a. | n.a. | n.a. | n.a. | n.a. | n.a. | no | n.a. | n.a. | n.a. | n.a. | n.a. |
| Bleimeister et al. | n.a. | n.a. | n.a. | n.a. | n.a. | n.a. | n.a. | n.a. | n.a. | no | n.a. | n.a. | n.a. | n.a. | n.a. |
| Bukhtiyarova et al. | lidocaine | s.c. | n.r. | n.r. | presurgically | immediately before surgery | 1 | n.a. | 0,005 | no | n.a. | n.a. | n.a. | n.a. | n.a. |
| Burgdorf et al. | n.a. | n.a. | n.a. | n.a. | n.a. | n.a. | n.a. | n.a. | n.a. | no | n.a. | n.a. | n.a. | n.a. | n.a. |
| Casanova-Carvajal et al. | n.a. | n.a. | n.a. | n.a. | n.a. | n.a. | n.a. | n.a. | n.a. | no | n.a. | n.a. | n.a. | n.a. | n.a. |
| Chen et al. C | n.a. | n.a. | n.a. | n.a. | n.a. | n.a. | n.a. | n.a. | n.a. | no | n.a. | n.a. | n.a. | n.a. | n.a. |
| Chen et al. E | n.a. | n.a. | n.a. | n.a. | n.a. | n.a. | n.a. | n.a. | n.a. | no | n.a. | n.a. | n.a. | n.a. | n.a. |
| Chen et al. A | n.a. | n.a. | n.a. | n.a. | n.a. | n.a. | n.a. | n.a. | n.a. | no | n.a. | n.a. | n.a. | n.a. | n.a. |
| Chen et al. B | n.a. | n.a. | n.a. | n.a. | n.a. | n.a. | n.a. | n.a. | n.a. | no | n.a. | n.a. | n.a. | n.a. | n.a. |
| Chen et al. F | n.a. | n.a. | n.a. | n.a. | n.a. | n.a. | n.a. | n.a. | n.a. | no | n.a. | n.a. | n.a. | n.a. | n.a. |
| Chitturi et al. | n.a. | n.a. | n.a. | n.a. | n.a. | n.a. | n.a. | n.a. | n.a. | no | n.a. | n.a. | n.a. | n.a. | n.a. |
| Christiaen et al. | n.a. | n.a. | n.a. | n.a. | n.a. | n.a. | n.a. | n.a. | n.a. | yes | meloxicam | s.c. | 1 | postsurgically | immediately after surgery |
| Colangeli et al. | n.a. | n.a. | n.a. | n.a. | n.a. | n.a. | n.a. | n.a. | n.a. | no | n.a. | n.a. | n.a. | n.a. | n.a. |
| da Silva Pacheco et al. | n.a. | n.a. | n.a. | n.a. | n.a. | n.a. | n.a. | n.a. | n.a. | no | n.a. | n.a. | n.a. | n.a. | n.a. |
| Daglas et al. | n.a. | n.a. | n.a. | n.a. | n.a. | n.a. | n.a. | n.a. | n.a. | no | n.a. | n.a. | n.a. | n.a. | n.a. |
| Dal-Pont et al. | n.a. | n.a. | n.a. | n.a. | n.a. | n.a. | n.a. | n.a. | n.a. | no | n.a. | n.a. | n.a. | n.a. | n.a. |
| Delaney et al. | n.a. | n.a. | n.a. | n.a. | n.a. | n.a. | n.a. | n.a. | n.a. | no | n.a. | n.a. | n.a. | n.a. | n.a. |
| Dreier et al. | n.a. | n.a. | n.a. | n.a. | n.a. | n.a. | n.a. | n.a. | n.a. | no | n.a. | n.a. | n.a. | n.a. | n.a. |
| Du et al. | n.a. | n.a. | n.a. | n.a. | n.a. | n.a. | n.a. | n.a. | n.a. | no | n.a. | n.a. | n.a. | n.a. | n.a. |
| Duveau et al. | n.a. | n.a. | n.a. | n.a. | n.a. | n.a. | n.a. | n.a. | n.a. | no | n.a. | n.a. | n.a. | n.a. | n.a. |
| Etter et al. | n.a. | n.a. | n.a. | n.a. | n.a. | n.a. | n.a. | n.a. | n.a. | no | n.a. | n.a. | n.a. | n.a. | n.a. |
| Ewell et al. | n.a. | n.a. | n.a. | n.a. | n.a. | n.a. | n.a. | n.a. | n.a. | no | n.a. | n.a. | n.a. | n.a. | n.a. |
| Farakhor et al. | n.a. | n.a. | n.a. | n.a. | n.a. | n.a. | n.a. | n.a. | n.a. | yes | meloxicam | n.r. | 0,2 | postsurgically | immediately after surgery |
| Farooq et al. | n.a. | n.a. | n.a. | n.a. | n.a. | n.a. | n.a. | n.a. | n.a. | no | n.a. | n.a. | n.a. | n.a. | n.a. |
| Fiath et al. | n.a. | n.a. | n.a. | n.a. | n.a. | n.a. | n.a. | n.a. | n.a. | no | n.a. | n.a. | n.a. | n.a. | n.a. |
| Fortress et al. | n.a. | n.a. | n.a. | n.a. | n.a. | n.a. | n.a. | n.a. | n.a. | no | n.a. | n.a. | n.a. | n.a. | n.a. |
| Hu et al. | n.a. | n.a. | n.a. | n.a. | n.a. | n.a. | n.a. | n.a. | n.a. | no | n.a. | n.a. | n.a. | n.a. | n.a. |
| Ilieva et al. | n.a. | n.a. | n.a. | n.a. | n.a. | n.a. | n.a. | n.a. | n.a. | no | n.a. | n.a. | n.a. | n.a. | n.a. |
| Jackson et al. | n.a. | n.a. | n.a. | n.a. | n.a. | n.a. | n.a. | n.a. | n.a. | no | n.a. | n.a. | n.a. | n.a. | n.a. |
| Jakkamsetti et al. | n.a. | n.a. | n.a. | n.a. | n.a. | n.a. | n.a. | n.a. | n.a. | no | n.a. | n.a. | n.a. | n.a. | n.a. |
| Jakkamsetti et al. | n.a. | n.a. | n.a. | n.a. | n.a. | n.a. | n.a. | n.a. | n.a. | no | n.a. | n.a. | n.a. | n.a. | n.a. |
| Jermakowicz et al. | n.a. | n.a. | n.a. | n.a. | n.a. | n.a. | n.a. | n.a. | n.a. | no | n.a. | n.a. | n.a. | n.a. | n.a. |
| Kaefer et al. | n.a. | n.a. | n.a. | n.a. | n.a. | n.a. | n.a. | n.a. | n.a. | yes | meloxicam | n.r. | 5 | intrasurgically | n.r. |
| Katagiri et al. | n.a. | n.a. | n.a. | n.a. | n.a. | n.a. | n.a. | n.a. | n.a. | no | n.a. | n.a. | n.a. | n.a. | n.a. |
| Kenny et al. | n.a. | n.a. | n.a. | n.a. | n.a. | n.a. | n.a. | n.a. | n.a. | no | n.a. | n.a. | n.a. | n.a. | n.a. |
| Kim et al. B | n.a. | n.a. | n.a. | n.a. | n.a. | n.a. | n.a. | n.a. | n.a. | no | n.a. | n.a. | n.a. | n.a. | n.a. |
| Kunori et al. | n.a. | n.a. | n.a. | n.a. | n.a. | n.a. | n.a. | n.a. | n.a. | no | n.a. | n.a. | n.a. | n.a. | n.a. |
| Kyyriainen et al. | n.a. | n.a. | n.a. | n.a. | n.a. | n.a. | n.a. | n.a. | n.a. | no | n.a. | n.a. | n.a. | n.a. | n.a. |
| Lee et al. C | n.a. | n.a. | n.a. | n.a. | n.a. | n.a. | n.a. | n.a. | n.a. | no | n.a. | n.a. | n.a. | n.a. | n.a. |
| Levata et al. | prilocaine | applied topically | n.r. | n.a. | presurgically | immediately before surgery | 1 | n.a. | cream | no | n.a. | n.a. | n.a. | n.a. | n.a. |
| Li et al. A | n.a. | n.a. | n.a. | n.a. | n.a. | n.a. | n.a. | n.a. | n.a. | no | n.a. | n.a. | n.a. | n.a. | n.a. |
| Li et al. D | n.a. | n.a. | n.a. | n.a. | n.a. | n.a. | n.a. | n.a. | n.a. | no | n.a. | n.a. | n.a. | n.a. | n.a. |
| Li et al. B | n.a. | n.a. | n.a. | n.a. | n.a. | n.a. | n.a. | n.a. | n.a. | no | n.a. | n.a. | n.a. | n.a. | n.a. |
| Luo et al. | n.a. | n.a. | n.a. | n.a. | n.a. | n.a. | n.a. | n.a. | n.a. | no | n.a. | n.a. | n.a. | n.a. | n.a. |
| Lv et al. | n.a. | n.a. | n.a. | n.a. | n.a. | n.a. | n.a. | n.a. | n.a. | no | n.a. | n.a. | n.a. | n.a. | n.a. |
| Ma et al. | n.a. | n.a. | n.a. | n.a. | n.a. | n.a. | n.a. | n.a. | n.a. | no | n.a. | n.a. | n.a. | n.a. | n.a. |
| Mastrella et al. | n.a. | n.a. | n.a. | n.a. | n.a. | n.a. | n.a. | n.a. | n.a. | yes | carprofen | i.p. | 2 to 4 | presurgically | n.a. |
| Mazza et al. | n.a. | n.a. | n.a. | n.a. | n.a. | n.a. | n.a. | n.a. | n.a. | no | n.a. | n.a. | n.a. | n.a. | n.a. |
| Mittal et al. | n.a. | n.a. | n.a. | n.a. | n.a. | n.a. | n.a. | n.a. | n.a. | no | n.a. | n.a. | n.a. | n.a. | n.a. |
| Mo et al. | n.a. | n.a. | n.a. | n.a. | n.a. | n.a. | n.a. | n.a. | n.a. | no | n.a. | n.a. | n.a. | n.a. | n.a. |
| Mohammad et al. | n.a. | n.a. | n.a. | n.a. | n.a. | n.a. | n.a. | n.a. | n.a. | yes | ketoprofen | s.c. | 5 | presurgically | immediately before surgery |
| Mohammadipoor-Ghasemabad et al. | n.a. | n.a. | n.a. | n.a. | n.a. | n.a. | n.a. | n.a. | n.a. | no | n.a. | n.a. | n.a. | n.a. | n.a. |
| Mohammadpoory et al. | n.a. | n.a. | n.a. | n.a. | n.a. | n.a. | n.a. | n.a. | n.a. | no | n.a. | n.a. | n.a. | n.a. | n.a. |
| Moller et al. | n.a. | n.a. | n.a. | n.a. | n.a. | n.a. | n.a. | n.a. | n.a. | yes | meloxicam | s.c. | 1 | presurgically | n.a. |
| Murai et al. | n.a. | n.a. | n.a. | n.a. | n.a. | n.a. | n.a. | n.a. | n.a. | no | n.a. | n.a. | n.a. | n.a. | n.a. |
| Njoku et al. | n.a. | n.a. | n.a. | n.a. | n.a. | n.a. | n.a. | n.a. | n.a. | no | n.a. | n.a. | n.a. | n.a. | n.a. |
| O'Brien et al. | n.a. | n.a. | n.a. | n.a. | n.a. | n.a. | n.a. | n.a. | n.a. | yes | carprofen | n.r. | n.r. | postsurgically | n.a. |
| Ogun et al. | n.a. | n.a. | n.a. | n.a. | n.a. | n.a. | n.a. | n.a. | n.a. | no | n.a. | n.a. | n.a. | n.a. | n.a. |
| Okada et al. | n.a. | n.a. | n.a. | n.a. | n.a. | n.a. | n.a. | n.a. | n.a. | no | n.a. | n.a. | n.a. | n.a. | n.a. |
| Park et al. | n.a. | n.a. | n.a. | n.a. | n.a. | n.a. | n.a. | n.a. | n.a. | yes | meloxicam | s.c. | 1 | postsurgically | immediately after surgery |
| Pettibone et al. | n.a. | n.a. | n.a. | n.a. | n.a. | n.a. | n.a. | n.a. | n.a. | no | n.a. | n.a. | n.a. | n.a. | n.a. |
| Pflüger et al. | n.a. | n.a. | n.a. | n.a. | n.a. | n.a. | n.a. | n.a. | n.a. | no | n.a. | n.a. | n.a. | n.a. | n.a. |
| Qiao et al. | n.a. | n.a. | n.a. | n.a. | n.a. | n.a. | n.a. | n.a. | n.a. | no | n.a. | n.a. | n.a. | n.a. | n.a. |
| Romoli et al. | n.a. | n.a. | n.a. | n.a. | n.a. | n.a. | n.a. | n.a. | n.a. | no | n.a. | n.a. | n.a. | n.a. | n.a. |
| Russell et al. | n.a. | n.a. | n.a. | n.a. | n.a. | n.a. | n.a. | n.a. | n.a. | no | n.a. | n.a. | n.a. | n.a. | n.a. |
| Sa et al. | n.a. | n.a. | n.a. | n.a. | n.a. | n.a. | n.a. | n.a. | n.a. | yes | ketoprofen | s.c. | n.r. | postsurgically | immediately after surgery |
| Sharma et al. | n.a. | n.a. | n.a. | n.a. | n.a. | n.a. | n.a. | n.a. | n.a. | no | n.a. | n.a. | n.a. | n.a. | n.a. |
| Shaver et al. | n.a. | n.a. | n.a. | n.a. | n.a. | n.a. | n.a. | n.a. | n.a. | yes | ketoprofen | s.c. | 5 | presurgically | immediately before surgery |
| Shiuchi et al. | n.a. | n.a. | n.a. | n.a. | n.a. | n.a. | n.a. | n.a. | n.a. | no | n.a. | n.a. | n.a. | n.a. | n.a. |
| Simader et al. | n.a. | n.a. | n.a. | n.a. | n.a. | n.a. | n.a. | n.a. | n.a. | no | n.a. | n.a. | n.a. | n.a. | n.a. |
| Slezia et al. | n.a. | n.a. | n.a. | n.a. | n.a. | n.a. | n.a. | n.a. | n.a. | no | n.a. | n.a. | n.a. | n.a. | n.a. |
| Souza et al. | n.a. | n.a. | n.a. | n.a. | n.a. | n.a. | n.a. | n.a. | n.a. | yes | ketoprofen | s.c. | 3 to 5 | postsurgically | immediately after surgery |
| Souza et al. | n.a. | n.a. | n.a. | n.a. | n.a. | n.a. | n.a. | n.a. | n.a. | no | n.a. | n.a. | n.a. | n.a. | n.a. |
| Stanchi et al. | n.a. | n.a. | n.a. | n.a. | n.a. | n.a. | n.a. | n.a. | n.a. | no | n.a. | n.a. | n.a. | n.a. | n.a. |
| Stanojlovic et al. | n.a. | n.a. | n.a. | n.a. | n.a. | n.a. | n.a. | n.a. | n.a. | no | n.a. | n.a. | n.a. | n.a. | n.a. |
| Sun et al. | n.a. | n.a. | n.a. | n.a. | n.a. | n.a. | n.a. | n.a. | n.a. | yes | ketoprofen | i.p. | 5 | presurgically | immediately before surgery |
| Suzuki et al. | n.a. | n.a. | n.a. | n.a. | n.a. | n.a. | n.a. | n.a. | n.a. | no | n.a. | n.a. | n.a. | n.a. | n.a. |
| Szonyi et al. | n.a. | n.a. | n.a. | n.a. | n.a. | n.a. | n.a. | n.a. | n.a. | yes | meloxicam | i.p. | 0,03 to 0,05 | postsurgically | immediately after surgery |
| Szonyi et al. | n.a. | n.a. | n.a. | n.a. | n.a. | n.a. | n.a. | n.a. | n.a. | no | n.a. | n.a. | n.a. | n.a. | n.a. |
| Tomov et al. | n.a. | n.a. | n.a. | n.a. | n.a. | n.a. | n.a. | n.a. | n.a. | no | n.a. | n.a. | n.a. | n.a. | n.a. |
| Villa-Cedillo et al. | n.a. | n.a. | n.a. | n.a. | n.a. | n.a. | n.a. | n.a. | n.a. | no | n.a. | n.a. | n.a. | n.a. | n.a. |
| Villasana et al. | n.a. | n.a. | n.a. | n.a. | n.a. | n.a. | n.a. | n.a. | n.a. | no | n.a. | n.a. | n.a. | n.a. | n.a. |
| Wang et al. F | n.a. | n.a. | n.a. | n.a. | n.a. | n.a. | n.a. | n.a. | n.a. | yes | carprofen | s.c. | 20 | postsurgically | n.a. |
| Wang et al. G | n.a. | n.a. | n.a. | n.a. | n.a. | n.a. | n.a. | n.a. | n.a. | no | n.a. | n.a. | n.a. | n.a. | n.a. |
| Wang et al. A | n.a. | n.a. | n.a. | n.a. | n.a. | n.a. | n.a. | n.a. | n.a. | no | n.a. | n.a. | n.a. | n.a. | n.a. |
| Wang et al. H | n.a. | n.a. | n.a. | n.a. | n.a. | n.a. | n.a. | n.a. | n.a. | no | n.a. | n.a. | n.a. | n.a. | n.a. |
| Wang et al. B | n.a. | n.a. | n.a. | n.a. | n.a. | n.a. | n.a. | n.a. | n.a. | no | n.a. | n.a. | n.a. | n.a. | n.a. |
| Wang et al. D | n.a. | n.a. | n.a. | n.a. | n.a. | n.a. | n.a. | n.a. | n.a. | no | n.a. | n.a. | n.a. | n.a. | n.a. |
| Wang et al. C | n.a. | n.a. | n.a. | n.a. | n.a. | n.a. | n.a. | n.a. | n.a. | no | n.a. | n.a. | n.a. | n.a. | n.a. |
| Wang et al. E | n.a. | n.a. | n.a. | n.a. | n.a. | n.a. | n.a. | n.a. | n.a. | no | n.a. | n.a. | n.a. | n.a. | n.a. |
| Wen et al. A | n.a. | n.a. | n.a. | n.a. | n.a. | n.a. | n.a. | n.a. | n.a. | no | n.a. | n.a. | n.a. | n.a. | n.a. |
| Wen et al. B | n.a. | n.a. | n.a. | n.a. | n.a. | n.a. | n.a. | n.a. | n.a. | yes | meloxicam | n.r. | 2 | presurgically | immediately before surgery |
| Xu et al. | n.a. | n.a. | n.a. | n.a. | n.a. | n.a. | n.a. | n.a. | n.a. | no | n.a. | n.a. | n.a. | n.a. | n.a. |
| Yang et al. | n.a. | n.a. | n.a. | n.a. | n.a. | n.a. | n.a. | n.a. | n.a. | no | n.a. | n.a. | n.a. | n.a. | n.a. |
| Yeung et al. | n.a. | n.a. | n.a. | n.a. | n.a. | n.a. | n.a. | n.a. | n.a. | no | n.a. | n.a. | n.a. | n.a. | n.a. |
| Zhang et al. C | n.a. | n.a. | n.a. | n.a. | n.a. | n.a. | n.a. | n.a. | n.a. | no | n.a. | n.a. | n.a. | n.a. | n.a. |
| Zhang et al. B | n.a. | n.a. | n.a. | n.a. | n.a. | n.a. | n.a. | n.a. | n.a. | no | n.a. | n.a. | n.a. | n.a. | n.a. |
| Zhang et al. A | n.a. | n.a. | n.a. | n.a. | n.a. | n.a. | n.a. | n.a. | n.a. | no | n.a. | n.a. | n.a. | n.a. | n.a. |
| Zhao et al. | n.a. | n.a. | n.a. | n.a. | n.a. | n.a. | n.a. | n.a. | n.a. | no | n.a. | n.a. | n.a. | n.a. | n.a. |

| study ID | administered how many times in total?  (number OR n.a. OR n.r.) | if reported: administration interval  (morning after surgery OR 24h post surgery OR n.r. OR n.a.) | if reported: administration interval  (h post first administration OR n.r. OR n.a.) | if reported:  pharmaceutical formulation (e.g. solution OR n.r. OR n.a.) | drug / compound 2 (name OR n.a.) | administration route  (s.c. OR i.m. OR i.p. OR per os OR n.a.) | dosage  (mg/kg) OR n.r. OR n.a. | timepoint of first administration  (pre-, intra-, postsurgically OR n.a.) | if reported: immediately before surgery OR immediately after surgery OR n.r. OR n.a. | administered how many times in total?  (number OR n.a. OR n.r.) | if reported: administration interval  (morning after surgery OR 24h post surgery OR n.r. OR n.a.) | if reported: administration interval  (h post first administration OR n.r. OR n.a.) | if reported:  pharmaceutical formulation (e.g. solution OR n.r. OR n.a.) |
| --- | --- | --- | --- | --- | --- | --- | --- | --- | --- | --- | --- | --- | --- |
| Aldehri et al. | n.a. | n.a. | n.a. | n.a. | n.a. | n.a. | n.a. | n.a. | n.a. | n.a. | n.a. | n.a. | n.a. |
| Asan et al. | n.a. | n.a. | n.a. | n.a. | n.a. | n.a. | n.a. | n.a. | n.a. | n.a. | n.a. | n.a. | n.a. |
| Baud et al. | n.a. | n.a. | n.a. | n.a. | n.a. | n.a. | n.a. | n.a. | n.a. | n.a. | n.a. | n.a. | n.a. |
| Bazzu et al. | 1 | n.a. | n.a. | n.a. | n.a. | n.a. | n.a. | n.a. | n.a. | n.a. | n.a. | n.a. | n.a. |
| Bertoglio et al. | n.a. | n.a. | n.a. | n.a. | n.a. | n.a. | n.a. | n.a. | n.a. | n.a. | n.a. | n.a. | n.a. |
| Bleimeister et al. | n.a. | n.a. | n.a. | n.a. | n.a. | n.a. | n.a. | n.a. | n.a. | n.a. | n.a. | n.a. | n.a. |
| Bukhtiyarova et al. | n.a. | n.a. | n.a. | n.a. | n.a. | n.a. | n.a. | n.a. | n.a. | n.a. | n.a. | n.a. | n.a. |
| Burgdorf et al. | n.a. | n.a. | n.a. | n.a. | n.a. | n.a. | n.a. | n.a. | n.a. | n.a. | n.a. | n.a. | n.a. |
| Casanova-Carvajal et al. | n.a. | n.a. | n.a. | n.a. | n.a. | n.a. | n.a. | n.a. | n.a. | n.a. | n.a. | n.a. | n.a. |
| Chen et al. C | n.a. | n.a. | n.a. | n.a. | n.a. | n.a. | n.a. | n.a. | n.a. | n.a. | n.a. | n.a. | n.a. |
| Chen et al. E | n.a. | n.a. | n.a. | n.a. | n.a. | n.a. | n.a. | n.a. | n.a. | n.a. | n.a. | n.a. | n.a. |
| Chen et al. A | n.a. | n.a. | n.a. | n.a. | n.a. | n.a. | n.a. | n.a. | n.a. | n.a. | n.a. | n.a. | n.a. |
| Chen et al. B | n.a. | n.a. | n.a. | n.a. | n.a. | n.a. | n.a. | n.a. | n.a. | n.a. | n.a. | n.a. | n.a. |
| Chen et al. F | n.a. | n.a. | n.a. | n.a. | n.a. | n.a. | n.a. | n.a. | n.a. | n.a. | n.a. | n.a. | n.a. |
| Chitturi et al. | n.a. | n.a. | n.a. | n.a. | n.a. | n.a. | n.a. | n.a. | n.a. | n.a. | n.a. | n.a. | n.a. |
| Christiaen et al. | 2 | n.a. | 24 | n.r. | n.a. | n.a. | n.a. | n.a. | n.a. | n.a. | n.a. | n.a. | n.a. |
| Colangeli et al. | n.a. | n.a. | n.a. | n.a. | n.a. | n.a. | n.a. | n.a. | n.a. | n.a. | n.a. | n.a. | n.a. |
| da Silva Pacheco et al. | n.a. | n.a. | n.a. | n.a. | n.a. | n.a. | n.a. | n.a. | n.a. | n.a. | n.a. | n.a. | n.a. |
| Daglas et al. | n.a. | n.a. | n.a. | n.a. | n.a. | n.a. | n.a. | n.a. | n.a. | n.a. | n.a. | n.a. | n.a. |
| Dal-Pont et al. | n.a. | n.a. | n.a. | n.a. | n.a. | n.a. | n.a. | n.a. | n.a. | n.a. | n.a. | n.a. | n.a. |
| Delaney et al. | n.a. | n.a. | n.a. | n.a. | n.a. | n.a. | n.a. | n.a. | n.a. | n.a. | n.a. | n.a. | n.a. |
| Dreier et al. | n.a. | n.a. | n.a. | n.a. | n.a. | n.a. | n.a. | n.a. | n.a. | n.a. | n.a. | n.a. | n.a. |
| Du et al. | n.a. | n.a. | n.a. | n.a. | n.a. | n.a. | n.a. | n.a. | n.a. | n.a. | n.a. | n.a. | n.a. |
| Duveau et al. | n.a. | n.a. | n.a. | n.a. | n.a. | n.a. | n.a. | n.a. | n.a. | n.a. | n.a. | n.a. | n.a. |
| Etter et al. | n.a. | n.a. | n.a. | n.a. | n.a. | n.a. | n.a. | n.a. | n.a. | n.a. | n.a. | n.a. | n.a. |
| Ewell et al. | n.a. | n.a. | n.a. | n.a. | n.a. | n.a. | n.a. | n.a. | n.a. | n.a. | n.a. | n.a. | n.a. |
| Farakhor et al. | 4 | 24h post-surgery | 24 | n.r. | n.a. | n.a. | n.a. | n.a. | n.a. | n.a. | n.a. | n.a. | n.a. |
| Farooq et al. | n.a. | n.a. | n.a. | n.a. | n.a. | n.a. | n.a. | n.a. | n.a. | n.a. | n.a. | n.a. | n.a. |
| Fiath et al. | n.a. | n.a. | n.a. | n.a. | n.a. | n.a. | n.a. | n.a. | n.a. | n.a. | n.a. | n.a. | n.a. |
| Fortress et al. | n.a. | n.a. | n.a. | n.a. | n.a. | n.a. | n.a. | n.a. | n.a. | n.a. | n.a. | n.a. | n.a. |
| Hu et al. | n.a. | n.a. | n.a. | n.a. | n.a. | n.a. | n.a. | n.a. | n.a. | n.a. | n.a. | n.a. | n.a. |
| Ilieva et al. | n.a. | n.a. | n.a. | n.a. | n.a. | n.a. | n.a. | n.a. | n.a. | n.a. | n.a. | n.a. | n.a. |
| Jackson et al. | n.a. | n.a. | n.a. | n.a. | n.a. | n.a. | n.a. | n.a. | n.a. | n.a. | n.a. | n.a. | n.a. |
| Jakkamsetti et al. | n.a. | n.a. | n.a. | n.a. | n.a. | n.a. | n.a. | n.a. | n.a. | n.a. | n.a. | n.a. | n.a. |
| Jakkamsetti et al. | n.a. | n.a. | n.a. | n.a. | n.a. | n.a. | n.a. | n.a. | n.a. | n.a. | n.a. | n.a. | n.a. |
| Jermakowicz et al. | n.a. | n.a. | n.a. | n.a. | n.a. | n.a. | n.a. | n.a. | n.a. | n.a. | n.a. | n.a. | n.a. |
| Kaefer et al. | 1 | n.a. | n.a. | n.r. | n.a. | n.a. | n.a. | n.a. | n.a. | n.a. | n.a. | n.a. | n.a. |
| Katagiri et al. | n.a. | n.a. | n.a. | n.a. | n.a. | n.a. | n.a. | n.a. | n.a. | n.a. | n.a. | n.a. | n.a. |
| Kenny et al. | n.a. | n.a. | n.a. | n.a. | n.a. | n.a. | n.a. | n.a. | n.a. | n.a. | n.a. | n.a. | n.a. |
| Kim et al. B | n.a. | n.a. | n.a. | n.a. | n.a. | n.a. | n.a. | n.a. | n.a. | n.a. | n.a. | n.a. | n.a. |
| Kunori et al. | n.a. | n.a. | n.a. | n.a. | n.a. | n.a. | n.a. | n.a. | n.a. | n.a. | n.a. | n.a. | n.a. |
| Kyyriainen et al. | n.a. | n.a. | n.a. | n.a. | n.a. | n.a. | n.a. | n.a. | n.a. | n.a. | n.a. | n.a. | n.a. |
| Lee et al. C | n.a. | n.a. | n.a. | n.a. | n.a. | n.a. | n.a. | n.a. | n.a. | n.a. | n.a. | n.a. | n.a. |
| Levata et al. | n.a. | n.a. | n.a. | n.a. | n.a. | n.a. | n.a. | n.a. | n.a. | n.a. | n.a. | n.a. | n.a. |
| Li et al. A | n.a. | n.a. | n.a. | n.a. | n.a. | n.a. | n.a. | n.a. | n.a. | n.a. | n.a. | n.a. | n.a. |
| Li et al. D | n.a. | n.a. | n.a. | n.a. | n.a. | n.a. | n.a. | n.a. | n.a. | n.a. | n.a. | n.a. | n.a. |
| Li et al. B | n.a. | n.a. | n.a. | n.a. | n.a. | n.a. | n.a. | n.a. | n.a. | n.a. | n.a. | n.a. | n.a. |
| Luo et al. | n.a. | n.a. | n.a. | n.a. | n.a. | n.a. | n.a. | n.a. | n.a. | n.a. | n.a. | n.a. | n.a. |
| Lv et al. | n.a. | n.a. | n.a. | n.a. | n.a. | n.a. | n.a. | n.a. | n.a. | n.a. | n.a. | n.a. | n.a. |
| Ma et al. | n.a. | n.a. | n.a. | n.a. | n.a. | n.a. | n.a. | n.a. | n.a. | n.a. | n.a. | n.a. | n.a. |
| Mastrella et al. | 7 | n.a. | 12 | n.r. | n.a. | n.a. | n.a. | n.a. | n.a. | n.a. | n.a. | n.a. | n.a. |
| Mazza et al. | n.a. | n.a. | n.a. | n.a. | n.a. | n.a. | n.a. | n.a. | n.a. | n.a. | n.a. | n.a. | n.a. |
| Mittal et al. | n.a. | n.a. | n.a. | n.a. | n.a. | n.a. | n.a. | n.a. | n.a. | n.a. | n.a. | n.a. | n.a. |
| Mo et al. | n.a. | n.a. | n.a. | n.a. | n.a. | n.a. | n.a. | n.a. | n.a. | n.a. | n.a. | n.a. | n.a. |
| Mohammad et al. | 4 | n.a. | 24 | n.r. | n.a. | n.a. | n.a. | n.a. | n.a. | n.a. | n.a. | n.a. | n.a. |
| Mohammadipoor-Ghasemabad et al. | n.a. | n.a. | n.a. | n.a. | n.a. | n.a. | n.a. | n.a. | n.a. | n.a. | n.a. | n.a. | n.a. |
| Mohammadpoory et al. | n.a. | n.a. | n.a. | n.a. | n.a. | n.a. | n.a. | n.a. | n.a. | n.a. | n.a. | n.a. | n.a. |
| Moller et al. | 2 | 24h post-surgery | n.a. |  | n.a. | n.a. | n.a. | n.a. | n.a. | n.a. | n.a. | n.a. | n.a. |
| Murai et al. | n.a. | n.a. | n.a. | n.a. | n.a. | n.a. | n.a. | n.a. | n.a. | n.a. | n.a. | n.a. | n.a. |
| Njoku et al. | n.a. | n.a. | n.a. | n.a. | n.a. | n.a. | n.a. | n.a. | n.a. | n.a. | n.a. | n.a. | n.a. |
| O'Brien et al. | n.r | n.r. | n.r. | n.r. | n.a. | n.a. | n.a. | n.a. | n.a. | n.a. | n.a. | n.a. | n.a. |
| Ogun et al. | n.a. | n.a. | n.a. | n.a. | n.a. | n.a. | n.a. | n.a. | n.a. | n.a. | n.a. | n.a. | n.a. |
| Okada et al. | n.a. | n.a. | n.a. | n.a. | n.a. | n.a. | n.a. | n.a. | n.a. | n.a. | n.a. | n.a. | n.a. |
| Park et al. | 1 | n.a. | n.a. | n.r. | acetaminophen | per os | n.r. | postsurgically | n.a. | n.r. | n.a. | n.a. | solution |
| Pettibone et al. | n.a. | n.a. | n.a. | n.a. | n.a. | n.a. | n.a. | n.a. | n.a. | n.a. | n.a. | n.a. | n.a. |
| Pflüger et al. | n.a. | n.a. | n.a. | n.a. | n.a. | n.a. | n.a. | n.a. | n.a. | n.a. | n.a. | n.a. | n.a. |
| Qiao et al. | n.a. | n.a. | n.a. | n.a. | n.a. | n.a. | n.a. | n.a. | n.a. | n.a. | n.a. | n.a. | n.a. |
| Romoli et al. | n.a. | n.a. | n.a. | n.a. | n.a. | n.a. | n.a. | n.a. | n.a. | n.a. | n.a. | n.a. | n.a. |
| Russell et al. | n.a. | n.a. | n.a. | n.a. | n.a. | n.a. | n.a. | n.a. | n.a. | n.a. | n.a. | n.a. | n.a. |
| Sa et al. | 1 | n.a. | n.a. | n.r. | n.a. | n.a. | n.a. | n.a. | n.a. | n.a. | n.a. | n.a. | n.a. |
| Sharma et al. | n.a. | n.a. | n.a. | n.a. | n.a. | n.a. | n.a. | n.a. | n.a. | n.a. | n.a. | n.a. | n.a. |
| Shaver et al. | 1 | n.a. | n.a. | n.a. | n.a. | n.a. | n.a. | n.a. | n.a. | n.a. | n.a. | n.a. | n.a. |
| Shiuchi et al. | n.a. | n.a. | n.a. | n.a. | n.a. | n.a. | n.a. | n.a. | n.a. | n.a. | n.a. | n.a. | n.a. |
| Simader et al. | n.a. | n.a. | n.a. | n.a. | n.a. | n.a. | n.a. | n.a. | n.a. | n.a. | n.a. | n.a. | n.a. |
| Slezia et al. | n.a. | n.a. | n.a. | n.a. | n.a. | n.a. | n.a. | n.a. | n.a. | n.a. | n.a. | n.a. | n.a. |
| Souza et al. | 4 | n.a. | 24 | n.r. | n.a. | n.a. | n.a. | n.a. | n.a. | n.a. | n.a. | n.a. | n.a. |
| Souza et al. | n.a. | n.a. | n.a. | n.a. | n.a. | n.a. | n.a. | n.a. | n.a. | n.a. | n.a. | n.a. | n.a. |
| Stanchi et al. | n.a. | n.a. | n.a. | n.a. | n.a. | n.a. | n.a. | n.a. | n.a. | n.a. | n.a. | n.a. | n.a. |
| Stanojlovic et al. | n.a. | n.a. | n.a. | n.a. | n.a. | n.a. | n.a. | n.a. | n.a. | n.a. | n.a. | n.a. | n.a. |
| Sun et al. | 1 | n.a. | n.a. | n.r. | n.a. | n.a. | n.a. | n.a. | n.a. | n.a. | n.a. | n.a. | n.a. |
| Suzuki et al. | n.a. | n.a. | n.a. | n.a. | n.a. | n.a. | n.a. | n.a. | n.a. | n.a. | n.a. | n.a. | n.a. |
| Szonyi et al. | 1 | n.a. | n.a. | n.r. | n.a. | n.a. | n.a. | n.a. | n.a. | n.a. | n.a. | n.a. | n.a. |
| Szonyi et al. | n.a. | n.a. | n.a. | n.a. | n.a. | n.a. | n.a. | n.a. | n.a. | n.a. | n.a. | n.a. | n.a. |
| Tomov et al. | n.a. | n.a. | n.a. | n.a. | n.a. | n.a. | n.a. | n.a. | n.a. | n.a. | n.a. | n.a. | n.a. |
| Villa-Cedillo et al. | n.a. | n.a. | n.a. | n.a. | n.a. | n.a. | n.a. | n.a. | n.a. | n.a. | n.a. | n.a. | n.a. |
| Villasana et al. | n.a. | n.a. | n.a. | n.a. | n.a. | n.a. | n.a. | n.a. | n.a. | n.a. | n.a. | n.a. | n.a. |
| Wang et al. F | 4 | n.a. | 24 | n.r. | n.a. | n.a. | n.a. | n.a. | n.a. | n.a. | n.a. | n.a. | n.a. |
| Wang et al. G | n.a. | n.a. | n.a. | n.a. | n.a. | n.a. | n.a. | n.a. | n.a. | n.a. | n.a. | n.a. | n.a. |
| Wang et al. A | n.a. | n.a. | n.a. | n.a. | n.a. | n.a. | n.a. | n.a. | n.a. | n.a. | n.a. | n.a. | n.a. |
| Wang et al. H | n.a. | n.a. | n.a. | n.a. | n.a. | n.a. | n.a. | n.a. | n.a. | n.a. | n.a. | n.a. | n.a. |
| Wang et al. B | n.a. | n.a. | n.a. | n.a. | n.a. | n.a. | n.a. | n.a. | n.a. | n.a. | n.a. | n.a. | n.a. |
| Wang et al. D | n.a. | n.a. | n.a. | n.a. | n.a. | n.a. | n.a. | n.a. | n.a. | n.a. | n.a. | n.a. | n.a. |
| Wang et al. C | n.a. | n.a. | n.a. | n.a. | n.a. | n.a. | n.a. | n.a. | n.a. | n.a. | n.a. | n.a. | n.a. |
| Wang et al. E | n.a. | n.a. | n.a. | n.a. | n.a. | n.a. | n.a. | n.a. | n.a. | n.a. | n.a. | n.a. | n.a. |
| Wen et al. A | n.a. | n.a. | n.a. | n.a. | n.a. | n.a. | n.a. | n.a. | n.a. | n.a. | n.a. | n.a. | n.a. |
| Wen et al. B | 1 | n.a. | n.a. | n.r. | n.a. | n.a. | n.a. | n.a. | n.a. | n.a. | n.a. | n.a. | n.a. |
| Xu et al. | n.a. | n.a. | n.a. | n.a. | n.a. | n.a. | n.a. | n.a. | n.a. | n.a. | n.a. | n.a. | n.a. |
| Yang et al. | n.a. | n.a. | n.a. | n.a. | n.a. | n.a. | n.a. | n.a. | n.a. | n.a. | n.a. | n.a. | n.a. |
| Yeung et al. | n.a. | n.a. | n.a. | n.a. | n.a. | n.a. | n.a. | n.a. | n.a. | n.a. | n.a. | n.a. | n.a. |
| Zhang et al. C | n.a. | n.a. | n.a. | n.a. | n.a. | n.a. | n.a. | n.a. | n.a. | n.a. | n.a. | n.a. | n.a. |
| Zhang et al. B | n.a. | n.a. | n.a. | n.a. | n.a. | n.a. | n.a. | n.a. | n.a. | n.a. | n.a. | n.a. | n.a. |
| Zhang et al. A | n.a. | n.a. | n.a. | n.a. | n.a. | n.a. | n.a. | n.a. | n.a. | n.a. | n.a. | n.a. | n.a. |
| Zhao et al. | n.a. | n.a. | n.a. | n.a. | n.a. | n.a. | n.a. | n.a. | n.a. | n.a. | n.a. | n.a. | n.a. |

| study ID | analgesia opioid administered? (yes / no OR n.r.) | drug / compound (name OR n.a.) | administration route  (s.c. OR i.m. OR i.p. OR per os OR n.a.) | dosage  (mg/kg) OR n.r. OR n.a. | timepoint of first administration  (pre-, intra-, postsurgically OR n.a.) | if reported: immediately before surgery OR immediately after surgery OR n.r. OR n.a. | administered how many times in total?  (number OR n.a.) | if reported: administration interval  (morning after surgery OR 24h after surgery OR n.r. OR n.a.) | if reported: administration interval  (h post first administration OR n.r. OR n.a.) | if reported:  pharmaceutical formulation (e.g. solution OR n.r. OR n.a.) | other analgesics used?  E.g. metamizol (dipyrone) (yes / no OR n.r.) | if so: drug / compound  (name OR n.a.) | multimodal approaches  (total number of used compound groups (analgesics and local anesthetics))  (excluding agents used for general anesthesia; if e.g. two local anesthetics administered it is counted as one since only one substance group)  (number) | other drugs (other than analgesics) used?  If so: compound (n.a. OR name) |
| --- | --- | --- | --- | --- | --- | --- | --- | --- | --- | --- | --- | --- | --- | --- |
| Aldehri et al. | yes | buprenorphine | s.c. | 0,1 | presurgically | n.a. | 1 | n.a. | n.a. | n.r. | no | n.a. | 1 | n.a. |
| Asan et al. | no | n.a. | n.a. | n.a. | n.a. | n.a. | n.a. | n.a. | n.a. | n.a. | no | n.a. | 0 | n.a. |
| Baud et al. | no | n.a. | n.a. | n.a. | n.a. | n.a. | n.a. | n.a. | n.a. | n.a. | no | n.a. | 0 | n.a. |
| Bazzu et al. | no | n.a. | n.a. | n.a. | n.a. | n.a. | n.a. | n.a. | n.a. | n.a. | no | n.a. | 1 | n.a. |
| Bertoglio et al. | yes | buprenorphine | s.c. | 0,01 | postsurgically | immediately after surgery | 1 | n.a. | n.a. | n.a. | no | n.a. | 1 | n.a. |
| Bleimeister et al. | no | n.a. | n.a. | n.a. | n.a. | n.a. | n.a. | n.a. | n.a. | n.a. | no | n.a. | 0 | n.a. |
| Bukhtiyarova et al. | yes | buprenorphine | s.c. | 0,1 | presurgically | immediately before surgery | 1 | n.a. | n.a. | n.a. | no | n.a. | 1 | n.a. |
| Burgdorf et al. | no | n.a. | n.a. | n.a. | n.a. | n.a. | n.a. | n.a. | n.a. | n.a. | no | n.a. | 0 | n.a. |
| Casanova-Carvajal et al. | no | n.a. | n.a. | n.a. | n.a. | n.a. | n.a. | n.a. | n.a. | n.a. | no | n.a. | 0 | n.a. |
| Chen et al. C | no | n.a. | n.a. | n.a. | n.a. | n.a. | n.a. | n.a. | n.a. | n.a. | no | n.a. | 0 | n.a. |
| Chen et al. E | no | n.a. | n.a. | n.a. | n.a. | n.a. | n.a. | n.a. | n.a. | n.a. | no | n.a. | 0 | n.a. |
| Chen et al. A | no | n.a. | n.a. | n.a. | n.a. | n.a. | n.a. | n.a. | n.a. | n.a. | no | n.a. | 0 | n.a. |
| Chen et al. B | no | n.a. | n.a. | n.a. | n.a. | n.a. | n.a. | n.a. | n.a. | n.a. | no | n.a. | 0 | n.a. |
| Chen et al. F | no | n.a. | n.a. | n.a. | n.a. | n.a. | n.a. | n.a. | n.a. | n.a. | no | n.a. | 0 | n.a. |
| Chitturi et al. | no | n.a. | n.a. | n.a. | n.a. | n.a. | n.a. | n.a. | n.a. | n.a. | no | n.a. | 0 | n.a. |
| Christiaen et al. | no | n.a. | n.a. | n.a. | n.a. | n.a. | n.a. | n.a. | n.a. | n.a. | no | n.a. | 1 | n.a. |
| Colangeli et al. | no | n.a. | n.a. | n.a. | n.a. | n.a. | n.a. | n.a. | n.a. | n.a. | no | n.a. | 0 | n.a. |
| da Silva Pacheco et al. | no | n.a. | n.a. | n.a. | n.a. | n.a. | n.a. | n.a. | n.a. | n.a. | no | n.a. | 0 | n.a. |
| Daglas et al. | no | n.a. | n.a. | n.a. | n.a. | n.a. | n.a. | n.a. | n.a. | n.a. | no | n.a. | 0 | n.a. |
| Dal-Pont et al. | no | n.a. | n.a. | n.a. | n.a. | n.a. | n.a. | n.a. | n.a. | n.a. | no | n.a. | 0 | n.a. |
| Delaney et al. | no | n.a. | n.a. | n.a. | n.a. | n.a. | n.a. | n.a. | n.a. | n.a. | no | n.a. | 0 | n.a. |
| Dreier et al. | no | n.a. | n.a. | n.a. | n.a. | n.a. | n.a. | n.a. | n.a. | n.a. | no | n.a. | 0 | n.a. |
| Du et al. | no | n.a. | n.a. | n.a. | n.a. | n.a. | n.a. | n.a. | n.a. | n.a. | no | n.a. | 0 | n.a. |
| Duveau et al. | yes | buprenorphine | i.p. | 0,01 | postsurgically | immediately after surgery | 1 | n.a. | n.a. | n.r. | no | n.a. | 1 | n.a. |
| Etter et al. | no | n.a. | n.a. | n.a. | n.a. | n.a. | n.a. | n.a. | n.a. | n.a. | no | n.a. | 0 | n.a. |
| Ewell et al. | no | n.a. | n.a. | n.a. | n.a. | n.a. | n.a. | n.a. | n.a. | n.a. | no | n.a. | 0 | n.a. |
| Farakhor et al. | no | n.a. | n.a. | n.a. | n.a. | n.a. | n.a. | n.a. | n.a. | n.a. | no | n.a. | 1 | n.a. |
| Farooq et al. | no | n.a. | n.a. | n.a. | n.a. | n.a. | n.a. | n.a. | n.a. | n.a. | no | n.a. | 0 | n.a. |
| Fiath et al. | no | n.a. | n.a. | n.a. | n.a. | n.a. | n.a. | n.a. | n.a. | n.a. | no | n.a. | 0 | n.a. |
| Fortress et al. | no | n.a. | n.a. | n.a. | n.a. | n.a. | n.a. | n.a. | n.a. | n.a. | no | n.a. | 0 | n.a. |
| Hu et al. | no | n.a. | n.a. | n.a. | n.a. | n.a. | n.a. | n.a. | n.a. | n.a. | no | n.a. | 0 | n.a. |
| Ilieva et al. | no | n.a. | n.a. | n.a. | n.a. | n.a. | n.a. | n.a. | n.a. | n.a. | no | n.a. | 0 | n.a. |
| Jackson et al. | yes | buprenorphine | s.c. | 0,05 | postsurgically | immediately after surgery | 1 | n.a. | n.a. | n.r. | no | n.a. | 1 | n.a. |
| Jakkamsetti et al. | no | n.a. | n.a. | n.a. | n.a. | n.a. | n.a. | n.a. | n.a. | n.a. | no | n.a. | 0 | n.a. |
| Jakkamsetti et al. | yes | buprenorphine | n.r. | 0,05 | postsurgically | immediately after surgery | 1 | n.a. | n.a. | n.r. | no | n.a. | 1 | n.a. |
| Jermakowicz et al. | yes | buprenorphine | s.c. | 0,2 | postsurgically | immediately after surgery | 7 | morning after surgery | 12 | n.r. | no | n.a. | 1 | n.a. |
| Kaefer et al. | yes | buprenorphine | n.r. | 0,1 | presurgically | n.r. | 1 | n.a. | n.a. | n.r. | no | n.a. | 2 | n.a. |
| Katagiri et al. | no | n.a. | n.a. | n.a. | n.a. | n.a. | n.a. | n.a. | n.a. | n.a. | no | n.a. | 0 | n.a. |
| Kenny et al. | no | n.a. | n.a. | n.a. | n.a. | n.a. | n.a. | n.a. | n.a. | n.a. | no | n.a. | 0 | n.a. |
| Kim et al. B | no | n.a. | n.a. | n.a. | n.a. | n.a. | n.a. | n.a. | n.a. | n.a. | no | n.a. | 0 | n.a. |
| Kunori et al. | no | n.a. | n.a. | n.a. | n.a. | n.a. | n.a. | n.a. | n.a. | n.a. | no | n.a. | 0 | n.a. |
| Kyyriainen et al. | no | n.a. | n.a. | n.a. | n.a. | n.a. | n.a. | n.a. | n.a. | n.a. | no | n.a. | 0 | n.a. |
| Lee et al. C | no | n.a. | n.a. | n.a. | n.a. | n.a. | n.a. | n.a. | n.a. | n.a. | no | n.a. | 0 | n.a. |
| Levata et al. | no | n.a. | n.a. | n.a. | n.a. | n.a. | n.a. | n.a. | n.a. | n.a. | no | n.a. | 0 | n.a. |
| Li et al. A | no | n.a. | n.a. | n.a. | n.a. | n.a. | n.a. | n.a. | n.a. | n.a. | no | n.a. | 0 | n.a. |
| Li et al. D | no | n.a. | n.a. | n.a. | n.a. | n.a. | n.a. | n.a. | n.a. | n.a. | no | n.a. | 0 | n.a. |
| Li et al. B | no | n.a. | n.a. | n.a. | n.a. | n.a. | n.a. | n.a. | n.a. | n.a. | no | n.a. | 0 | n.a. |
| Luo et al. | no | n.a. | n.a. | n.a. | n.a. | n.a. | n.a. | n.a. | n.a. | n.a. | no | n.a. | 0 | n.a. |
| Lv et al. | no | n.a. | n.a. | n.a. | n.a. | n.a. | n.a. | n.a. | n.a. | n.a. | no | n.a. | 0 | n.a. |
| Ma et al. | no | n.a. | n.a. | n.a. | n.a. | n.a. | n.a. | n.a. | n.a. | n.a. | no | n.a. | 0 | n.a. |
| Mastrella et al. | no | n.a. | n.a. | n.a. | n.a. | n.a. | n.a. | n.a. | n.a. | n.a. | no | n.a. | 1 | n.a. |
| Mazza et al. | yes | buprenorphine | n.r. | 0,05 | presurgically | n.r. | 1 | n.a. | n.a. | n.r. | no | n.a. | 1 | n.a. |
| Mittal et al. | no | n.a. | n.a. | n.a. | n.a. | n.a. | n.a. | n.a. | n.a. | n.a. | no | n.a. | 0 | n.a. |
| Mo et al. | no | n.a. | n.a. | n.a. | n.a. | n.a. | n.a. | n.a. | n.a. | n.a. | no | n.a. | 0 | n.a. |
| Mohammad et al. | no | n.a. | n.a. | n.a. | n.a. | n.a. | n.a. | n.a. | n.a. | n.a. | no | n.a. | 1 | n.a. |
| Mohammadipoor-Ghasemabad et al. | no | n.a. | n.a. | n.a. | n.a. | n.a. | n.a. | n.a. | n.a. | n.a. | no | n.a. | 0 | n.a. |
| Mohammadpoory et al. | no | n.a. | n.a. | n.a. | n.a. | n.a. | n.a. | n.a. | n.a. | n.a. | no | n.a. | 0 | n.a. |
| Moller et al. | no | n.a. | n.a. | n.a. | n.a. | n.a. | n.a. | n.a. | n.a. | n.a. | no | n.a. | 1 | epinephrine |
| Murai et al. | no | n.a. | n.a. | n.a. | n.a. | n.a. | n.a. | n.a. | n.a. | n.a. | no | n.a. | 0 | n.a. |
| Njoku et al. | no | n.a. | n.a. | n.a. | n.a. | n.a. | n.a. | n.a. | n.a. | n.a. | no | n.a. | 0 | n.a. |
| O'Brien et al. | no | n.a. | n.a. | n.a. | n.a. | n.a. | n.a. | n.a. | n.a. | n.a. | no | n.a. | 1 | n.a. |
| Ogun et al. | no | n.a. | n.a. | n.a. | n.a. | n.a. | n.a. | n.a. | n.a. | n.a. | no | n.a. | 0 | n.a. |
| Okada et al. | yes | butorphanol | i.p. | 2,5 | presurgically | immediately before surgery | 1 | n.a. | n.a. | solution | no | n.a. | 1 | n.a. |
| Park et al. | no | n.a. | n.a. | n.a. | n.a. | n.a. | n.a. | n.a. | n.a. | n.a. | no | n.a. | 2 | n.a. |
| Pettibone et al. | no | n.a. | n.a. | n.a. | n.a. | n.a. | n.a. | n.a. | n.a. | n.a. | no | n.a. | 0 | n.a. |
| Pflüger et al. | no | n.a. | n.a. | n.a. | n.a. | n.a. | n.a. | n.a. | n.a. | n.a. | no | n.a. | 0 | n.a. |
| Qiao et al. | no | n.a. | n.a. | n.a. | n.a. | n.a. | n.a. | n.a. | n.a. | n.a. | no | n.a. | 0 | n.a. |
| Romoli et al. | no | n.a. | n.a. | n.a. | n.a. | n.a. | n.a. | n.a. | n.a. | n.a. | no | n.a. | 0 | n.a. |
| Russell et al. | no | n.a. | n.a. | n.a. | n.a. | n.a. | n.a. | n.a. | n.a. | n.a. | no | n.a. | 0 | n.a. |
| Sa et al. | no | n.a. | n.a. | n.a. | n.a. | n.a. | n.a. | n.a. | n.a. | n.a. | no | n.a. | 1 | n.a. |
| Sharma et al. | no | n.a. | n.a. | n.a. | n.a. | n.a. | n.a. | n.a. | n.a. | n.a. | no | n.a. | 0 | n.a. |
| Shaver et al. | no | n.a. | n.a. | n.a. | n.a. | n.a. | n.a. | n.a. | n.a. | n.a. | no | n.a. | 1 | n.a. |
| Shiuchi et al. | no | n.a. | n.a. | n.a. | n.a. | n.a. | n.a. | n.a. | n.a. | n.a. | no | n.a. | 0 | n.a. |
| Simader et al. | no | n.a. | n.a. | n.a. | n.a. | n.a. | n.a. | n.a. | n.a. | n.a. | no | n.a. | 0 | n.a. |
| Slezia et al. | no | n.a. | n.a. | n.a. | n.a. | n.a. | n.a. | n.a. | n.a. | n.a. | no | n.a. | 0 | n.a. |
| Souza et al. | no | n.a. | n.a. | n.a. | n.a. | n.a. | n.a. | n.a. | n.a. | n.a. | no | n.a. | 1 | n.a. |
| Souza et al. | no | n.a. | n.a. | n.a. | n.a. | n.a. | n.a. | n.a. | n.a. | n.a. | no | n.a. | 0 | n.a. |
| Stanchi et al. | no | n.a. | n.a. | n.a. | n.a. | n.a. | n.a. | n.a. | n.a. | n.a. | no | n.a. | 0 | n.a. |
| Stanojlovic et al. | no | n.a. | n.a. | n.a. | n.a. | n.a. | n.a. | n.a. | n.a. | n.a. | no | n.a. | 0 | n.a. |
| Sun et al. | no | n.a. | n.a. | n.a. | n.a. | n.a. | n.a. | n.a. | n.a. | n.a. | no | n.a. | 1 | n.a. |
| Suzuki et al. | no | n.a. | n.a. | n.a. | n.a. | n.a. | n.a. | n.a. | n.a. | n.a. | no | n.a. | 0 | n.a. |
| Szonyi et al. | no | n.a. | n.a. | n.a. | n.a. | n.a. | n.a. | n.a. | n.a. | n.a. | no | n.a. | 1 | n.a. |
| Szonyi et al. | yes | buprenorphine | i.p. | 0,1 | postsurgically | immediately after surgery | 1 | n.a. | n.a. | n.r. | no | n.a. | 1 | n.a. |
| Tomov et al. | no | n.a. | n.a. | n.a. | n.a. | n.a. | n.a. | n.a. | n.a. | n.a. | no | n.a. | 0 | n.a. |
| Villa-Cedillo et al. | no | n.a. | n.a. | n.a. | n.a. | n.a. | n.a. | n.a. | n.a. | n.a. | no | n.a. | 0 | n.a. |
| Villasana et al. | no | n.a. | n.a. | n.a. | n.a. | n.a. | n.a. | n.a. | n.a. | n.a. | no | n.a. | 0 | n.a. |
| Wang et al. F | yes | buprenorphine | s.c. | 0,1 | postsurgically | n.a. | 10 | n.a. | 8 | n.r. | no | n.a. | 2 | n.a. |
| Wang et al. G | no | n.a. | n.a. | n.a. | n.a. | n.a. | n.a. | n.a. | n.a. | n.a. | no | n.a. | 0 | n.a. |
| Wang et al. A | no | n.a. | n.a. | n.a. | n.a. | n.a. | n.a. | n.a. | n.a. | n.a. | no | n.a. | 0 | n.a. |
| Wang et al. H | no | n.a. | n.a. | n.a. | n.a. | n.a. | n.a. | n.a. | n.a. | n.a. | no | n.a. | 0 | n.a. |
| Wang et al. B | no | n.a. | n.a. | n.a. | n.a. | n.a. | n.a. | n.a. | n.a. | n.a. | no | n.a. | 0 | n.a. |
| Wang et al. D | no | n.a. | n.a. | n.a. | n.a. | n.a. | n.a. | n.a. | n.a. | n.a. | no | n.a. | 0 | n.a. |
| Wang et al. C | no | n.a. | n.a. | n.a. | n.a. | n.a. | n.a. | n.a. | n.a. | n.a. | no | n.a. | 0 | n.a. |
| Wang et al. E | no | n.a. | n.a. | n.a. | n.a. | n.a. | n.a. | n.a. | n.a. | n.a. | no | n.a. | 0 | n.a. |
| Wen et al. A | yes | buprenorphine | s.c. | 2 | intrasurgically | n.a. | n.r | n.a. | 6 to 12 | n.r. | no | n.a. | 1 | n.a. |
| Wen et al. B | no | n.a. | n.a. | n.a. | n.a. | n.a. | n.a. | n.a. | n.a. | n.a. | no | n.a. | 1 | n.a. |
| Xu et al. | no | n.a. | n.a. | n.a. | n.a. | n.a. | n.a. | n.a. | n.a. | n.a. | no | n.a. | 0 | n.a. |
| Yang et al. | no | n.a. | n.a. | n.a. | n.a. | n.a. | n.a. | n.a. | n.a. | n.a. | no | n.a. | 0 | n.a. |
| Yeung et al. | no | n.a. | n.a. | n.a. | n.a. | n.a. | n.a. | n.a. | n.a. | n.a. | no | n.a. | 0 | atipamezole |
| Zhang et al. C | no | n.a. | n.a. | n.a. | n.a. | n.a. | n.a. | n.a. | n.a. | n.a. | no | n.a. | 0 | n.a. |
| Zhang et al. B | no | n.a. | n.a. | n.a. | n.a. | n.a. | n.a. | n.a. | n.a. | n.a. | no | n.a. | 0 | n.a. |
| Zhang et al. A | no | n.a. | n.a. | n.a. | n.a. | n.a. | n.a. | n.a. | n.a. | n.a. | no | n.a. | 0 | n.a. |
| Zhao et al. | yes | buprenorphine | n.r. | n.r. | presurgically | n.r. | 1 | n.a. | n.a. | n.r. | no | n.a. | 1 | n.a. |

| study ID | antibiotic agent  used? If so, list drug/ compound (name OR unclear OR no OR n.r.) | administration route  (s.c. OR i.m. OR topically OR per os OR n.a.) | specific monitoring reported? (heartrate, breathing, body temperature) (yes / no) | if so, specify: e.g. heartbeat, pulse, breathing, body temperature OR n.a. | peri-operative care reported?  (yes / no) | if so, specify:  e.g. body temperature maintained, saline drip OR n.a. | non-pharmacological measures for pain management reported? (probably not) (yes / no) | if so, specify: e.g. cooling of incision, non-pharmacological substances applied OR n.a. | refinement measures reported?  (yes / no) | if so, specify:  e.g. recovery on heating pad, bolus of saline, housed individually after surgery OR n.a. |
| --- | --- | --- | --- | --- | --- | --- | --- | --- | --- | --- |
| Aldehri et al. | unclear | n.a. | no | n.a. | yes | eye ointment applied | no | n.a. | no | n.a. |
| Asan et al. | unclear | n.a. | yes | heart rate, blood-oxygen, body temperature | yes | body temperature maintained | no | n.a. | no | n.a. |
| Baud et al. | unclear | n.a. | no | n.a. | no | n.a. | no | n.a. | yes | housed individually |
| Bazzu et al. | unclear | n.a. | yes | breathing, body temperature | yes | body temperature maintained, eye ointment applied | no | n.a. | yes | recover in climate controlled (36 degrees Celsius) chamber, s.c. bolus of saline, housed individually after surgery |
| Bertoglio et al. | unclear | n.a. | no | n.a. | no | n.a. | no | n.a. | yes | 10 ml/kg Hartmann's solution after surgery, body temperature maintained until ambulatory |
| Bleimeister et al. | unclear | n.a. | yes | breathing, body temperature | yes | body temperature maintained | no | n.a. | no | n.a. |
| Bukhtiyarova et al. | unclear | n.a. | no | n.a. | no | n.a. | no | n.a. | yes | bolus of saline after surgery |
| Burgdorf et al. | unclear | n.a. | no | n.a. | no | n.a. | no | n.a. | no | n.a. |
| Casanova-Carvajal et al. | unclear | n.a. | yes | body temperature | yes | body temperature maintained | no | n.a. | no | n.a. |
| Chen et al. C | unclear | n.a. | no | n.a. | no | n.a. | no | n.a. | no | n.a. |
| Chen et al. E | unclear | n.a. | no | n.a. | no | n.a. | no | n.a. | no | n.a. |
| Chen et al. A | unclear | n.a. | no | n.a. | no | n.a. | no | n.a. | no | n.a. |
| Chen et al. B | unclear | n.a. | no | n.a. | no | n.a. | no | n.a. | yes | recovery on heating pad |
| Chen et al. F | unclear | n.a. | no | n.a. | yes | body temperature maintained | no | n.a. | no | n.a. |
| Chitturi et al. | unclear | n.a. | no | n.a. | no | n.a. | no | n.a. | no | n.a. |
| Christiaen et al. | unclear | n.a. | no | n.a. | no | n.a. | no | n.a. | no | n.a. |
| Colangeli et al. | unclear | n.a. | no | n.a. | no | n.a. | no | n.a. | yes | housed individually |
| da Silva Pacheco et al. | unclear | n.a. | no | n.a. | no | n.a. | no | n.a. | no | n.a. |
| Daglas et al. | unclear | n.a. | no | n.a. | no | n.a. | no | n.a. | no | n.a. |
| Dal-Pont et al. | unclear | n.a. | no | n.a. | no | n.a. | no | n.a. | no | n.a. |
| Delaney et al. | unclear | n.a. | no | n.a. | no | n.a. | no | n.a. | no | n.a. |
| Dreier et al. | unclear | n.a. | yes | breathing, systemic arterial pressure, body temperature, pO2, blood pH | yes | body temperature maintained | no | n.a. | no | n.a. |
| Du et al. | unclear | n.a. | no | n.a. | no | n.a. | no | n.a. | no | n.a. |
| Duveau et al. | unclear | n.a. | yes | breathing, cardiac rhythm, body temperature | yes | body temperature maintained, eye ointment applied | no | n.a. | yes | housed individually |
| Etter et al. | unclear | n.a. | no | n.a. | no | n.a. | no | n.a. | no | n.a. |
| Ewell et al. | unclear | n.a. | no | n.a. | no | n.a. | no | n.a. | no | n.a. |
| Farakhor et al. | enrofloxacin | n.r. | no | n.a. | no | n.a. | no | n.a. | no | n.a. |
| Farooq et al. | unclear | n.a. | yes | depth of anesthesia | yes | body temperature maintained | no | n.a. | no | n.a. |
| Fiath et al. | unclear | n.a. | yes | body temperature | yes | body temperature maintained | no | n.a. | no | n.a. |
| Fortress et al. | unclear | n.a. | no | n.a. | no | n.a. | no | n.a. | no | n.a. |
| Hu et al. | unclear | n.a. | no | n.a. | no | n.a. | no | n.a. | no | n.a. |
| Ilieva et al. | unclear | n.a. | no | n.a. | no | n.a. | no | n.a. | yes | antiseptic powder, lactated Ringer's solution 2ml/100g/day s.c., vitamins, moistened rat chow |
| Jackson et al. | enrofloxacin | i.m. | no | n.a. | no | n.a. | no | n.a. | yes | bolus of saline after surgery |
| Jakkamsetti et al. | unclear | n.a. | yes | body temperature | yes | body temperature maintained | no | n.a. | no | n.a. |
| Jakkamsetti et al. | unclear | n.a. | no | n.a. | no | n.a. | no | n.a. | no | n.a. |
| Jermakowicz et al. | gentamycin | s.c. | no | n.a. | no | n.a. | no | n.a. | no | n.a. |
| Kaefer et al. | unclear | n.a. | no | n.a. | no | n.a. | no | n.a. | yes | housed individually |
| Katagiri et al. | unclear | n.a. | no | n.a. | no | n.a. | no | n.a. | no | n.a. |
| Kenny et al. | unclear | n.a. | yes | body temperature | yes | body temperature maintained | no | n.a. | yes | recovery under oxygen hood |
| Kim et al. B | unclear | n.a. | no | n.a. | no | n.a. | no | n.a. | no | n.a. |
| Kunori et al. | unclear | n.a. | no | n.a. | no | n.a. | no | n.a. | no | n.a. |
| Kyyriainen et al. | unclear | n.a. | no | n.a. | no | n.a. | no | n.a. | no | n.a. |
| Lee et al. C | unclear | n.a. | yes | body temperature | yes | body temperature maintained | no | n.a. | no | n.a. |
| Levata et al. | unclear | n.a. | no | n.a. | yes | heating pad | no | n.a. | no | n.a. |
| Li et al. A | unclear | n.a. | no | n.a. | no | n.a. | no | n.a. | no | n.a. |
| Li et al. D | unclear | n.a. | no | n.a. | no | n.a. | no | n.a. | no | n.a. |
| Li et al. B | unclear | n.a. | no | n.a. | no | n.a. | no | n.a. | no | n.a. |
| Luo et al. | unclear | n.a. | no | n.a. | no | n.a. | no | n.a. | no | n.a. |
| Lv et al. | unclear | n.a. | yes | body temperature | yes | body temperature maintained | no | n.a. | no | n.a. |
| Ma et al. | unclear | n.a. | no | n.a. | no | n.a. | no | n.a. | no | n.a. |
| Mastrella et al. | unclear | n.a. | no | n.a. | yes | heating pad, eye ointment | no | n.a. | no | n.a. |
| Mazza et al. | unclear | n.a. | no | n.a. | no | n.a. | no | n.a. | no | n.a. |
| Mittal et al. | unclear | n.a. | no | n.a. | no | n.a. | no | n.a. | no | n.a. |
| Mo et al. | unclear | n.a. | no | n.a. | no | n.a. | no | n.a. | no | n.a. |
| Mohammad et al. | unclear | n.a. | no | n.a. | no | n.a. | no | n.a. | no | n.a. |
| Mohammadipoor-Ghasemabad et al. | unclear | n.a. | no | n.a. | no | n.a. | no | n.a. | no | n.a. |
| Mohammadpoory et al. | unclear | n.a. | no | n.a. | no | n.a. | no | n.a. | no | n.a. |
| Moller et al. | marbofloxacin | s.c. | no | n.a. | no | n.a. | no | n.a. | no | n.a. |
| Murai et al. | unclear | n.a. | no | n.a. | no | n.a. | no | n.a. | no | n.a. |
| Njoku et al. | unclear | n.a. | yes | body temperature | yes | body temperature maintained | no | n.a. | no | n.a. |
| O'Brien et al. | unclear | n.a. | no | n.a. | yes | eye ointment applied | no | n.a. | yes | recovery in warm cage, housed individually |
| Ogun et al. | unclear | n.a. | no | n.a. | no | n.a. | no | n.a. | no | n.a. |
| Okada et al. | unclear | n.a. | no | n.a. | no | n.a. | no | n.a. | no | n.a. |
| Park et al. | enrofloxacin | s.c. | yes | body temperature | yes | body temperature maintained | no | n.a. | yes | housed individually |
| Pettibone et al. | unclear | n.a. | no | n.a. | no | n.a. | no | n.a. | no | n.a. |
| Pflüger et al. | unclear | n.a. | yes | breathing, reflexes, level of anesthesia | no | n.a. | no | n.a. | yes | pair housed after surgery |
| Qiao et al. | unclear | n.a. | no | n.a. | no | n.a. | no | n.a. | no | n.a. |
| Romoli et al. | unclear | n.a. | no | n.a. | no | n.a. | no | n.a. | no | n.a. |
| Russell et al. | n.r. | n.r. | yes | body temperature, pulse oximetry | yes | body temperature maintained | no | n.a. | no | n.a. |
| Sa et al. | streptomycin | i.m. | no | n.a. | no | n.a. | no | n.a. | no | n.a. |
| Sharma et al. | gentamycin | i.p. | no | n.a. | yes | heating pad, eye ointment | no | n.a. | yes | warmed until ambulatory, housed individually, clinical signs monitored |
| Shaver et al. | triple antibiotic | n.a. | no | n.a. | no | n.a. | no | n.a. | no | housed individually |
| Shiuchi et al. | unclear | n.a. | no | n.a. | no | n.a. | no | n.a. | no | n.a. |
| Simader et al. | unclear | n.a. | no | n.a. | no | n.a. | no | n.a. | no | n.a. |
| Slezia et al. | unclear | n.a. | yes | reflexes, whisker movement, breathing, body temperature | yes | body temperature maintained | no | n.a. | no | n.a. |
| Souza et al. | ampicillin | i.p. | yes | body temperature, tail pinch reflex | yes | body temperature maintained | no | n.a. | no | n.a. |
| Souza et al. | unclear | n.a. | no | n.a. | no | n.a. | no | n.a. | no | n.a. |
| Stanchi et al. | unclear | n.a. | no | n.a. | no | n.a. | no | n.a. | yes | recovery on heating pad |
| Stanojlovic et al. | unclear | n.a. | no | n.a. | no | n.a. | no | n.a. | no | n.a. |
| Sun et al. | unclear | n.a. | yes | body temperature, breathing | yes | eye ointment applied, body temperature maintained | no | n.a. | yes | recovery on heating pad |
| Suzuki et al. | unclear | n.a. | no | n.a. | no | n.a. | no | n.a. | no | n.a. |
| Szonyi et al. | unclear | n.a. | no | n.a. | no | n.a. | no | n.a. | yes | bolus of saline after surgery |
| Szonyi et al. | unclear | n.a. | no | n.a. | no | n.a. | no | n.a. | yes | bolus of saline after surgery |
| Tomov et al. | unclear | n.a. | no | n.a. | yes | eye ointment applied | no | n.a. | yes | soaked chew, bolus of saline after surgery |
| Villa-Cedillo et al. | unclear | n.a. | no | n.a. | no | n.a. | no | n.a. | no | n.a. |
| Villasana et al. | unclear | n.a. | no | n.a. | no | n.a. | no | n.a. | yes | recovery on heating pad |
| Wang et al. F | chloramphenicol | topically | no | n.a. | no | n.a. | no | n.a. | yes | bolus of saline injected every 24h |
| Wang et al. G | erythromycin | topically | no | n.a. | no | n.a. | no | n.a. | no | n.a. |
| Wang et al. A | unclear | n.a. | yes | body temperature | yes | body temperature maintained | no | n.a. | no | n.a. |
| Wang et al. H | unclear | n.a. | no | n.a. | no | n.a. | no | n.a. | no | n.a. |
| Wang et al. B | unclear | n.a. | no | n.a. | no | n.a. | no | n.a. | no | n.a. |
| Wang et al. D | unclear | n.a. | no | n.a. | no | n.a. | no | n.a. | no | n.a. |
| Wang et al. C | unclear | n.a. | no | n.a. | no | n.a. | no | n.a. | no | n.a. |
| Wang et al. E | unclear | n.a. | no | n.a. | no | n.a. | no | n.a. | no | n.a. |
| Wen et al. A | unclear | n.a. | yes | eye reflex, pain reflex, body temperature | yes | body temperature maintained | no | n.a. | yes | recovery on heating pad |
| Wen et al. B | unclear | n.a. | no | n.a. | no | n.a. | no | n.a. | no | n.a. |
| Xu et al. | unclear | n.a. | yes | body temperature | yes | body temperature maintained | no | n.a. | yes | housed individually |
| Yang et al. | unclear | n.a. | yes | arterial blood pressure, blood gases, brain temperature, body temperature | yes | body temperature maintained | no | n.a. | no | n.a. |
| Yeung et al. | unclear | n.a. | no | n.a. | no | n.a. | no | n.a. | no | n.a. |
| Zhang et al. C | unclear | n.a. | no | n.a. | no | n.a. | no | n.a. | no | n.a. |
| Zhang et al. B | unclear | n.a. | yes | body temperature | yes | body temperature maintained | no | n.a. | no | n.a. |
| Zhang et al. A | penicillin | i.p. | no | n.a. | no | n.a. | no | n.a. | no | n.a. |
| Zhao et al. | unclear | n.a. | no | n.a. | no | n.a. | no | n.a. | no | n.a. |

| study ID | assessment of  analgesic efficacy post surgery reported?  (yes / no) | parameters testing efficacy of pain/stress reducing  measures post surgery reported?  (e.g. Mouse Grimace Scale) (yes / no) | if so:  which parameters are reported?  (name of method OR n.a.) | blinding reported?  (yes / no) | randomization reported?  (yes / no) | Power Analysis reported? (yes / no) | if so: Power Analysis reported in detail? (yes / no OR n.a.) |
| --- | --- | --- | --- | --- | --- | --- | --- |
| Aldehri et al. | no | no | n.a. | no | yes | no | n.a. |
| Asan et al. | no | no | n.a. | no | no | no | n.a. |
| Baud et al. | no | no | n.a. | no | no | no | n.a. |
| Bazzu et al. | no | no | n.a. | no | no | no | n.a. |
| Bertoglio et al. | no | no | n.a. | no | no | no | n.a. |
| Bleimeister et al. | no | no | n.a. | yes | yes | no | n.a. |
| Bukhtiyarova et al. | no | no | n.a. | no | no | no | n.a. |
| Burgdorf et al. | no | no | n.a. | no | yes | no | n.a. |
| Casanova-Carvajal et al. | no | no | n.a. | no | no | no | n.a. |
| Chen et al. C | no | no | n.a. | no | yes | no | no |
| Chen et al. E | no | no | n.a. | no | yes | no | n.a. |
| Chen et al. A | no | no | n.a. | yes | no | no | n.a. |
| Chen et al. B | no | no | n.a. | yes | yes | no | n.a. |
| Chen et al. F | no | no | n.a. | yes | yes | no | n.a. |
| Chitturi et al. | no | no | n.a. | no | yes | no | n.a. |
| Christiaen et al. | no | no | n.a. | no | yes | no | n.a. |
| Colangeli et al. | no | no | n.a. | no | no | no | n.a. |
| da Silva Pacheco et al. | no | no | n.a. | no | no | no | n.a. |
| Daglas et al. | no | no | n.a. | yes | yes | no | n.a. |
| Dal-Pont et al. | no | no | n.a. | no | no | no | n.a. |
| Delaney et al. | no | no | n.a. | yes | yes | no | n.a. |
| Dreier et al. | no | no | n.a. | no | no | no | no |
| Du et al. | no | no | n.a. | no | yes | no | n.a. |
| Duveau et al. | no | no | n.a. | yes | yes | no | n.a. |
| Etter et al. | no | no | n.a. | yes | yes | no | n.a. |
| Ewell et al. | no | no | n.a. | no | no | no | n.a. |
| Farakhor et al. | no | no | n.a. | no | no | no | n.a. |
| Farooq et al. | no | no | n.a. | no | no | no | n.a. |
| Fiath et al. | no | no | n.a. | no | no | no | n.a. |
| Fortress et al. | no | no | n.a. | no | no | no | n.a. |
| Hu et al. | no | no | n.a. | no | yes | no | n.a. |
| Ilieva et al. | no | no | n.a. | no | no | no | n.a. |
| Jackson et al. | no | no | n.a. | no | yes | no | n.a. |
| Jakkamsetti et al. | no | no | n.a. | no | yes | no | n.a. |
| Jakkamsetti et al. | no | no | n.a. | yes | yes | no | n.a. |
| Jermakowicz et al. | no | no | n.a. | no | no | no | n.a. |
| Kaefer et al. | no | no | n.a. | no | no | no | n.a. |
| Katagiri et al. | no | no | n.a. | yes | yes | no | n.a. |
| Kenny et al. | no | no | n.a. | no | yes | no | n.a. |
| Kim et al. B | no | no | n.a. | no | no | no | n.a. |
| Kunori et al. | no | no | n.a. | no | no | no | n.a. |
| Kyyriainen et al. | no | no | n.a. | yes | no | no | n.a. |
| Lee et al. C | no | no | n.a. | no | no | no | n.a. |
| Levata et al. | no | no | n.a. | no | no | no | n.a. |
| Li et al. A | no | no | n.a. | yes | no | no | n.a. |
| Li et al. D | no | no | n.a. | no | no | no | n.a. |
| Li et al. B | no | no | n.a. | no | yes | no | n.a. |
| Luo et al. | no | no | n.a. | yes | no | no | n.a. |
| Lv et al. | no | no | n.a. | no | no | no | n.a. |
| Ma et al. | no | no | n.a. | yes | yes | no | n.a. |
| Mastrella et al. | no | no | n.a. | no | no | no | n.a. |
| Mazza et al. | no | no | n.a. | no | no | no | n.a. |
| Mittal et al. | no | no | n.a. | no | no | no | n.a. |
| Mo et al. | no | no | n.a. | no | yes | no | n.a. |
| Mohammad et al. | no | no | n.a. | no | no | no | n.a. |
| Mohammadipoor-Ghasemabad et al. | no | no | n.a. | no | yes | no | n.a. |
| Mohammadpoory et al. | no | no | n.a. | no | no | no | n.a. |
| Moller et al. | no | no | n.a. | no | yes | no | n.a. |
| Murai et al. | no | no | n.a. | yes | yes | no | n.a. |
| Njoku et al. | no | no | n.a. | yes | yes | no | n.a. |
| O'Brien et al. | no | no | n.a. | no | no | no | n.a. |
| Ogun et al. | no | no | n.a. | no | yes | no | n.a. |
| Okada et al. | no | no | n.a. | no | no | no | n.a. |
| Park et al. | no | no | n.a. | no | no | no | n.a. |
| Pettibone et al. | no | no | n.a. | no | no | no | n.a. |
| Pflüger et al. | no | no | n.a. | no | no | no | n.a. |
| Qiao et al. | no | no | n.a. | no | no | no | n.a. |
| Romoli et al. | no | no | n.a. | yes | no | no | n.a. |
| Russell et al. | no | no | n.a. | no | yes | no | n.a. |
| Sa et al. | no | no | n.a. | no | no | no | n.a. |
| Sharma et al. | no | no | n.a. | no | no | no | n.a. |
| Shaver et al. | no | no | n.a. | no | yes | no | n.a. |
| Shiuchi et al. | no | no | n.a. | no | no | no | n.a. |
| Simader et al. | no | no | n.a. | no | no | no | n.a. |
| Slezia et al. | no | no | n.a. | no | no | no | n.a. |
| Souza et al. | no | no | n.a. | no | no | no | n.a. |
| Souza et al. | no | no | n.a. | no | no | no | n.a. |
| Stanchi et al. | yes | no | n.a. | no | no | no | n.a. |
| Stanojlovic et al. | no | no | n.a. | no | yes | no | n.a. |
| Sun et al. | no | no | n.a. | no | no | no | n.a. |
| Suzuki et al. | no | no | n.a. | no | yes | no | n.a. |
| Szonyi et al. | no | no | n.a. | no | no | no | n.a. |
| Szonyi et al. | no | no | n.a. | no | no | no | n.a. |
| Tomov et al. | no | no | n.a. | no | no | no | n.a. |
| Villa-Cedillo et al. | no | no | n.a. | no | no | no | n.a. |
| Villasana et al. | no | no | n.a. | yes | no | no | n.a. |
| Wang et al. F | no | no | n.a. | no | no | no | n.a. |
| Wang et al. G | no | no | n.a. | yes | yes | no | n.a. |
| Wang et al. A | no | no | n.a. | yes | yes | no | n.a. |
| Wang et al. H | no | no | n.a. | yes | yes | no | n.a. |
| Wang et al. B | no | no | n.a. | no | no | no | n.a. |
| Wang et al. D | no | no | n.a. | no | no | no | n.a. |
| Wang et al. C | no | no | n.a. | no | yes | no | n.a. |
| Wang et al. E | no | no | n.a. | no | no | no | n.a. |
| Wen et al. A | no | no | n.a. | no | no | no | n.a. |
| Wen et al. B | no | no | n.a. | no | no | no | n.a. |
| Xu et al. | no | no | n.a. | yes | yes | no | n.a. |
| Yang et al. | no | no | n.a. | yes | no | no | n.a. |
| Yeung et al. | no | no | n.a. | yes | no | no | n.a. |
| Zhang et al. C | no | no | n.a. | no | yes | no | n.a. |
| Zhang et al. B | no | no | n.a. | yes | yes | no | n.a. |
| Zhang et al. A | no | no | n.a. | no | no | no | n.a. |
| Zhao et al. | no | no | n.a. | yes | no | no | n.a. |

**Supplementary table S3: List of all included studies and evaluated parameters**

Information on all evaluated parameters in all included studies from 2009 (k= 911) and 2019 (k= 1333). N.a.= not applicable, this drug was not administered; n.r.= drug administered but no information on substance used was provided; preoperatively= all timepoints before skin incision; postoperatively= all timepoints after end of surgery; intraoperatively= all timepoints between skin incision and end of surgery

| **first author  (last name)** | **title** | **year of publication  (2009, 2019)** | **journal (in which the study was published)   (name)** | **issue  (number or n.r.)** | **pages or article number  (x - x)** | **anesthesia and other compounds (sedatives, hypnotics)  (drug OR n.r.)** | **analgesics preoperatively  (drug OR n.a.)** | **analgesics introperatively  (drug OR n.a.)** | **analgesics postoperatively  (drug OR n.a.)** |
| --- | --- | --- | --- | --- | --- | --- | --- | --- | --- |
| Aalbers | Horner's syndrome: a complication of experimental carotid artery surgery in rats | 2009 | Autonomic Neuroscience: Basic and Clinical | 147 | 64 to 69 | isoflurane | buprenorphine | n.a. | n.a. |
| AbdAlla | Angiotensin II AT2 receptor oligomers mediate G-protein dysfunction in an animal model of Alzheimer disease | 2009 | Journal of Biological Chemistry | 284 | 6554 to 6565 | n.r. | n.a. | n.a. | n.a. |
| Abdel Baki | A hierarchy of neurobehavioral tasks discriminates between mild and moderate brain injury in rats | 2009 | Brain Research | 1280 | 98 to 106 | isoflurane | n.a. | n.a. | n.a. |
| Abdipranoto-Cowley | Activin A is essential for neurogenesis following neurodegeneration | 2009 | Stem Cells | 27 | 1330 to 1346 | ketamine / xylazine | n.a. | n.a. | n.a. |
| Aberg | Peripheral administration of GH induces cell proliferation in the brain of adult hypophysectomized rats | 2009 | Journal of Endocrinology | 201 | 141 to 150 | n.r. | n.a. | n.a. | n.a. |
| Able | Localisation of melanin-concentrating hormone receptor 1 in rat brain and evidence that sleep parameters are not altered despite high central receptor occupancy | 2009 | European Journal of Pharmacology | 616 | 101 to 106 | isoflurane | n.a. | n.a. | n.a. |
| Abrahamson | Simvastatin therapy prevents brain trauma-induced increases in beta-amyloid peptide levels | 2009 | Annals of Neurology | 66 | 407 to 414 | isoflurane | n.a. | n.a. | n.a. |
| Abuhamed | Experimental epileptic discharge can be transmitted between 2 brains in rats | 2009 | Neurosciences | 14 | 128 to 130 | chloral hydrate | n.a. | n.a. | n.a. |
| Abuirmeileh | The CRF-like peptide urocortin greatly attenuates loss of extracellular striatal dopamine in rat models of Parkinson's disease by activating CRF(1) receptors | 2009 | European Journal of Pharmacology | 604 | 45 to 50 | n.r. | n.a. | n.a. | n.a. |
| Abulafia | Cerebral activity during the anesthesia-like state induced by mesopontine microinjection of pentobarbital | 2009 | The Journal of Neuroscience | 29 | 7053 to 7064 | ketamine / xylazine | n.a. | n.a. | n.a. |
| Acosta-Garcia | D4 and D1 dopamine receptors modulate [3H] GABA release in the substantia nigra pars reticulata of the rat | 2009 | Neuropharmacology | 57 | 725 to 730 | chloral hydrate | n.a. | n.a. | n.a. |
| Adams | Serotonergic lesions of the dorsal hippocampus differentially modulate locomotor hyperactivity induced by drugs of abuse in rats: implications for schizophrenia | 2009 | Psychopharmacology | 206 | 665 to 676 | isoflurane | carprofen | n.a. | n.a. |
| Adhikari | Characterisation of cortical activity in response to deep brain stimulation of ventral-lateral nucleus: modelling and experiment | 2009 | Journal of Neuroscience Methods | 183 | 77 to 85 | isoflurane | n.a. | n.a. | n.a. |
| Adriani | Increased impulsive behavior and risk proneness following lentivirus-mediated dopamine transporter over-expression in rats' nucleus accumben | 2009 | Neuroscience | 159 | 47 to 58 | n.r. | n.a. | n.a. | n.a. |
| Agnesi | Wireless Instantaneous Neurotransmitter Concentration System-based amperometric detection of dopamine, adenosine, and glutamate for intraoperative neurochemical monitoring | 2009 | Journal of Neurosurgery | 111 | 701 | urethane | n.a. | n.a. | n.a. |
| Agostini | Peripheral anti-nociceptive effect of nociceptin/orphanin FQ in inflammation and stress-induced colonic hyperalgesia in rats | 2009 | Pain | 141 | 292 to 299 | ketamine / acepromazine | n.a. | n.a. | n.a. |
| Ahn | Intratrigeminal ganglionic injection of LPA causes neuropathic pain-like behavior and demyelination in rats | 2009 | Pain | 146 | 114 to 120 | pentobarbital | n.a. | n.a. | n.r. |
| Ahn | Compression of the trigeminal ganglion produces prolonged nociceptive behavior in rats | 2009 | European Journal of Pain | 13 | 568 to 575 | pentobarbital | n.a. | n.a. | n.r. |
| Akbar | Delivery of temozolomide to the tumor bed via biodegradable gel matrices in a novel model of intracranial glioma with resection | 2009 | Journal of Neurooncology | 94 | 203 to 212 | ketamine / xylazine | n.a. | n.a. | n.a. |
| Al-Ashmouny | IBCOM (intra-brain communication) microsystem: wireless transmission of neural signals within the brain | 2009 | Annual International Conference of the IEEE Engineering in Medicine and Biology Society | n.a. | 2054 to 2057 | n.r. | n.a. | n.a. | n.a. |
| Alberti | Prolonged survival and expression of neural markers by bone marrow-derived stem cells transplanted into brain lesions | 2009 | Medical Science Monitor | 15 | 47 to 54 | chloral hydrate | n.a. | n.a. | n.a. |
| Alfaro-Rodriguez | Neuro-protective effects of carbamazepine on sleep patterns and head and body shakes in kainic acid-treated rats | 2009 | Chemico-Biological Interactions | 180 | 376 to 382 | pentobarbital | n.a. | n.a. | n.a. |
| Ambati | ICV vs. VMH injection of leptin: comparative effects on hypothalamic gene expression | 2009 | Behavioral Brain Research | 196 | 279 to 285 | ketamine / xylazine / acepromazine | n.a. | n.a. | flunixin / meglumine |
| Ampo | Induction of pancreatitis-associated protein (PAP) family members in neurons after traumatic brain injury | 2009 | Journal of Neurotrauma | 26 | 1683 to 1693 | pentobarbital | n.a. | n.a. | n.a. |
| An | Central administration of Orphanin FQ inhibits GnRH secretion by ORL1 receptor in the median eminence of freely moving ovariectomized rats | 2009 | Neuroscience Bulletin | 25 | 1 to 6 | pentobarbital | n.a. | n.a. | n.a. |
| Anaclet | Orexin/hypocretin and histamine: distinct roles in the control of wakefulness demonstrated using knock-out mouse models | 2009 | Journal of Neuroscience | 29 | 14423 to 14438 | isoflurane | n.a. | n.a. | n.a. |
| Ananda | Modulation of instantaneous synchrony during seizures by deep brain stimulation | 2009 | Annual International Conference of the IEEE Engineering in Medicine and Biology Society | n.a. | 3310 to 3313 | ketamine / xylazine | n.a. | n.a. | n.a. |
| Andersen | Electrophysiological correlates of sleep disturbance induced by acute and chronic administration of D-amphetamine | 2009 | Brain Research | 1249 | 162 to 172 | ketamine / diazepam | n.a. | n.a. | diclofenac |
| Anderson | Impaired expression of neuroprotective molecules in the HIF-1alpha pathway following traumatic brain injury in aged mice | 2009 | Journal of Neurotrauma | 26 | 1557 to 1566 | isoflurane | n.a. | n.a. | n.a. |
| Andrade | Effects of bilateral lesions in thalamic reticular nucleus and orbitofrontal cortex in a T-maze perseverative model produced by 8-OH-DPAT in rats | 2009 | behavioral Brain Research | 203 | 108 to 112 | ketamine | n.a. | n.a. | n.a. |
| Andreou | Activation of iGluR5 kainate receptors inhibits neurogenic dural vasodilatation in an animal model of trigeminovascular activation | 2009 | British Journal of Pharmacology | 157 | 464 to 473 | pentobarbital | n.a. | n.a. | n.a. |
| Andrioli | Different patterns of neuronal activation and neurodegeneration in the thalamus and cortex of epilepsy-resistant Proechimys rats versus Wistar rats after pilocarpine-induced protracted seizures | 2009 | Epilepsia | 50 | 832 to 848 | n.r. | n.a. | n.a. | n.a. |
| Antonucci | Intrahippocampal infusion of botulinum neurotoxin E (BoNT/E) reduces spontaneous recurrent seizures in a mouse model of mesial temporal lobe epilepsy | 2009 | Epilepsia | 50 | 963 to 966 | midazolam / fluanisone | fentanyl | n.a. | n.a. |
| Appelberg | The effects of a ketogenic diet on behavioral outcome after controlled cortical impact injury in the juvenile and adult rat | 2009 | Journal of Neurotrauma | 26 | 497 to 506 | isoflurane | n.a. | n.a. | n.a. |
| Arrigoni | Long-term synaptic plasticity is impaired in rats with lesions of the ventrolateral preoptic nucleus | 2009 | The European Journal of Neuroscience | 30 | 2112 to 2120 | chloral hydrate | n.a. | n.a. | n.a. |
| Atkin | Transgenic mice expressing a cameleon fluorescent Ca2+ indicator in astrocytes and Schwann cells allow study of glial cell Ca2+ signals in situ and in vivo | 2009 | Journal of Neuroscience Methods | 181 | 212 to 226 | isoflurane | carprofen | n.a. | n.a. |
| Atkins | Deficits in ERK and CREB activation in the hippocampus after traumatic brain injury | 2009 | Neuroscience Letters | 459 | 52 to 56 | halothane | n.a. | n.a. | n.a. |
| Auriat | Delayed rehabilitation lessens brain injury and improves recovery after intracerebral hemorrhage in rats | 2009 | Brain Research | 1251 | 262 to 268 | isoflurane | bupivacaine | n.a. | n.a. |
| Ayala-Guerrero | Effect of oxcarbazepine on sleep architecture | 2009 | Epilepsy & Behavior | 15 | 287 to 290 | pentobarbital | n.a. | n.a. | n.a. |
| Aydin | Comparison of power spectrum predictors in computing coherence functions for intracortical EEG signals | 2009 | Annals of Biomedical Engineering | 37 | 192 to 200 | n.r. | n.a. | n.a. | n.a. |
| Ayling | Automated light-based mapping of motor cortex by photoactivation of channelrhodopsin-2 transgenic mice | 2009 | Nature Methods | 6 | 219 to 224 | isoflurane | n.a. | n.a. | n.a. |
| Baek | Polyimide-based multi-channel arrayed electrode for measuring EEG signal on the skull of mouse | 2009 | Annual International Conference of the IEEE Engineering in Medicine and Biology Society | 2009 | 7022 to 7025 | ketamine / xylazine | n.a. | n.a. | n.a. |
| Bahraminasab | Physics of brain dynamics: Fokker-Planck analysis reveals changes in EEG delta and theta activity during anaesthesia | 2009 | New Journal of Physiology | 9 | 103051 | ketamine / xylazine | n.a. | n.a. | n.a. |
| Bai | Orexin A attenuates unconditioned sexual motivation in male rats | 2009 | Pharmacology, Biochemistry and Behavior | 91 | 581 to 589 | chloral hydrate | n.a. | n.a. | n.a. |
| Bansal | Traumatic brain injury and intestinal dysfunction: uncovering the neuro-enteric axis | 2009 | Journal of Neurotrauma | 8 | 1353 to 1359 | isoflurane | n.a. | n.a. | n.a. |
| Baraban | Reduction of seizures by transplantation of cortical GABAergic interneuron precursors into Kv1.1 mutant mice | 2009 | PNAS | 106 | 15472 to 15477 | hypothermia / ketamine /xylazine | n.a. | n.a. | n.a. |
| Baracskay | Status epilepticus affects the gigantocellular network of the pontine reticular formation | 2009 | BioMed Central Neuroscience | 10 | 133 | halothane | n.a. | n.a. | n.a. |
| Barnwell | Kv4.2 knockout mice demonstrate increased susceptibility to convulsant stimulation | 2009 | Epilepsia | 50 | 1741 to 1751 | ketamine / xylazine | n.a. | n.a. | n.a. |
| Barresi | Neuronal responses to tilt within the rat cerebellar vermis | 2009 | Brain Research Bulletin | 78 | 182 to 188 | urethane | procaine | n.a. | n.a. |
| Bartolomucci | Chronic intracerebroventricular injection of TLQP-21 prevents high fat diet induced weight gain in fast weight-gaining mice | 2009 | Genes Nutritional | 4 | 49-57 | ketamine / xylazine | n.a. | n.a. | n.a. |
| Bassi | The time course of the probability of transition into and out of REM sleep | 2009 | Sleep | 32 | 655 to 669 | pentobarbital | n.a. | n.a. | n.a. |
| Bastedo | Modulation of genioglossus muscle activity across sleep-wake states by histamine at the hypoglossal motor pool | 2009 | Sleep | 32 | 1313 to 1324 | ketamine / xylazine | buprenorphine | n.a. | n.a. |
| Batista-Brito | The cell-intrinsic requirement of Sox6 for cortical interneuron development | 2009 | Neuron | 63 | 466 to 481 | ketamine / xylazine | n.a. | n.a. | n.a. |
| Bazzu | Real-time monitoring of brain tissue oxygen using a miniaturized biotelemetric device implanted in freely moving rats | 2009 | Annals of Chemistry | 81 | 2235 to 2241 | chloral hydrate | n.a. | n.a. | n.a. |
| Behrend | Toward feedback controlled deep brain stimulation: Dynamics of glutamate release in the subthalamic nucleus in rats | 2009 | Journal of Neuroscience Methods | 180 | 278-289 | ketamine / xylazine | n.a. | n.a. | n.a. |
| Beig | Epileptic seizure-induced hypertension and its prevention by calcium channel blockers: a real-time study in conscious telemetered rats | 2009 | Canadian Journal of Physiology and Pharmacology | 87 | 572 to 580 | ketamine / xylazine | carprofen | n.a. | carprofen |
| Bell | PICK1-mediated GluR2 endocytosis contributes to cellular injury after neuronal trauma | 2009 | Cell Death and Differentiation | 16 | 1665 to 1680 | halothane | n.a. | n.a. | n.a. |
| Ben Taib | Trains of transcranial direct current stimulation antagonize motor cortex hypoexcitability induced by acute hemicerebellectomy | 2009 | Journal of Neurosurgery | 111 | 796 to 806 | chloral hydrate | n.a. | n.a. | n.a. |
| Benaliouad | Effects of the dopamine stabilizer, OSU-6162, on brain stimulation reward and on quinpirole-induced changes in reward and locomotion | 2009 | European Neuropsychopharmacology | 19 | 416 to 430 | isoflurane | n.a. | n.a. | n.a. |
| Bercovici | Monoamine variability in the chronic model of atypical absence seizures | 2009 | Epilepsia | 50 | 768 to 775 | pentobarbital | n.a. | n.a. | n.a. |
| Berdiev | Cholinergic stimulation of the nucleus basalis of Meynert and reticular thalamic nucleus affects spike-and-wave discharges in WAG/Rij rats | 2009 | Neuroscience Letters | 463 | 249 to 253 | isoflurane | n.a. | n.a. | n.a. |
| Berretta | A rodent model of schizophrenia derived from postmortem studies | 2009 | Behavioral Brain Research | 204 | 363 to 368 | n.r. | n.a. | n.a. | n.a. |
| Bible | Attachment of stem cells to scaffold particles for intra-cerebral transplantation | 2009 | Nature protocols | 4 | 1440 to 1453 | n.r. | n.a. | n.a. | n.a. |
| Bicher | Hyperthermia-induced morphological changes in cerebral tissue of the rat | 2009 | Georgian Medical News | n.a. | 72 to 75 | chloral hydrate | n.a. | n.a. | n.a. |
| Bickerdike | NNZ-2566: a Gly-Pro-Glu analogue with neuroprotective efficacy in a rat model of acute focal stroke | 2009 | Journal of Neurological Sciences | 278 | 85 to 90 | halothane | n.a. | n.a. | n.a. |
| Biella | Probing for local activity-related modulation of the infrared backscattering of the brain cortex | 2009 | Journal of Biophotonics | 2 | 588-595 | isoflurane / pentobarbital | n.a. | n.a. | n.a. |
| Bigford | A novel protein complex in membrane rafts linking the NR2B glutamate receptor and autophagy is disrupted following traumatic brain injury | 2009 | Journal of Neurotrauma | 26 | 703 to 720 | halothane | n.a. | n.a. | n.a. |
| Biondolillo | Blocking glutamate receptors in the waist area of the parabrachial nucleus decreases taste reactivity behaviors in conscious rats | 2009 | Chemical Senses | 34 | 221 to 230 | pentobarbital | n.a. | n.a. | n.a. |
| Black | Role of polysialylated neural cell adhesion molecule in rapid eye movement sleep regulation in rats | 2009 | European Journal of Neuroscience | 30 | 2190 to 2204 | ketamine / xylazine / acepromazine | n.a. | n.a. | n.a. |
| Bledsoe | Development of the Wireless Instantaneous Neurotransmitter Concentration System for intraoperative neurochemical monitoring using fast-scan cyclic voltammetry | 2009 | Journal of Neurosurgery | 111 | 712 to 723 | urethane | n.a. | n.a. | n.a. |
| Blevins | Forebrain melanocortin signaling enhances the hindbrain satiety response to CCK-8 | 2009 | American Journal of Physiology, regulatory, integrative and comparative physiology | 296 | 476 to 484 | ketamine / xylazine / acepromazine | n.a. | n.a. | n.a. |
| Blomquist | Estimation of thalamocortical and intracortical network models from joint thalamic single-electrode and cortical laminar-electrode recordings in the rat barrel system | 2009 | PLoS Computational Biology | 5 | 1000328 | chloralose / halothane | lidocaine | n.a. | n.a. |
| Blumenfeld | Role of hippocampal sodium channel Nav1.6 in kindling epileptogenesis | 2009 | Epilepsia | 50 | 44 to 55 | ketamine / xylazine / acepromazine | n.a. | n.a. | n.a. |
| Bobeck | Drug dependent sex-differences in periaqueducatal gray mediated antinociception in the rat | 2009 | Pain | 47 | 210 to 216 | pentobarbital | n.a. | n.a. | n.a. |
| Bocian | The effect of carbenoxolone on hippocampal formation theta rhythm in rats: in vitro and in vivo approaches | 2009 | Brain Research Bulletin | 78 | 290 to 298 | halothane / urethane | n.a. | n.a. | n.a. |
| Boison | Engineered adenosine-releasing cells for epilepsy therapy: human mesenchymal stem cells and human embryonic stem cells | 2009 | Neurotherapeutics | 2 | 278 to 283 | n.r. | n.a. | n.a. | n.a. |
| Boni | The in vivo effect of VIP, PACAP-38 and PACAP-27 and mRNA expression of their receptors in rat middle meningeal artery | 2009 | Cephalalgia | 29 | 837-847 | pentobarbital | n.a. | n.a. | n.a. |
| Bonilla | Delayed intralesional transplantation of bone marrow stromal cells increases endogenous neurogenesis and promotes functional recovery after severe traumatic brain injury | 2009 | Brain Injury | 23 | 760 to 769 | sevoflurane | n.a. | n.a. | n.a. |
| Borna | A small, light-weight, low-power, multichannel wireless neural recording microsystem | 2009 | Annual International Conference of the IEEE Engineering in Medicine and Biology Society | 2009 | 5413 to 5416 | ketamine / xylazine / acepromazine | n.a. | n.a. | n.a. |
| Borowicz | 2-Methyl-6-phenylethynyl-pyridine (MPEP), a non-competitive mGluR5 antagonist, differentially affects the anticonvulsant activity of four conventional antiepileptic drugs against amygdala-kindled seizures in rats | 2009 | Pharmacological Report | 61 | 621 to 630 | pentobarbital | n.a. | n.a. | n.a. |
| Boucetta | Activity profiles of cholinergic and intermingled GABAergic and putative glutamatergic neurons in the pontomesencephalic tegmentum of urethane-anesthetized rats | 2009 | Journal of Neuroscience | 29 | 4664 to 4674 | urethane | n.a. | n.a. | n.a. |
| Bouilleret | Progressive brain changes on serial manganese-enhanced MRI following traumatic brain injury in the rat | 2009 | Journal of Neurotrauma | 26 | 1999 to 2013 | isoflurane | n.a. | n.a. | n.a. |
| Bragin | The cause of the imbalance in the neuronal network leading to seizure activity can be predicted by the electrographic pattern of the seizure onset | 2009 | Journal of Neuroscience | 29 | 3660 to 3671 | isoflurane | n.a. | n.a. | n.a. |
| Bramlett | Sex differences in XIAP cleavage after traumatic brain injury in the rat | 2009 | Neuroscience Letters | 461 | 49-53 | halothane | n.a. | n.a. | n.a. |
| Brown | Activity of neurochemically heterogeneous dopaminergic neurons in the substantia nigra during spontaneous and driven changes in brain state | 2009 | Journal of Neuroscience | 29 | 2915 to 2925 | halothane | n.a. | n.a. | n.a. |
| Bryant | A technique for stereotaxic recordings of neuronal activity in awake, head-restrained mice | 2009 | Journal of Neuroscientific Methods | 178 | 75 to 79 | tribromoethanol | n.a. | n.a. | n.a. |
| Budzinska | Serotoninergic modulation of cortical and respiratory responses to episodic hypoxia | 2009 | European Journal of Medical Research | 14 | 32 to 37 | urethane | n.a. | n.a. | n.a. |
| Bukhatwa | An immunohistochemical and stereological analysis of PSI-induced nigral neuronal degeneration in the rat | 2009 | Journal of Neurochemistry | 109 | 52 to 59 | isoflurane | n.a. | n.a. | n.a. |
| Buki | Clinical and model research of neurotrauma | 2009 | Methods in Molecular Biology | 566 | 41 to 55 | isoflurane | n.a. | n.a. | n.a. |
| Byun | Kainic Acid-induced Neuronal Death is Attenuated by Aminoguanidine but Aggravated by L-NAME in Mouse Hippocampus | 2009 | Korean Journal of physiology & pharmacology | 13 | 265-271 | ether | n.a. | n.a. | n.a. |
| Cabrera | Lactation is a natural model of hippocampus neuroprotection against excitotoxicity | 2009 | neuroscience letters | 461 | 136 to 139 | xylazine | n.a. | n.a. | n.a. |
| Caltana | Neuronal and glial alterations due to focal cortical hypoxia induced by direct cobalt chloride (CoCl2) brain injection | 2009 | Neurotoxicity Research | 15 | 348-358 | sevoflurane | n.a. | n.a. | n.a. |
| Campos | Evidence for a potential role for TRPV1 receptors in the dorsolateral periaqueductal gray in the attenuation of the anxiolytic effects of cannabinoids | 2009 | progress in neuro-psychopharmacology & biological psychiatry | 33 | 1517 to 1521 | tribromoethanol | n.a. | n.a. | n.a. |
| Cannella | Persistent increase of alcohol-seeking evoked by neuropeptide S: an effect mediated by the hypothalamic hypocretin system | 2009 | neuropsychopharmacology | 34 | 2125 to 2134 | tiletamine / zolazepam | n.a. | n.a. | n.a. |
| Cao | Endothelin rather than 20-HETE contributes to loss of pial arteriolar dilation during focal cerebral ischemia with and without polymeric hemoglobin transfusion | 2009 | American Journal of Physiology, regulatory, integrative and comparative physiology | 296 | 1412 to 1418 | isoflurane | n.a. | n.a. | n.a. |
| Capone | Estrous cycle-dependent neurovascular dysfunction induced by angiotensin II in the mouse neocortex | 2009 | Hypertension | 54 | 302 to 307 | isoflurane / urethane / chloralose | n.a. | n.a. | n.a. |
| Capozzo | Low frequency stimulation of the pedunculopontine nucleus modulates electrical activity of subthalamic neurons in the rat | 2009 | Journal of Neural Transmission | 116 | 51 to 56 | chloral hydrate | n.a. | n.a. | n.a. |
| Carcak | Effect of stage 2 kindling on local cerebral blood flow rates in rats with genetic absence epilepsy | 2009 | Epilepsia | 50 | 33-43 | ketamine / xylazine | n.a. | n.a. | n.a. |
| Cemil | The effect of mitomycin C as fibrosis preventive agent during craniectomies | 2009 | British Journal of Neurosurgery | 23 | 304-308 | n.r. | n.a. | n.a. | n.a. |
| Cendelin | A preliminary study of solid embryonic cerebellar graft survival in adult B6CBA Lurcher mutant and wild type mice | 2009 | the anatomical record | 292 | 1986 to 1992 | ketamine / xylazine | n.a. | n.a. | n.a. |
| Cenier | Respiration-gated formation of gamma and beta neural assemblies in the mammalian olfactory bulb | 2009 | European Journal of Neuroscience | 29 | 921 to 930 | urethane | n.a. | n.a. | n.a. |
| Chang | Microthalamotomy effect during deep brain stimulation: potential involvement of adenosine and glutamate efflux | 2009 | Annual International Conference of the IEEE Engineering in Medicine and Biology Society | 2009 | 3294 to 3297 | urethane | n.a. | n.a. | n.a. |
| Chapon | Imaging E-selectin expression following traumatic brain injury in the rat using a targeted USPIO contrast agent | 2009 | MAGMA | 22 | 167 to 174 | isoflurane | n.a. | n.a. | n.a. |
| Charrueau | Metabolic response and nutritional support in traumatic brain injury: evidence for resistance to renutrition | 2009 | Journal of Neurotrauma | 26 | 1 to 10 | chloral hydrate | n.a. | n.a. | n.a. |
| Chauviere | Early deficits in spatial memory and theta rhythm in experimental temporal lobe epilepsy | 2009 | Neurobiology of Disease | 29 | 5402 to 5410 | ketamine / xylazine | n.a. | n.a. | n.a. |
| Chavez | The basolateral amygdala modulates specific sensory memory representations in the cerebral cortex | 2009 | American Journal of Physiology, regulatory, integrative and comparative physiology | 296 | 1412 to 1418 | urethane | n.a. | n.a. | n.a. |
| Chen | GluR6-containing KA receptor mediates the activation of p38 MAP kinase in rat hippocampal CA1 region during brain ischemia injury | 2009 | Hippocampus | 19 | 79-89 | n.r. | n.a. | n.a. | n.a. |
| Chen | Simvastatin reduces secondary brain injury caused by cortical contusion in rats: possible involvement of TLR4/NF-kappaB pathway | 2009 | Experimental Neurology | 216 | 398 to 406 | urethane | n.a. | n.a. | n.a. |
| Chen | Premarin stimulates estrogen receptor-alpha to protect against traumatic brain injury in male rats | 2009 | Critical Care Medicine | 37 | 3097 to 3106 | ketamine / xylazine / pentobarbital | n.a. | n.a. | n.a. |
| Chen | The effects of topiramate on caspase-3 expression in hippocampus of basolateral amygdala (BLA) electrical kindled epilepsy rat | 2009 | journal of molecular neuroscience | 38 | 201 to 206 | chloral hydrate | n.a. | n.a. | n.a. |
| Chen | Glucocorticoids aggravate retrograde memory deficiency associated with traumatic brain injury in rats | 2009 | Journal of Neurotrauma | 26 | 253 to 260 | chloral hydrate | n.a. | n.a. | n.a. |
| Chen | Design and fabrication of a polyimide-based microelectrode array: application in neural recording and repeatable electrolytic lesion in rat brain | 2009 | Journal of Neuroscience Methods | 182 | 6 to 16 | pentobarbital | n.a. | n.a. | n.a. |
| Chen | Human amnion-derived multipotent progenitor cell treatment alleviates traumatic brain injury-induced axonal degeneration | 2009 | Journal of Neurotrauma | 26 | 1987 to 1997 | isoflurane | n.a. | n.a. | n.a. |
| Cheng | Cobratoxin inhibits pain-evoked discharge of neurons in thalamic parafascicular nucleus in rats: involvement of cholinergic and serotonergic systems | 2009 | toxicon | 54 | 224 to 232 | chloral hydrate / gallamine triethiodide | n.a. | n.a. | n.a. |
| Cheong | Deletion of phospholipase C beta4 in thalamocortical relay nucleus leads to absence seizures | 2009 | PNAS | 106 | 21912 to 21917 | tribromoethanol | n.a. | n.a. | n.a. |
| Chernyy | Time dependence of stimulation/recording-artifact transfer function estimates for neural interface systems | 2009 | Annual International Conference of the IEEE Engineering in Medicine and Biology Society | 2009 | 1380 to 1383 | n.r. | n.a. | n.a. | n.a. |
| Chikahisa | Central AMPK contributes to sleep homeostasis in mice | 2009 | Neuropharmacology | 57 | 369 to 374 | ketamine / xylazine | n.a. | n.a. | n.a. |
| Christoforidis | High resolution ultra high field magnetic resonance imaging of glioma microvascularity and hypoxia using ultra-small particles of iron oxide | 2009 | investigative radiology | 44 | 375 to 383 | ketamine / xylazine | n.a. | n.a. | n.a. |
| Chuang | Contribution of nitric oxide, superoxide anion, and peroxynitrite to activation of mitochondrial apoptotic signaling in hippocampal CA3 subfield following experimental temporal lobe status epilepticus | 2009 | Epilepsia | 50 | 731 to 746 | chloral hydrate | n.a. | n.a. | n.a. |
| Chuang | Preservation of mitochondrial integrity and energy metabolism during experimental status epilepticus leads to neuronal apoptotic cell death in the hippocampus of the rat | 2009 | Seizure | 18 | 420 to 428 | chloral hydrate | n.a. | n.a. | n.a. |
| Chung | Metallothionein treatment attenuates microglial activation and expression of neurotoxic quinolinic acid following traumatic brain injury | 2009 | Neurotoxicity Research | 15 | 381 to 389 | pentobarbital | n.a. | n.a. | n.a. |
| Cifani | Possible common central pathway for resistin and insulin in regulating food intake | 2009 | Acta Physiologica | 196 | 395-400 | tiletamine / zolazepam | n.a. | n.a. | n.a. |
| Cirelli | Proteomic profiling of the rat cerebral cortex in sleep and waking | 2009 | Archives Italiennes de Biologie | 147 | 59 to 68 | n.r. | n.a. | n.a. | n.a. |
| Clark | Treatments (12 and 48 h) with systemic and brain-selective hypothermia techniques after permanent focal cerebral ischemia in rat | 2009 | Experimental Neurology | 220 | 391 to 399 | isoflurane | bupivacaine | n.a. | n.a. |
| Clark | Feeding-elicited cataplexy in orexin knockout mice | 2009 | neuroscience | 161 | 970 to 977 | ketamine / xylazine | n.a. | n.a. | n.a. |
| Clausen | Neutralization of interleukin-1beta modifies the inflammatory response and improves histological and cognitive outcome following traumatic brain injury in mice | 2009 | European Journal of Neuroscience | 30 | 385 to 396 | isoflurane | bupivacaine | n.a. | n.a. |
| Colgin | Frequency of gamma oscillations routes flow of information in the hippocampus | 2009 | Nature | 462 | 353 to 358 | pentobarbital / chloral hydrate | n.a. | n.a. | n.a. |
| Comi | Impact of age and strain on ischemic brain injury and seizures after carotid ligation in immature mice | 2009 | international journal of developmental neuroscience | 27 | 271 to 277 | isoflurane | n.a. | n.a. | n.a. |
| Console | Effect of insulin-like growth factor-I gene therapy on the somatotropic axis in experimental prolactinomas | 2009 | Cells Tissues Organs | 190 | 20 to 26 | ketamine / xylazine | n.a. | n.a. | n.a. |
| Conte | Multiple neuroanatomical tract-tracing using fluorescent Alexa Fluor conjugates of cholera toxin subunit B in rats | 2009 | Nature protocols | 4 | 1157 to 1166 | ketamine / xylazine / isoflurane | n.a. | n.a. | n.a. |
| Cooke | Peripheral and central administration of xenin and neurotensin suppress food intake in rodents | 2009 | obesity | 17 | 1135 to 1143 | ketamine / xylazine | n.a. | n.a. | n.a. |
| Coon | Brain-penetrating 2-aminobenzimidazole H(1)-antihistamines for the treatment of insomnia | 2009 | Bioorganic & Medicinal Chemistry Letters | 19 | 4380 to 4384 | n.r. | n.a. | n.a. | n.a. |
| Cordeiro | Improvement in hippocampal kindling analysis through computational processing data | 2009 | Arquivos de neuro-psiquiatria | 67 | 677 to 683 | ketamine / xylazine | n.a. | n.a. | buprenorphine |
| Cortez | Infantile spasms and Down syndrome: a new animal mode | 2009 | pediatric research | 65 | 499 to 503 | pentobarbital | n.a. | n.a. | buprenorphine |
| Cota | Distinct patterns of electrical stimulation of the basolateral amygdala influence pentylenetetrazole seizure outcome | 2009 | Epilepsy & Behavior | 14 | 26 to 31 | thiopental | lidocaine | n.a. | flunixin |
| Covel | Design and evaluation of novel biphenyl sulfonamide derivatives with potent histamine H(3) receptor inverse agonist activity | 2009 | journal of medical chemistry | 52 | 5603 to 5611 | ketamine / xylazine | n.a. | n.a. | n.a. |
| Covey | Using fast-scan cyclic voltammetry to evaluate striatal dopamine release elicited by subthalamic nucleus stimulation | 2009 | Annual International Conference of the IEEE Engineering in Medicine and Biology Society | 2009 | 3306 to 3309 | urethane | n.a. | n.a. | n.a. |
| Crawford | Apolipoprotein E-genotype dependent hippocampal and cortical responses to traumatic brain injury | 2009 | neuroscience | 159 | 1349 to 1365 | isoflurane | n.a. | n.a. | n.a. |
| Criado | Event-related oscillations as risk markers in genetic mouse models of high alcohol preference | 2009 | neuroscience | 163 | 506 to 523 | halothane | n.a. | n.a. | n.a. |
| Cui | The effect of baclofen on alterations in the sleep patterns induced by different stressors in rats | 2009 | Journal of Pharmacological Sciences | 109 | 518 to 524 | pentobarbital | n.a. | n.a. | n.a. |
| Cunningham | Microglia and the urokinase plasminogen activator receptor/uPA system in innate brain inflammation | 2009 | Glia | 57 | 1802-1814 | n.r. | n.a. | n.a. | n.a. |
| Cunningham | Amygdalar GABAergic-rich neural grafts attenuate anxiety-like behavior in rats | 2009 | Behavioral Brain Research | 205 | 146 to 153 | ketamine / xylazine | n.a. | n.a. | n.a. |
| Dai | Quantitative detection of the expression of mitochondrial cytochrome c oxidase subunits mRNA in the cerebral cortex after experimental traumatic brain injury | 2009 | brain research | 1251 | 287 to 295 | pentobarbital | n.a. | n.a. | n.a. |
| D'Alimonte | Altered distribution and function of A2A adenosine receptors in the brain of WAG/Rij rats with genetic absence epilepsy, before and after appearance of the disease | 2009 | European journal of neuroscience | 30 | 1023 to 1035 | chloral hydrate | n.a. | n.a. | n.a. |
| D'Ambrosio | Functional definition of seizure provides new insight into post-traumatic epileptogenesis | 2009 | brain | 132 | 2805 to 2821 | halothane | n.a. | n.a. | n.a. |
| Dash | Long-term homeostasis of extracellular glutamate in the rat cerebral cortex across sleep and waking states | 2009 | The Journal of Neuroscience | 29 | 620 to 629 | isoflurane | n.a. | n.a. | n.a. |
| Dash | Histone deactylase inhibition combined with behavioral therapy enhances learning and memory following traumatic brain injury | 2009 | neuroscience | 163 | 1 to 8 | isoflurane | n.a. | n.a. | n.a. |
| Dash | Sulforaphane improves cognitive function administered following traumatic brain injury | 2009 | neuroscience letters | 460 | 103 to 107 | isoflurane | n.a. | n.a. | n.a. |
| Datta | Identification of cholinergic and non-cholinergic neurons in the pons expressing phosphorylated cyclic adenosine monophosphate response element-binding protein as a function of rapid eye movement sleep | 2009 | Neuroscience | 163 | 397-414 | pentobarbital | n.a. | n.a. | n.a. |
| de Araujo Furtado | Analyzing large data sets acquired through telemetry from rats exposed to organophosphorous compounds: an EEG study | 2009 | Journal of Neuroscience Methods | 184 | 176 to 183 | n.r. | n.a. | n.a. | n.a. |
| de Faria | Antidipsogenic effects of central adenosine-5'-triphosphate | 2009 | Brazilian Journal of Medical and Biological Research | 42 | 105 to 113 | ketamine / xylazine | n.a. | n.a. | ketoprofen |
| de Lima | L-glutamine supplementation during the lactation period facilitates cortical spreading depression in well-nourished and early-malnourished rats | 2009 | Life Sciences | 85 | 241 to 247 | urethane / chloralose | n.a. | n.a. | n.a. |
| de Menezes | Sympathetic cutaneous vasomotor alerting responses (SCVARs) are associated with hippocampal theta rhythm in non-moving conscious rats | 2009 | brain research | 1298 | 123 to 130 | isoflurane | n.a. | n.a. | carprofen |
| de Oliveira | Anti-proliferative effect of the gastrin-release peptide receptor antagonist RC-3095 plus temozolomide in experimental glioblastoma models | 2009 | Journal of Neurooncology | 93 | 191 to 201 | pentobarbital | n.a. | n.a. | n.a. |
| de Rivero Vaccari | Therapeutic neutralization of the NLRP1 inflammasome reduces the innate immune response and improves histopathology after traumatic brain injury | 2009 | Journal of Cerebral Blood Flow & Metabolism | 29 | 1251 to 1261 | halothane | n.a. | n.a. | n.a. |
| Deboer | Sleep and sleep homeostasis in constant darkness in the rat | 2009 | Journal of Sleep Research | 18 | 357 to 364 | n.r. | n.a. | n.a. | n.a. |
| Degos | Chronic but not acute dopaminergic transmission interruption promotes a progressive increase in cortical beta frequency synchronization: relationships to vigilance state and akinesia | 2009 | Cerebral Cortex | 19 | 1616 to 1630 | pentobarbital / ketamine | n.a. | n.a. | n.a. |
| Dejean | Cortical effects of subthalamic stimulation correlate with behavioral recovery from dopamine antagonist induced akinesia | 2009 | Cerebral Cortex | 19 | 1055 to 1063 | ketamine / medetomidine | n.a. | n.a. | n.a. |
| Dekel | Effects of anesthesia on brain mitochondrial function, blood flow, ionic and electrical activity monitored in vivo | 2009 | Advances in experimental medicine and biology | n.r. | 49 to 54 | pentobarbital / chloral hydrate | n.a. | n.a. | n.a. |
| Del Campo | Seizure-like activity in the hypoglycemic rat: lack of correlation with the electroencephalogram of free-moving animals | 2009 | Epilepsy Research | 83 | 243 to 248 | ketamine / xylazine | n.a. | n.a. | n.a. |
| Demchenko | Phosphodiesterase-5 inhibitors oppose hyperoxic vasoconstriction and accelerate seizure development in rats exposed to hyperbaric oxygen | 2009 | journal of applied physiology | 106 | 1234 to 1242 | urethane | n.a. | n.a. | n.a. |
| Dennis | Hemorrhagic shock after experimental traumatic brain injury in mice: effect on neuronal death | 2009 | Journal of Neurotrauma | 26 | 889 to 899 | isoflurane | n.a. | n.a. | n.a. |
| Dentico | c-Fos expression in preoptic nuclei as a marker of sleep rebound in the rat | 2009 | European journal of neuroscience | 30 | 651 to 661 | ketamine / diazepam | n.a. | n.a. | n.a. |
| Descamps | Influence of the novel antidepressant and melatonin agonist/serotonin2C receptor antagonist, agomelatine, on the rat sleep-wake cycle architecture | 2009 | Psychopharmacology | 205 | 93 to 106 | chloral hydrate | n.a. | n.a. | n.a. |
| Destot-Wong | The AMPA receptor positive allosteric modulator, S18986, is neuroprotective against neonatal excitotoxic and inflammatory brain damage through BDNF synthesis | 2009 | Neuropharmacology | 57 | 277 to 286 | isoflurane | n.a. | n.a. | n.a. |
| Deurveilher | Estradiol and progesterone modulate spontaneous sleep patterns and recovery from sleep deprivation in ovariectomized rats | 2009 | Sleep | 31 | 865 to 878 | ketamine / xylazine / acepromazine | n.a. | n.a. | ketoprofen |
| Devonshire | Design and evaluation of a low-cost respiratory monitoring device for use with anaesthetized animals | 2009 | Laboratory Animals | 43 | 382 to 389 | isoflurane | n.a. | n.a. | n.a. |
| Dhillo | The thyroid hormone derivative 3-iodothyronamine increases food intake in rodents | 2009 | Diabetes, Obesity and Metabolism | 11 | 251 to 260 | ketamine / xylazine | n.a. | n.a. | n.a. |
| di Tomaso | PDGF-C induces maturation of blood vessels in a model of glioblastoma and attenuates the response to anti-VEGF treatment | 2009 | plos one | 4 | 5123 | n.r. | n.a. | n.a. | n.a. |
| Dias | Antagonism of orexin receptor-1 in the retrotrapezoid nucleus inhibits the ventilatory response to hypercapnia predominantly in wakefulness | 2009 | Journal of Physiology | 587 | 2059 to 2067 | ketamine / xylazine | n.a. | n.a. | n.a. |
| Diesch | Electroencephalographic responses to tail clamping in anaesthetized rat pups | 2009 | Laboratory Animals | 43 | 224-231 | halothane | n.a. | n.a. | n.a. |
| Diguet | Normal aging modulates the neurotoxicity of mutant huntingtin | 2009 | PLoS One | 4 | 4637 | ketamine / xylazine | n.a. | n.a. | n.a. |
| Dimpfel | Rat electropharmacograms of the flavonoids rutin and quercetin in comparison to those of moclobemide and clinically used reference drugs suggest antidepressive and/or neuroprotective action | 2009 | Phytomedicine | 16 | 287 to 294 | n.r. | n.a. | n.a. | n.a. |
| Ding | Sensitization of ventral tegmental area dopamine neurons to the stimulating effects of ethanol | 2009 | Alcoholism, clinical and experimental research | 33 | 1571-1581 | isoflurane | n.a. | n.a. | n.a. |
| Ding | Involvement of local serotonin-2A but not serotonin-1B receptors in the reinforcing effects of ethanol within the posterior ventral tegmental area of female Wistar rats | 2009 | Psychopharmacology | 204 | 381 to 390 | halothane | n.a. | n.a. | n.a. |
| Divani | Augmenting regional cerebral blood flow using external-to-internal carotid artery flow diversion method | 2009 | Annals of Biomedical Engineering | 37 | 2428 to 2435 | pentobarbital | n.a. | n.a. | n.a. |
| do Amaral | Drug/nutrition interaction in the developing brain: dipyrone enhances spreading depression in rats | 2009 | Experimental Neurology | 219 | 492 to 498 | urethane / chloralose | n.a. | n.a. | n.a. |
| Doan | Simultaneous two-voxel localized (1)H-observed (13)C-edited spectroscopy for in vivo MRS on rat brain at 9.4T: Application to the investigation of excitotoxic lesions | 2009 | Journal of Magnetic Resonance | 198 | 94-104 | isoflurane | n.a. | n.a. | n.a. |
| Doi | Perfusion fluids used in neurosurgery affect cerebrospinal fluid and surrounding brain parenchyma in the rat ventriculocisternal perfusion model | 2009 | the journal of toxological sciences | 34 | 511 to 518 | pentobarbital | n.a. | n.a. | n.a. |
| Doll | Pharyngeal selective brain cooling improves neurofunctional and neurocognitive outcome after fluid percussion brain injury in rats | 2009 | Journal of Neurotrauma | 26 | 1 to 8 | ketamine / midazolam | n.a. | n.a. | n.a. |
| Dong | Activation of orexin signal in basal forebrain facilitates the emergence from sevoflurane anesthesia in rat | 2009 | Neuropeptides | 43 | 179 to 185 | pentobarbital | n.a. | n.a. | n.a. |
| Donner | Estrogen receptor beta regulates the expression of tryptophan-hydroxylase 2 mRNA within serotonergic neurons of the rat dorsal raphe nuclei | 2009 | neuroscience | 163 | 705 to 718 | ketamine / xylazine / acepromazine | n.a. | n.a. | n.a. |
| Doretto | Role of the superior colliculus in the expression of acute and kindled audiogenic seizures in Wistar audiogenic rats | 2009 | Epilepsia | 50 | 2563-2574 | tribromoethanol | n.a. | n.a. | n.a. |
| Dortch | Evidence of multiexponential T2 in rat glioblastoma | 2009 | NMR in Biomedicine | 22 | 609 to 618 | isoflurane | n.a. | n.a. | n.a. |
| Dou | Macrophage delivery of nanoformulated antiretroviral drug to the brain in a murine model of neuroAIDS | 2009 | the journal of immunology | 183 | 661 to 669 | n.r. | n.a. | n.a. | n.a. |
| Du | In vivo proton MRS to quantify anesthetic effects of pentobarbital on cerebral metabolism and brain activity in rat | 2009 | magnetic resonances in medicine | 62 | 1285 to 1393 | isoflurane / pentobarbital | n.a. | n.a. | n.a. |
| Du | Curcumin inhibits amygdaloid kindled seizures in rat | 2009 | Chinese Medical Journal | 122 | 1435 to 1438 | chloral hydrate | n.a. | n.a. | n.a. |
| Duan | Nucleus of solitary tract mediates cardiac sympathetic afferent reflex in rats | 2009 | European journal of physiology | 459 | 1 to 9 | urethane / chloralose | n.a. | n.a. | n.a. |
| Duan | Thiopental exaggerates ischemic brain damage and neurological deficits after experimental stroke in spontaneously hypertensive rats | 2009 | brain research | 1294 | 176 to 182 | thiopental / chloral hydrate / pentobarbital | n.a. | n.a. | n.a. |
| Dumas | Bone grafts cultured with bone marrow stromal cells for the repair of critical bone defects: an experimental study in mice | 2009 | Journal of Biomedical Materials Research | 90 | 1218 to 1229 | ketamine / xylazine | n.a. | n.a. | n.a. |
| Dunn | Functional brain mapping at 9.4T using a new MRI-compatible electrode chronically implanted in rats | 2009 | magnetic resonances in medicine | 61 | 222 to 228 | ketamine / xylazine | lidocaine | n.a. | n.a. |
| Dux | Involvement of capsaicin-sensitive afferent nerves in the proteinase-activated receptor 2-mediated vasodilatation in the rat dura mater | 2009 | Neuroscience | 161 | 887 to 894 | ketamine / xylazine / acepromazine | n.a. | n.a. | n.a. |
| Echegoyen | Single application of a CB1 receptor antagonist rapidly following head injury prevents long-term hyperexcitability in a rat model | 2009 | Epilepsy Research | 85 | 123 to 127 | tribromoethanol | n.a. | n.a. | n.a. |
| Eguibar | Serotonergic-postsynaptic receptors modulate gripping-induced immobility episodes in male taiep rats | 2009 | Synapse | 63 | 737 to 744 | chloral hydrate | n.a. | n.a. | n.a. |
| Ehlers | Event-related oscillations in mice: effects of stimulus characteristics | 2009 | Journal of Neuroscience Methods | 181 | 52 to 57 | halothane | n.a. | n.a. | n.a. |
| Ehrlichman | N-methyl-d-aspartic acid receptor antagonist-induced frequency oscillations in mice recreate pattern of electrophysiological deficits in schizophrenia | 2009 | Neuroscience | 158 | 705to 712 | isoflurane | n.a. | n.a. | n.a. |
| Eikermann | Pentobarbital dose-dependently increases respiratory genioglossus muscle activity while impairing diaphragmatic function in anesthetized rats | 2009 | anesthesiology | 110 | 327 to 334 | chloral hydrate | n.a. | n.a. | n.a. |
| El Boustani | Network-state modulation of power-law frequency-scaling in visual cortical neurons | 2009 | PLoS Computational Biology | 5 | 1000519 | pentobarbital | n.a. | n.a. | n.a. |
| Elkiweri | Competitive substrates for P-glycoprotein and organic anion protein transporters differentially reduce blood organ transport of fentanyl and loperamide: pharmacokinetics and pharmacodynamics in Sprague-Dawley rats | 2009 | anesthesia and analgesia | 108 | 149 to 159 | ketamine / xylazine | n.a. | n.a. | n.a. |
| Ellens | Development of spike-wave seizures in C3H/HeJ mice | 2009 | Epilepsy Research | 85 | 53 to 59 | ketamine / xylazine / acepromazine | n.a. | n.a. | n.a. |
| Elliott | Hypertonic saline attenuates tissue loss and astrocyte hypertrophy in a model of traumatic brain injury | 2009 | brain research | 1305 | 183 to 191 | ketamine / xylazine | n.a. | n.a. | n.a. |
| Elsas | Hippocampal zinc infusion delays the development of afterdischarges and seizures in a kindling model of epilepsy | 2009 | Epilepsia | 50 | 870 to 879 | ketamine / xylazine / pentobarbital | n.a. | n.a. | n.a. |
| Engelhorn | In vivo micro-CT imaging of rat brain glioma: a comparison with 3T MRI and histology | 2009 | neuroscience letters | 458 | 28 to 31 | ketamine / xylazine | n.a. | n.a. | n.a. |
| Englot | Cortical deactivation induced by subcortical network dysfunction in limbic seizures | 2009 | The Journal of Neuroscience | 29 | 13006 to 13018 | ketamine / xylazine | n.a. | n.a. | n.a. |
| Estrada-Sanchez | Glutamate toxicity in the striatum of the R6/2 Huntington's disease transgenic mice is age-dependent and correlates with decreased levels of glutamate transporters | 2009 | Neurobiology of Disease | 34 | 78 to 86 | halothane | n.a. | n.a. | n.a. |
| Etholm | Seizure elements and seizure element transitions during tonic-clonic seizure activity in the synapsin I/II double knockout mouse: a neuroethological description | 2009 | Epilepsy & Behavior | 14 | 582 to 590 | n.r. | n.a. | n.a. | n.a. |
| Evanson | GluR5-mediated glutamate signaling regulates hypothalamo-pituitary-adrenocortical stress responses at the paraventricular nucleus and median eminence | 2009 | Psychoneuroendocrinology | 34 | 1370 to 1379 | ketamine / xylazine | butorphanol | n.a. | n.a. |
| Exo | Resuscitation of traumatic brain injury and hemorrhagic shock with polynitroxylated albumin, hextend, hypertonic saline, and lactated Ringer's: Effects on acute hemodynamics, survival, and neuronal death in mice | 2009 | Journal of Neurotrauma | 26 | 2403 to 2408 | isoflurane | n.a. | n.a. | n.a. |
| Ezhilarasan | The hemopexin domain of MMP-9 inhibits angiogenesis and retards the growth of intracranial glioblastoma xenograft in nude mice | 2009 | International Journal of Cancer | 124 | 306 to 315 | n.r. | n.a. | n.a. | n.a. |
| Falkenstein | Pattern of long-term sensorimotor recovery following intrastriatal and--accumbens DA micrografts in a rat model of Parkinson's disease | 2009 | Journal of Neurophysiology | 122 | 2621 to 2629 | n.r. | n.a. | n.a. | n.a. |
| Fan | Nocifensive behaviors components evoked by brief laser pulses are mediated by C fibers | 2009 | neuroscience letters | 690 | 120 to 125 | pentobarbital | n.a. | n.a. | n.a. |
| Fang | The effect of different EEG derivations on sleep staging in rats: the frontal midline-parietal bipolar electrode for sleep scoring | 2009 | the journal of comparative neurology | 515 | 41 to 55 | pentobarbital | n.a. | n.a. | n.a. |
| Farias | Injury-related production of cysteinyl leukotrienes contributes to brain damage following experimental traumatic brain injury | 2009 | Journal of Neurotrauma | 26 | 1977 to 1986 | isoflurane | n.a. | n.a. | n.a. |
| Farias-Santos Rde | Exposure of developing well-nourished and malnourished rats to environmental heating facilitates cortical spreading depression propagation at adulthood | 2009 | physiology & behavior | 98 | 108 to 117 | urethane / chloralose | n.a. | n.a. | n.a. |
| Fedeli | The paraventricular nucleus of the hypothalamus is a neuroanatomical substrate for the inhibition of palatable food intake by neuropeptide S | 2009 | physiological measurement | 30 | 589 to 601 | tiletamine / zolazepam | n.a. | n.a. | n.a. |
| Fellin | Endogenous nonneuronal modulators of synaptic transmission control cortical slow oscillations in vivo | 2009 | neuroscience letters | 454 | 218 to 222 | urethane | n.a. | n.a. | n.a. |
| Fendt | Fear-reducing effects of intra-amygdala neuropeptide Y infusion in animal models of conditioned fear: an NPY Y1 receptor independent effect | 2009 | European journal of neuroscience | 30 | 1594 to 1602 | ketamine / xylazine | n.a. | n.a. | buprenorphine |
| Feng | The effect of clomipramine on wake/sleep and orexinergic expression in rats | 2009 | PNAS | 106 | 15037 to 15042 | pentobarbital | n.a. | n.a. | n.a. |
| Fenik | Differential localization of carbachol- and bicuculline-sensitive pontine sites for eliciting REM sleep-like effects in anesthetized rats | 2009 | Psychopharmacology | 206 | 291 to 301 | isoflurane / urethane / pancuronium bromide | n.a. | n.a. | n.a. |
| Fenoglio-Simeone | Anticonvulsant effects of the selective melatonin receptor agonist ramelteon | 2009 | journal of psychopharmacology | 23 | 559 to 566 | isoflurane | n.a. | n.a. | n.a. |
| Fenoglio-Simeone | Ketogenic diet treatment abolishes seizure periodicity and improves diurnal rhythmicity in epileptic Kcna1-null mice | 2009 | Journal of Sleep Research | 18 | 99 to 112 | isoflurane | n.a. | n.a. | n.a. |
| Ferreira-Silva | Modulatory role of locus coeruleus and estradiol on the stress response of female rats | 2009 | Epilepsy & Behavior | 16 | 52 to 57 | tribromoethanol | n.a. | n.a. | n.a. |
| Filipski | Circadian disruption in experimental cancer processes | 2009 | Epilepsia | 50 | 2027 to 2034 | n.r. | n.a. | n.a. | n.a. |
| Fisher | Sleep-promoting action of IIK7, a selective MT2 melatonin receptor agonist in the rat | 2009 | Endocrinology | 35 | 166 to 176 | ketamine / medetomidine | n.a. | n.a. | n.a. |
| Foerch | Rapid reversal of anticoagulation reduces hemorrhage volume in a mouse model of warfarin-associated intracerebral hemorrhage | 2009 | Journal of Cerebral Blood Flow & Metabolism | 29 | 1015 to 1021 | isoflurane | n.a. | n.a. | n.a. |
| Fogel | Evidence for 2-stage models of sleep and memory: learning-dependent changes in spindles and theta in rats | 2009 | Brain Research Bulletin | 79 | 445 to 451 | isoflurane | n.a. | n.a. | acetaminophen |
| Folbergrova | Posttreatment with group II metabotropic glutamate receptor agonist 2R,4R-4-aminopyrrolidine-2,4-dicarboxylate is only weakly effective on seizures in immature rats | 2009 | brain research | 1273 | 144 to 154 | ether | n.a. | n.a. | n.a. |
| Foley | Magnetic resonance imaging assessment of macrophage accumulation in mouse brain after experimental traumatic brain injury | 2009 | Journal of Neurotrauma | 26 | 1509 to 1519 | isoflurane | n.a. | n.a. | n.a. |
| Foti | Delivering multiple gene products in the brain from a single adeno-associated virus vector | 2009 | Gene Therapy | 16 | 1314 to 1319 | isoflurane | n.a. | n.a. | n.a. |
| Fournier | Decreased levels of disrupted-in-schizophrenia 1 (DISC1) are associated with expansion of the dentate granule cell layer in normal and kindled rats | 2009 | neuroscience letters | 455 | 134 to 139 | n.r. | n.a. | n.a. | n.a. |
| Fournier | Altered synapsin I immunoreactivity and fear behavior in male and female rats subjected to long-term amygdala kindling | 2009 | Behavioral Brain Research | 196 | 106 to 115 | pentobarbital | n.a. | n.a. | n.a. |
| Francois | Selective reorganization of GABAergic transmission in neonatal ventral hippocampal-lesioned rats | 2009 | International Journal of Neuropsychopharmacology | 12 | 1097 to 1110 | ketamine / xylazine | n.a. | n.a. | n.a. |
| Franklin | A single, moderate ethanol exposure alters extracellular dopamine levels and dopamine d receptor function in the nucleus accumbens of wistar rats | 2009 | Alcoholism, clinical and experimental research | 33 | 1721 to 1730 | isoflurane | n.a. | n.a. | n.a. |
| Frey | A novel apparatus for lateral fluid percussion injury in the rat | 2009 | Journal of Neuroscience Methods | 177 | 267 to 277 | isoflurane | n.a. | n.a. | n.a. |
| Friedman | Programmed acute electrical stimulation of ventral tegmental area alleviates depressive-like behavior,2009,,,Neuropsychopharmacology | 2009 | neuropsychopharmacology | 34 | 1057 to 1066 | chloral hydrate | n.a. | n.a. | n.a. |
| Friese | Sleep deprivation after septic insult increases mortality independent of age | 2009 | the journal of trauma | 66 | 50 to 54 | ketamine / xylazine | n.a. | n.a. | n.a. |
| Frilot | Magnetosensory function in rats: localization using positron emission tomography | 2009 | Synapse | 63 | 421 to 428 | isoflurane | n.a. | n.a. | n.a. |
| Fritsch | Pathological alterations in GABAergic interneurons and reduced tonic inhibition in the basolateral amygdala during epileptogenesis | 2009 | Neuroscience | 29 | 415 to 429 | ketamine / medetomidine | n.a. | n.a. | n.a. |
| Frye | Infusions of bicuculline to the ventral tegmental area attenuates sexual, exploratory, and anti-anxiety behavior of proestrous rats | 2009 | pharmacology, biochemistry and behavior | 93 | 474 to 481 | ketamine / xylazine | n.a. | n.a. | n.a. |
| Fu | Molecular hydrogen is protective against 6-hydroxydopamine-induced nigrostriatal degeneration in a rat model of Parkinson's disease | 2009 | neuroscience letters | 453 | 81 to 85 | n.r. | n.a. | n.a. | n.a. |
| Fujiki | Specificity of direct transition from wake to REM sleep in orexin/ataxin-3 transgenic narcoleptic mice | 2009 | Experimental Neurology | 217 | 46 to 54 | isoflurane | n.a. | n.a. | n.a. |
| Fukuda | Interleukin-1beta enhances susceptibility to hyperthermia-induced seizures in developing rats | 2009 | seizure | 18 | 211 to 214 | pentobarbital | n.a. | n.a. | n.a. |
| Fukushima | Ablation of NMDA receptors enhances the excitability of hippocampal CA3 neurons | 2009 | plos one | 4 | 3993 | urethane | n.a. | n.a. | n.a. |
| Fukushima | Metabolic and histologic effects of sodium pyruvate treatment in the rat after cortical contusion injury | 2009 | Journal of Neurotrauma | 26 | 1095 to 1110 | isoflurane | n.a. | n.a. | n.a. |
| Funke | Short-latency afferent inhibition varies with cortical state in rat somatosensory cortex | 2009 | neuroreport | 20 | 1313 to 1318 | urethane | n.a. | n.a. | n.a. |
| Gaillard | Anatomical and functional reconstruction of the nigrostriatal pathway by intranigral transplants | 2009 | Neurobiology of Disease | 35 | 477 to 488 | tribromoethanol | n.a. | n.a. | n.a. |
| Galati | The pharmacological blockade of medial forebrain bundle induces an acute pathological synchronization of the cortico-subthalamic nucleus-globus pallidus pathway | 2009 | journal of physiology | 587 | 4405 to 4423 | urethane | n.a. | n.a. | n.a. |
| Gallego | Continuous bilateral infusion of GABA in the dorsomedian nucleus of the thalamus elevates the generalized seizure threshold in amygdala-kindled rats | 2009 | seizure | 18 | 537 to 540 | ketamine / diazepam | n.a. | n.a. | n.a. |
| Gao | Anterior thalamic nucleus stimulation modulates regional cerebral metabolism: an FDG-MicroPET study in rats | 2009 | Neurobiology of Disease | 34 | 477 to 483 | chloral hydrate | n.a. | n.a. | n.a. |
| Gao | Conditional knockout of brain-derived neurotrophic factor in the hippocampus increases death of adult-born immature neurons following traumatic brain injury | 2009 | Journal of Neurotrauma | 26 | 1325 to 1335 | isoflurane | n.a. | n.a. | n.a. |
| Gao | Moderate traumatic brain injury promotes proliferation of quiescent neural progenitors in the adult hippocampus | 2009 | Experimental Neurology | 219 | 516 to 523 | isoflurane | n.a. | n.a. | n.a. |
| Garbayo | Effective GDNF brain delivery using microspheres--a promising strategy for Parkinson's disease | 2009 | journal of controlled release | 17 | 119 to 126 | ketamine / xylazine | n.a. | n.a. | n.a. |
| Garzon | Gz mediates the long-lasting desensitization of brain CB1 receptors and is essential for cross-tolerance with morphine | 2009 | molecular pain | 5 | 11 | ether | n.a. | n.a. | n.a. |
| Gass | The role of the basal forebrain adenosine receptors in sleep homeostasis | 2009 | NeuroReport | 20 | 1013 to 1018 | ketamine / medetomidine / diazepam | n.a. | n.a. | n.a. |
| Gilby | Chronic omega-3 supplementation in seizure-prone versus seizure-resistant rat strains: a cautionary tale | 2009 | Neuroscience | 163 | 750 to 758 | pentobarbital | n.a. | n.a. | n.a. |
| Gilby | Postnatal epigenetic influences on seizure susceptibility in seizure-prone versus seizure-resistant rat strains | 2009 | behavioral neuroscience | 123 | 337 to 346 | pentobarbital | n.a. | n.a. | acetaminophen, bupivacaine |
| Gill | Reductions in paradoxical sleep time in adult rats treated neonatally with low dose domoic acid | 2009 | behavioral Brain Research | 205 | 564 to 567 | midazolam / fluanisone | fentanyl | n.a. | carprofen |
| Gilmer | Early mitochondrial dysfunction after cortical contusion injury | 2009 | Journal of Neurotrauma | 26 | 1271 to 1280 | isoflurane | n.a. | n.a. | n.a. |
| Girardeau | Selective suppression of hippocampal ripples impairs spatial memory | 2009 | nature neuroscience | 12 | 1222 to1223 | n.r. | n.a. | n.a. | n.a. |
| Glavaski-Joksimovic | Reversal of dopaminergic degeneration in a parkinsonian rat following micrografting of human bone marrow-derived neural progenitors | 2009 | Cell Transplantation | 18 | 801 to 814 | isoflurane | n.a. | n.a. | lidocaine |
| Goard | Basal forebrain activation enhances cortical coding of natural scenes | 2009 | nature neuroscience | 12 | 1444 to 1449 | urethane | n.a. | n.a. | n.a. |
| Godukhin | The effects of interleukin-10 on the development of epileptiform activity in the hippocampus induced by transient hypoxia, bicuculline, and electrical kindling | 2009 | neuroscience and behavioral physiology | 39 | 625 to 631 | pentobarbital | n.a. | n.a. | n.a. |
| Goffin | Longitudinal microPET imaging of brain glucose metabolism in rat lithium-pilocarpine model of epilepsy | 2009 | experimental neurology | 217 | 205 to 209 | pentobarbital | n.a. | n.a. | n.a. |
| Gogichadze | Opioid system of the brain and ethanol | 2009 | Georgian Medical News | 4 | 60 to 64 | hexenal OR chloral hydrate | n.a. | n.a. | n.a. |
| Goldman | Arrhythmia in heart and brain: KCNQ1 mutations link epilepsy and sudden unexplained death | 2009 | scientific translational medicine | 1 | 2ra6 | tribromoethanol | n.a. | n.a. | n.a. |
| Golshani | Internally mediated developmental desynchronization of neocortical network activity | 2009 | The Journal of Neuroscience | 29 | 10890 to 10899 | isoflurane OR urethane | n.a. | n.a. | lidocaine |
| Gong | GABA transporter-1 activity modulates hippocampal theta oscillation and theta burst stimulation-induced long-term potentiation | 2009 | The Journal of Neuroscience | 29 | 15836 to 15845 | ketamine / medetomidine | n.a. | n.a. | n.a. |
| Gonzalez-Flores | Nitric oxide and ERK/MAPK mediation of estrous behavior induced by GnRH, PGE2 and db-cAMP in rats | 2009 | physiology & behavior | 96 | 606 to 612 | ketamine / xylazine | n.a. | n.a. | n.a. |
| Gonzalez-Trujano | Effect of repeated administration of Annona diversifolia Saff. (ilama) extracts and palmitone on rat amygdala kindling | 2009 | Epilepsy & Behavior | 16 | 590 to 595 | ketamine / xylazine | n.a. | n.a. | n.a. |
| Good | Control of synchronization of brain dynamics leads to control of epileptic seizures in rodents | 2009 | International Journal of neural systems | 19 | 173-196 | ketamine / xylazine / acepromazine | n.a. | n.a. | n.a. |
| Gottfried-Blackmore | Acute in vivo exposure to interferon-gamma enables resident brain dendritic cells to become effective antigen presenting cells | 2009 | PNAS | 106 | 20918 to 20923 | n.r. | n.a. | n.a. | n.a. |
| Gourley | Prelimbic cortex bdnf knock-down reduces instrumental responding in extinction | 2009 | Learning Memory | 16 | 756 to 760 | methyl butanol / tribromoethanol | n.a. | n.a. | n.a. |
| Goyagi | The combined neuroprotective effects of lidocaine and dexmedetomidine after transient forebrain ischemia in rats | 2009 | Acta Anaesthsiologica Scandinavica | 53 | 1176 to 1183 | halothane | n.a. | n.a. | n.a. |
| Gradinaru | Optical deconstruction of parkinsonian neural circuitry | 2009 | Howard Hughes Medical Institute | 324 | 354 to 359 | isoflurane | n.a. | n.a. | n.a. |
| Grasso | Neuroprotective effect of erythropoietin and darbepoetin alfa after experimental intracerebral hemorrhage | 2009 | Neurosurgery | 65 | 763 to 770 | ketamine / xylazine | n.a. | n.a. | n.a. |
| Greenwood | Seizures, enhanced excitation, and increased vesicle number in Lis1 mutant mice | 2009 | Annals of Neurology | 66 | 644 to 653 | n.r. | n.a. | n.a. | n.a. |
| Griesbach | Exercise-induced improvement in cognitive performance after traumatic brain injury in rats is dependent on BDNF activation | 2009 | Brain Research | 1288 | 105 to 115 | isoflurane | n.a. | n.a. | bupivacaine |
| Griesbach | Controlled contusion injury alters molecular systems associated with cognitive performance | 2009 | Journal of Neuroscience Research | 87 | 795 to 805 | isoflurane | n.a. | n.a. | bupivacaine |
| Gruner | The roles of dopamine transport inhibition and dopamine release facilitation in wake enhancement and rebound hypersomnolence induced by dopaminergic agents | 2009 | Sleep Journal | 32 | 1425 to 1438 | pentobarbital | lidocaine | n.a. | n.a. |
| Guedes | Sexual differentiation of cortical spreading depression propagation after acute and kindled audiogenic seizures in the Wistar audiogenic rat (WAR) | 2009 | Epilepsy Research | 83 | 207 to 214 | urethane / chloralose | n.a. | n.a. | n.a. |
| Guevara-Lopez | Effect of acute gouty arthritis on sleep patterns: a preclinical study | 2009 | European Journal of Pain | 13 | 146 to 153 | pentobarbital | n.a. | n.a. | n.a. |
| Guidine | Electroencephalographic evidence of brainstem recruitment during scorpion envenomation | 2009 | NeuroToxicology | 30 | 90 to 96 | halothane | n.a. | n.a. | n.a. |
| Guo | Self-assembling peptide nanofiber scaffold promotes the reconstruction of acutely injured brain | 2009 | Nanomedicine | 1549 | 345 to 351 | ketamine / xylazine | n.a. | n.a. | n.a. |
| Guo | Differential effects of acute and repeat dosing with the H3 antagonist GSK189254 on the sleep-wake cycle and narcoleptic episodes in Ox-/- mice | 2009 | British Journal of Pharmacology | 157 | 104 to 117 | isoflurane | n.a. | n.a. | n.a. |
| Guo | Correlation of CD34+ cells with tissue angiogenesis after traumatic brain injury in a rat model | 2009 | Journal of Neurotrauma | 26 | 1337 to 1344 | chloral hydrate | n.a. | n.a. | n.a. |
| Gurevicius | Genetic ablation of tenascin-C expression leads to abnormal hippocampal CA1 structure and electrical activity in vivo | 2009 | Hippocampus | 19 | 1232 to 1246 | pentobarbital / chloral hydrate | n.a. | n.a. | carprofen |
| Gurkoff | Acute neuroprotection to pilocarpine-induced seizures is not sustained after traumatic brain injury in the developing rat | 2009 | Neuroscience | 164 | 862 to 876 | isoflurane | n.a. | n.a. | n.a. |
| Habibi-Asl | Central administration of minocycline and riluzole prevents morphine-induced tolerance in rats | 2009 | pain mechanisms | 109 | 936 to 942 | pentobarbital | n.a. | n.a. | n.a. |
| Hadamitzky | Effects of acute intra-cerebral administration of the 5-HT(2A/C) receptor ligands DOI and ketanserin on impulse control in rats | 2009 | behavioral Brain Research | 204 | 88 to 92 | chloral hydrate | n.a. | n.a. | n.a. |
| Haddad | Endotoxin-mediated regulation of nuclear factor-kappaB nuclear translocation and activation in the hippocampus of the central nervous system: modulation by intracerebroventricular treatment with thymulin and the immunomodulatory role of the IkappaB-alpha/pIkappaB-alpha pathway | 2009 | neuroscience | 164 | 1509 to 1520 | n.r. | n.a. | n.a. | n.a. |
| Hahn | Survival and early functional integration of dopaminergic progenitor cells following transplantation in a rat model of Parkinson's disease | 2009 | journal of neuroscience research | 87 | 2006 to 2019 | n.r. | n.a. | n.a. | n.a. |
| Haj-ali | Intracerebroventricular insulin improves spatial learning and memory in male Wistar rats | 2009 | behavioral neuroscience | 123 | 1309 to 1314 | ketamine / xylazine | n.a. | n.a. | n.a. |
| Hakami | NMDA receptor hypofunction leads to generalized and persistent aberrant gamma oscillations independent of hyperlocomotion and the state of consciousness | 2009 | plos one | 4 | 6755 | ketamine / xylazine | n.a. | n.a. | n.a. |
| Halassa | Astrocytic modulation of sleep homeostasis and cognitive consequences of sleep loss | 2009 | Neuron | 61 | 213 to 219 | isoflurane | n.a. | n.a. | n.a. |
| Hamani | Bilateral anterior thalamic nucleus lesions are not protective against seizures in chronic pilocarpine epileptic rats | 2009 | stereotactic and functional neurosurgery | 87 | 143 to 147 | ketamine / xylazine | n.a. | n.a. | n.a. |
| Hamid | Effect of ouabain on sodium pump alpha-isoform expression in an animal model of mania | 2009 | progress in neuro-psychopharmacology & biological psychiatry | 33 | 1103 to 1106 | ketamine / xylazine | n.a. | n.a. | n.a. |
| Hampson | A wireless recording system that utilizes Bluetooth technology to transmit neural activity in freely moving animals | 2009 | Journal of Neuroscience Methods | 182 | 195 to 204 | n.r. | n.a. | n.a. | n.a. |
| Hangya | GABAergic neurons of the medial septum lead the hippocampal network during theta activity | 2009 | the journal of neuroscience | 29 | 8094 to 8102 | urethane | n.a. | n.a. | n.a. |
| Hanlon | Effects of skilled training on sleep slow wave activity and cortical gene expression in the rat | 2009 | Sleep Journal | 32 | 719 to 729 | isoflurane | n.a. | n.a. | n.a. |
| Hara | Involvement of extracellular ascorbate and iron in hydroxyl radical generation in rat striatum in carbon monoxide poisoning | 2009 | toxicology | 264 | 69 to 73 | pentobarbital | n.a. | n.a. | n.a. |
| Harris | Traumatic brain injury results in disparate regions of chondroitin sulfate proteoglycan expression that are temporally limited | 2009 | journal of neuroscience research | 87 | 2937 | isoflurane | n.a. | n.a. | bupivacaine |
| Hart | Systemic or intra-amygdala injection of a benzodiazepine (midazolam) impairs extinction but spares re-extinction of conditioned fear responses | 2009 | Learning Memory | 16 | 53 to 61 | ketamine / xylazine | n.a. | n.a. | procaine |
| Hartig | Triple fluorescence labelling of neuronal, glial and vascular markers revealing pathological alterations in various animal models | 2009 | Journal of Chemical Neuroanatomy | 37 | 128 to 138 | medetomidine / midazolam | fentanyl | n.a. | n.a. |
| Harting | Intravenous mesenchymal stem cell therapy for traumatic brain injury | 2009 | journal of neurosurgery | 110 | 1189 to 1197 | isoflurane | n.a. | n.a. | n.a. |
| Harting | Subacute neural stem cell therapy for traumatic brain injury | 2009 | journal of surgical research | 153 | 188 to 194 | isoflurane | n.a. | n.a. | n.a. |
| Hartings | Recovery of slow potentials in AC-coupled electrocorticography: application to spreading depolarizations in rat and human cerebral cortex | 2009 | Journal of Neurophysiology | 102 | 2563 to 2575 | isoflurane | n.a. | n.a. | n.a. |
| Harvey | Intracellular dynamics of hippocampal place cells during virtual navigation | 2009 | Nature | 461 | 941 to 946 | n.r. | n.a. | n.a. | n.a. |
| Hasan | How to keep the brain awake? The complex molecular pharmacogenetics of wake promotion | 2009 | neuropsychopharmacology | 34 | 1625 to 1640 | ketamine / xylazine | n.a. | n.a. | n.a. |
| Hassani | Discharge profiles of identified GABAergic in comparison to cholinergic and putative glutamatergic basal forebrain neurons across the sleep-wake cycle | 2009 | The Journal of Neuroscience | 29 | 11828 to 11840 | ketamine / xylazine / acepromazine | n.a. | n.a. | n.a. |
| Hayashi | Quantitative analyses of matrix metalloproteinase activity after traumatic brain injury in adult rats | 2009 | brain research | 1280 | 172 to 177 | ketamine / xylazine | buprenorphine | n.a. | n.a. |
| He | The transcriptional repressor DEC2 regulates sleep length in mammals | 2009 | science | 325 | 866 to 870 | isoflurane | n.a. | n.a. | n.a. |
| Heida | Separating kindling and LTP: lessons from studies of PKM zeta in developing and adult rats | 2009 | neuroscience letters | 453 | 229 to 232 | ketamine / xylazine | n.a. | n.a. | n.a. |
| Heile | Cerebral transplantation of encapsulated mesenchymal stem cells improves cellular pathology after experimental traumatic brain injury | 2009 | neuroscience letters | 463 | 176 to 181 | ketamine / medetomidine | n.a. | n.a. | n.a. |
| Hellier | NMDA receptor-mediated long-term alterations in epileptiform activity in experimental chronic epilepsy | 2009 | Neuropharmacology | 56 | 414 to 421 | isoflurane | n.a. | n.a. | buprenorphine |
| Henderson | Cortical kindling induces elevated levels of AMPA and GABA receptor subunit mRNA within the amygdala/piriform region and is associated with behavioral changes in the rat | 2009 | Epilepsy & Behavior | 16 | 404 to 410 | isoflurane | lidocaine | n.a. | n.a. |
| Hennige | Insulin-mediated cortical activity in the slow frequency range is diminished in obese mice and promotes physical inactivity | 2009 | Diabetologica | 52 | 2416 to 2424 | isoflurane | n.a. | n.a. | n.a. |
| Henshall | Electroencephalographic and behavioral convulsant effects of hydrobromide and hydrochloride salts of bupropion in conscious rodents | 2009 | neuropsychiatric disease and treatment | 5 | 189 to 206 | isoflurane | n.a. | n.a. | n.a. |
| Heredia-Lopez | A digital programmable telemetric system for recording extracellular action potentials | 2009 | behavior research methods | 41 | 352 to 358 | pentobarbital | n.a. | n.a. | n.a. |
| Hernandez-Gonzalez | Ethanol changes the electroencephalographic correlation of the ventral tegmental area and nucleus accumbens, components of the mesoaccumbens system in rats | 2009 | Pharmacology, Biochemistry and Behavior | 92 | 124 to 130 | pentobarbital | n.a. | n.a. | n.a. |
| Hessel | Phenotyping mouse chromosome substitution strains reveal multiple QTLs for febrile seizure susceptibility | 2009 | genes, brain and behavior | 8 | 248 to 255 | isoflurane | n.a. | n.a. | n.a. |
| Hipolito | Local salsolinol modulates dopamine extracellular levels from rat nucleus accumbens: shell/core differences | 2009 | neurochemistry international | 55 | 187 to 192 | chloral hydrate | n.a. | n.a. | n.a. |
[truncated: 422,668 more chars]
